# Abstracts of the Italian Society of Thoracic Endoscopy (SIET) 2024 Annual Congress ^†^

**DOI:** 10.3390/jcm13195954

**Published:** 2024-10-07

**Authors:** Carmelina Cristina Zirafa, Mohsen Ibrahim, Lorenzo Corbetta, Lorenzo Rosso, Piero Candoli, Beatrice Manfredini, Giovanni Galluccio, Cecilia Menna, Rocco Trisolini, Sara Ricciardi, Gaetano Romano, Giuseppe Cardillo, Franca Melfi, Federico Raveglia

**Affiliations:** 1 Minimally Invasive and Robotic Thoracic Surgery, Surgical, Medical, Molecular, and Critical Care Pathology Department, University Hospital of Pisa, 56124 Pisa, Italy; beatrice.manfredini91@gmail.com (B.M.); gaetano.romano@ao-pisa.toscana.it (G.R.); franca.melfi@unipi.it (F.M.); 2 Division of Thoracic Surgery, Sant’Andrea Hospital, Faculty of Medicine and Psychology, Sapienza University of Rome, 00185 Rome, Italy; mohsen.ibrahim@uniroma1.it (M.I.); cecilia.menna@uniroma1.it (C.M.); 3 Department of Experimental and Clinical Medicine, University of Florence, 50121 Firenze, Italy; lorenzo.corbetta@unifi.it; 4 Thoracic Surgery and Lung Transplant Unit, Fondazione Istituto di Ricovero e Cura a Carattere Scientifico Ca’ Granda Ospedale Maggiore Policlinico, 20900 Milano, Italy; lorenzo.rosso@policlinico.mi.it; 5 Interventional Pulmonology Unit, IRCCS Azienda Ospedaliero-Universitaria Policlinico di Sant’Orsola, 40138 Bologna, Italy; piero.candoli@aosp.bo.it; 6 Unit of Thoracic Endoscopy, San Camillo Forlanini Hospital, 00100 Rome, Italy; ggalluccio@scamilloforlanini.rm.it; 7 Interventional Pulmonology Division, Department of Neurosciences, Sense Organs and Thorax, Fondazione Policlinico Universitario Agostino Gemelli IRCCS, 00168 Rome, Italy; rocco.trisolini@policlinicogemelli.it; 8 Unit of Thoracic Surgery, Azienda Ospedaliera San Camillo Forlanini, 00152 Rome, Italy; sricciardi2@scamilloforlanini.rm.it (S.R.); gcardillo@scamilloforlanini.rm.it (G.C.); 9 Department of Thoracic Surgery, IRCCS San Gerardo dei Tintori, 20900 Monza, Italy

**Keywords:** lung cancer, screening, trachea, lung airways, mediastinum, thoracic disease, trauma, endoscopic strategy

## Abstract

We are pleased to introduce the abstracts of the XXIII National Congress of the Italian Society of Thoracic Endoscopy (SIET), which will be held in Florence from 17 to 19 October 2024. The principal objectives of SIET are to (1) Promote research and innovation in the fields of thoracic surgery and endoscopy, facilitating the development and implementation of innovative techniques and technologies; (2) Provide education and training for surgeons, endoscopists, pulmonologists and other related specialties; and (3) Facilitate the exchange of knowledge with the aim of creating a cohesive and active scientific community. The Congress will address the integration of traditional surgical and endoscopic techniques with emerging technologies, with the goal of promoting innovation and education among professionals. The theme of integration will be explored throughout the programme, with a particular focus on the collaborative efforts of different medical specialties to improve patient outcomes. This event will host a multidisciplinary cohort comprising thoracic surgeons, endoscopists, pulmonologists, oncologists, pathologists, radiologists and anaesthetists, who will assume a pivotal role in the multidisciplinary sessions of the scientific programme. The Congress will include several core areas of expertise, including lung cancer, interventional endoscopy, pathology, and upper airway reconstruction. Emphasis will be placed on both the theoretical aspects of these subjects and their practical applications in patient care. The theme of integration will be explored throughout the programme, with particular attention on the impact of recent technological developments in the fields of thoracic surgery and endoscopy. Additionally, the Congress will examine the contributions of allied health professionals, including nurses, physiotherapists, and speech pathologists, to patient care.

## 1. Conference Abstracts

### 1.1. Abstract 1 (Oral Presentation): Feasibility and Safety of Transvascular Endosonography: A Case Series

**Cristina Albrici, Carmine Salerni, Simone Contino, Paolo Carlucci, Stefano Centanni and Michele Mondoni**

Respiratory Unit, ASST Santi Paolo e Carlo, San Paolo Hospital, Department of Health Sciences, University of Milan, Milan, Italy

Background: Endosonography is the preferred technique for diagnosing mediastinal lymphadenopathy and the gold standard for mediastinal staging of lung cancer. The presence of major vessels interposed between the airways/oesophagus and the target lesion has long been regarded as a concern for major bleeding. Recent scientific evidence, mostly based on case reports/series, have demonstrated the feasibility and the safety of traversing major thoracic vessels to sample lymph nodes or lung lesions. However, most of the reported cases were based on EBUS-TBNA.

Methodology: We herein present a case series of six transvascular endosonographies in which three patients underwent EUS-B-FNA without any complication (Figure 1). 

Demographic, clinical and procedural data were collected and summarized in Table 1. 

Results: Diagnosis of malignancy was obtained by endoscopic procedures in 5 out of 6 patients with an overall diagnostic yield of 83.3%, in accordance with recent meta-analyses reporting an overall accuracy of 82.1–85%. 3.5 (0.5) mean (standard deviation) needle passes were performed. No major complications were noted. A mild endobronchial bleeding was recorded in a single case. No haematomas or pseudo-aneurysms were reported. All the patients were monitored for at least three hours after the procedure and contacted in the following days to record any complication. 

Transvascular approach should be considered only when the result of the biopsy will significantly impact patients’ management and outcomes even though literature reports a low incidence of major adverse events (2.7%) with an incidence of bleeding of any severity of 1.4%. Indeed, possible complications following the puncture of intrathoracic vessels may be represented by fatal haemorrhage, intramural hematoma, pneumomediastinum and tracheal stenosis due to hematoma compression. 

Conclusions: Transvascular approach during conventional EUS-FNA has been frequently reported in the literature but to our knowledge, only one case report is available on transvascular EUS-B-FNA. In this case series we confirm the good accuracy and safety of transvascular endosonography of mediastinal lymph nodes and pulmonary lesions. We first demonstrate, in a larger number of patients, that transvascular EUS-B-FNA is a useful and safe technique. 

Further research, based on a larger cohort of patients, are warranted to validate our findings. 

### 1.2. Abstract 2 (Oral Presentation): Diagnostic Role of Circulating Free DNA and Circulating Tumor DNA in Pulmonary Ground Glass Nodules: A Pilot Study

**Massimiliano Bassi ^1^, Beatrice Zacchini ^1^, Gianluigi De Renzi ^2^, Michela De Meo ^2^, Caterina Chiappetta ^3^, Francesco Ferrante ^1^, Rita Vaz Sousa ^1^, Anastasia Centofanti ^1^, Antonio Pio Evangelista ^1^, Valerio Sebastianelli ^1^, Francesco M. Mattoccia ^1^, Camilla Poggi ^1^, Daniele Diso ^1^, Tiziano De Giacomo ^1^, Federico Venuta ^1^, Paola Gazzaniga ^2^ and Marco Anile ^1^**

1. UOC Chirurgia Toracica e Trapianti di Polmone, Dipartimento di Chirurgia Generale e Specialistica, Sapienza Università di Roma
2. Dipartimento di Medicina Molecolare, Dipartimento di Chirurgia Generale e Specialistica, Sapienza Università di Roma
3. Dipartimento di Scienze Medico-Chirurgiche e Biotecnologie, Sapienza Università di Roma

Background: Pulmonary Ground Glass Nodules (GGNs) are frequently encountered in daily clinical practice due to the improvement of radiological tools. Most are benign and transient inflammatory entities, but up to 37% of that are associated with early-stage lung cancer. Long-term radiological follow-up is currently the gold-standard for a differential diagnosis. The aim of this pilot study is a preliminary evaluation of the diagnostic role of circulating free DNA (cfDNA) and circulating tumor DNA (ctDNA) in peripheral blood of patients with radiological findings of GGNs.

Methodology: Ten consecutive patients (M:F = 1:1, age 68.1 ± 10.8 years) with finding of GGN on CT scan were prospectively enrolled in the study. At the first visit, 20 mL of peripheral venous blood was collected and the plasma was stored at −80 °C after centrifugation. The cfDNA extracted from plasma was quantified using a diagnostic kit, the EasyPGX^®^ (Diatech Pharmacogenetics, Milano, Italy). A targeted approach was used for the detection of ctDNA, using Myriapod^®^ NGS Cancer Panel DNA. Patients underwent radiological follow up or histological sample following international guidelines. The radiological/histological diagnosis was compared with the DNA data to evaluate a possible diagnostic role in malignant GGNs. Iin addition, ctDNA results were compared with histological sample to assess a possible correlation in gene mutations. 

Results: Six patients had a histological diagnosis of malignant GGN, while 4 patients had a radiological/histological diagnosis of inflammatory nodule. Pure GGNs were more frequently benign than mixed GGNs (*p*-value = 0.0228). The average amount of cfDNA was 1.23 ± 1.68 ng/μL per patient. Patients with a malignant GGN had lower levels of cfDNA in the peripheral blood than patients with inflammatory GGNs (0.613 vs. 2.156 ng/μL; *p* = 0.031).

Nine patients had reliable results in the ctDNA analysis. Of the 17 genes evaluated, the most frequently mutated genes were HRAS and KIT (40%), followed by NRAS (30%), IDH-2 (20%), RET (20%) and PIK3CA (20%). However, no concordant genes were found between ctDNA and tissue analysis.

Conclusions: According to our preliminary analysis, lung cancer in its very early stage does not release relevant amounts of ctDNA into the peripheral blood. Therefore, ctDNA detection does not currently appear to play a role in the early diagnosis of lung cancer in patients with GGN, at present. However, in our sample, malignant GGNs showed lower levels of cfDNA than patients with inflammatory disease. This may open new lines of future research.

### 1.3. Abstract 3 (Oral Presentation): Intraoperative Ultrasound Detection of Pulmonary Nodule during Robotic-Assisted Thoracic Surgery Resection

**Sebastiano Angelo Bastone ^1,2^, Federico Tacconi ^1^, Chiara Combattelli ^1^, Alexandro Patirelis ^1^, Cristiano Casciani ^1^, Karan Kumar ^1^, Raul Randazzo ^1^, Luciano Cialì Sposato ^1^ and Vincenzo Ambrogi ^1^**

1. Thoracic Surgery Unit, Tor Vergata University Policlynic, Viale Oxford 81, 00133 Rome, Italy
2. PhD program in Applied Medical and Surgical Sciences, Department of Surgical Sciences, Tor Vergata University of Rome, Via Montpellier 2, 00133 Rome, Italy

Background: Intraoperative detection of deeply-located pulmonary nodules might be challenging in totally robotic-assisted thoracic surgery (RATS) without utility incision, due to loss of tactile feedback. This study was aimed at analyzing sensibility of Intraoperative Lung Ultrasound (ILU) in this setting. 

Methodology: Patients with solid or part-solid pulmonary nodules distant >1 cm from visceral pleura were scheduled for RATS lobectomy with intraoperative frozen section. Lung nodules were detected by means of a dedicated ultrasound probe, after gentle retraction and immobilization of the involved lung region. The target area was marked through electrocautery device, and resected with robotic stapler after ultrasound confirmation of adequate stapler apposition. 

Results: The ILU was used in 26 patients. Median nodule diameter was 12 mm (IQR 9–14 mm). Success rate for nodule detection was 96.1% (25/26 patients). In all these patients, presence of a nodule consistent with CT-findings was confirmed at frozen section, with median tumor-free margin of 11 mm (IQR 9–13 mm). Lobectomy was performed in 23 patients. In the other ones the procedure was interrupted after frozen section because of indolent lesion (*N* = 1) or intolerance to one-lung ventilation (*N* = 1). Median time for nodule detection was 12 min (IQR 10–15 min). 

Conclusions: In our experience, ILU was successful to detect deeply-located nodules during RATS lung resection. In this way, more patients might be scheduled for upfront RATS surgery thereby avoiding preop tissue diagnosis or interventional detection techniques such as metal coil placement or methylene blue injection. 

### 1.4. Abstract 4 (Oral Presentation): Malignant Obstruction of Central Airways: Interventional Bronchoscopy for Symptom Palliation

**Sara Cagnetti, Gianluca Ancona, Casimiro Eugenio Giorgetta, Francesco Inzirillo, Eugenio Ravalli, Giuseppe Naldi and Paolo Scanagatta**

Division of Thoracic Surgery, ASST Valtellina e Alto Lario, “Eugenio Morelli” Hospital, 23035 Sondalo, Italy

Background: The number of long-term cancer survivors is increasing and so palliative treatments leading to quality-of-life improvement. However, even the newest therapies are rarely able to resolve the airway obstruction and the related symptoms in patients with obstructive bronchial tumors. For this reason, the bronchoscopic treatment of obstructive lesions, especially in the event of obstruction of the central airways become increasingly important.

Methodology: Between June 2005 and December 2023, 416 consecutive patients were treated in our Thoracic Endoscopy Unit. There were 213 males (51%) and 203 (48%) females, aged 22 to 86 years (mean age 64.8 years). Overall, 86 (20.7%) patients were affected by benign lesions (61 post tracheostomy stenosis or post prolonged intubation, 5 foreign body, 5 tracheomalacia one of which by Mounier-Kuhn Syndrome, 4 tracheobronchial amyloidosis and 11 bronchopleural fistulas). Conversely, 330 (79.3%) patients had central airway malignant obstructions. These lesions involved the trachea in 96 patients (15 proximal, 19 mid and 62 distal trachea or carina), the left main bronchus in 67, the right main bronchus in 54, the bronchus intermedius in 43 and the lobar bronchi in 61.

Results: An average of 1235 procedure per patient were performed (64 patients received more then 1 procedure in order to maintain airway patency). In 24 patients (5.9%) the procedure has been performed for persistent major airway obstruction due to small cell lung cancer despite chemotherapy. Rigid Bronchoscopies were 372 (301 laser and mechanical ablation, 44 with ballon dilatation association and 27 only stent removal). In 141 procedures the stents have been placed to preserve airway patency. Airway rupture without septic sequelae was observed in 8 patient (1.9%) whereas in only 1 patient (0.25%) the procedure has been complicated by massive haemorrhage and perioperative death.

Conclusions: Rigid bronchoscopy play a key role in malignant airway obstructions as palliative procedure which allows a more appropriate control of any major bleeding. In patients with endobronchial tumors in advanced stage, the final aim of all bronchoscopic procedures is the resolution of respiratory failure as well as the improvement of the quality of in the last stages of life.

### 1.5. Abstract 5 (Oral Presentation): Sublobar Lung Resections: A Retrospective, Comparative Analysis of Open Thoracotomy, Vats and Rats Approaches for Lung Segmentectomy

**Chiara Catelli ^1^, Miriana D’Alessandro ^2^, Federico Mathieu ^3^, Susanna Guerrini ^4^, Piero Paladini ^3^ and Luca Luzzi ^1^**

1. Lung Transplant Unit, Department of Medical, Surgical and Neuro Sciences, Azienda Ospedaliero-Universitaria Senese, University of Siena, 53100 Siena, Italy
2. Respiratory Diseases Unit, Department of Medicine, Surgery and Neurosciences, University of Siena, 53100 Siena, Italy
3. Thoracic Surgery Unit, Department of Medical, Surgical and Neuroscience Sciences, University Hospital of Siena, 53100 Siena, Italy
4. Diagnostic Imaging Unit, Department of Medical Sciences, University of Siena, Azienda Ospedaliero-Universitaria Senese, Siena, Italy

Background: This study aims to evaluate short- and long-term outcomes of patients undergoing segmentectomy for lung cancer using Open thoracotomy, VATS, and RATS techniques.

Methodology: Patients (*n* = 157) who underwent lung segmentectomy from 2015 to 2024 at the Thoracic Surgery of Siena were retrospectively enrolled and divided into three groups based on the surgical approach: open thoracotomy (Group 1, *n* = 60), VATS (Group 2, *n* = 58), and RATS (Group 3, *n* = 39). Intraoperative and perioperative outcomes were compared. In patients affected by NSCLC, long-term outcomes (90-day mortality, 1-year and 2-year OS, disease recurrence) were compared.

Results: The conversion rate to open surgery was 23.4% for VATS and 7.3% for RATS (*p* = 0.0417). ICU-stay was shorter for patients undergoing VATS than open surgery (*p* = 0.03); no difference was found between VATS and RATS (*p* = 0.446). Pleural effusion on first post-operative day (pod) was lower in RATS than VATS (*p* = 0.0006) and open surgery (*p* < 0.0001). Pleural effusion on second pod was lower for RATS than open surgery (*p* = 0.002) but not compared to VATS (*p* = 0.12). The maximum VAS score was lower in RATS than open surgery (*p* = 0.016) and VATS (*p* = 0.013). Surgery time was longer for RATS than open (*p* = 0.0014) and VATS (*p* = 0.0128). No differences were found in length of hospital stay, post-operative complications, and day of drain removal. In Group 1, a lower number of lymph nodes were removed compared to VATS (*p* = 0.01) and RATS (*p* = 0.0052), while no differences were found between the latter two techniques. A higher number of lymph node stations were removed in the VATS group compared to open surgery (*p* = 0.0064), while no difference was found with RATS (*p* = 0.924). In the subgroup of patients undergoing segmentectomy for NSCLC (*n* = 106), the median follow-up was 25 months. The 90-day survival rate was 97.4%, with 4 patients (2.54%) died after open surgery (*p* = 0.0362). The 1-year OS was 95% (*p* = 0.02) and the 2-year OS was 87.6% (Figure 2).

No deaths were recorded in the RATS group. The number of lymph nodes retrieved was higher in the VATS (*p* = 0.015) and RATS (*p* = 0.06) than open thoracotomy group. No differences were found in disease recurrence.

Conclusions: Minimally invasive surgery entails reduced ICU-stay, less post-operative pain, less pleural effusion, a higher number of removed lymph nodes. RATS segmentectomy is highly reliable, with a lower conversion rate to open thoracotomy, and no differences in post-operative complication rates with VATS. In patients with NSCLC, RATS has better 1- and 2-years OS compared to VATS and open surgery, but it is still burdened by prolonged operating times.

### 1.6. Abstract 6 (Oral Presentation): Usefulness of Stent in Central Airways Malignancy for Palliative or Bridging Purposes: A Five-Year’s Experience

**Serena Conforti, Arash Astaneh, Marco Reda, Luca Pogliani, Giacomo Grisorio and Massimo Torre**

Department of Thoracic Surgery, ASST Grande Ospedale Metropolitano Niguarda, Milano, Italy

Background: Malignant central airway obstruction is a serious, life-threatening complication of several disease of both intrathoracic and extra thoracic tumors that impact performance status and access to systematic oncological therapies. Central airways obstruction was found in 13% of patients newly diagnosed. The aim of this study is to review clinical presentation, perioperative complications, and access to further oncological treatment in patients undergone bronchoscopic desobstruction procedures at our institution. We focus bronchoscopic tools available especially on tracheobronchial stent placement. 

Methodology: We retrospectively revised clinical report of a continuous series of patients underwent rigid bronchoscopy for malignant central airways obstruction in the last 5 years at our institution. 

Results: Between 2019 and 2023, 168 patients underwent rigid bronchoscopy for malignant airway obstruction; all procedures were performed in OR under deep sedation. 48% of patients were female; mean age was 65.8 (18–89); 30.6% of procedures were urgent for acute respiratory distress or airway bleeding. Squamous histology was the most frequent (31.5%), followed by adenocarcinoma and SCLC. Metastatic tumors proved to be more heterogeneous with bowel and renal metastasis (half of the histology). All patients underwent disostruction: in 139 patients (86.7%) coring manoeuvres were performed; in 48% of procedures laser ablative tools was used for cytoreduction and hemostasis; biopsy was performed in 128 patients. 61% of patients were considered eligible for auto-expandible stent. We frequently used the Y-shaped (Innova Medica, MicroTech, Ancona, Italy). Only 2 major intraoperative bleeding occurred and 4 displacements withing the first 5 days that required replacement. Considering we have solid information about only 67% of patients after first month of follow-up, 51 patients went on to further CT/RT treatment while 16 patients were addressed only to supportive care. Only in 8 cases we were able to remove the stent due to successful recanalization and disease control, within a mean time of 193 days (27–882) from the initial procedure.

Conclusions: Central airway obstruction may occur either due to endoluminal disease, extrinsic compression, or a combination of both; recanalization manoeuvres and stent placement are effective and safe either with palliative intent or bridge to systemic or local treatment. Its safety and efficacy make stent placement an optimal procedure for secure airways patency and stabilize critically ill patients allowing subsequent revaluation of disease and general conditions for further treatments. In our experience these procedures should be performed in a high-volume endoscopic center by thoracic surgeon or an expert team familiar with rigid bronchoscopy and airway pathology.

### 1.7. Abstract 7 (Oral Presentation): Robotic Surgery in Diagnosing Mediastinal Lymphoma Relapses: A Prospective Study

**Giacomo Cusumano ^1^, Rita Aulino ^1^; Giovanna Motta ^2^, Francesco Borrata ^1^, Alessandra Criscione ^1^, Damiano Calvo ^1^, Emanuele Fontana ^1^, Mariapia Gangemi ^1^, Maurizio Mannino ^1^, Diomira Tabacco ^1^, Marco Cavaleri ^3^, Francesco Di Raimondo ^2^ and Alberto Terminella ^1^**

1. UOC Chirurgia toracica, Azienda ospedaliero universitario Policlinico-San Marco, Catania, Italy
2. Unità operativa di Ematologia 1, Azienda ospedaliero universitario Policlinico-San Marco, Catania, Italy
3. Servizio di anestesia 1, Azienda ospedaliero universitario Policlinico-San Marco, Catania, Italy

Background: The onset or persistence of anterior mediastinal masses in lymphoma patients undergoing chemotherapy often indicates relapse or persistent disease. Post-chemotherapy thymic hyperplasia can also occur in the same location. CT and FDG-PET scans may be inconclusive for differential diagnosis, necessitating tissue confirmation to avoid overtreatment. The effectiveness of percutaneous or open biopsies depends on the lesion’s location and size, and scar tissue from chemotherapy. Additionally, cytohistological sampling may be inadequate for onco-hematological diagnostics. Robotic surgery is an effective tool for accessing any mediastinal site and is the gold standard in treating mediastinal pathologies. This study explores the use of robotics in invasive diagnostics for persistent or recurrent mediastinal lymphoma, evaluating short-term outcomes, complications, and the ability to obtain representative tissue samples. It compares results with non-invasive techniques like CT and PET, assessing if radiological and metabolic parameters can more reliably predict recurrence or persistent disease rather than thymic hyperplasia or chronic inflammation.

Methodology: This prospective observational study was conducted at the University Hospital of Catania. The analysis focused on patients with a clinical diagnosis of suspected recurrence or persistence of lymphoma with a unique mediastinal location. Data on initial diagnosis and first-line treatment, including clinical and laboratory exams, CT, bone marrow biopsy, and FDG-PET, were collected. All patients underwent a post-treatment FDG-PET scan; those with a positive test were evaluated with a biopsy of the positive lesion. Metabolic response to therapy was assessed using the Deauville criteria. Surgical data, postoperative outcomes, and pathological issues were collected retrospectively. All procedures were performed using the Xi Da Vinci Robotic System (Intuitive, Sunnyvale, CA, USA), guided by PET uptake area.

Results: The study included 20 patients, their clinical characteristics are reported in Table 2. All interventions were performed robotically without the need for conversion and without intraoperative complications. Total thymectomy was performed in 2 cases (10%), while 18 cases (90%) had partial thymectomy. The average postoperative hospital stay was 3.4 ± 0.68 days, with no postoperative mortality or major complications. One patient (5%) experienced a minor complication (wound dehiscence). Pathological analysis revealed relapse in 9 cases (45%). The comparison of PET-derived metabolic parameters and relapse detection showed a significant association with Deauville 5 (Pearson Chi2 12.7; *p*-value < 0.001). An increase in the SUV max/hepatic mean SUV ratio also showed a significant correlation (*p*-value = 0.003).

Conclusions: This study confirms the importance of surgical histological typing and suggests robotic surgery as a promising technique for diagnosing lymphoma relapses, with significant benefits in safety and diagnostic accuracy. Deauville score 5 and the SUV max/hepatic mean SUV ratio are reliable predictors of relapse. The study emphasizes the importance of histological confirmation in the presence of suspicious PET uptakes.

### 1.8. Abstract 8 (Oral Presentation): Management of Endobronchial Foreign Bodies Removed with Rigid Bronchoscopy

**Vito D’Agnano ^1^, Umberto Masi ^1^, Cristiano Cesaro ^2^, Roberta Cianci ^1^, Ilaria Menichini ^3^, Andrea Bianco ^1^ and Giovanni Galluccio ^3^**

1. Department of Translational Medicine, University of Campania “L. Vanvitelli”, Naples, Italy
2. Bronchology Unit, Monaldi Hospital, Naples, Italy
3. Center of Thoracic Endoscopy and Interventional Pulmonology, Regina Apostolorum Hospital, Albano Laziale, Italy

Background: The aim of this study is to evaluate the effectiveness and safety of rigid bronchoscopy in the removal of endobronchial foreign bodies, which are medical emergencies that can lead to severe respiratory complications such as airway obstruction, lung infections, and respiratory failure.

Methodology: We conducted a retrospective analysis on a cohort of patients with diagnosed endobronchial foreign bodies treated at our institution between 2018 and 2021. The presence of foreign bodies was confirmed through radiologic imaging (chest X-ray or CT scan) and preliminary diagnostic flexible bronchoscopy. All procedures were performed under general anesthesia. Various instruments, including biopsy forceps, retrieval baskets, and suction devices, were used depending on the size, shape, and location of the foreign body. Statistical analysis was performed using descriptive statistics and frequency analysis.

Results: We enrolled 20 patient, 12 male e 8 female, with a mean age of 35 years (range: 2–65 years). Common presenting symptoms included cough (75%), dyspnoea (60%), stridor (40%), hemoptysis (20%), asphyxia (15%). The main location of the foreign bodies was right main bronchus (40%), followed by left main bronchus (35%) and trachea (25%). In 50% of patients (*n* = 10) the foreign body was food material, in 35% (*n* = 7) non-food material, in 15% (*n* = 3) organic material. The removal of foreign bodies was successful in all 20 cases using rigid bronchoscopy, with no instances of failure or the need for thoracic surgery. The average duration of the procedure was 45 min (range: 20–90 min). Significant respiratory improvement was observed in 18 patients (90%). Postoperative recovery was swift, with most patients discharged within 48 h. No serious adverse events were associated with the procedure. Minor complications such as mild bleeding and airway irritation were managed effectively during the hospital stay.

Conclusions: Rigid bronchoscopy is a highly effective and safe method for the removal of endobronchial foreign bodies. Our experience with 20 patients demonstrated a 100% success rate with minimal complications. This technique offers direct visualization and precise control, making it the gold standard for managing this condition. Timely and adequate management of airway foreign bodies is crucial to prevent serious complications and improve clinical outcomes. 

### 1.9. Abstract 9 (Oral Presentation): Prognostic Factors and Clinical Outcomes of Surgical Treatment of Major Thoracic Trauma

**Federica Danuzzo ^1,2^, Maria Chiara Sibilia ^1,2^, Francesca Spinelli ^1,2^, Andrea Cara ^2^, Enrico Mario Cassina ^2^, Lidia Libretti ^2^, Emanuele Pirondini ^2^, Federico Raveglia ^2^, Antonio Tuoro ^2^, Sara Vaquer ^1,2^ and Francesco Petrella ^2^**

1. University of Milan, Milan, Italy
2. Department of Thoracic Surgery, Fondazione IRCCS San Gerardo dei Tintori, 20900 Monza, Italy

Background: Major thoracic trauma represents a life-threatening condition, requiring a prompt multidisciplinary approach and appropriate pathways for effective recovery. While acute morbidity and mortality are well known outcomes in thoracic traumatized patients, long-term quality of life in patients surviving surgical treatment has not been widely investigated before. Although it is well known that earlier interventions in post-traumatic scenario provide the greatest benefit for the patient, an effective method to predict the clinical courses of these patients is not yet available. The aim of the present paper is to assess prognostic factors and to foresee clinical outcomes of patients with major thoracic trauma treated by surgery.

Methodology: This was a single-center, retrospective and observational study: between November 2016 and November 2023, thirty-two patients were operated on in our department because of thoracic trauma. Thoracic trauma was defined as any form of physical injury to the chest including the ribs, sternum, heart, lungs and any other organ within the chest, with or without extra-thoracic concurrent trauma. Among them 3 were lost to follow up because of logistic reasons (foreign patients living abroad) and one was still admitted at the time this article was written. Data were therefore extracted from 28 operated patients. Age, sex, comorbidities, location and extent of thoracic trauma, Injury Severity Score (ISS), Abbreviated Injury Scale (AIS), Organ Injury Scale (OIS), intra and extra-thoracic organ involvement, mechanism of injury, type of surgical procedure, postoperative com-plications, ICU and total length of stay, immediate clinical outcomes and long-term quality of life—evaluated through the EQ-5D-3L scale and Numeric Rate Pain Score (NPRS)—were collected for each patient.

Results: EQ-5D-3L exhibited a significant negative correlation with pain; Injury Severity Score (ISS) emerged as a predictor of prolonged intensive care unit (ICU) stay and, consequently, overall hospitalization. The Log-Rank test was conducted to assess the quality of life by arbitrarily stratifying EQ-5D-3L into “good” (>0.50) and “bad” (<0.49) categories based on AIS (Abbreviated Injury Scale) and ISS. Results indicated no significant difference in EQ-5D-3L among patients with thoracic trauma based on AIS (*p* = 0.55), but a significant difference was observed in relation to ISS (*p* = 0.000011). 

Conclusions: We observed that ISS—rather than AIS—is correlated with EQ-5D-3L and emerged as the best prognostic factor, in terms of long-term quality of life in patients surviving major thoracic trauma undergoing surgical treatment.

### 1.10. Abstract 10 (Oral Presentation): Initial Experience of Ultrathin Cryobiopsy Combined with Lung Vision Platform for Pulmonary Nodules

**Thomas Galasso ^1^, Lorenzo Petroni ^2^, Gian Piero Bandelli ^1^, Marco Ferrari ^1^, Martina Ferioli ^1^, Filippo Natali ^1^ and Piero Candoli ^1^**

1. Interventional Pulmonology Unit, IRCCS Azienda Ospedaliero-Universitaria di Bologna, Bologna Italy
2. Università degli Studi di Firenze, Scuola di Specializzazione Malattie dell’Apparato Respiratorio, Firenze Italy

Background: Diagnosing peripheral lung nodules (PLNs) via bronchoscopy is a formidable challenge in interventional pulmonology, often necessitating the concurrent use of multiple diagnostic tools. Transbronchial cryobiopsy offers an advancement over traditional forceps biopsies by securing larger, higher-quality tissue samples. This study examines the diagnostic yield and safety of an ultrathin cryoprobe used in conjunction with an ultrathin bronchoscope and the Lung Vision platform (LV, Body Vision Medical LTD, Campbell, CA, USA), which integrates navigation and augmented fluoroscopy through digital tomosynthesis and intraoperative real-time imaging.

Methodology: A retrospective analysis was performed on patients who underwent bronchoscopies for suspected PLNs at our institution from January to May 2024. Procedures were conducted under deep sedation using a laryngeal mask without muscle paralysis, employing a 3.0 mm bronchoscope (BF-MP 190 F, Olympus, Tokyo, Japan) paired with the LV system for precise lesion navigation. Localization was confirmed via radial endobronchial ultrasound or LV “tool in lesion”. Tissue samples were collected with a 1.1 mm ultrathin cryoprobe under a 6-s freezing protocol, guided by LV-enhanced fluoroscopy. The “two-scope technique” outlined by Nakai T et al. was adopted, involving a swift switch to a standard caliber bronchoscope post-cryoprobe removal for any necessary bleeding control and endoscopic treatment [1].

The primary endpoints were the diagnostic yield and complications rate, including bleeding (according to Nashville scale) and pneumothorax. A specimen was deemed diagnostic only if it confirmed a malignancy or a specific non-malignant condition (e.g., hyphae).

Results: The cohort consisted of 27 patients with 28 lesions, predominantly solid (18/28) and mainly located in the upper lobes (75%) or the median lung third (50%). Lesion size averaged 16.5 mm (range: 5–30 mm). A bronchus sign was evident in 24 of 28 lesions (12 inside, 12 adjacent). Most lesions (23/28) had an intermediate risk of malignancy per the Brock model. The diagnostic yield was 71.4%. Bleeding complications were reported in 21% of procedures (mild in 2, moderate in 4), with no occurrences of severe bleeding or pneumothorax.

Conclusions: The integration of an ultrathin cryoprobe with the Lung Vision system and the two-scope technique provided a highly favorable safety profile and significant diagnostic yield, confirming its value as a minimally invasive approach for the early detection and detailed characterization of lung nodules. This initial experience highlights the potential for broader adoption in clinical practice.

### 1.11. Abstract 11 (Oral Presentation): Early-Stage Lung Cancer Behavior in Caucasian Race: Does Sex Make a Difference?

**Delia Giovanniello ^1,2^, Sara Ricciardi ^1,3^, Anastasia Centofanti ^1,2^, Eleonora Coviello ^1,4^, Massimo Osvaldo Jaus ^1^, Marco Di Martino ^1^, Luigi Carbone ^1^, Francesco Carleo ^1^, Lorenzo Salvadori ^1^, Sara Mantovani ^1^, Andrea Tornese ^1^, Stefano Treggiari ^1^ and Giuseppe Cardillo ^1^**

1. San Camillo Forlanini Hospital, Rome, Italy
2. University of Rome, Rome, Italy
3. University of Bologna, Bologna, Italy
4. University of Perugia, Perugia, Italy

Background: Over the past decade, there has been a notable increase in the incidence of lung cancer among women, reaching unprecedented levels. This trend underscores the urgency of understanding the disease’s characteristics and progression. Our primary objective is to conduct a comprehensive investigation into the behavior of early-stage lung cancer, with a particular focus on identifying and analyzing sex-based differences.

Methodology: We retrospectively analysed data on patients who underwent thoracic surgery for lung cancer stage I from January 2020 to December 2021 at one institution (San Camillo Forlanini Hospital, Rome, Italy). Based on gender, two groups were compared in terms of type of surgery, type of tumor and its specific characteristics such as tumor dimensions (T), tumor differentiation (G), pleural invasion (PL), lymphovascular invasion (LVI), need of chemotherapy/radiotherapy/immunotherapy, disease free survival (DFS) and overall survival (OS). Χ^2^ test was used for categorical variables, U di Mann-Whitney test for metric variables, while DFS and OS were analyzed by Kaplan-Meyer curve. 

Results: 143 consecutive patients were analyzed (66 F; 76 M). Median age was 67 ± 1 years. Type of surgery included 103 lobectomies, 15 segmentectomies, 23 wedge resections and 1 lobectomy + segment. Among these, 60 were performed in open surgery and 82 in VATS, with tri-portal being the preferred surgical access. Surgical type and approach did not statistically influence the two groups. Adenocarcinoma was the most frequent tumor (76.9%), gender independent for tumor dimensions (T), tumor differentiation (G), pleural invasion (PL), lymphovascular invasion (LVI). In our cohort men resulted to die more than women (*p* = 0.017), especially in the range >65 years old (*p* = 0.038). Risk of recurrence was not statistically significant for sex and age, also in terms of type of surgery (VATS *p* = 0.483 vs. OPEN *p* = 0.725). Women seemed to have increased DFS compared to men, yet not statistically significant (*p* = 0.933) 

Conclusions: While the type, approach of surgery and tumor characteristics did not significantly impact outcomes, gender differences were notable in mortality rates and patterns of recurrence. Men, especially those over 65, had higher mortality rate. These findings suggest the need for gender-specific considerations in post-surgical treatment and monitoring strategies for lung cancer patients.

### 1.12. Abstract 12 (Oral Presentation): Lacunar Channels Identified in the Visceral Pleura by Electron Microscopy: A Potential Source of Air Leak?

**Chiara Grispi ^1^, Federica Rossi ^2^, Simona Sobrero ^1^, Alessandra Russo ^1^, Marco Marcaccini ^1^, Luisella Righi ^3^, Catalina Ciocan ^2^, Alessandro Godono ^2^ and Francesco Leo ^1^**

1. Thoracic Surgery Division, Department of Oncology, San Luigi Hospital, University of Turin, Orbassano, Italy
2. Occupational Medicine Division, Laboratory of Toxicology and Industrial Epidemiology, Department of Public Health and Paediatrics, University of Turin, Turin, Italy
3. Patology Unit, Department of Oncology, San Luigi Hospital, University of Turin, Orbassano, Italy

Background: Sometimes pneumothorax may occur in absence of bullae. This suggests the alternative hypothesis that visceral pleura may present lacunar areas through which air leak occasionally occurs. In order to verify this hypothesis, visceral pleura was investigated by scanning electron microscope (SEM) both in pneumothorax cases and in controls.

Methodology: Visceral pleura was sampled from 10 patients operated for recurrent pneumothorax and 16 patients who underwent lobectomy for lung cancer (controls). Fresh material was stored at −80 °C and then cut by a microtome at a thickness of 60 microns parallel to the pleura surface in the first 20 cases and perpendicularly in the other 6 cases, in order to obtain bidimensional information. After coating the samples with a film of gold, samples were analysed by SEM (Coxem-Em 30 AX Plus, Media System Lab S.r.l., Monza e Brianza, Italy) at 15 Kev and 2 k magnification.

Results: In all 26 cases, SEM identified round, well-defined lacunar structures with a homogeneous thickness ranging from 1 to 4 micron. A great heterogeneity was detected in terms of density and diameter: in pneumothorax cases, channels had an average diameter of 10.4 micron and a density of 57 structure per field. Among controls, channels were smaller (average diameter 1.6 micron) and less dense (21.6 per field). The *p* values were statistically significant both for the difference in density (*p* value < 0.01) and for the difference in pore size (*p* value < 0.0001).

Conclusions: Visceral pleura presents lacunar channels whose dimension make them visible by electron microscopy only. These channels are larger and denser in pneumothorax patients, suggesting their potential role as a source of air leak.

### 1.13. Abstract 13 (Oral Presentation): Onset of Pleural Effusion Preceding Diagnosis of Malignant Pleural Mesothelioma: An Observational Retrospective Study in a Cohort of Previously Asbestos-Exposed Patients

**Giacomo Guglielmi ^1^, Gianluca Nerli ^3^, Francesco Porciatti ^3^, Marco Gherardi ^2^, Luciano Gabbrielli ^2^, Giovanni Guglielmi ^4^, Laura Carrozzi ^1^ and Guido Marchi ^2^**

1. Department of Surgical, Medical, and Molecular Pathology and Critical Care Medicine, University of Pisa, Pisa, Italy
2. Pulmonary Unit, Cardiothoracic and Vascular Department, Pisa University Hospital, Pisa, Italy
3. Department of Translational Research and New technologies in Medicine and Surgery, University of Pisa, Pisa, Italy
4. Preventive and Occupational Medicine Unit, Pisa University Hospital, Pisa, Italy

Background: Malignant pleural mesothelioma (MPM) is marked by its challenging diagnosis and poor prognosis. Pleural effusion (PE) can precede the diagnosis of MPM in individuals with a history of asbestos exposure, often manifesting as the initial symptom. This study aimed to assess the incidence of PE preceding MPM diagnosis, along with evaluating relevant variables such as diagnostic modalities, patient characteristics, MPM histology, and the temporal relationship between PE onset and MPM diagnosis.

Methodology: A retrospective analysis was conducted on a cohort of 235 previously asbestos-exposed patients followed by the Pulmonology Unit and Occupational Preventive Medicine Unit at Pisa University Hospital from April 2015 to February 2024. Patients who developed PE were identified, and among them, those who subsequently received a diagnosis of MPM were thoroughly assessed. Data concerning diagnostic methodologies, patient demographics, histological subtypes of MPM, and the temporal interval from PE onset to MPM diagnosis were collected.

Results: Among asbestos-exposed patients, 8.9% developed PE during the study period. All affected individuals were male, with an average age of 79 years. Of these, 38% received a diagnosis of MPM, while 62% had effusions unrelated to MPM (e.g., congestive heart failure). The median time from PE onset to MPM diagnosis in our cohort was 10.8 months (range, 1–51 months). MPM diagnosis was predominantly established through video-assisted thoracic surgery (62.5%), with a minority of cases identified via pleural fluid cytology obtained through thoracentesis (12.5%). Ultrasound or CT-guided pleural biopsy was not used for MPM diagnosis in any patients. In 25% of cases, diagnosis relied on clinical suspicion due to patient frailty precluding invasive biopsy. The most prevalent histological subtype of MPM was epithelioid (50%), followed by sarcomatoid (25%).

Conclusions: Previously asbestos-exposed patients can develop PE, which may precede MPM diagnosis. Vigilant monitoring is crucial due to PE’s potential as the initial sign of MPM. Our study underscores this, observing a notable incidence of PE preceding MPM diagnosis. Thoracoscopic biopsy remains the most appropriate procedure for definitive diagnosis, as determining the histological subtype is crucial for guiding management and predicting prognosis. In a growing population of elderly frail patients, however, biopsy as an invasive procedure may not always be performed. Our findings stress the importance of early detection strategies and tailored diagnostic approaches in asbestos-exposed individuals who develop PE. Timely MPM identification is crucial for initiating proper management. Further studies are needed to evaluate the impact of targeted diagnostic strategies and early management on patient prognosis.

### 1.14. Abstract 14 (Oral Presentation): Use of Biological Suine-Derived Mesh in Chest Wall Reconstruction

**Karan Kumar ^1^, Federico Tacconi ^1^, Cristiano Casciani ^1^, Sebastiano Angelo Bastone ^1,2^, Alexander Patirelis ^1^, Luciano Sposato ^1^, Chiara Combattelli ^1^, Raul Randazzo ^1^, Gian Luca Natali ^1^ and Vincenzo Ambrogi ^1^**

1. Departement of Surgical Sciences, University of Rome Tor Vergata, Via Montpellier 1, 00133, Rome, Itay
2. Ph.D. Program in Applied Medical-Surgical Sciences, Department of Surgical Science, Policlinico Tor Vergata University, Rome, Italy

Background: Several materials have been used so far for the reconstruction of chest wall. Biological suine-derived meshes abdominal parietal reconstruction proved easy and safe. We have recently extended the use of this material for chest wall reconstruction. Indications and results were hereby analyzed.

Methodology: From January 2021 to December 2023, we reconstructed anterior and lateral chest wall defects using a biological suine-derived mesh in 5 consecutive patients. We recorded demographic characteristics, sex, comorbidities, body mass index, chest wall pathology, resection margins according to the Enneking classification, number of involved ribs, defect area, duration of hospitalization, short- and medium-term complications according to Clavien-Dindo classification, the presence of clinically and/or radiologically observed paradoxical respiratory movements (e.g., lung hernia) and overall survival at 30 days. 

Results: A total of 5 patients,1 female and 4 males, with a median age of 53 (IQR: 50–55) years and mass body index of 24 (18–26) were admitted for either primary (*n* = 3) or secondary chest wall tumor (*n* = 2). Number of resected ribs ranged from 3 to 5 with a median defect area of 60 cm^2^ (range 10.7 to 120 cm^2^). Duration of hospital stay was 7 (IQR: 7–9) days. No patients developed paradoxical respiratory movements (e.g., lung hernia). Neither 30-days mortality nor major medical morbidity was reported. One patient who had a complex anterior chest wall reconstruction with mesh and rectus abdominis transposition necessitated surgical revision due to a hematoma, which was successfully managed with subsequent discharge without further complications. At discharge, pain scores were minimal with negligible analgesic requirements. 

Conclusions: Our preliminary experience suggests that the use of bioprosthetic meshes for chest wall reconstruction is both safe and feasible, offering promising outcomes in terms of anatomical restoration, infection control, and pain management of the chest wall. It stands as a viable alternative to synthetic materials and rib plates.

### 1.15. Abstract 15 (Oral Presentation): Suitability of Transbronchial Biopsies Using Ultrathin Bronchoscope (Utb) and 1.2 Mm Forceps for Genotyping of Peripheral Pulmonary Lesions (Ppl)

**Giuseppe La Mattina, Michele Gallo and Giuseppe Arcoleo**

A.O.O.R. Villa Sofia—Cervello—Palermo (Italy)

Background: UTB has shown promise in enhancing diagnostic yield by improving visualization of small caliber airways. However, there is a lack of studies investigating the utility of UTB with TBB using 1.2 mm biopsy forceps for genotyping peripheral lung cancer lesions.

Methodology: This prospective cohort study focuses on patients referred for biopsy of PPL. UTB (3 mm outer diameter at tip and 1.7 mm working channel) was routinely employed. Radial EBUS and 2D fluoroscopy were utilized when lesions weren’t visualized endoscopically. Biopsy samples were obtained using 1.2 mm forceps. Molecular analyses, including next-generation sequencing (NGS) and immunohistochemistry (IC) for PD-L1 expression, were performed on biopsy material post-diagnosis. 

Results: From 3 July 2023 to 25 April 2024, 40 patients with PPL were studied. The median longest diameter in the axial plane was 28.7 mm, ranging from 10 mm to 55 mm. The diagnostic yield was at 95%, and one pneumothorax was reported. The diagnosis were 20 lung adenocarcinoma, 5 NOS, 5 squamous cell carcinoma, 3 small-cell carcinoma, 2 metastasis, 3 granulomatous inflammation and 2 non diagnostic (1 lymphoma and 1 lung adenocarcinoma). 

Conclusions: The use of UTB with 1.2 mm biopsy forceps in transbronchial biopsies proves to be a reliable tool for diagnosing PPL. The obtained diagnostic material consistently proved adequate for both PD-L1 expression and NGS analyses. These findings suggest the potential clinical utility of 1.2 mm forceps in improving the accuracy of genotyping peripheral lung lesions, contributing valuable insights for future diagnostic approaches. 

### 1.16. Abstract 16 (Oral Presentation): Is Increased Surgical Risk Due to Smoking Only Partially Reversible? Results from the French Society for Thoracic and Cardiovascular Surgery (Epithor) Database

**Francesco Leo, MD ^1,4^, Giuseppe Migliaretti ^2^, Simona Sobrero ^1^, Dan Angelescu ^3^, Tarun Mc Bride ^3^, Marcel Dahan ^5^ and Jacques Jougon ^6^ on behalf of the Société Française de Chirurgie Thoracique et Cardiovasculaire (SFCTCV)**

1. Thoracic Surgery Division, Oncology Department, S. Luigi Hospital, University of Torino, Italy
2. Department of Public Health and Paediatric Sciences, University of Torino, Italy
3. Thoracic Surgery Division, Périgueux General Hospital, France
4. Smoking Cessation Unit, Périgueux General Hospital, France
5. Thoracic Surgery Division, Toulouse University Hospital, France
6. Thoracic Surgery Division, Bordeaux University Hospital, France

Background: In smokers, the probability of developing postoperative complications after lung resection is increased but this additional risk is difficult to assess as data from large cohorts are limited. The aim of this study was to measure the impact of smoking on postoperative morbidity and differences between active and former smokers analysing data from Epithor, the database of the French Society of Thoracic and Cardiovascular Surgery (SFCTCV).

Methodology: Data of patients who underwent lung resection for primary lung cancer in the period 2002–2020 were extracted from Epithor and cases without information on smoking history were excluded. Mortality and morbidity were analysed according to smoking status and a further analysis compared former smokers and active smokers. The risk of overall and specific postoperative complications according to smoking status was defined by logistic regression models and results were presented in terms of OR and relative 95% confidence intervals adjusted for confounding factors identified in the descriptive analysis.

Results: The study cohort was composed of 7204 patients, 18.9% of which were never smokers. As compared to never smokers, in patients having an history of smoking, an increased mortality and morbidity were recorded in smokers (respectively 1.1% vs. 0.4%, *p* 0.01, and 30.4% vs. 20.2%, *p* < 0.01) due to a significantly higher incidence of respiratory events, prolonged air leak, infections and neurological problems. As compared to never smokers, former smokers (*n* = 4393) presented an intermediate risk of respiratory complications (OR 1.95, CI 1.2–3) and infections (OR 3.2, CI 1.6–6.3), which was even higher in active smokers (OR 3.2 CI 1.9–5.2 and OR 4.66, CI 2.3–9.4 respectively). Median hospital stay was shorter in non smokers (6 days) as compared to ex smokers (7 days) and current smokers (8 days).

Conclusions: Smokers are at increased risk for morbidity and mortality after lung resection. In former smokers, this risk is reduced but does not reach the low risk profile of never smokers, suggesting that damage due to cigarettes is not fully reversible.

### 1.17. Abstract 17 (Oral Presentation): Efficacy, Safety and Cost-Effectiveness of Intrapleural Urokinase in Complicated Parapneumonic Pleural Effusion: An Italian Experience

**Maria Angela Vittoria Anna Chiara Licata ^1^, Gina Gualano ^1^, Alberto Zolezzi ^1^, Annelisa Mastrobattista ^1^, Serena Maria Carli ^1^, Paola Mencarini ^1^, Silvia Mosti ^1^, Chiara Pierandrei ^2^, Maria Serafini ^2^, Gian Paolo Mattioli ^2^, Francesca Marchesani ^2^, Nicolo’ Maria Vanoni ^3^, Cipolla Giuseppe ^3^, Ferroni Valentina ^3^, Michele Morriello ^4^, Sara Colella ^4^, Stefano Marinari ^4^, Daniel Piamonti ^5^, Paolo Palange ^5^, Morgana Vermi ^6^, Alessandro Andreani ^6^, Pierfrancesco Longo ^7^, Roberto Cascone ^8^, Alessandra Pesci ⁹ and Fabrizio Palmieri ^1^**

1. UOC Malattie infettive dell’apparato respiratorio, IRCCS INMI Spallanzani-Rome (Italy)
2. UOC Pneumologia AST Macerata, Ospedale Generale di Macerata (Italy)
3. UOC Pneumologia, ASST Lodi (Italy)
4. UOC Pneumologia Teramo (Italy)
5. UOC Pneumologia, dipartimento di Sanità pubblica e Malattie Infettive, Sapienza Università di Roma (Italy)
6. UOC Pneumologia Mirandola ASL Modena (Italy)
7. UOC Pneumologia, Ospedale Cardinale G. Panico Tricase (Italy)
8. UOC Chirurgia Toracica, IRCCS Centro di Riferimento Oncologico della Basilicata, Rionero in Vulture (Italy)
9. UOC Medicina Interna ad alta intensità, Ospedale Santa Maria Nuova, Firenze (Italy)

Background: The MIST2 trial showed that intrapleural alteplase (tPA) and dornase alfa (DNase) improved fluid drainage in pleural infection and reduced surgical referrals Since the publication of MIST2, tPA + DNase has become the most commonly used intrapleural fibrinolytic in Europe. Few studies compare urokinase and tPA + DNase, showing similar efficacy, but tPA + DNase has been associated with a higher incidence of complications. The aim of this study was to evaluate, in an Italian setting, the efficacy, the safety and the cost-effectiveness of intrapleural instillation of urokinase in patients affected by complicated parapneumonic pleural effusion.

Methodology: From March 2023 to May 2024, we retrospectively enrolled 40 patients with complicated parapneumonic pleural effusion from different Italian centres treated with chest tube placement and intrapleural instillation of urokinase. We evaluated tube size, pleural fluid cultures, the duration of fibrinolysis (with the injection via chest tube of 1 vial of 100,000 IU per day), the duration of drainage, complications of fibrinolysis, length of hospital stay, the fibrinolytic cost (around 60 euros per vial), death at 30 days and the surgical referral. 

Results: The mean age was 53.55 ± 20.39 years, with a male predominance (77.5%). 24 patients had comorbidities: included cardiovascular diseases (16), diabetes (4), and immunodeficiencies (7). The pleural effusion predominantly affected the right side (62.5%). Chest tube size ranged from 12 to 28 French (median 15 French), only 9 patients had a tube size greater than 15 French. There were 17 positive pleural fluid cultures, including 5 for tuberculosis. Fibrinolysis lasted 3 ± 1.32 days while duration of chest drainage was 9.11 ± 4.45 days. Only one patient had complications (pain and dyspnoea) during urokinase injection, resolved by opening the tube. The length of hospital stay was 21 ± 9.4 days. The mean fibrinolytic cost was 180 ± 63.73 euros per procedure. No patients died within 30 days. Despite the reduced size of the chest tubes positioned, only 2 patients (5%) required surgery. 

Conclusions: Although in Europe the use of tPA + DNase has been preferred as an intrapleural treatment for parapneumonic pleural effusions since the publication of MIST2, our Italian experience shows that urokinase is a valid, safe, effective and low-cost alternative.

### 1.18. Abstract 18 (Oral Presentation): Bronchoscopic and Surgical Treatment of Tracheobronchomalacia: A French Referral Centre Experience

**Sara Lo Torto ^1^, Francesco Ferrante ^2^, Ilaria Onorati ^3^, Ana Maria Portela ^3^, Marine Peretti ^3^, Dana Radu ^3^, Olivia Freynet ^4^, Yurdagül Uzunhan ^4^, Gilles Mangiapan ^5^, Olivier Huet ^6^, Hervé Dutau ^7^ and Emmanuel Martinod ^3^**

1. Department of Thoracic Surgery, Tor Vergata University Polyclinic, Rome, Italy
2. Department of Thoracic Surgery, University of Rome Sapienza, Rome, Italy
3. AP-HP, Hôpitaux Universitaires Paris Seine-Saint-Denis, Hôpital Avicenne, Chirurgie Thoracique et Vasculaire, Université Sorbonne Paris Nord, Faculté de Médecine SMBH, Bobigny, France
4. AP-HP, Hôpitaux Universitaires Paris Seine-Saint-Denis, Hôpital Avicenne, Pneumologie, Université Sorbonne Paris Nord, Faculté de Médecine SMBH, Bobigny, France
5. Service de Pneumologie CHI de Créteil, Créteil, France
6. Department of Anaesthesiology and Intensive Care Medicine, Brest University Hospital, Brest, France
7. Department of Thoracic Oncology, Pleural Diseases and Interventional Pulmonology, APHM, Marseille, France

Background: Tracheobronchomalacia (TBM) is a rare disease, with various clinical and anatomic forms and aetiologies. The aim of this pilot study was to identify a combined algorithm of treatment for adult patients affected by tracheobronchomalacia for a future randomized study (Beluga Trial).

Methodology: Since 2009, at our referral centre for tracheal disease, we proposed a bronchoscopic treatment by stent placement for patients affected from severe TBM after medical optimization of comorbidities. In 2018 we started a pilot study based on Boston’s experience (Beth Israel Deaconess Medical Centre), consisting in surgical stabilization of the central malacic airways by posterior splinting with a prosthetic mesh (tracheobronchoplasty, TBP) after a first stent-trial step. All cases were discussed and validated by a multi-disciplinary team.

Results: Twenty-five patients, with a mean age of 61 ± 1 years (range, 25–88 years) were included. Fifteen patients (2009 to 2016) had an exclusive bronchoscopic treatment with airway stent insertion and 10 patients, since 2018, had the endoscopic treatment followed by TBP. There was no 90-day mortality in both groups, whereas in endoscopic group 7 patients (47%) died at long term follow up and only 3 of them keep a stent. Decrease of airway collapse at 12 months after TBP was found out in 80% of the patients associated with good peri-operative follow up, with the exception of 2 patients who reported respectively basal atelectasis and asthma exacerbation requiring medical treatment changing. Long term follow-up in TBP group was excellent in 7 patients and favourable in 3, without in-hospital readmission within 12 months after surgery and improvement of pulmonary function. In endoscopic group the procedure was effective in 13 patients (87%) and dyspnoea and cough were improved in 6 patients. Lung obstruction was relieved in 3 patients. The procedure was not successful in 2 patients (13%) as malacia was associated with a subglottic stenosis. With a mean follow-up of 18.3 months, stents were removed in 7 patients because of granuloma, non-efficacy or cough. 

Conclusions: Airway stenting in severe adult TBM could improve symptoms at first and allow efficient patient’s selection by stent trial to surgical airway stabilisation. TBP was associated with good results in terms of symptomatology improvement and health-related quality of life in carefully selected patients. Our pilot study confirms the feasibility of protocol proposed by Beth Israel Deaconess Medical Centre and its results. A multicentric randomized study will be performed in our centre.

### 1.19. Abstract 19 (Oral Presentation): Downregulation of Extracellular Vesicle Mirna-141 in Patients with Non-Small Cell Lung Cancer Harboring Egfr Mutations

**Domenico Loizzi ^1^, Dian J. Salih ^2^, Nicoletta Pia Ardò ^1^, Maria Teresa Bevilacqua ^1–3^, Diletta Mongiello ^1–3^, Francesco Sollitto ^1^ and Teresa Santantonio ^2^**

1. Università di Foggia, Azienda ospedaliero-universitaria “Policlinico Riuniti” Foggia, Struttura Complessa di Chirurgia Toracica Universitaria, Foggia-Italy
2. Infectious Diseases Unit, Department of Clinical and Surgical Sciences, University of Foggia, Foggia-Italy
3. Università degli Studi di Bari, Azienda ospedaliero-universitaria “Policlinico Consorziale” Bari-Italy

Background: MicroRNAs (miRNAs) play crucial roles in cancer biology, including the regulation of key oncogenic pathways. This study investigates the role of miRNA-141 in regulating epidermal growth factor receptor (EGFR) expression in non-small cell lung cancer (NSCLC). 

Methodology: We isolated extracellular vesicles (EVs) from the serum of 15 NSCLC patients, including 4 with EGFR mutations (exon 19 deletion and exon 21-point mutation). EVs were characterized using nanoparticle tracking analysis (NTA) and western blotting for the markers CD9, CD81, and TSG101, confirming successful isolation. We then evaluated the levels of miRNA-141 in the EVs and serum of these patients.

Results: Our results showed that miRNA-141 was significantly downregulated in both the serum and EVs of patients with EGFR mutations compared to those with wild-type EGFR and healthy controls. This downregulation suggests that miRNA-141 may play a role in modulating EGFR expression in NSCLC, particularly in cases with specific EGFR mutations. The findings imply that miRNA-141 could serve as a potential biomarker for EGFR mutation status in NSCLC and might contribute to the development of targeted therapies.

Conclusions: These results underscore the importance of miRNA-141 in the context of EGFR-mutant NSCLC and highlight its potential as a therapeutic target. Further research is needed to elucidate the precise mechanisms by which miRNA-141 regulates EGFR expression and to explore its utility in clinical applications.

### 1.20. Abstract 20 (Oral Presentation): Stereotactic Body Radiotherapy (Sbrt) Guided by Fiducials in Patients with Primary and Secondary Ultracentral Malignant Thoracic Lesions: Preliminary Data of Fluxux Trial

**Valentina Luzzi ^1^, Mauro Loi ^2^, Marco Trigiani ^1^, Lorenzo Corbetta ^1^, Luca Ciani ^1^, Leonardo Giuntoli ^1^ and Sara Tomassetti ^1^**

1. Pneumologia Interventistica, AOU Careggi, Firenze
2. Radioterapia, AOU Careggi, Firenze

Background: The use of ablative Stereotactic Body Radiotherapy (SBRT) for the treatment of mediastinal and hilar lymph nodal metastases (MHL) has been traditionally limited due to proximity of critical organs and uncertainty in target recognition and treatment administration due to respiratory motion, potentially resulting in fatal adverse events. Fiducial-markers implanted via Endobronchial Ultrasound (EBUS) may improve treatment delivery by allowing Real-time tumor tracking (RTTT) or by refining Cone Beam Computed tomography (CBCT) image guidance. 

Methodology: Data from a prospective cohort of patients treated from December 2021 to December 2023 with ablative SBRT for oligometastatic or oligoprogressive MHL from miscellaneous primary tumors were collected. Gold-anchor 25 G FM were placed intralesionally via rigid bronchoscopy under general anesthesia following EBUS. One week later, blank and contrast enhanced simulation CT was acquired: in order to verify the solidarity between the FM and the tumor across the respiratory cycle an additional 4D CT was obtained. SBRT was delivered using RTTT with a robotic-arm linac or surface and CBCT guided deep-inspiration breath hold (DIBH). Preliminary outcome and toxicity results were assessed.

Results: Eighteen patients, accounting for 21 MHL were included. Median age was 73 (range 48–87) years. Median follow-up was 6 months. Primary tumor location were lung (*n* = 8), breast (*n* = 4), gastroesophageal (*n* = 3), and other (*n* = 3). Disease setting was oligometastatic and oligoprogressive MHL in 7 and 11 patients, respectively. MHL were located in nodal station 10–11 (*n* = 7), 4 (*n* = 6), 7 (*n* = 5), 2–3 (*n* = 2) and 8 (*n* = 1). Median number of implanted FM was 3 (range 2–3): no periprocedural complication or migration was observed. RTT and DIBH were used in 15 and 3 patients, respectively. In 3 patients, 2 MHL were simultaneously treated during the same treatment course, the remaining patients receiving SBRT to a single nodal site. Median prescription dose was 35 Gy (range, 30–40 Gy) in 5 fractions, corresponding to a median EQD2 of 49.5 Gy (range, 48–100 Gy) assuming α/β = 10. Local control (LC) was 95% at 1 year. Distant relapse-free (DRFS) and overall survival (OS) were 54% and 73% at 1 year, respectively. All 6 patients who were chemotherapy-naïve before SBRT were free from systemic therapy at the time of the analysis. Acute transient toxicity, consisting of grade 1 cough and pyrosis was observed in 2 patients. Only one patient experienced a grade ≥ 3 late toxicity, consisting of aorto-oesophageal fistula resulting in fatal hematemesis.

Conclusions: FM placement is a minimally invasive and well tolerated procedure in elderly patients and allow the delivery of ablative SBRT to MHL with promising outcome and toxicity results. Caution is required in case of suspicion of mediastinal organ infiltration.

### 1.21. Abstract 21 (Oral Presentation): Improvement in the Management of Nontuberculous Mycobacteria after Lobar Collapse Therapy with Endobronchial Valves

**Valentina Luzzi ^1^, Jessica Mencarini ^2^ and Lorenzo Corbetta ^1^**

1. Pneumologia Interventistica, AOU Careggi, Firenze
2. Malattie infettive e Tropicali, AOU Careggi, Firenze

Background: The incidence of non tuberculous mycobacteria (NTM) has been increasing worldwide and the treatment success rate is unsatisfactory with a successfully eradicate in only 60–80% of patients with a significant proportion of successfully treated patients experience disease recurrence. In a previous study Corbetta et al. [2] the Zephyr^®^ EBV (Pulmonx Corporation, Redwood City, CA, USA) have been using in the successful management of inoperable cases of MDR and XDR-TB with cavities.

We describe our first case series of NTM with cavities not responder to therapy or/and relapsed treated with EBV as treatment adjunct to pharmacotherapy in order to increase our experience whilst awaiting the beginning of our randomized protocol that is under evaluation by the Ethics Committee.

Methodology: We recruited patients with NTM with cavities deemed unsuitable for surgery at the Careggi hospital in Florence and valves were implanted in rigid bronchoscopy in general anesthesia. The number of implanted valves depended on the endobronchial anatomy of each patient. 

Results: We treated 3 patients (2F/1M) affected by M. Xenopi (2/3) and M. Avium complex (1/3) with positive sputum at the time of the procedure. Target of EBV placement were the cavities that were located Right upper lobe (2/3) and right lower lobe (1/3). A median of 3 valves were inserted to isolate the cavities. Loss of volume of the treated lobe were seen in all patients. No complications were observed after procedure. Sputum at 3 months after procedure were negative.

Conclusions: According to the results of international studies such the study of Huiru An et al. [3], the study of Corbetta et al. [2] and Levin et al. [4], our early experience showed benefit in terms of conversion of sputum smear and culture to negative and radiological closure or reduce in size of the cavities (Figure 3).

This confirm the need to develop a prospective and randomized study and our team is working on it. The protocol is currently under consideration by the Ethics Committee of AOU Careggi.

### 1.22. Abstract 22 (Oral Presentation): Treatment of Tracheobronchial Amyloidosis with Rigid Laser-Assisted Bronchoscopy: Results of a 24–36 Month Follow-Up

**Umberto Masi ^1^, Vito D’Agnano ^1^, Cristiano Cesaro ^2^, Roberta Cianci ^1^, Ilaria Menichini ^3^, Andrea Bianco ^1^ and Giovanni Galluccio ^3^**

1. Department of Translational Medicine, University of Campania “L. Vanvitelli”, Naples, Italy
2. Bronchology Unit, Monaldi Hospital, Naples, Italy
3. Center of Thoracic Endoscopy and Interventional Pulmonology, Regina Apostolorum Hospital, Albano Laziale, Italy

Background: The aim of this study is to evaluate the efficacy and safety of rigid laser-assisted bronchoscopy in the treatment of tracheobronchial amyloidosis. This rare condition is characterized by the deposition of amyloid proteins in the tracheobronchial tree, resulting in airway obstruction and compromised respiratory function. Long-term outcomes of tracheobronchial stent placement in select cases were also evaluated.

Methodology: A retrospective analysis was conducted on a cohort of patients with tracheobronchial amyloidosis treated at our institution between 2018 and 2021. The diagnosis was confirmed through bronchial biopsy and Congo Red staining. All patients underwent rigid bronchoscopy under general anesthesia, using Nd-YAG laser to vaporize and remove amyloid deposits and biopsy forceps for larger fragments. Patients were followed up for 24 to 36 months to monitor respiratory function and quality of life. Reduction in airway obstruction was confirmed by bronchoscopy and chest computed tomography. The effectiveness was measured by improvement in respiratory function tests and symptom resolution. Data were collected and analyzed using descriptive statistics.

Results: We enrolled 10 patients. Demographic and clinical data are reported in table. The average duration of the procedure was 30 min (range: 20–45 min) with no serious adverse events reported. Minor complications, including transient pain and minimal bleeding in three case (30%), were successfully managed. Significant respiratory improvement was observed within one month post-procedure: 90% showed significant improvement of dyspnoea, 70% reported resolution of persistent cough, 40% experienced complete relief of stridor. Two patients (20%) required a second procedure 12 months later due to recurrence of symptoms. Stents were placed in 2 patients (20%) to maintain airway patency after the laser procedure. Pre- and post-procedure spirometry showed a mean improvement in FEV1 of 25% (range: 15–35%). After 24–36 months, 9 of 10 patients (90%) maintained good respiratory function without additional interventions. One patient experienced local recurrences that required minor revision procedures.

Conclusions: Rigid laser-assisted bronchoscopy proved to be an effective and safe technique for the treatment of tracheobronchial amyloidosis, with significant improvements in respiratory function and quality of life. Tracheobronchial stent placement provided valuable support in more severe cases, ensuring airway patency in the long term. The results of the 24–36 month follow-up are promising, but further studies with a larger sample size and extended follow-up are needed to confirm and optimize treatment strategies.

### 1.23. Abstract 23 (Oral Presentation): Burnout among Thoracic Surgery Residents in Italy: Results from the Italian Residents Survey

Giovanni Mattioni ^1^, Riccardo Orlandi ^1^, Andrea Onofri ^2^, Andrea Anastasi ^3^, Doroty Sampietro ^4^, Diletta Mongiello ^4,5^, Graziana Carleo ^5^, Cinzia Scala ^6,7^, Luigi Paladini ^8,9^ and Federico Raveglia ^10^ and on behalf of the SIET Residents’ Board *

1. Department of Thoracic Surgery, University of Milan, Milan, Italy
2. Department of Agricultural, Food and Environmental Sciences, University of Perugia, Perugia, Italy
3. Department of Mental Health, Fondazione IRCCS Ca’ Granda Ospedale Maggiore Policlinico, Milan, Italy
4. Thoracic Surgery Unit, Department of Precision and Regenerative Medicine and Ionian Area, University of Bari Aldo Moro, Bari, Italy
5. Thoracic Surgery Unit, Policlinico Universitario Riuniti di Foggia, Foggia, Italy
6. Division of Thoracic Surgery, IRCCS San Raffaele Scientific Institute, Milan, Italy
7. Università Vita-Salute San Raffaele, Milan, Italy
8. Department of Medical and Surgical Sciences, Institute of Respiratory Disease, University Hospital, Foggia, Italy
9. Respiratory Diseases Unit Antonio Blasi, Presidio Ospedaliero A. Perrino, Brindisi, Italy
10. Department of Thoracic Surgery, IRCCS San Gerardo Hospital, Monza, Italy

*  Correspondence: Noemi Camarda, Alessandra Cotroneo, Delia Giovanniello, Antonio Lamesta, Beatrice Leonardi, Fabiana Messa, Adriana Nocera, Letizia Perri, Giorgia Piccioni, Domenico Pourmolkara, Emanuele Vocale

Background: Burnout is defined as a state of physical and mental exhaustion arising from a protracted exposure to chronic stressors. It is a well-recognized but often underestimated issue affecting residency programs, particularly within surgical ones. It has been associated to cynicism, poor self-efficacy, impaired interpersonal relationships, lower job satisfaction, and increased frequency of medical errors. These factors might negatively affect both patients (reduced care quality) and residents (mental health-related issues and poorer training). Nonetheless, the spread of this condition among Italian thoracic surgery residents has never been previously investigated. This study aims to explore the current prevalence of burnout within this cohort of healthcare practitioners.

Methodology: A cross-sectional study was conducted using a nation-wide anonymous facultative survey, which was generated through SurveyMonkey and administered to Italian thoracic surgery residents (excluding 1st year residents), from December 2023 to March 2024. The survey consisted in 74 questions in Italian language. Burnout risk was assessed in a dedicated section, validated according to the Maslach Burnout Inventory (MBI) Scale based on 22 items, exploring the three dimensions of emotional exhaustion (EE), depersonalization (DP) and personal accomplishment (PA). Cutoff values for each risk category were determined from previous normative MBI data for Italian healthcare workers (low EE ≤ 14, intermediate EE = 15–23, high EE ≥ 24; low DP ≤ 3, intermediate DP = 4–8, high DP ≥ 9; low PA ≥ 37, intermediate PA = 30–36, high PA ≤ 29) [5]. Burnout was defined as the highest score in at least one subscale (i.e., EE, DP, PA). Therefore, the highest score in all the three subscales corresponded to a high level of burnout.

Results: The mean scores were 24 ± 11.3 for emotional exhaustion, 6.9 ± 5.9 for depersonalization, and 36.6 ± 6.8 for personal accomplishment. Burnout features were identified in 64 (65.3%) residents, with no significant gender prevalence (males-to-female ratio = 0.94). The highest subscale score categories were found in 51 (52%) for EE, 15 (15.3%) for DP, and 34 (34.7%) for PA. Burnout was present in one subscale in 34 (34.7%) cases, in two subscales in 24 (24.5%), and a high level of burnout (i.e., in all the three subscales) was found in six (6.1%) residents, equally distributed between males and females (50%).

Conclusions: A significant prevalence of burnout was observed among Italian thoracic surgery residents. The thoracic surgery residency program presents intrinsic complexities that contribute to an increased risk of developing burnout. Despite this, efforts must be made to identify potential causal factors and propose tailored interventions.

### 1.24. Abstract 24 (Oral Presentation): Appropriateness of Lymphadenectomy in Resectable Non-Small Cell Lung Cancer: In Search for the Correct Approach

**Diletta Mongiello ^1,2^, Maria Teresa Bevilacqua ^1,2^, Francesca Cialdella ^1^, Nicoletta Pia Ardò ^1^, Roberto De Bellis ^1^, Francesco Lastaria ^1^, Sara Tango ^1^, Giulia Pacella ^1^, Rita Daniela Marasco ^1^, Leonardo Fino ^1^, Francesco Sollitto ^1^ and Domenico Loizzi ^1^**

1. Università di Foggia, Azienda ospedaliero-universitaria “Policlinico Riuniti” Foggia, Struttura Complessa di Chirurgia Toracica Universitaria, Foggia-Italy
2. Università degli Studi di Bari, Azienda ospedaliero-universitaria “Policlinico Consorziale” Bari-Italy

Background: Lymphatic invasion is the most significant prognostic factor impacting survival in NSCLC. Appropriate assessment of lymph node status is essential for correct staging, and appropriate lymph node dissection has also been shown in several studies to have prognostic value. In resectable NSCLC, the definition of appropriate lymphadenectomy is not yet standardized. 

The approach of surgeons to lymphadenectomy is varied and the literature is gradually enriched with new evidence in search for the most correct approach. The questions that usually arise regarding the appropriateness of lymphadenectomy concern how many and which stations it is correct to carry out. 

This study aims to clarify the current evidence of the appropriateness of lymphadenectomy for resectable NSCLC. 

Methodology: We reviewed the literature over the last 10 years in the main search systems with the following keywords: resectable NSCLC, lymphadenectomy, and early-stage lung cancer. The selected papers were focused on several aspects concerning lymphadenectomy: current guidelines on lymphadenectomy for NSCLC, number of lymph nodes harvested, number of stations sampled, the prognostic role of lymphadenectomy, skip metastasis, and micrometastasis.

Results: Lung cancer surgery cannot be considered sufficient without an adequate lymphadenectomy. Various guidelines can be found in the literature describing the results of surgical techniques for sampling or removal of lymph nodes in NSCLC. The effectiveness of one technique over another is still controversial. Nodal upstaging cannot be predicted based on the surgical technique. The number of lymph nodes taken is more important than the number of stations sampled. Tumor size and non-squamous histological type were found to be predictors of nodal upstaging. Skip metastasis is a frequent pattern in NSCLC, and it might be a distinct subgroup for *N* staging. Consequently, the intrapulmonary lymph node assessment is needed, as well as a frozen section. The presence of occult lymph-node micrometastases is considered a marker for primary tumors with high metastatic potential, and molecular biology techniques are more effective for the study of them.

Conclusions: The assessment of a patient about *N* descriptor should take several parameters into account. The outcome of the *N* collection should not be considered alone as the final descriptor of pathology but should probably also be supplemented with variables that are predictors of lymph node invasion. It remains that the more lymph nodes are removed, the more adequate the lymphadenectomy will be.

### 1.25. Abstract 25 (Oral Presentation): Predictors of Compensatory Sweating and Satisfaction Following Endoscopic Thoracic Sympathetic Chain Clipping for Palmar/Axillary Hyperhidrosis

**Dania Nachira ^1^, Adriana Nocera ^1^, Antonio Giulio Napolitano ^1^, Elisa Meacci ^1^, Maria Teresa Congedo ^1^, Marco Chiappetta ^1^, Carolina Sassorossi ^1^, Khrystyna Kuzmych ^1^, Filippo Lococo ^1^, Maria Letizia Vita ^1^, Leonardo Petracca-Ciavarella ^1^ and Stefano Margaritora ^1^**

1. Department of Thoracic Surgery, Fondazione Policlinico Universitario A.Gemelli IRCCS, Università Cattolica del Sacro Cuore, Rome, Italy

Background: Endoscopic thoracic sympathetic (ETS) chain clipping is a definitive treatment for palmar and/or axillary primary hyperhidrosis (PH), however compensatory sweating (CS) remains the most feared complication. Aims of this study are to investigate the factors associated with CS and satisfaction and to evaluate the post-operative quality of life (QoL).

Methodology: From January 2003 to August 2023, 180 patients undergone two-stage ETS were prospectively asked to complete a post-operative questionnaire (2 weeks after each side surgery and on a mean follow-up of 30.2 ± 5.4 months) on satisfaction, CS and QoL in several daily activities.

Results: Seventy-nine patients (45.7%) were male, 52 (30.1%) active smokers, with a mean body max index (BMI) of 22.6 ± 3.14. The most part of population (112 (62.2%)) was operated for combined palmar and axillary PH, whereas 56 (31.1%) patients had only palmar and 12 (6.7%) only axillar PH. Forty-eight (26.7%) patients developed CS after first side ETS (right side was operated first in 85.6% of cases), mainly on contralateral hand, thorax and feet, with a 100% effectiveness on the operated side. Only 122 (67.8%) patients completed ETS on both side (median:3 months between the two surgeries) and follow-up in the study period. CS after second side ETS was 40.2% (49 patients), mainly on thorax, thighs and feet, with an effectiveness of 96.7%. At final follow-up, CS was 50.8% (62 patients), severe CS in 7 cases (5.7%); 9 (7.4%) patients developed a gustatory CS. The final effectiveness of ETS was 95.9%, with a reported improvement in QoL in 95.3% of cases (mainly in manual work and socialization); 94.1% of patients was satisfied and would do ETS again. At multivariable analysis only older age (>24 years) was a predictor of CS (HR: 1.084, 95%CI [1.023–1.149], *p*: 0.007) and severe CS (HR: 1.076, 95%CI (1.002–1.156), *p*: 0.042). No predictor for satisfaction was found.

Conclusions: ETS by clipping can improve QoL in case of palmar/axillar hyperhidrosis. Older patients must be informed of a higher risk of CS.

### 1.26. Abstract 26 (Oral Presentation): Post-Operative Air Leaks Management Using Polymeric Hydrogel Matrix after Thoracoscopic Lung Segmentectomy

**Samuele Nicotra, Alessandro Bonis, Luca Melan, Viola Sambataro, Giulia Pagliarini, Vincenzo Verzeletti, Luigi Lione, Giorgio Cannone, Giovanni Maria Comacchio, Marco Mammana, Alessandro Rebusso, Marco Schiavon, Andrea Dell’Amore and Federico Rea**

Padova University Hospital—Thoracic Surgery Unit, Padova, Italy

Background: A still unsolved issue in thoracoscopic lung resections is represented by intraoperative alveolar air leaks (IOAALs), which if prolonged beyond the fifth postoperative day can lead to higher risk of complications and higher medical costs. The polymeric hydrogel matrix (PHM) is a novel tool to manage intraoperative IOAALs. The primary end-point of our study was to verify whether PHM would be able to reduce postoperative air leaks; secondary end-points were the possible reduction of the permanence time of the chest drain (CD) and the hospital length (HL) in the PHM group compared with no treatment.

Methodology: We enrolled patients with benign and malignant lung diseases suitable for anatomical sublobar thoracoscopic resection at the Thoracic Surgery Unit—University Hospital of Padua, within one year. In the presence of IOAALs, leakage was graded according to a Ventilation Mechanical Test as mild (leak < 100 mL/min), moderate (leak = 100–400 mL/min) and severe (leak > 400 mL/min). We subsequently selected those cases with moderate IOAALs at the end of the resection, and randomized to receive the PHM or not (controls), after the standard closure technique. IOAALs < 30 mL/respiratory act were excluded from the evaluation because self-limiting in most cases and classified as screen failure (SF).

Results: Investigating differences among samples (PHM vs. controls vs. SF), no significance was available in terms of population’s characteristics. 67% of cases were simple or combined upper lobe resections (S1, S2, S3 or lingulectomy). Lower lobe was involved in 33%. 54% of cases were complex segmentectomies. PHM was carried out in 30 cases (33%). Only 2 segmentectomies were performed in RATS, while all others in VATS. We did not register neither conversion nor postoperative major complications. Comparing PHM and controls, CD median time was respectively 1 day and 2 days (*p* = 0.004). The median HL of was 3.5 days while and 2 days in controls (*p* = 0.007).

Conclusions: This study indicates a significant reduction of the chest-tube duration time with a shorter hospital stay in patients treated with PHM. Despite its cost and the small sample investigated, it might be advisable to use sealant in patients eligible for lung segmentectomies with moderate IOAALs and consequently to reduce hospitalization costs.

### 1.27. Abstract 27 (Oral Presentation): Satisfaction in Thoracic Surgery Residency Program in Italy: Insights from a Nation-Wide Survey

Riccardo Orlandi ^1^, Giovanni Mattioni ^1^, Andrea Onofri ^2^, Doroty Sampietro ^3^, Diletta Mongiello ^3,4^, Graziana Carleo ^3^, Cinzia Scala ^5^, Luigi Paladini ^6^, Francesco Petrella ^7^ and Federico Raveglia ^7^ and on behalf of the SIET Residents’ Board *

1. Department of Thoracic Surgery, University of Milan, Milan, Italy.
2. Department of Agricultural, Food and Environmental Sciences, University of Perugia, Perugia, Italy.
3. Unit of Thoracic Surgery, Department of Precision and Regenerative Medicine and Ionian Area, University of Bari “Aldo Moro”, Bari, Italy.
4. Unit of Thoracic Surgery, Policlinico Universitario Riuniti di Foggia, Foggia, Italy
5. Department of Thoracic Surgery, IRCCS San Raffaele Scientific Institute, Università Vita-Salute San Raffaele, Milan, Italy.
6. Department of Medical and Surgical Sciences, “Institute of Respiratory Diseases”, University Hospital, Foggia, Italy.
7. Department of Thoracic Surgery, IRCCS San Gerardo Hospital, Monza, Italy.

*  Correspondence: Noemi Camarda, Alessandra Cotroneo, Delia Giovanniello, Antonio Lamesta, Beatrice Leonardi, Fabiana Messa, Adriana Nocera, Letizia Perri, Giorgia Piccioni, Domenico Pourmolkara, Emanuele Vocale.

Background: In Italy, thoracic surgery residency program has undergone deep changes throughout the last years, to comply with the high-quality European standards and to face off the increasing application of young medical doctors; nonetheless the perspectives of the residents have never been investigated so far. The aim of this study was to assess the satisfaction of thoracic surgery residents in Italy with their training program and to identify any potential influencing factors.

Methodology: We designed a cross-sectional study based on a nation-wide anonymous facultative survey, which was generated through SurveyMonkey and spread among Italian thoracic surgery residents, from December 2023 to March 2024. The survey consisted in 74 questions in Italian language. Specifically, satisfaction was investigated through 10-point Likert scale. Factors influencing satisfaction were analyzed with univariable and multivariable models.

Results: A total of 103 out of 193 residents completed the survey, accomplishing a response rate of 53.4%. Demographic characteristics are reported in Table 3. 

Fifty-three (51.5%) were females, and most participants (46.6%) were from the northern regions of Italy. The mean overall satisfaction for residency program was 6.2 ± 1.9 out of 10. Theorical educational offer (*p* value < 0.001), provision of simulation courses (*p* value 0.002), practice training offer (*p* value < 0.001), monthly number of surgical procedures performed (*p* value 0.048), satisfaction in personal life (*p* value < 0.001), working hours (*p* value 0.001), administrative working hours (*p* value < 0.001), availability of free-time (*p* value 0.044), and quality of colleagues (*p* value < 0.001) were found to be positively associated with higher satisfaction. When combining significant variables into a stepwise multivariable model, higher practical training activity (OR 1.27, C.I. 95%: 1.21–1.49; *p* value < 0.001), higher quality of colleagues (OR 1.35, C.I. 95%: 1.21–1.49; *p* value < 0.001), richer theorical educational offer (OR 1.30, C.I. 95%: 1.18–1.42; *p* value < 0.001), and fewer administrative working hours (OR 0.96, C.I. 95%: 0.89–0.99; *p* value 0.02) were shown to be independent factors for overall satisfaction.

Conclusions: Thoracic surgery residency program aims at the professional development of future thoracic surgeons. However, since education is not a one-way pathway, residents’ perspective should be constantly considered. According to our results, overall satisfaction of residents in thoracic surgery residency program was acceptable, though improvable, and significantly influenced by practice training offer, quality of colleagues, theorical educational offer, and administrative working hours; therefore, we recommend focusing on the highlighted criticalities to enhance residents’ satisfaction and to promote fair education.

### 1.28. Abstract 28 (Oral Presentation): Application of Cone-Beam Ct in Difficult Lung Samplings: A Monocentric Experience

**Roberto Piro ^1^, Matteo Fontana ^1^, Alessandro Capuano ^2^, Eleonora Casalini ^1^, Patrizia Ruggiero ^1^, Chiara Pollorsi ^1^ and Nicola Facciolongo ^1^**

1. Pulmonology Unit, Azienda Unità Sanitaria Locale-IRCCS di Reggio Emilia—Reggio Emilia (RE)
2. Institute of Respiratory Disease, University of Medicine “Aldo Moro”—Bari (BA)

Background: Cone-beam computed tomography (CBCT), applied to bronchoscopy, provides not only an intra-procedural identification of small and non-solid nodules, but also their precise location in relation with forceps or needle, supporting difficult samplings. The objective of this study was to evaluate the feasibility and diagnostic performances of CBCT-guided transbronchial lung samplings.

Methodology: Data from patients who underwent a CBCT-guided bronchoscopy between November 2020 and January 2024 at Azienda USL-IRCCS of Reggio Emilia were analysed. The procedures were conducted under general anesthesia, with a standard or an ultrathin bronchoscope and a radial endobronchial ultrasound probe. Sampling instruments were needles, forceps and cryoprobes. Procedure was scheduled in a hybrid operating room equipped with CBCT machine (Discovery IGS 740, GE Healthcare, Milano, Italy).

Results: The target lesions of 27 patients had a mean diameter of 16 mm. Most nodules were solid (85%) but also ground glass and mixed lesions were sampled. Sixteen (59%) of the patients had multiple lesions. A biopsy was possible and performed in 23 patients, reaching a diagnosis in 16 cases; the diagnostic yield was 70%. Six false negatives samplings (30%) were observed. No adverse event was reported. Data are shown in the table.

Conclusions: CBCT-guided diagnostic bronchoscopy is a feasible and safe technique that could help to reach small and difficult lung nodules. 

### 1.29. Abstract 29 (Oral Presentation): Surgery for Post-Intubation Tracheoesophageal Fistula: Retrospective Case Series Analysis from a Single Center

**Domenico Pourmolkara ^1^, Eleonora Coviello ^1^, Riccardo Amatucci ^1^, Mario Di Stasio ^2^, Lucio Cagini ^2^ and Francesco Puma ^1^**

1. Department of Thoracic Surgery, University of Perugia Medical School, Perugia, Italy
2. Department of Thoracic Surgery, Ospedale del Mare, ASL NA1 Centro, Naples, Italy

Background: Post-intubation tracheoesophageal fistula (PITEF) is a severe condition, often mistreated. This case series reviewed both the choice and timing of surgical technique and the outcome of PITEF patients.

Methodology: We retrospectively analyzed 15 patients, who underwent oesophageal defect repair and airway resection/reconstruction, between 2000 and 2023. We collected the following information: general condition, medical history, preparation to surgery, diagnostic work-up, procedure and timing of surgery, fistula size and site, ventilation type, nutrition, post-operative course and complications.

Results: All patients underwent tracheal resection/reconstruction, according to the Grillo’s technique. Overall, 6/15 patients had undergone a preliminary period of medical preparation. Additionally, 3 patients had already had a tracheostomy, one had had a gastrostomy and 4 had both. Concomitant tracheal stenosis had been found in 9 patients. One patient had a Dumon stent that caused a major enlargement of the fistula. The mean length of the fistulas was 22.6 mm (median 20 mm; range, 8–45 mm), at a median distance from the glottis of 43 mm (range, 20–68 mm). All patients were submitted to tracheal resection; the fistula was included in the resection in 10 patients, while it was excluded in the remaining patients, due to the excessive distance between fistula-tracheostomy- stenosis. Tracheostomy below the tracheal anastomosis was performed in one patient to allow for possible mechanical ventilation. Anastomotic morbidity was recorded in 3 patients and was managed by tracheostomy and T-tube. One patient died from bleeding from oesophageal varices. Definitive closure of PITEF was achieved in all patients. At the 5-year follow-up, the 14 surviving patients had no fistula-related morbidity.

Conclusions: Primary oesophageal closure and tracheal resection/reconstruction appeared to be an effective treatment for PITEF. Systemic conditions, stable weaning from mechanical ventilation, thorough preoperative assessment and adequate preparation were associated with the outcome of these challenging procedures.

### 1.30. Abstract 30 (Oral Presentation): Endoscopic Treatment of Benign Tracheal Stenosis: A Comparative Study

**Emma Repaci ^1^, Flavio Marco Mirabelli ^1^, Ilaria Menichini ^2^, Paolo Palange ^1^ and Giovanni Galluccio ^2^**

1. Department of Public Health and Infectious Diseases, Sapienza University of Rome, Rome, Italy
2. Thoracic Endoscopy, Ospedale Regina Apostolorum, Albano Laziale, Rome, Italy

Background: Tracheal stenosis, defined as a pathological narrowing of the trachea, can occur due to external compression, cervico-mediastinal expansive processes, or intrinsic pathologies of the tracheal wall, resulting from benign or malignant conditions. Among the malignant causes, tracheobronchial tumors are particularly significant. The most common causes of benign tracheal stenosis are orotracheal intubation and tracheostomy, treated with radical endoscopy. Other benign causes include tracheomalacia, chronic inflammatory diseases (amyloidosis, sarcoidosis, relapsing polychondritis), infectious diseases (tuberculosis, rhinoscleroma), and collagen vascular diseases (granulomatosis with polyangiitis, Wegener’s granulomatosis). Benign tracheal stenosis can be classified by etiology (congenital or acquired), morphology (web-like, concentric, granulomatous, “bottle neck” or complex), and localization (subglottic, upper third, lower two-thirds). Diagnosis is performed using respiratory function tests, imaging (X-ray, CT, MRI); endoscopic inspection is the gold standard. Treatment includes laser-assisted mechanical dilation (LAMD), stents, tracheostomy, resection-anastomosis. The management depends on the patient’s clinical condition and stenosis that occludes at least 50% of the tracheal lumen. LAMD uses a rigid bronchoscope and an Nd-YAG laser to make radial incisions and mechanical dilations. In cases not treatable with LAMD or with recurrence, tracheal endoprostheses (silicone or metal) are placed. In complex stenosis, surgical resection of the stenotic segment and anastomosis is preferred.

Methodology: From 1996 to 2017, 425 patients (227 males, 198 females, average age 46 years) were treated for tracheobronchial stenosis at the Thoracic Endoscopy—S. Camillo-Forlanini Hospital in Rome. Diagnosis was made using flexible bronchoscopy and treatment of simple stenosis with rigid bronchoscopy and laser. In cases of recurrence, Dumon prostheses were implanted and followed up (Figure 4). 

Results: The prevalence of simple stenosis (72%) was higher than complex ones (28%). The etiologies included post-orotracheal intubation (66%), post-tracheostomy (26%), and other causes (8%). The predominant location was the upper trachea post-intubation (78%). The cure rate for simple stenosis varied from 52% to 91% with LAMD and prosthesis implantation. For patients with complex stenosis, treatment involved a higher number of interventions, prostheses, and a lower cure rate (65% with stents). Most simple stenosis with granulomas and diaphragm showed high success rates with LAMD.

Conclusions: The treatment of benign tracheal stenosis remains controversial, with the choice between surgery and endoscopic procedures often based on the experience of the centers. Endoscopic techniques, although less invasive, offer good results, especially in patients not eligible for surgery. Our experience indicates a crucial role for pulmonologists in managing this complex pathology.

### 1.31. Abstract 31 (Oral Presentation): Surgical and Pathological Outcomes in Segmentectomies

**Carolina Sassorossi ^1,2^, Marco Chiappetta ^1,2^, Dania Nachira ^1,2^, Filippo Lococo ^1,2^, Elisa Meacci ^1,2^, Maria Teresa Congedo ^1,2^, Antonio Giulio Napolitano ^1,2^, Adriana Nocera ^1,2^ and Stefano Margaritora ^1,2^**

1. Thoracic Surgery, A. Gemelli University Hospital Foundation IRCCS, Rome, Italy
2. Catholic University of Sacred Heart, Rome, Italy

Background: The indications to segmentectomies for NSCLC are increasing, with different possible segments combinations, including different extension of parenchymal, vessels and bronchial resection, which may influence the post-operative and pathological results. Aim of this study is to compare different kind of segmentectomy in terms of complication rate and pathological results.

Methodology: Characteristics of patients who underwent segmentectomy from 1 January 2013 to 31 December 2022 were reviewed and retrospectively analyzed. Patients with clinical nodal involvement and/or distant metastases, receiving neoadjuvant treatment and underwent completion lobectomy were excluded. Operatory and pathological report were reviewed to collect data on surgical characteristics, complication rate and lymphadenectomy. 

Segmentectomies were categorized according to number of resected segments as single/multiple, and according to surgical resection as simple/complex (requiring more than one intersegmental plan resection).

Clinical and pathological characteristics, complications occurrence, kind of complications and nodal parameters were associate to segmentectomy using The Pearson’s χ_2_ or the Fisher’s exact test. The Mann−Whitney U and *t*-tests were used to compare quantitative variables. 

Results: The final analysis was conducted on 127 patients that met the inclusion criteria, 96 (75.5%) were performed in Uniportal-VATS.

Multiple and complex segmentectomies were performed in 63 (49.6%) and 40 (37.3%) cases, respectively.

Complications occurred in 33 (26%) patients, with prolonged air leak (PAL) in 10, haemorrhagic in 4, lung failure in 5, atrial fibrillation in 7, fever and other complications in 7 cases, respectively. No differences were found comparing complex vs. simple or multiple vs. single segmentectomy in terms of all complications and regarding PAL, respiratory failure and ICU need, even if simple segmentectomy presented an higher rate of haemorrhagic complications:4 cases vs0 in complex (*p* = 0.051).

Patients who underwent single segmentectomy presented a significantly higher number of total resected (*p* = 0.047), N2 resected nodes (*p* = 0.012) and a closer margin distance (*p* = 0.012) compared to multiple.

Patients who underwent complex segmentectomy presented a significantly higher number of N2 resected nodes (*p* = 0.020) compared to simple (Table 4).

Conclusions: Kind of segmentectomy is not correlated with complications, but may present significantly differences in terms of margin distance and resected lymph nodes.

### 1.32. Abstract 32 (Oral Presentation): Haller Index and Correction Index in Pectus Excavatum Are Related to Subjective Dyspnea and Cardiorespiratory Function?

**Cinzia Scala ^1^, Piergiorgio Muriana ^2^, Domenico Pontillo ^3^, Stefano Viscardi ^2^ and Paola Ciriaco ^2^**

1. Vita-Salute San Raffaele University, Milan
2. Department of Thoracic Surgery, IRCCS Scientific Institute San Raffaele, Milan
3. Department of Anesthesia and Intensive Care, IRCCS Scientific Institute San Raffaele, Milan

Background: Pectus excavatum is considered a cosmetic problem without a clear correlation with subjective dyspnoea and cardiorespiratory function. This study aims to study the possible relationship between the Haller index and the correction index, with subjective dyspnoea and cardiorespiratory function in patients suffering from pectus excavatum to determine the most appropriate diagnostic path to define the indications for treatment.

Methodology: A retrospective study was carried out on all patients with pectus excavatum evaluated and deemed suitable for surgical or conservative corrective treatment with Vacuum Bell. The Haller index and the correction index were calculated with a magnetic resonance of the chest. The respiratory functional study included in the calculation of FEV1, FVC, and the FEV1/FVC ratio. Pediatric patients unable to perform pulmonary function tests were excluded from this series. Dyspnoea was quantified according to the mMRC-DS scale. The cardiological function was assessed by echocardiography with the calculation of ejection fraction.

Results: Between January 2020 and May 2024, 24 patients (21 men and 3 women) suffering from pectus excavatum worthy of correction were studied at our Thoracic Surgery Unit. The median age was 18.5 with a median Haller index and correction index of 4.35 and 36.5, respectively. A moderate positive correlation of the Haller index with the MRC-DS dyspnoea scale was demonstrated (r = 0.40), with borderline significance (*p* = 0.052). The correction index had a weak positive correlation with the MRC-DS scale (r = 0.23), although, it was not statistically significant (*p* = 0.277). Regarding cardiorespiratory function, the two indices showed a weak negative correlation, not statistically significant, with almost all variables. An exception is the FEV1/FVC ratio, with which the Haller index and the correction index showed a weak positive correlation (r = 0.04) and the other a weak negative correlation (r = −0.06), although both did not reach statistical significance (*p* = 0.848; *p* = 0.795). Subjective dyspnoea was not found to correlate with cardiorespiratory function values.

Conclusions: In our series of patients suffering from pectus excavatum, the only correlation that reaches borderline statistical significance is between the Haller index and the subjective dyspnoea scale. However, subjective dyspnoea does not correlate with cardiorespiratory function. Based on this limited sample of patients, the prevalent aesthetic aspect of this thoracic malformation is confirmed.

### 1.33. Abstract 33 (Oral Presentation): The Role, Timing and Outcome of Surgical Treatment of Thoracic Trauma

**Cinzia Scala ^1^, Piergiorgio Muriana ^2^, Alessandro Bandiera ^2^, Francesca Rossetti ^2^ and Paola Ciriaco ^2^**

1. Università Vita-Salute San Raffaele, Milano
2. Unità Operativa di Chirurgia Toracica, IRCCS Istituto Scientifico San Raffaele, Milano

Background: Thoracic trauma includes a wide spectrum of injuries ranging from simple rib fractures to life-threatening damages. It represents the third cause of death among patients with polytrauma with studies reporting a mortality up to 60% of cases. This emphasizes the need for early evaluation and adequate management to reduce complications and improve outcomes. The work aims to establish the role of thoracic surgery in the treatment of post-traumatic injuries in terms of timing and surgical technique.

Methodology: A retrospective study was carried out on all thoracic traumas referred at the Emergency Department of our Institute and surgically treated from January 2011 to March 2024. Airway, heart, and large vessels traumas were excluded. Patients were divided into two groups. Group I underwent early surgery within 24 h from admission and Group II included patients undergoing late surgery. Differences between the two groups were analyzed.

Results: A total 255 patients were admitted for thoracic trauma; 38 (15%) of them required a surgical treatment (mean age 58 ± 19 years). Ten patients (26%) were included in Group I and 28 (74%) in Group II. Thoracic injuries included rib fractures (53%), pneumothorax (50%), sternal fractures (16%), pulmonary contusion (16%), and chest wall contusion (8%), respectively. Compared with Group II, hemothorax (90% vs. 53.6%, *p* = 0.05) and penetrating injuries (40% vs. 3.6%, *p* = 0.01) were the most frequent cause of early surgery in Group I. Video-assisted thoracic surgery (VATS) was used in 44% of Group I patients and in 39% of Group II patients. Patients in Group I, after surgery, required admission in Intensive Care Unit in 70% of cases reaching a statistically difference compared to Group II (*p* = 0.01). Hospital stay was longer for patients in Group II (mean 18.7 days vs. 8.6 days), although this data was not statistically significant. No statistically significant differences were observed among the groups regarding in-hospital mortality and early readmission (*p* = 0.26 and *p* > 0.99).

Conclusions: Our experience demonstrated that surgery for thoracic trauma is required in a limited group of patients. Early surgical treatment is necessary and can be successfully performed in case of major thoracic trauma including hemothorax and penetrating injuries with good perioperative results. These patients, very often, require postoperative intensive treatment in the ICU setting.

### 1.34. Abstract 34 (Oral Presentation): Robotic Thymectomy and Outcomes Related to Intersurgeons Variations, Single Centre 9-Year Experience

**Elisa Sicolo, Carmelica Cristina Zirafa, Gaetano Romano, Beatrice Manfedini, Ilaria Ceccarelli, Federico Davini and Franca Melfi**

Minimally Invasive and Robotic Thoracic Surgery—Surgical, Medical, Molecular and Critical Care Pathology Department, University Hospital of Pisa

Background: Robotic thymectomy is a growing procedure in the treatment of thymic tumors and the management of neurological symptoms in patients with myasthenia gravis. This technique offers various surgical advantages and good postoperative outcomes; several factors, however, including patient characteristics, nature of the lesion, and neurological syndromes, may influence outcomes. An additional factor of variability may be inter-surgical variables. This study aims to identify factors that influence outcomes of robotic thymectomy, with a focus on intersurgical variables, in a single center.

The main objective is to analyze the influence of variables such as gender, age, presence of myasthenia gravis, histology, thymus volume, surgeon experience, operative time, and postoperative complications on the outcomes of robotic thymectomy.

Methodology: The study included patients undergoing robotic thymectomy from 2014 to 2023. Clinical, surgical, and pathological data were collected retrospectively. Patients were divided according to clinical and surgical characteristics, including those with thymic neoplasia or thymic hyperplasia and myasthenic and nonmyasthenic patients. Surgeons who had performed more than 20 robotic thymectomies were considered experts.

Results: The total sample consisted of 410 patients, 244 (59.9%) women and 166 (40.5%) men, of whom 105 were over 60 years old (25.6%). Within the sample, 329 patients (80.2%) had myasthenia gravis and 294 (71.7%) had thymic neoplasm. Postoperative complications were found in 7.3% of cases. Multivariate analysis showed that operative time was influenced by gender (*p* 0.033), histology (*p* 0.022), thymic volume (*p* < 0.001), and surgeon experience (*p* < 0.001) (Table 5). 

Male gender (*p* < 0.001) and neoplastic histology (*p* 0.005) were associated with higher thymic volume, explaining the longer operative times. A postoperative hospitalization of more than 3 days was associated with the development of postoperative complications (*p* < 0.001) and neoplastic histology (*p* 0.010), but not with surgeon experience. No variables, including surgeon experience, significantly influenced the development of postoperative complications.

Conclusions: Robotic thymectomy can be considered a safe and feasible procedure even for surgeons in training, despite longer operative times due to the learning curve. The results indicate that inter-surgical variables do not significantly influence postoperative complications, suggesting that surgeon experience is not an impacting factor on postoperative outcomes.

### 1.35. Abstract 35 (Oral Presentation): Unexpected Thoracotomy: A Defeat for Minimally Invasive Surgery

**Francesca Signore, Anna Lucia Urgese, Giovanna Imbriglio, Roberta Rapanà, Luigi Andriolo and Camillo Lopez**

U.O.C. Chirurgia Toracica, Ospedale “Vito Fazzi”, Piazza Filippo Muratore 1 Lecce, 73016 LE

Background: The remarkable spreading of video-assisted thoracoscopic surgery (VATS) [6,7], has drawn attention to the possibility of intraoperative conversion to thoracotomy.

We present a retrospective study showing our experience.

Methodology: This monocentric retrospective study was conducted between May 2012 and Mars 2024 and included 851 patients who were undergoing VATS lobectomy. The patients were divided in two groups: VATS lobectomy (Group A) and converted VATS lobectomies (Group B). The median age was 70 years in Group A and 70.5 years and in Group B. Males were 70% in Group A and 80% in Group B, while females were 30% and 20%, respectively. 

Results: The overall incidence of conversion to thoracotomy was 10%. The most common cause of conversion was vascular injury. The other causes were presence of adherences, incomplete scissure and extension of tumor. 

Surgery time was 100 min in Group A and 140 min in group B. The day-stay-drainage was 4 days in Group A and 5 days in Group B. The 30-day mortality was 0% in Group A and 3% in Group B. Minor complications occurred in the 15% of Group B and in the 8% of Group A. There was the need for re-intervention in 1% of cases in Group A and in 0% of cases in Group B. 

A statistical analysis has been carried out to identify a correlation between the need for conversion and smoking habits and the need for conversion and the occurrence of previous cardiovascular diseases. The analysis identified smoke habitude as a factor statistically related to conversion. 

Conclusions: According to a review published by Power, the most common reasons for thoracotomy are vascular injury, difficulty of lymph node dissection and adhesions [8]. Different adverse outcomes are related to conversion [9,10]. Several independent risk factors of conversion have been identified [11]. In our experience, the incidence of conversion is fairly low and the main cause of conversion is vascular injury. The study identifies smoke habit as a potential risk factor of conversion, associated with increased postoperative complications. VATS anatomic lung resection is a safe procedure in highly experienced surgeon’s hands. The option of conversion needs always to be held. Our experience is aligned with data in the literature. Patients’ selection is a final aspect to consider with the aim of ensuring the best treatment.

### 1.36. Abstract 36 (Oral Presentation): Endoscopic Treatment of Early Complications after Tracheal Surgery: A Single Center Experience

**Beatrice Trabalza Marinucci, Cecilia Menna, Matteo Tiracorrendo, Alessandra Siciliani, Giorgia Piccioni, Fabiana Messa, Anna Maria Ciccone, Antonio D’Andrilli, Giulio Maurizi, Claudio Andreetti, Camilla Vanni, Erino Angelo Rendina and Ibrahim Mohsen**

Thoracic Surgery Sant’Andrea Hospital, La Sapienza University, 00186 Rome, Italy

Background: Tracheal resection-anastomosis is considered the surgery of choice for benign tracheal stenosis, with stable long-term results. Despite judicious pre-operative evaluation, meticulous surgical techniques and accurate post-operative care, complications can occur even in high-volume centers.

Early post-operative complications can be related to anastomotic and non-anastomotic complications. 

The aim of the present study is to present successful endoscopic treatment of early post-operative complications after tracheal surgery in a single center.

Methodology: Between January 2020–December 2023, a total of 208 consecutive patients underwent tracheal and laryngo-tracheal resection-anastomosis in a single center. We excluded: malignant stenosis, carinal resections, Tracheal-Esophageal fistulas. All patients underwent cervical incision. The upper limit of the stenosis ranged between involvement of the vocal cords to 2.5 cm from the glottis. When early post-operative complications were evidenced, a specific endoscopy-guided treatment was planned. Endoscopic treatments included: early re-intubation (after endotracheal tube/laryngeal mask removal) under bronchoscopy guidance with a small nasal-tracheal uncuffed tube left in site 24 h (while judicious dose of steroids was administered), stent insertion and/or dilation with rigid bronchoscopy, and surgical/percutaneous tracheostomy under bronchoscopy guidance. Data were prospectively collected and retrospectively analysed.

Results: Mean length of tracheal resection was 2.52 ± 1.10 cm. Early airway complication rate was 19.7% (41/208): anastomotic dehiscence was described in 1.9% (4/208), recurrent stenosis in 4.3% (9/208), laryngeal edema requiring early re-intubation in 13.5% (28/208).

Permanent tracheostomy was described in 2.4% (5/208). Three patients received temporary tracheostomy because of persistent laryngeal edema, successfully removed after 6 months. 

Stenting was performed in 2 patients for anastomotic dehiscence (Figure 5) and in 3 patients because of re-stenosis (2.4%), and all except 1 were successfully removed after an average time of 24–30 months.

Dilatation was performed in 44% (4/9) of re-stenosis, with optimal caliber restitution. 

The success rate of the endoscopic treatment of restenosis was 89% (8/9). Successful treatment of airway complications was described in 87.8% (36/41).

Conclusions: To the best of our knowledge, our center deals with one of the largest experiences in the world in the surgical treatment of benign tracheal stenosis. The improving results registered over the last years provided excellent outcomes but also a novel insight in the management of the most common early post-operative complications that unfortunately can arise, independently by the accuracy and the meticulous peri-operative management. Our experience suggests that endoscopic treatment of early post-operative complications can ensure definitive success rate, leading to stable results after surgery without any sequelae.

### 1.37. Abstract 37 (Oral Presentation): Analysis of the Risk Factors of Spontaneous Pneumothorax Recurrence after Surgery

**Serena Zanardo, Francesco Londero, William Grossi, Elisa De Franceschi, Gianluca Masullo and Andrea Zuin**

Department of Thoracic Surgery, Ospedale Santa Maria della Misericordia, Udine, Italy

Background: Several studies previously investigated the risk factors for postoperative recurrence of PSP, with conflicting results. Identification of patients at greater risk of recurrence may help optimize therapeutic strategies. The aim of this study is to evaluate the risk factors for postoperative recurrence of PSP and compare our results with the available literature. 

Methodology: We retrospectively evaluated all patients undergoing surgery for PSP in our institution between January 2005 and December 2022. We analysed data on patient characteristics, surgical details, method of pleurodesis and perioperative outcomes and used logistic regression analysis to identify predictors of postoperative ipsilateral recurrence. 

Results: The complete data of 169 patients were obtained after exclusion criteria were applied. Surgery was conducted with VATS technique in 167 patients (99.9%) and surgical pleurodesis was performed in all patients; additional medical pleurodesis was performed in 19 patients (11.2%); 150 patients (88.2%) had at least one bulla resected; 72 (66.1%) patients had a history of ipsilateral PNX and 37 patients (33.9%) of contralateral PNX; intrahospital recurrence occurred in 3 patients. Late recurrence occurred in 19 patients (11.2%). Multivariate analysis revealed that an history of previous episodes of contralateral PNX (*p* = 0.028), and the development of intrahospital PNX recurrence (*p* = 0.043) were independent predictive factors of PNX recurrence.

Conclusions: Particular attention should be paid in the intra-operative identification of all possible sites of aerial loss in order to make the surgery more effective. Especially in patients with a previous history of contralateral pneumothorax.

### 1.38. Abstract 38 (Poster Competition): Prediction of Respiratory Failure in Vats and Rats Lobectomy Using a New Device Monitoring Chest Wall Motion

**Cristiano Casciani ^1^, Federico Tacconi ^1^, Matteo Russo ^2^, Marco Ceccarelli ^2^, Manuel D’Onofrio ^1^, Sebastiano Angelo Bastone ^1,3^, Chiara Combattelli ^1^, Raul Randazzo ^1^, Luciano Cialì Sposato ^1^, Damiano Saggio ^1^, Andra Stefana Sirbu ^1^, Alexandro Patirelis ^1^, Karan Kumar ^1^ and Vincenzo Ambrogi ^1^**

1. Department of Thoracic Surgery, University of Rome Tor Vergata, Via Montpellier 1, 00133, Rome, Italy
2. Department of Industrial Engineering, University of Rome Tor Vergata, Via del Politecnico 1, 00133, Rome, Italy
3. Ph.D. Program in Applied Medical-Surgical, Department of Surgical Sciences, Policlinico Tor Vergata University, Rome, Italy

Background: Continuous monitoring of vital parameters is one of the most stimulating challenges of advanced postoperative care. We have recently developed a new miniaturized, wearable device (RespirHolter^®^) capable of detecting the rib cage motion, using an inertial unit combined with an accelerometer and a gyroscope. In the present we evaluated the efficacy of the new device in detecting postoperative changes in chest wall motion and in predicting respiratory failure after either video- or robot-assisted thoracoscopic pulmonary lobectomy. 

Methodology: The RespirHolter^®^ was placed over the sixth rib of the operated side for 6 h before and immediately after the procedure. Analysis was performed on 20 patients undergoing either video-thoracoscopic (VATS, *N* = 10) or robotic (RATS, *N* = 10) lobectomy, the nights before and the after the operation. 

Data were reduced using a selected significant trait of one minute duration free of evident interferences. Chest wall motion was expressed as a cumulative variable by combining motion along two spatial axes (roll and pitch). The main endpoint of the study was assessment of postoperative changes in chest wall motion. Secondary outcome measures were postoperative changes by surgical approach and correlation with respiratory complications. Non parametric Wilcoxon test was used to compare the changes in roll and pitch angles before and after surgery. The comparison between RATS and VATS outcomes was performed using non parametric Mann-Whitney U test. 

Results: Median age was 73 (61–75). A significant decrease in chest wall motion was found between pre and post operative median values of chest wall motion,0.1563° (IQR: 0.1371°–0.1981°) vs. 0.2270° (IQR: 0.1985°–0.2678°), (*p* < 0.001). Patients who underwent RATS exhibited a less pronounced decrease, −34% (IQR: −39–−33) vs. −38% (IQR: −42–−35), even though statistical significance was not reached (*p* = 0.35). Three patients developed postoperative hypoxemia between postoperative day 1 and 4 (RATS 1, VATS 2). Interestingly, all these patients showed the worst chest wall motion drop immediately after the operation, with a median score significantly lower compared to patients with no respiratory complication, −102% (IQR: −113–−83) vs. −35% (IQR: −34–−33) (*p* = 0.002), of those who did not show post-operative desaturation.

Conclusions: Real-time digital detection of chest wall motion might potentially help detection of early changes allowing timely intervention before development of frank respiratory complications. Our study also showed that changes in chest wall motion might be less pronounced after RATS, yet not significantly. Our data deserve larger investigation in a more appropriate study setting.

### 1.39. Abstract 39 (Poster Competition): The Value of Predicting the Infiltrative Nature and Degree of Infiltration of Pulmonary Ground-Glass Nodules Based on Computed-Tomography Features and Enhanced Quantitative Analysis

**Chiara Catelli ^1^, Susanna Guerrini ^2^, Federico Mathieu ^3^, Floriana Barra ^4^, Miriana D’Alessandro ^5^, Angela Galgano ^3^, Tommaso Ligabue ^3^, Piero Paladini ^3^, Maria Antonietta Mazzei ^4^ and Luca Luzzi ^1^**

1. Lung Transplant Unit, Department of Medical, Surgical and NeuroSciences, Azienda Ospedaliero- Universitaria Senese, Siena, Italy
2. Diagnostic Imaging Unit, Department of Medical Sciences, University of Siena, Azienda Ospedaliero- Universitaria Senese, Siena, Italy
3. Thoracic Surgery Unit, Department of Medical, Surgical and Neuro Sciences, University of Siena, Azienda Ospedaliero-Universitaria Senese, Siena, Italy
4. Diagnostic Imaging Unit, Department of Medical, Surgical and Neuro Sciences and of Medical Sciences, University of Siena, Azienda Ospedaliero-Universitaria Senese, Siena, Italy
5. Department of Medical Sciences, Surgery and Neurosciences, Respiratory Disease and Lung Transplant Unit, Respiratory

Background: To investigate the utility of CT characteristics and enhanced quantitative analysis in predicting the invasive nature and extent of infiltration of pure and mixed GGNs.

Methodology: This retrospective monocentric study included all patients (*n* = 187) who underwent lung resection for adenocarcinomas presenting as GGNs between January 2016 and December 2023. Patients were divided into 3 groups according to GGN’s histology (Invasive adenocarcinoma (IAC), Minimally-invasive adenocarcinoma (MIA) or Adenocarcinoma in situ (AIS). Qualitative (GGN density, margins morphology, bronchus sign, pleural traction sign, type of feeding vessels) and quantitative (GGNs diameter, volume, CT-value in plain and arterial phase of enhancement, the difference CT-value and the Relative Attenuation) CT-parameters were compared; ROC curve analysis was performed for each quantitative data. Binary logistic regression analysis evaluated independent predictors of GGNs invasiveness.

Results: IAC showed a bigger maximum diameter (*p* = 0.001) and lower percentage of GG component than MIA and AIS (*p* < 0.0001). The lepidic component was predominant in AIS (*p* < 0.0001). The acinar component was greater in IAC (*p* < 0.0001) and in MIA (*p* = 0.05) than AIS. The papillary component was more represented in IAC compared to MIA (*p* = 0.02). Pure GGNs correlated more frequently with MIA and AIS (*p* < 0.0001), as well as round margins (*p* = 0.0002). No differences emerged in pleural tag, type of vascularization, bronchus sign, bubble-like lucency. Plein and arterial CT-values of the GG component were higher in AIC than MIA (*p* < 0.0001), but not compared to AIS (plein *p* = 0.146, arterial *p* = 0.287). There were no differences between MIA and AIS GG CT-values (plein *p* = 0.935, arterial *p* = 0.999). No differences were observed in the solid part CT-values, Difference attenuation, Relative attenuation, total volume of the GGNs, volumes of solid and GG components. ROC curve analysis distinguished IAC and non-IAC according to GG component CT arterial phase cut-off value of −506 HU (*p* = 0.001) and CT plain phase cut-off value of −537 HU (*p* = 0.0004). Maximum diameter and percentage of solid component were predictive factors of IAC compared to MIA (respectively OR 0.80, *p* = 0.007, and OR 0.19, *p* < 0.0001) while only the percentage of solid is a predictive factor of IAC compared to AIS (OR 0.72, *p* < 0.0001).

Conclusions: The quantitative analysis in plain and post-contrast enhancement CT scans have important value in predicting the invasiveness of GGNs, in particular between IAC and MIA. The maximum diameter and the percentage of solid component are predictive factors of GGNs invasiveness. This finding could guide the extent of lung resections.

### 1.40. Abstract 40 (Poster Competition): A Novel Scoring System in Endobronchial Ultrasound-Guided Transbronchial Needle Aspiration

**Ilaria Cuccaro ^1^, Krisstopher Richard Flores ^1^, Rossana Vigliarolo ^1^, Federica Olmati ^2^, Giuseppina Gioffrè ^1^, Angela Maria Pia Succu ^1^, Leo Guidobaldi ^3^, Tiziana Trequattrini ^1^, Maria Cristina Zappa ^1^ and Paolo Palange ^2^**

1. Division of Pulmonary Medicine, Sandro Pertini Hospital—Rome (Italy)
2. Department of Public Health and Infectious Diseases, Sapienza University. Division of Pulmonary Medicine, Policlinico Umberto I Hospital—Rome (Italy)
3. Division of Anatomic Pathology, Sandro Pertini Hospital—Rome (Italy)

Background: Endobronchial ultrasound-guided transbronchial needle aspiration (EBUS-TBNA) is a minimally invasive diagnostic examination often requiring a deep sedation of the patient, who needs to be assisted by mechanical ventilation with additional risks to the procedure. In order to reduce procedural time frames, it would be helpful to find a diagnostic tool able to improve the prediction of malignancy. 

Evaluating all the ultrasound (US) features predictive of malignancy in B-mode, Colour-Doppler (CD) and Elastography, we aimed to assess their sensibility, sensitivity, diagnostic accuracy and both predictive values. 

Methodology: We enrolled all the patients above 5 months with mediastinal and hilar adenopathy on chest tomography, candidates for diagnostic EBUS-TBNA (*n* = 38) and prospectively recorded the following lymph node (*n* = 99) US characteristics: size, shape, echogenicity, margins, central hilar structure, coagulation necrosis sign (CNS), CD vascularization pattern and elastography. These features were evaluated by 3 raters and then compared with histopathology. Statistical analysis was performed with IBM SPSS 26.0 software. 

Results: At the multivariate analysis 5 US features revealed to be independent predictors for malignancy (*p* < 0.05): short axis > 1 cm, round shape, heterogeneous echogenicity, distinct margins and CNS present. They were subjected to ROC analysis, obtaining5 items scoring system (scoring from 0 to 5; cutoff point of 3) with a negative predictive value of 91.40%. 

Conclusions: This novel score would eventually allow the ruling out of lymph nodes scoring < 3, thus reducing procedural time and risks. Further studies are required to assess its reliability.

### 1.41. Abstract 41 (Poster Competition): Elective Extra Corporeal Membrane Oxygenation for High-Risk Rigid Bronchoscopy

**Francesco Ferrante ^1^, Ilaria Onorati ^2^, Dana Mihaela Radu ^2^, Sara Lo Torto ^3^, Elena Maiolino ^2^, Yurdagül Uzunhan ^4^, Olivia Freynet ^4^, Charles Juvin ^5^, Guillaume Lebreton ^5^, Olivier Huet ^6^ and Emmanuel Martinod ^2^**

1. Department of Thoracic Surgery, Policlinic Umberto I, University of Rome Sapienza, Rome, Italy
2. Thoracic and Vascular Surgery, Assistance Publique—Hôpitaux de Paris, Bobigny, France
3. Department of Thoracic Surgery, Tor Vergata University Polyclinic, Rome, Italy
4. Pulmonology, Assistance Publique—Hôpitaux de Paris, Bobigny, France
5. Cardiovascular Surgery, Assistance Publique Hôpitaux de Paris, Paris, France
6. Anesthesiology, Assistance Publique Hôpitaux de Paris, Bobigny, France

Background: The use of extracorporeal membrane oxygenation (ECMO) for high-risk rigid bronchoscopy is a poorly studied topic, only reported in few urgent cases. We present our experience with this technique, highlighting ECMO enrolment criteria, performed cases, details of the procedure and follow-up.

Methodology: Between 2016 and 2024, we electively performed nine high risk rigid bronchoscopies under elective veno-venous (VV) ECMO (2% of our Airway Diseases Center case history). We enrolled 5 women and 4 men (mean age 45.6, range 20–67) with potential inability to ventilate the patients during the procedure using conventional techniques due to extensive tracheobronchial lesions or major risk of massive bleeding. All case files were validated by our tracheobronchial diseases multidisciplinary team meeting. 

Results: Different procedures were performed, including extended Y stenting (*n* = 3); incomplete bronchoscopic tumor removal and stenting (*n* = 1); stent replacement (*n* = 3), in situ fenestration of tracheal stent at the level of main left bronchus (*n* = 1); and tracheobronchial dilatation followed by stenting (*n* = 1). In 7 patients VV-ECMO was removed at the end of the procedure. Six patients have tracheal stent in place. With a maximum follow-up of 7 years and 7 months, all patients are alive but one. No long-term complications have been associated with the use of VV ECMO in this series. Patient characteristics including medical history, type of diseases and previous treatment are presented in Table 6.

Conclusions: Elective ECMO support during high-risk rigid bronchoscopy is a valuable therapeutical resource in cases where the procedure is theoretically contraindicated due to inability to ventilate for airway obstruction, extended airway lesions or massive bleeding. 

### 1.42. Abstract 42 (Poster Competition): Surgical and Neurological Outcomes in Robotic Thymectomy for Myasthenic Thymomatous Patients

**Khrystyna Kuzmych, Dania Nachira, Alessia Senatore, Maria Teresa Congedo, Marco Chiappetta, Filippo Lococo, Carolina Sassorossi, Stefano Margaritora and Elisa Meacci**

Department of Thoracic Surgery, Fondazione Policlinico Universitario A.Gemelli IRCCS, Università Cattolica del Sacro Cuore, Rome, Italy

Background: The main aim of this study was to evaluate surgical and neurological outcomes of patients undergone robotic thymectomy (RATS) for thymoma. In Myasthenic patients, neurological outcomes were also analyzed. 

Methodology: Among 128 Robotic thymectomies performed at our center from October 2013 to January 2022, the clinical-pathological data of 55 patients with diagnosis of thymoma were reviewed. Thirty (54.5%) patients had concomitant acetylcholine-receptor-antibody-associated Myasthenia Gravis (MG). The Myasthenia Gravis Foundation of America post-intervention score (MGFA-PIS) was used to assess neurological outcomes. 

Results: Thirty-nine (70.9%) procedures were performed from the left side. The mean surgical duration was 196.9 ± 79.9 min in MG patients vs. 175.8 ± 61.6 min in non-MG (*p*: 0.285), with a longer in-hospital stay (4.8 ± 2.6 vs. 3.3 ± 2.2 days, *p*: 0.01) and a significant necessity of ICU admission (*p* < 0.01) compared to the other group. Mortality was null. Conversions (3.3% vs. 4.0%, *p*: 0.895) and complications (*p*: 0.813) were similar in myasthenic and non-myasthenic thymomas. At multivariable analysis, the main risk factors for conversion and complications were lung involvement (*p*: 0.023) and extended resection (*p*: 0.019), respectively. 

The mean age of surgery for MG patients was 54.5 ± 15.9 years. After a mean follow-up of 35.6 ± 25.7 months, 18 (60%) myasthenic patients had an improvement of their clinical conditions: complete stable remission (CSR) was observed in 2 (6.6%) patients, pharmacological remission (PR) in 2 (6.6%), minimal manifestation (MM) in 12 (40.0%), PR + MM in 4 (13.3%). Twelve patients (40%) showed a stable condition. No worsening was recorded. The 2- and 5-year overall improvement rate was 50% and 90%, respectively. 

Conclusions: RATS thymectomy is safe and feasible treatment in thymomatous patients. Patients with concomitant MG may benefit from a good rate of neurological improvement.

### 1.43. Abstract 43 (Poster Competition): Late Haemothorax in Patients Experiencing Post-Traumatic Rib Fractures: Analysis of Risk Factors and Clinical Management Implications

**Francesco Londero, Alberto Di Rienzo, Jacqueline Cinel, Elisa De Franceschi, William Grossi, Gianluca Masullo, Andrea Zuin**

Thoracic Surgery Unit—Cardiothoracic Department, Azienda Sanitaria Universitaria Friuli Centrale, Presidio Ospedaliero S Maria della Misericordia, Udine, Italy

Background: To assess the incidence of late haemothorax among patients sustaining post-traumatic rib fractures and identify predictors of occurrence.

Methodology: A retrospective analysis of patients who sustained post-traumatic rib fractures and evaluated during the interval 2018–2021 at our institution was performed. Subjects deceased within the first 24 h or with a follow up time lower than 30 days were excluded from the analysis. Demographic and clinical data and information regarding modality of trauma, associated injuries and early treatments were collected. CT scans performed at admission were reviewed to assess number of rib fractures, number of fracture points and kind of fracture (aligned/displaced). A Cox multivariate analysis was performed to identify determinants of late haemothorax. Kaplan-Meyer curves were used to depict differential incidence of haemothorax among groups subdivided according to identified cut-offs.

Results: During the time interval 295 patients fulfilled inclusion criteria and were included in the study. Of them, 74.2% were male and median age was 60 years [(QR 45–74). Most traumas were due to road traffic accidents (56.9%) and domestic accidents (25%). In 37.5% of cases a bilateral involvement was observed. Median number of rib fractures was 6 (IQR 4–9). Late haemothorax occurred in 28 (9.5%) patients after a median time of 5 (IQR 4–11) days from trauma. At Cox multivariable analysis age (HR 1.03, *p* = 0.022), number of displaced rib fractures (HR 1.14, *p* = 0.037), necessity for early intubation (HR 2.88, *p* = 0.050) and early chest tube thoracostomy (HR 0.12, *p* < 0.001) were significant predictors of late haemothorax.

Conclusions: A rigorous follow up should be undertaken in patients who suffered severe chest trauma, especially in case of multiple displaced rib fractures or with untreated air and fluid pleural collection, in order to timely identify late complications and reduce further related risks.

### 1.44. Abstract 44 (Poster Competition): Uniportal Robotic Thoracic Surgery (U-Rats) Major Pulmonary Resections: Single Center Advanced Experience

**Nicola Martucci, Mary Bove, Giorgia Opromolla, Giuseppe De Luca, Antonello La Rocca, Carmine La Manna and Edoardo Mercadante**

Thoracic Surgery Unit, Istituto Nazionale Tumori IRCCS Fondazione G. Pascale, Naples, Italy

Background: Minimally invasive surgery has developed rapidly over the past 30 years. In the present era, even complex major lung resections can be performed by VATS using a single small incision of 4 cm or less (Uniportal-VATS). Robotic-Assisted Thoracoscopic Surgery (RATS) is an alternative to VATS for lung lobectomy, with the advantages of 3D vision and the use of small-wristed instruments that can facilitate complex movements in the chest. RATS is routinely performed using three or four ports with at least one service incision, in contrast to the real concept of less invasiveness. The aim of the study is to describe the results of our advanced experience with U-RATS. 

Methodology: From January 2022 to April 2024, 120 patients (68 males and 52 females, mean age 67 years) underwent U-RATS anatomical lung resections with systematic lymphadenectomy. We performed 108 lobectomies and 12 segmentectomies. All patients underwent U-RATS with 3 arms da Vinci Xi robotic system. Postoperative pain was recorded using the Numeric Pain Rating Scale (NRS).

Results: All the procedures but two were completed with the uniportal robotic technique. Mean total operative time was 156 min (range 65–350 min). In the last 10 cases, the operative time was significantly reduced to 122 min, according to the improving learning curve. In 80% of cases, chest drain was removed in the third postoperative day. Mean hospital stay was 4 days. In two cases the approach was converted to VATS (1 patient) and to thoracotomy (1 patient), for intraoperative major bleeding. No intraoperative or perioperative mortality were observed. The postoperative NRS was evaluated and resulted comparable to our experience with U-VATS.

Conclusions: U-RATS is a safe and feasible technique, combining the advantages of U-VATS with the well-known advantages of robotic surgery. The technique is not simple but in experienced U-VATS and robotic surgeons, the learning curve is quite rapid and can allow a wide spreading of the technique.

### 1.45. Abstract 45 (Poster Competition): Modified Blade: An Interventional Option in Rigid Bronchoscopy for Non-Resectable Benign Tracheal Stenosis

**Messina Gaetana, Giuseppe Vicario, Riccardo Vinciguerra, Davide Pica, Francesca Capasso, Vicenzo Di Filippo, Beatrice Leonardi, Francesco Leone, Maria Antonietta Puca, Maria Marvulli, Noemi Giorgiana, Alessia Caputo, Giovanni Natale, Giovanni Vicidomini and Alfonso Fiorelli**

UOC Chirurgia Toracica Università degli studi della Campania ‘Luigi Vanvitelli’ Napoli

Background: Benign tracheobronchial stenosis is a abnormal tracheal lumen narrowing that may incur progressive dyspnea and life-threatening hypoxemia. There is no consensus on which patients should be treated with endoscopic or surgical method. This study investigates the outcomes of bronchoscopic dilatation in the treatment of benign tracheal stenosis using a device equipped with a blade to cut the stenotic lesions with dense fibrosis. 

Methodology: The procedure was carried out in an operating room under general anesthesia. All patients were intubated with a Rigid Bronchoscope (RB) placed just above the stenosis. Through Rigid Bronchoscopy combined modalities were used as needed: radial incisions of the mucosal stenosis with blade at the levels of 4, 8 and 12 o’clock, with back-and-forth movements, then the stenotic area was dilated more easily with a rigid bronchoscope. Dilatation was performed by passing the RB of increasing diameter through stenotic areas and then Balloon dilatation of increasing diameter. There were no complications during the procedure. 

Results: We conducted an observational, retrospective, single-centre study in the Thoracic Surgery Unit of the University of ‘Luigi Vanvitelli’ of Naples from November 2014 to September 2023. We included all consecutive patients with benign tracheal stenosis inoperable. During the study period, 113 patients were referred to our department with benign tracheal stenosis inoperable. 61 patients were treated with the blade. During the follow-up, a recurrence of the stenosis was observed in 8 patients in the first month and in 4 patients in the third month. Instead in the patients treated with the use of laser (52 patients), during the follow-up a recurrence was observed in 16 patients in the first month and in 6 patients in the third month; no patient relapsed after 6 months and after 1 year. Long term successful bronchoscopic management with blade was attained by 99% in simple and 93% in mixed stenosis and in complex type stenosis. 

Conclusions: Our study underlines the importance of the use of the blade in bronchoscopic treatment as a valid conservative approach in the management of patients with inoperable benign tracheal stenosis as an alternative to the use of the laser, reducing the abnormal inflammatory reaction in order to limit recurrences.

### 1.46. Abstract 46 (Poster Competition): Oncological Outcomes and Prognostic Factors of Pulmonary Metastasectomy in Pancreatic Cancer

**Dania Nachira ^1^, Giuseppe Calabrese ^1^, Luca Bertolaccini ^2^, Elisa Meacci ^1^, Maria Teresa Congedo ^1^, Pierluigi Novellis ^3^, Angela De Palma ^4^, Rosalia Romano ^5^, Pietro Bertoglio ^6^, Gianmaria Ferretti ^7^, Jessica Evangelista ^1^, Matteo Chiari ^2,8^, Francesca Misceo ^3,9^, Francesco De Blasi ^4^, Mariangela Valentini ^4^, Leonardo Valentini ^6,10^, Piergiorgio Solli ^6,10^, Marco Taurchini ^7^, Giuseppe Bogina ^11^, Giulia Veronesi ^3,12^, Lorenzo Spaggiari ^2,8^ and Stefano Margaritora ^1^**

1. Department of Thoracic Surgery, Fondazione Policlinico Universitario A.Gemelli IRCCS, Università Cattolica del Sacro Cuore, Rome, Italy
2. Division of Thoracic Surgery, IEO, European Institute of Oncology, IRCCS, Milan, Italy
3. Division of thoracic surgery, IRCCS San Raffaele Scientific Institute, Milan, Italy
4. Unit of Thoracic Surgery, Department of Precision and Regenerative Medicine and Ionian Area, University of Bari, Bari, Italy
5. Division of Thoracic Surgery, IRCCS Sacro Cuore-Don Calabria Hospital, Verona, Italy
6. Division of Thoracic Surgery, IRCCS Azienda Ospedaliera Universitaria Bologna, Bologna, Italy
7. Division of Thoracic Surgery, SS. Annunziata Hospital, Taranto, Italy
8. Department of Oncology and Hemato-Oncology, University of Milan, Milan, Italy
9. Università Vita-Salute San Raffaele, Milan, Milan, Italy
10. Alma Mater Studiorum, Università di Bologna, Bologna, Italy
11. Pathology Anatomy, IRCCS Sacro Cuore-Don Calabria Hospital, Verona, Italy
12. Università Vita-Salute San Raffaele, Milan, Italy

Background: The number of long-term survivors after pancreatic cancer is increasing due to recent advances in diagnosis and multidisciplinary treatments. However, the effectiveness of pulmonary metastasectomy remains uninvestigated. This study aims to evaluate the oncological outcomes and to identify potential prognostic factors of pulmonary metastasectomy in pancreatic cancer.

Methodology: Patients who had undergone pulmonary metastasectomy were accurately selected among patients with radical pancreatic cancer surgery and no evidence of disease in other organs. Clinical data of 56 selected patients from 7 high volume centers were retrospectively analyzed. Long-term oncological outcomes and prognostic factors were evaluated.

Results: Five- and 10-year OS from pancreatectomy was 76% and 42%, respectively. Multivariable analyses confirmed as negative prognostic factors for OS: male sex (HR = 25.7, 95%CI: 3.5–190.2, *p* = 0.001), major lung metastasis diameter > 1.3 cm (HR = 50.8, 95%CI: 3.7–692.2, *p* = 0.003) and synchronous metastases with primary cancer (HR = 61.2, 95%CI: 3.1–1203.2, *p* = 0.007). 

The five-year DFI from pancreatectomy and first lung metastasis was 13%, with a median of 32 months. The only prognostic factor affecting DFI was pN2 pancreatic disease (*p* = 0.008) at multivariable analysis. The 1-year DFI between first and second lung metastasis was 9%, with a median of 11 months. 

The 5-year OS from the first pulmonary metastasectomy was 47%. The main prognostic factors at univariable analysis for OS after first metastasectomy were: pT (*p* < 0.001), RT/CT neoadjuvant or adjuvant therapy after pancreatectomy (*p* = 0.01), major lung metastasis diameter > 1.3 cm (*p* = 0.04) and reiterative lung surgery for pulmonary recurrence after first metastasectomy (*p* = 0.04). No factor was confirmed in the multivariable analysis.

Conclusions: Pulmonary metastasectomy after radical surgery for pancreatic cancer seems to be a valuable treatment in well-selected patients. Furthermore, some patients, such as females with metachronous metastases smaller than 1.3 cm, would benefit from a better prognosis. Reiterative pulmonary surgery could also have an essential role in selected cases. Further studies are needed to confirm our results.

### 1.47. Abstract 47 (Poster Competition): Postoperative Incentive Spirometry Versus Early Ambulation in Resected Lung Cancer Patients: Propensity Score-Matched Analysis

**Riccardo Orlandi ^1^, Monica Casiraghi ^2^, Antonio Mazzella ^2^, Luca Bertolaccini ^2^, Giorgio Lo Iacono ^2^, Lara Girelli ^2^, Cristina Diotti ^2^, Giovanni Caffarena ^2^ and Lorenzo Spaggiari ^2^**

1. Department of Thoracic Surgery, University of Milan, Milan, Italy.
2. Department of Thoracic Surgery, IEO, European Institute of Oncology IRCCS, Milan, Italy.

Background: Postoperative physiotherapy is considered a key element of any Enhanced Recovery After Surgery (ERAS) program, even more after lung resection. Nonetheless, literature still surprisingly lacks clear recommendations on this topic and unambiguous guidelines are demanded. In particular, the role of incentive spirometry in patients undergoing lung resection is yet to be clarified. The aim of this study was to investigate whether in resected lung cancer patients the addition of incentive spirometry could be beneficial over early-ambulation alone.

Methodology: We designed a retrospective case-control study, enrolling patients who underwent lung resection for primary lung cancer from June 2020 to June 2022, at European Institute of Oncology (IEO), Milan, Italy. Patients were divided into two cohorts, based on the adopted postoperative physiotherapy protocol: early ambulation alone compared to early ambulation together with incentive spirometry. Primary endpoint was the rate of postoperative pulmonary complications between the two groups, whereas secondary endpoints included length of hospital stay and time to chest drain removal. Propensity score-matched analysis was performed, based on age, sex, and BMI. Descriptive data are reported as number and percentages for categorical variables, or median and ranges for continuous variables, and compared by Chi-squared test, or Student T test, as appropriate.

Results: A total of 304 patients were enrolled, 153 in the intervention group, 151 in the control cohort. After propensity-score matching, 52 patients in each cohort have been compared: 48.1% were female, median age was 65, median BMI was 25.4. Preoperative, operative, and postoperative characteristics are reported in Table 7. No difference was noted between the two groups in terms of postoperative oxygen requirement (17.3% vs. 11.5%, *p* value 0.40), fever (17.3% vs. 7.7%, *p* value 0.14), atelectasis (1.9% vs. 1.9%, *p* value 1.00), residual pneumothorax (78.8% vs. 69.2%, *p* value 0.26), need for bronchoscopy toilette (9.6% vs. 9.6%, *p* value 1.00), and re-hospitalization rate (5.8% vs. 3.8%, *p* value 0.65). 

Though the incentive spirometry group showed a trend towards shorter length of hospital stay (4.5 days vs. 5 days), and lower time to chest drain removal (3 days vs. 4 days) compared to the control group, both variables did not reach statistical significance (*p* value 0.82 and 0.49, respectively).

Conclusions: Incentive spirometry did not significantly enhance postoperative outcomes of patients undergoing lung resection for primary lung cancer, compared to early ambulation alone, even if slightly lower length of hospital stay and time to chest drain were evident in the interventional group. Larger, prospective, and randomized trials are demanded for definitively confirming these results.

### 1.48. Abstract 48 (Poster Competition): Prognostic Factors in Pn1 Nsclc Affected Patients: A Multicentric Prospective Study

**Carolina Sassorossi ^1^, Marco Chiappetta ^1^, Filippo Lococo ^1^, Alessia Senatore ^1^, Franco Facciolo ^2^, Filippo Gallina ^2^, Giuseppe Cardillo ^3^, Sara Ricciardi ^3^, Francesco Guerrera ^4^, Eleonora Della Beffa ^4^, Marco Lucchi ^5^, Vittorio Aprile ^5^, Giulia Veronesi ^6^, Pierluigi Novellis ^6^, Riccardo Di Fonzo ^6^, Marco Alloisio ^7^, Emanuele Voulaz ^7^, Ludovic Fournel ^8^, Thomas Charrier ^8^ and Stefano Margaritora ^1^**

1. Thoracic Surgery Unit, Università Cattolica del Sacro Cuore## Fondazione Policlinico, Universitario A. Gemelli, IRCCS, Rome, Italy
2. Thoracic Surgery Unit, Istituto Nazionale Tumori “Regina Elena”, Rome, Italy
3. Thoracic Surgery Unit, Ospedale San Camillo Forlanini, Rome, Italy
4. Thoracic Surgery Unit, Università di Torino, Turin, Italy
5. Thoracic Surgery Unit, Università di Pisa, Pisa, Italy
6. Thoracic Surgery Unit, San Raffaele Hospital, Milan, Italy
7. Thoracic Surgery Unit, Humanitas Hospital, Milan, Italy
8. Thoracic Surgery Unit, APHP, Centre Université de Paris Cité, site Cochin, Paris, France

Background: The aim of this study is to describe the prognostic factors for survival in pN1 NSCLC patients who underwent surgical treatment for NSCLC, with particular interest on nodal characteristics and pathology.

Methodology: Data of patients surgically treated for NSCLC in 8 centres between 1 May 2020 and 30 June 2023 that resulted to be pN1, were prospectively collected and analysed. Patients who underwent wedge resection, with distant metastasis at the time of surgery, and with incomplete data about the lymphadenectomy, were excluded.

The primary outcome was Disease free survival (DFS), calculated as the time between the surgery and the recurrence occurrence. Clinical and pathological characteristics were described using descriptive statistics. Clinical and pathological characteristics were associated to DFS using Kaplan-Meier curves and log rank analysis.

Results: The final analysis was conducted on 158 pN1 patients that met the inclusion criteria. Clinical and pathological characteristics are reported in Table 8. 

At preoperative, 112 (70.9%) were cN0 and 46 (29.1%) were cN1. The majority of tumours had dimension between 2 and 3 cm. Lymphatic invasion was present in 46 (29.1%) patients. STAS was present in 26 (16.5%) patients. In the majority of cases (12,176.6%) more than 1 hilar station and more than 3 mediastinal station (8352.5%) were removed. At univariate analysis, cN1 (*p* = 0.05) and lymphatic invasion presence (*p* = 0.03) were significantly correlated to worse DFS (Figure 6). 

In detail, 3YDFS resulted 66.1% vs. 44% in cN0 vs. c N1 patients and 51.4% in patients with lymphatic invasion vs. 72.7% in patients without. No significant association was found between DFS and the dimension, the number of removed hilar and mediastinal stations and removed nodes and the number of positive hilar stations.

Conclusions: Our study showed a survival advantage in patients pre-operatively cN0 patients and without lymphatic invasion at the pathological analysis. These parameters may be further validated to plan ad hoc post-operative treatments. 

### 1.49. Abstract 49 (Poster Competition): Why Don’t We Do More Superior Bilobectomy for Right-Sided Tumours? How Reliable Is the Horizontal Fissure Boundary?

**Sara Ugolini, Gowthanan Santhirakumaran and David Waller**

Barts Thorax Centre, St Bartholomew’s Hospital, Barts Health NHS Foundation Trust, London, UK

Background: The reliance on conventional anatomical boundaries may result in potential discrepancy in the treatment of upper lobe tumours on the right and left. The reason given is that there is well developed Horizontal Fissure (HF) on the right but not on the left. We aimed to test this hypothesis.

Methodology: We retrospectively analysed anatomical lung resections performed in 2019–2023 for <4 cm 

T1a-T2a primary lung cancer and StratX reports of consecutive patients undergoing assessment for lung volume reduction in 2023. A comparison was made between the number of resected segments on the right side and left side (*t* test) for: (I) entire sample, (II) subpopulation of “upper resections” (lower lobes excluded). We recorded the estimate of HF integrity and correlated this with the emphysema severity in the right upper and middle lobes as estimated by the %voxel density < 910 HU (Pearson coefficient). 

Results: 584 resections were included: 43.8% males (256/584), age 68.8 years (±9.7). The number of resected segments was 2.8 (±1.35). 57.36% (335/584) were right-sided. The number of resected segments on the right/left side was of 2.67 (±1.31) and 2.99 (±1.39) respectively. The difference was of 0.32 segments (±0.11) (*p* = 0.004). Among the “Upper resections” 67.12% (392/584)], 58.67% (230/392) were right-sided. The number of resected segments on the right/left side was of 2.4 (±0.79) and 3.08 (±1.39) respectively. The difference was of 0.66 segments (±0.1) (*p* = 0.001). 

In a population of 100 ex-smokers with severe emphysema (males 52% (52/100), age 68.6 years (±7.2)) the HF integrity was 76.8% (±17.47%). *N* = 4 (4%) had a 100% complete HF. The severity of emphysema in the right upper and middle lobes was 54.25% (±16.65%) and 46.25% (±14.89%) respectively. No correlation was found between HF integrity and severity of emphysema (Pearson Index: RUL = 0.152; RUML = 0.153; RML = 0.044) (Figure 7).

Conclusions: In a population of anatomical lung resections performed at a single centre, we found a statistically significant higher number of segments removed on the left side. The finding was retained in the “upper resections”. There was no correlation between severity of disease and HF integrity. The excessive conduct of a left upper lobectomy rather than trisegmentectomy when compared to the treatment of similar right sided tumours cannot be supported by the belief in the anatomical barrier of the HF, since it is rarely complete.

### 1.50. Abstract 50 (ePoster): Lung Nodules Localization through Radionucleotide Tracer

**Riccardo Amatucci ^1^, Claudia Colafigli ^2^, Salvatore Messina ^3^, Mara Romito ^1^, Jacopo Vannucci ^1^ and Francesco Puma ^1^**

1. Thoracic Surgery, University of Perugia
2. Interventional Radiology Perugia
3. Nuclear Medicine, Perugia

Background: Minimally invasive lung resection modern techniques can lead to difficult intraoperative localization of low-density or pleural-surfacing nodules. Different methods are used to guide the surgeons, but there is no technique considered to be a “gold standard”. We report our experience using CT-guided radiotracer percutaneous injection (technetium-labelled macroaggregated albumin, MAAs) and SPECT monitoring.

Methodology: After years of experience using CT-guided metal coil tracers, recently we modified our technique to better localize lung nodules using MAAs pre-operative percutaneous and CT-guided injection. In this project are involved the Nuclear Medicine, Interventional Radiology and Thoracic Surgery. The nuclear medicine specialist defines the radionucleotide’s dose to be injected and takes care of the following monitoring thought scintigraphy and spectrometry. The target is identified with CT-scan and marked by the radiotracer injection thought fine needle (20 G) the same day of surgery. Nodule identification is done during videothoracoscopic surgery using a “gammaprobe”. This last is used also on the resected lung portion and on the residual suture line to verify the complete lesion asportation and resection.

Results: The technique has allowed an easy and rapid target localization in all cases. This included a patient with post-procedural pneumothorax and mild radiotracer leaking from the visceral pleura. The procedure can be performed even hours before surgery with patient’s moderate discomfort. A radionucleotide dose equals to 0.2 milliCurie is used. The radioactivity absence from the surgical field after resection has always been related to histological proof.

Conclusions: Compared to nodule localization through metal coil tracers, the proposed technique presents the following advantages: lower patient’s discomfort; wider utilization time range with better planning of other surgeries, which are not hampered by the procedure timing; no limitation related to patient’s BMI, to the emphysema severity and to the anatomical nodule localization (posterior side or behind the shoulder blade). The radionucleotide dose does not lead to any radiological risk for the patient and for the equipe. The only disadvantage is connected to the increased cost and to the essential nuclear medicine specialist involvement during surgery.

### 1.51. Abstract 51 (ePoster): Ex Vivo Confocal Laser Scanning Microscopy: Beyond Touch Imprint Cytology for Rapid On-Site Evaluation of Cryobiopsies

**Gian Piero Bandelli ^1^, Marco Ferrari ^1^, Chiara Bucchi ^2,3^, Francesca Giunchi ^4^, Mattia Riefolo ^4^, Martina Ferioli ^1^, Thomas Galasso ^1^, Filippo Natali ^1^, Stefania Damiani ^4^, Tommaso Abbate ^1^ and Piero Candoli ^1^**

1. Interventional Pulmonology Unit, IRCCS Azienda Ospedaliero-Universitaria di Bologna, Italy
2. IRCCS Azienda Ospedaliero-Universitaria di Bologna, University Hospital Sant’Orsola-Malpighi—Respiratory and Critical Care Unit, Bologna, Italy
3. Alma Mater Studiorum, Department of Medical and Surgical Sciences (DIMEC), University of Bologna, Italy
4. Pathology Unit, IRCCS Azienda Ospedaliero-Universitaria di Bologna, Department of Medical and Surgical Science (DIMEC), Italy

Background: Rapid on-site evaluation (ROSE) is commonly used to examine cytology specimens. The recently published joint guideline by Roy-Chowdhuri and colleagues [12]) issued strong advice to include the use of touch imprint cytology (TIC) preparations of histologic samples for ROSE. A published study by Muto and colleagues indicated that histologic transbronchial cryobiopsy samples could be assessed using ROSE with promising results [13] However, the study was performed in a monomodality sampling setting that analyzed only cryobiopsies. In addition, other studies have reported on the possible tissue-depletion effect caused by TIC processing, which could result in a lower diagnostic yield. Finally, in a recent study published by Kops and colleagues, it was not specified the concordance between TIC-ROSE and final pathology when TIC was derived from cryobiopsies [14]. In this study we wanted to explore the possibility of carrying out a ROSE of cryobiopsies using ex vivo confocal laser scanning microscopy (CLSM).

Methodology: A retrospective analysis was conducted on patients that underwent transbronchial cryobiopsies for suspected lung lesions at our institution from November 2023 to May 2024, with concurrent analysis of cryobiopsies in the endoscopic room via ex vivo CLSM VivaScope 2500 M (VivaScope GmbH, Munich, Germany). 

TIC of criobiopsies was performed in all cases placing them on a microscope slide and then rolling them over the slide.

All cryobiopsies have been performed under the guidance of Lung Vision (LV, Body Vision Medical LDT, Campbell, CA, USA) and using 1.1 mm cryoprobe (Erbe Elektromedizin GmbH, Tübingen, Germany) with a freezing time of 6 s. Thus, the CLSM analysis results were compared with final pathology. 

Results: The study included 7 patients. The lesions had an average size of 19.6 mm (range: 9–53 mm) and were mainly located in the upper lobes (85.7%) and in the median third of the lung (85.7%). A bronchus sign was present in all cases, mainly compressed/adjacent (71.4%). 

In 2 cases, TIC correlated with the final diagnosis (no neoplasia); in other cases, TIC showed the presence of an inadequate sample (absence of cells or only bronchial cells). 

In 5 patients, CLSM perfectly correlated with the final diagnosis (presence of neoplasia in 4 cases, mostly adenocarcinoma, Figure 8). 

In 2 cases, CLSM showed suspicious findings, with adenocarcinoma confirmed in one case at the final diagnosis.

Conclusions: Ex vivo CLSM may be a promising method of performing ROSE on cryobiopsies, avoiding tissue depletion or no cell release during TIC.

### 1.52. Abstract 52 (ePoster): Routinely Obtained C-Reactive Protein Level after Elective Surgery for Lung Cancer Can Be Omitted in Most Patients: A Retrospective Observational Study

**A. Bellini^1^; E. Passone ^2^, S. Sterrantino ^1^, S. Parini ^2^, F. Migliano ^2^, AP. Ciarrocchi ^1^, S. Mazzarra ^1^, KM. Amores Naranjo ^1^, S. Congiu ^2^, D. Argnani ^1^, O. Rena ^2^ and F. Stella ^1^**

1. Department of Diagnostic and Specialty Medicine, University of Bologna, Thoracic Surgery Unit, Morgagni-Pierantoni Hospital, Forli, Italy
2. Thoracic Surgery, University Hospital Maggiore della Carita, Novara, Italy

Background: The patients’ readmission is one of the major issues both for retreatement of patients and hospital budgeting. After minimally invasive pulmonary lobectomy for lung cancer, some post-operative complications have a greater risk of readmission, such as empyema and pneumoniae. The aim of this study is to analyse if assessment of C-reactive protein (CRP) has a role in predict the chance of readmission for infective complications.

Methodology: In this bicenter study, we retrospectively reviewed 280 patients who underwent pulmonary lobectomy for lung cancer from January 2020 and June 2023. 

Exclusion criteria were advanced NSCLC, lymphoproliferative disorders, carcinomatous lung abscess, readmission for non-infectious reasons, open surgery. 

We collected baseline characteristics of patients, respiratory function, ASA, type and duration of surgery, histology, p-stage, infective complications and 30- and 60-day readmissions.

CRP and GB count were checked routinely on post-operative day 1 and at discharge, regardless of the clinical conditions of the patient. At discharge all patients were afebrile.

Continuous and categorical variables were reported as median (IQR) and number (%), respectively; Fisher exacts and MannWhitney test were used for comparison and univariable logistic regression was employed to identify risk factors for RC.

Results: Sixty-day readmission rate was 3.2%: out of 280 patients, 9 of them were readmitted to the hospital. In particular, empyema 5 cases, pneumoniae 4 cases.

The characteristics of patients are summarized in Table 9.

At univariable analysis, readmission occurred mostly in older patients, HR 1.19 (CI95% 1.04–1.35), *p* = 0.010, while no differences were found for preadimission GB count, HR 1.00 (CI95% 0.99–1.00) *p* = 0.635, and CRP levels [HR 1.99 (CI95% 0.98–1.01), *p* = 0.793], increasing trend of GB, HR 0.85 (CI95% 0.17–4.20) *p* = 0.845, and CRP, HR 0.80 (CI95% 0.21–3.06) *p* = 0.752.

Due to the small number of the events, multivariable analysis was not performed.

Conclusions: Our study shows that routinely assessments of CRP and GB count are not valuable predictors of readmission, thus a more individual approach should be encouraged in order to avoid a waste of resources, bearing in mind that older patients are more likely to risk readmission for infective complications.

### 1.53. Abstract 53 (ePoster): Tracheobronchial Foreign Body Aspiration in Adults: Twenty-Year Single Center Experience

**Graziana Carleo, Debora Brascia, Mirko Girolamo Cantatore, Maria Luisa Zhurda, Naomi Savarelli, Loredana D’Aucelli, Ondina Pizzuto, Rosatea Quercia, Marcella Genualdo and Angela De Palma**

Thoracic Surgery Unit, Department of Precision and Regenerative Medicine and Ionian Area, University of Bari “Aldo Moro”, Bari, Italy

Background: Tracheobronchial foreign body (TFB) aspiration is a frequent clinical emergency in children. Population aging and adult disability have led to an increased incidence of TFB aspiration in adults. The aim of this study was to identify the type of adult patient most susceptible to occurrence of TFB aspiration and to evaluate intervention modalities for TFB removal. 

Methodology: Data of adult patients with TFB aspiration admitted to our Unit of Thoracic Surgery from 2004 to 2024 were retrospectively collected: gender, age, predisposing factors, type of inhalation, treatment, location and nature of FBs, complications, hospital stay. Flexible fiberoptic bronchoscopy was performed in all patients.

Results: Twenty-nine patients, 26 (90%) males, 3 (10%) females, mean age 61 years, with TFB aspiration were treated. No predisposing factors were detected in 23 (79%); the most common ones were tracheostomy (2; 7%), psychiatric disease (2; 7%), neoplastic disease involving the trachea (1; 3.5%) and mental retardation (1; 3.5%). Inhalation was acute in 16 (55%) cases, subacute in 7, chronic in 6. Flexible fiberoptic bronchoscopy was performed urgently in 17 (59%), electively in 12 (41%), allowing TFB removal in all cases. The TFB was located in the right bronchi in 22 (76%), in the left in 7 (24%) and was organic in 14 (48%), inorganic in 15 (52%) (Figure 9). 

Immediate post-procedural complications occurred in 3 patients only, including mild bleeding in one case which resolved with medical therapy. Mean hospital stay was 3 days. 

Conclusions: In our experience, most adult patients did not show any predisposing factors favoring TFB aspiration. Flexible fibreoptic bronchoscopy proved to be a safe and effective tool for the removal of TFBs in adults. 

### 1.54. Abstract 54 (ePoster): Virtual Chromobronchoscopy for Intraoperative Assessment of the Airway in Vats Lobectomy

**Roberto Cascone, Annalisa Carlucci, Domenico Caporale, Gabriella Giudice and Cosimo Lequaglie**

Department of Thoracic Surgery, IRCCS-CROB Centro di Riferimento Oncologico della Basilicata, Rionero in Vulture, PZ, Italy

Background: lobectomy represents the therapeutic option of choice for the treatment of non-small cell lung cancer (NSCLC). In some cases, the neoplasm can be localized near the bronchus, with the possibility of having an incomplete resection (R1) and leaving microscopic foci of disease at the level of the bronchial suture after lung resection. This event is minimized with cryostat examination of the bronchial section. In our study we wanted to analyze the usefulness of virtual chromobronchoscopy used intraoperatively for the evaluation of the bronchial mucosa before using the stapling device, with the aim of guiding the suture to a point likely to be free of disease.

Methodology: the data of 26 consecutive patients from January 2022 to December 2023 were retrospectively analyzed. The study population had a diagnosis of lung cancer localized near the lobar bronchus which was treated with a minimally invasive technique. Before stapling the lobar bronchus, a bronchoscopic evaluation was performed through the orotracheal tube, with image enhancement using virtual chromobroncoscopy. This method, highlighting anomalies of the bronchial mucosa, guided the surgeon in the optimal positioning of the stapler to reduce the possibility of R1 resection. In all cases, cryostat examination of the bronchial stump was always performed to confirm the absence of neoplasia.

Results: of the 26 patients undergoing pulmonary lobectomy for NSCLC, the cryostat examination of the bronchial suture was negative in the entire study population, agreeing with the enhanced imaging of the bronchoscopy performed before the use of the stapler. In the immediate post-operative period, no patient developed bronchopleural fistulas. Persistent air leaks were present in 2 patients, in whom the fistula etiology was excluded by bronchoscopy performed during hospitalization. At the 6-month follow-up, all patients were still without radiological and clinical evidence of bronchopleural fistula.

Conclusions: the use of virtual chromobronchoscopy, a real-time enhanced imaging method, during a VATS lobectomy operation can represent a valid guide for the surgeon in stapling the bronchus when the tumor is in close relationship with it, allowing to obtain a R0 resection and, in fact, reducing the possibility of bronchopleural fistula.

### 1.55. Abstract 55 (ePoster): Unilateral Diaphragmatic Paralysis (Udp): Pperioperative Outcomes of Robot-Assisted (Rats) Diphragmatic Plication Tecnique

**Chiara Catelli ^1^, Federico Mathieu ^2^, Susanna Guerrini ^3^, Maddalena Messina ^4^, Alessio Campisi ^5^, Piero Paladini ^3^, Maria Antonietta Mazzei ^6^ and Luca Luzzi ^1^**

1. Lung Transplant Unit, Department of Medical, Surgical and NeuroSciences, Azienda Ospedaliero-Universitaria Senese, Siena, Italy
2. Thoracic Surgery Unit, Department of Medical, Surgical and Neuro Sciences, University of Siena, Azienda Ospedaliero-Universitaria Senese, Siena, Italy
3. Diagnostic Imaging Unit, Department of Medical Sciences, University of Siena, Azienda Ospedaliero-Universitaria Senese, Siena, Italy
4. Respiratory Diseases Unit, Department of Medicine, Surgery and Neurosciences, University of Siena, Siena 53100, Italy
5. Thoracic Surgery Department, University and Hospital Trust—Ospedale Borgo Trento, Verona, Italy
6. Diagnostic Imaging Unit, Department of Medical, Surgical and Neuro Sciences and of Medical Sciences, University of Siena, Azienda Ospedaliero-Universitaria Senese, Siena, Italy

Background: Unilateral diaphragmatic paralysis (UDP) is a condition often overlooked, despite affecting the quality of life of affected patients, and therefore only a few of them undergo surgical diaphragmatic repair treatment. However, there is currently no standardized surgical approach. The aim of our study is to describe our robot-assisted (RATS) diaphragmatic plication technique, analyzing the perioperative outcomes.

Methodology: From 2016 to date, 9 patients have undergone RATS diaphragmatic plication at the Thoracic Surgery of Siena. All patients underwent pre- and post-operative assessment of respiratory function using global spirometry and alveolar-capillary diffusion testing (DLCO), as well as radiological evaluation using chest X-ray, chest CT scan and/or dynamic magnetic resonance imaging. The degree of pre- and post-operative dyspnoea was also evaluated for each patient using the mMRC (Medical Research Council) scale. The surgical technique involves a triple thoracoscopic access, with an anterior access in the 4th intercostal space and two accesses in the 6th intercostal space; the Robot is then reverse docked, with the arms directed towards the hemidiaphragm. The hemidiaphragm is folded onto itself using two or more continuous sutures in an anteroposterior and lateromedial direction towards the central tendon of the diaphragm (“fan suture”). The first two sutures are then sutured together with an additional continuous suture, only after removing CO2 to prevent further diaphragm ascent.

Results: Between January 2016 and March 2024, 9 patients aged 49–76 years underwent diaphragmatic plication in RATS, of which 6 had idiopathic, 2 post-traumatic, and 1 iatrogenic UDP. The average preoperative FEV1, FVC, and DLCO were 184.3 ± 59.0 mL, 215.0 ± 74.9 mL, and 6.7 ± 4.8 mL respectively. The average duration of the surgical procedure was 158 min; no intraoperative complications were recorded. Only one patient developed post-operative pulmonary thromboembolism 7 days after the surgery. The daily amount of pleural fluid was 233 ± 109 mL, with drainage removed after 2.5 days and discharge on the fourth postoperative day. The perceived pain according to the VAS scale was 2.6 on the first day and 1.3 at discharge. The average postoperative mMRC value was 0.9 ± 0.7 compared to 2.7 ± 0.7 preoperatively. A statistically significant difference was observed between the average radiological area of diaphragmatic relaxation calculated on preoperative and postoperative chest X-rays (*p* = 0.04).

Conclusions: RATS diaphragmatic plication is a valid approach for UDP repair; this minimally invasive technique allows for excellent control of postoperative pain, reducing dyspnoea and patient perception of it. Further comparative studies are needed to compare this technique with the VATS or open approach.

### 1.56. Abstract 56 (ePoster): Stabilization of Multiple Rib Fractures without Flail Chest Using Customizable Titanium Plates Fixed with Self-Tapping Screws

**Luciano Cialì Sposato ^1^, Alexandro Patirelis ^1^, Sebastiano Angelo Bastone ^1,2^, Matteo Russo ^3^, Cristiano Casciani ^1^, Chiara Combattelli ^1^, Kumar Karan ^1^ and Raul Randazzo ^1^ and Vincenzo Ambrogi ^1^**

1. Department of Thoracic Surgery, Tor Vergata University Polyclinic, Via Montpellier 1, Rome, Italy
2. Ph.D. Program in Applied Medical-Surgical Sciences, Department of Surgical Science, Tor Vergata University Polyclinic, Rome, Italy
3. Department of Industrial Engineering, University of Rome Tor Vergata, Via del Politecnico 1, Rome, Italy

Background: Rib fractures are a common injury, observed in 20–39% of patients with blunt chest trauma. Currently, there is no consensus on the ideal treatment for multiple rib fractures without flail chest. Options range from conservative treatment to surgical stabilization. This study aims at demonstrating the efficacy and safety of the stabilization of rib fractures by a novel type of customizable titanium plates.

Methodology: Since 2023, we have performed stabilization for multiple rib fractures in 5 patients with anterior rib arch fractures using the RibFix Blu^®^ Thoracic Fixation System (Zimmer Biomet, Warsaw, IN, USA), which consists of customizable titanium plates fixed with self-tapping screws. Indications for surgery included displaced fractures of at least 3 ribs in the anterior arch and uncontrolled pain. The procedure was performed within 72 h of the trauma. Data recorded were: pain at 24 and 48 h measured with the Visual Analog Scale, number of different analgesic drugs administrated (1, 2, or 3 medications), episodes of desaturation (SpO_2_ < 85%), morbidity rate, length of hospital stay. We also evaluated the extent of rib movement measured as angular range of motion around two spatial axes (roll and pitch) using the RespirHolter, a wearable monitoring system consisting in an inertial unit combined with an accelerometer and a gyroscope, developed by our center. We compared these results with those from 5 patients who previously received only conservative treatment.

Results: The treated group showed lower pain at 24 h (Median 6, Interquartile Range 4–7 vs. 6, 5.5–6.5; *p* = 0.69) and at 48 h (3, 2–4 vs. 6, 4–6.5; *p* = 0.032), inferior number of analgesic drugs (2, 1.5–2.5 vs. 3, 2–3; *p* = 0.22), fewer episodes of desaturation (0 vs. 4; *p* = 0.024), lesser morbidity rate (1 vs. 2; *p* = 0.024), shorter hospital stay (6, 5–6 days vs. 10, 8.5–14.5 days; *p* = 0.008). The RespirHolter showed a significant reduction in the range of motion measured in degrees (0.187°, 0.152°–0.198° vs. 0.227°, 0.215°–0.304°; *p* = 0.009). 

Conclusions: Despite the small sample size, our study demonstrated that stabilization significantly reduces pain, need for analgesic medication, episodes of desaturation, morbidity rate and hospital stay. The data obtained with the RespirHolter confirmed the effectiveness of stabilization compared to untreated patients.

### 1.57. Abstract 57 (ePoster): Practice of Oxygenation and Respiratory Support during Fiberoptic Bronchoscopy: The Oxy-FOB STUDY

**Isacco Curto ^1^ and Federico Longhini ^2^**

1. Terapia Intensiva Neurochirurgica, Ospedale Santa Chiara, Trento
2. Università degli Studi di Catanzaro “Magna Graecia”

Background: Fiberoptic bronchoscopy (FOB) is a diagnostic and sometimes therapeutic procedure commonly performed both in elective and emergency conditions for airway, lung parenchymal or mediastinal disorders. Plug removal, bleeding management and endobronchial foreign body removal represent the main therapeutic indications for FOB. FOB is also utilized for diagnostic purposes alone or in combination with bronchoalveolar lavage (BAL), brushing, bronchial and transbronchial lung biopsy, intrathoracic lymph node sampling by means of EBUS. 

In spite of a relevant number of available studies, most investigations consider physiological rather than clinical outcome variables with heterogeneous populations with respect to severity, type of procedure and supportive means. Because of these limitations, no guidelines are today available and the daily clinical practice varies among centers.

The primary aim is to describe the current practice of supports in patients undergoing FOB, stratified by baseline respiratory condition, co-morbidities, FOB procedure and hospital settings.

In addition, we aim to assess: the occurrence of adverse events (i.e., severe desaturation, need for procedure interruption, hypotensive or hypertensive events, new onset of cardiac arrhythmias or myocardial ischemia or electrocardiographic ST-alterations); the lowest oxygen saturation; maximum intraprocedural drop in oxygen saturation from baseline; need to increase inspired oxygen fraction (FiO_2_); the adopted sedation strategies; need for escalating respiratory support; need for ICU admission (only for patients not admitted in ICU); hospital length of stay; mortality.

Methodology: This is a multicentre, international, prospective, observational study on adult patients requiring any type of FOB. The study protocol is designed and it will be reported according to the Strengthening the Reporting of Observational Studies in Epidemiology (STROBE) Statement. For all involved centers, Ethics approval or a waiver is mandatory to start with enrolments. The study is currently involving 44 centers in Europe. In view of the observational nature of the study, a formal sample size calculation is not done, but a target sample of at least 10.000 patients is planned. The enrolment will be conducted over a first 4-week participation period over 2 months, and a second one (4 weeks) 6 months after the first, for each unit.

Statistical analysis will be performed by an independent expert statistician, in collaboration with the investigators. A database has been created and the statistical analysis of the first data is currently underway.

Conclusions: Data collection is still ongoing. The primary analysis of the study is promising. We are sure that the analysis of the data collection will contribute to the production of useful and effective guidelines for the correct management of oxygenation and respiratory support during fiberoptic bronchoscopy.

### 1.58. Abstract 58 (ePoster): Medical Thoracoscopy with or without Pleural Fluid: Different Diagnostic Yield? 

**Foltran Gabriele, Lanfranchi Filippo, Mancino Laura, Michieletto Lucio**

Respiratory Disease Unit, Department of Cardiac Thoracic and Vascular Scienses, Ospedale dell’Angelo, Venice, Italy.

Background: Medical thoracoscopy (MT) is a common, safe and minimally invasive endoscopic practice which is currently used to diagnose pleural effusion of unknown etiology [15] or to perform talc pleurodesis in malignant pleural fluid; according to recent evidence, medical thoracoscopy is a safe and effective technique also for the treatment of complicated parapneumonic fluid and empyema [16]. MT can be performed in patients with or without pleural fluid. The aim of this study is to compare diagnostic yield (DY) between these two subgroups in our center.

Methodology: From January 2021 to April 2024, we enrolled 157 patients with suspected malignant pleural fluid or suspected pleural thickening. Patients were subjected to chest CT scan; MT was performed in deep sedation plus local anesthesia. Thoracic ultrasound was used to evaluate patients before the procedure and to divide them in three subgroups: free pleural fluid, no pleural fluid or organized pleural fluid. DY was evaluated for each subgroup: chi-quadro score has been used to compare different DY.

Results: 157 patients were included (111 male, 46 female) and subjected to MT: 131 of these were diagnostic (83.4%). The first subgroup (free pleural fluid) collects 118 thoracoscopy: 105 of these were diagnostic (88.9%). The second subgroup (no pleural fluid) collects 20 thoracoscopy: 15 of these were diagnostic (75%). The third subgroup (organized pleural fluid) collects 19 thoracoscopy: 11 of these were diagnostic (57.8%).

DY was not significantly different between the first and the second subgroup (88.9% vs. 75%; chi-quadro 2.9481; *p*-value 0.086). Otherwise, DY was significantly different between the first and the third subgroup (88.9% vs. 57.8%; chi-quadro 12.1863; *p*-value 0.000481) (Table 10).

Conclusions: In our center MT is a safe and largely used procedure and its DY is in line with literature. According to this study, the absence of pleural fluid is not a limitation for DY: in fact, DY of MT without pleural fluid is not significantly different from DY of thoracoscopy with pleural fluid. Otherwise, DY is significantly different between thoracoscopy with free pleural fluid and thoracoscopy with organized pleural fluid. Further studies are needed to validate this hypothesis.

### 1.59. Abstract 59 (ePoster): Diagnostic and Staging Accuracy of Ebus-Tbna in Lung Cancer: Single-Center Case Series Collected at the Thoracic Endoscopy Service of the Campus Bio-Medico University Hospital of Rome from 2019–2023

**Davide Onofrio Fontana, Evelyn Di Matteo, Panaiotis Finamore, Francesca Di Nunzio and Simone Scarlata**

Campus Bio-Medico University Hospital Foundation of Rome, Santa Scolastica Hospital of Cassino

Background: To estimate the diagnostic accuracy of EBUS, its determinants, and the concordance of endoscopic and pathological staging.

Methodology: The case series from the Thoracic Endoscopy Service of the Campus Bio-Medico University Hospital of Rome from 2019–2023 was retrospectively analyzed. Patients with diagnoses other than lung cancer and those without indication for radical surgery were excluded. The final sample included 31 patients. Data were collected on the characteristics of the primary tumor and lymphadenopathies based on imaging exams (CT and/or PET-CT), data related to the endoscopic procedures performed, and staging of the *N* parameter. Diagnostic accuracy, sensitivity, and specificity of EBUS in identifying lymph node metastases in “N2” locations, according to the classification proposed by the AIOM 2021 guidelines, compared to lymph node metastases in “N1” locations or absence of lymph node metastases “N0”, were tested. The characteristics of the population stratified according to the correctness of the classification were compared. To establish the positive predictive value (PPV) and negative predictive value (NPV) based on the pre-test and post-test probability of disease, a statistical algorithm applying Bayesian analysis for the probability of mediastinal involvement in non-small cell lung cancer estimation was used (M.E.S.S.i.a. software, Available online: messiaproject.com).

Results: The adequacy of the sample collection was 81%, consistent with literature data. Lymph node size was the only significant determinant of the adequacy of transbronchial needle aspiration (TBNA). EBUS sensitivity and specificity were 50% and 96%, respectively, in line with literature data. The overall accuracy of endoscopic staging was 87%, higher than reported by several studies. EBUS predictive values varied based on the pre-test probability of disease, particularly showing an increase in NPV when the pre-test probability was low. Considering the aggregate pre-test probability given by CT + PET-CT, the NPV reached 89%. According to the M.E.S.S.i.a. algorithm, the threshold of prior probability to address the patient to radical surgery without further invasive tests is <8%. In our study group, patients with a residual lymph nodal involvement probability >8% who underwent surgery (*n* = 18) showed concordance of staging in almost all cases, except for two cases where EBUS was falsely negative for N2 (11.1%).

Conclusions: EBUS is highly sensitive and specific, crucial for staging the “*N*” parameter with very high accuracy, capable of avoiding more invasive approaches.

### 1.60. Abstract 60 (ePoster): Start-Up of Ebus Procedures in Non-Teaching Hospital

**Giorgio Fumagalli ^1^, Mariarosaria Calvello ^1^, Marino De Rosa ^1^, Linda Tagliaboschi ^1^, Roberto Carlucci ^2^, Stefano Nardoni ^3^ and Luca Triolo ^1^**

1. Lung Unit, S. Filippo Neri Hospital, ASLROMA1, Rome
2. Intensive Care Unit, S. Filippo Neri Hospital, ASLROMA1, Rome
3. Pathology Unit, S. Filippo Neri Hospital, ASLROMA1, Rome

Background: Endobronchial Ultrasound (EBUS) is a minimally invasive bronchoscopic technique for diagnosis of lung and mediastinal diseases. The learning curve associated with EBUS is an essential consideration for both new practitioners and experienced pulmonologists transitioning to this advanced technique. Studies have demonstrated that a minimum number of procedures, typically ranging from 20 to 50, are necessary to achieve competency in EBUS, improve efficacy and diagnostic yield. 

Methodology: We describe the start-up of EBUS procedures in a tertiary non-teaching hospital. In the first 6 months, we evaluated 25 patients with malignant and non-malignant pathologies: 3 patients were excluded due to unsuitability for general anaesthesia. 19 patients underwent EBUS. All procedures were performed with deep sedation, mechanical ventilation via laryngeal mask, and overseen by experienced anaesthesiologists. Each session also included 2 pneumologists, 5 nurses, and a pathologist for Rapid On-Site Evaluation (ROSE). The team was assembled with redundancy to ensure safety and enhance learning, nevertheless significant organizational commitment. 

Results: The duration of EBUS procedures was longer for non-malignant diseases compared to malignant cases (88 min vs. 81 min), higher than in reference centers but decreasing with experience. Procedure times increased progressively with the number of sampled lymph node stations: primary lesion, 1 or 2 lymph node stations took 48, 80, and 104 min respectively. Between 3 to 9 passes were made for each target, with higher instances occurring when the pathologist was absent (8 passes) and in repeated EBUS for unconfirmed hematologic malignancies (9 passes). Complete agreement was achieved between ROSE findings and definitive cytologic and histologic results in malignant pathologies, but not consistently in non-malignant and onco-haematologic diseases. No complications were observed within the first 180 min post-procedure period and in the 7-day after. None of the patients required ICU recovery or multiparametric monitoring after procedure. 

Conclusions: In conclusion, mastering the EBUS procedure is a dynamic process that emphasizes the importance of ongoing education and practical experience. The commitment to overcoming the initial learning challenges is rewarded with the ability to provide patients with precise, minimally invasive diagnostic and therapeutic interventions. Improvement in skills is necessary to reduce procedure times and enhance diagnostic yield, particularly for diseases other than lung cancer. 

### 1.61. Abstract 61 (ePoster): Chest Wall Reconstruction: Our Experience with a New Titanium Mesh

**Eleonora La Rocca, Federica Mellone, Mattia Manitto, Giacomo Leoncini Antonella Alloisio, Ludovica Balsamo, Lucia Morelli, Luca Novello, Maria Teresa Piras and Gian Luca Pariscenti**

U.O.C. Chirurgia Toracica, IRCCS Ospedale Policlinico San Martino, Genova

Background: Chest wall reconstruction is intrinsically problematical because of curve ribs shape and the need to accommodate rib cage movements during breathing. Multiple devices have been developed to remodel chest wall in traumatic lesions, after chronic infective or inflammatory diseases or after demolition for oncological purpose. 

Methodology: We report our experience in chest wall reconstruction by using a peculiar titanium mesh projected by alternating triangular and round micro-holes. This feature gives non-deformable, resistant, elastic and chest-accommodating biomechanics. It is slim (0.4–0.6 mm), trimmable by scissors, pliable and useful to repair considerable substance loss. It can be fixed by non-absorbable sutures or bicortical screws, checkable from the inner side with thoracoscope.

Results: First patient exhibited right hemithorax pain and multiple cutaneous fistulas. After CT scan, a suspect chronic osteomyelitis of 7th right rib was diagnosed, confirmed by bone biopsy. We performed removal of 7th right rib and prosthetic reconstruction with titanium mesh. 

Second patient exhibited Poland Syndrome with agenesis of the right anterior chest wall. We reconstructed the wall defect with titanium mesh, bovine pericardium and breast prosthesis. 

Third patient presented with a mass of left upper lobe infiltrating chest wall. He underwent open left upper lobectomy en-bloc with wedge resection of the apical inferior segment, posterior portion of 4th, 5th and 6th rib, parietal pleura and intercostal muscles. The wall defect was rebuilt with titanium mesh covered in bovine pericardium sheet to minimize friction with superficial layers. 

Fourth patient had a post-traumatic incidental diagnosis of synovial sarcoma of left chest wall. The patient underwent resection of the neoplasm en-bloc with 4th and 5th left rib and adjacent intercostal muscles. Wall defect was corrected with titanium mesh. 

Fifth patient was diagnosed with right posterior chest wall sarcoma, involving D1–D6 vertebrae, without infiltration of medullary canal. Surgery was conducted together with neurosurgeons; resection of D1 to D6 posterior laminae, spinous and transverse processes along with right thoracectomy from 2nd to 6th rib were performed, with following repair of the relevant wall defect with titanium mesh.

No one resulted with complications due to the mesh. No chronic pain, infection or loss of pulmonary function was detected. 

Conclusions: In our preliminary experience, titanium mesh appears to be safe and easy to apply in chest-wall reconstructions. It gives strength and flexibility to the rib cage at the same time, perfectly fitting physiological breathing movements.

### 1.62. Abstract 62 (ePoster): Competences in Linear Ebus Using Franseen Needle Tip: The (Almost) Certainty of the Diagnosis

**Filippo Lanfranchi, Laura Mancino, Gabriele Foltran and Lucio Michieletto**

Respiratory Disease Unit, Department of Cardiac Thoracic and Vascular Sciences, Ospedale dell’Angelo, Venice, Italy

Background: EBUS-TBNA and EUS-B-FNA is a safe and minimally invasive procedure to evaluate hilar and mediastinal lymph nodes (LN). Franseen needle provides a transbronchial needle biopsy (TBNB) or a fine needle biopsy (FNB) if linear EBUS (L-EBUS) is inserted into esophagus (EUS-B). Various TBNB sized needles are available. In literature, diagnostic yield (DY) and sample adequacy (SA) between TBNB needle size are still debated. In this study we reported the DY of TBNB/FNB needles dividing DY per year, in order to evaluate if expertise and skills can improve competences and consequently the DY.

Methodology: We retrospectively evaluated 283 TBNB procedures (L-EBUS and EUS-B) from January 2021 to December 2022. EBUS-TBNB/EUS-B-FNB was performed with 22 G or 25 G needle size (Acquire pulmonary, Boston Scientific Co., Natick, MA, USA). (Figure 10).

DY and SA for predictive markers were evaluated, and the results were divided in two groups: 2021 and 2022 group. DY and needle size were recorded. 

DY was defined as the number of sample-positive patients plus sample-negative patients (confirmed by consequent clinical-radiological follow-up), divided by the total cohort size.

Every procedure was performed by expert bronchoscopist.

Results: In 2021, 90 TBNB procedures were performed. 22 G needle was used in 53 procedures (59%). Diagnosis was obtained in 46/53 patients, reaching a DY of 86.7% using 22 G needle. 25 G was used in 37 patients, with an overall DY of 34/37 (91.8%) using 25 G needles (Figure 11).

In 2022, 180 TBNB procedures were performed, and 13 EUS-B-FNB procedures were made.

22 G needle was used in 107 procedures (59%). Diagnosis was obtained in 103/107 patients, with a 96.2% DY with 22 G needle. 25 G was used in 73 patients, with an overall DY of 67/73 (=91.7%) with 25 G needles. Moreover, 13 EUS-B-FNB procedures produced 13 diagnostic samples (DY 100%).

Conclusions: Franseen needle tip allows to perform TBNB/FNB samples with a very high DY.

Training can further increase their performance: in fact, different sampling techniques can be used and improved, increasing bronchoscopist’s competence and consequently the DY of these needles. Further studies are needed to evaluate if a EBUS-TBNB training programme can raise up DY in every endoscopy suite.

### 1.63. Abstract 63 (ePoster): Evaluation of Artificial Intelligence Answers to Frequently Asked Question for Lung Cancer Surgery

**Stefano Lovadina ^1^, Alice Ravasin ^3^, Davide Drigo ^2^, Alessia Arbore ^1^, Simonetta Masaro ^1^, Elisabetta Benci ^1^, Enrico Arbore ^1^, Marina Troian ^1^ and Maurizio Cortale ^1^**

1. Thoracic Surgery Unit, Cattinara University Hospital, Trieste, Italy
2. Department of General Surgery, Cattinara University Hospital, Trieste, Italy
3. Thoracic Surgery Unit, Careggi University Hospital, Florence, Italy

Background: Nowadays, patients have numerous digital resources that allow quick and easy access to information regarding health issues. In this context, artificial intelligence (AI) based chatbots and machine learning are rapidly gaining popularity, and patients may turn to this technology to ask questions about surgical procedures, preoperative assessments, and postoperative aspects. Therefore, we sought to determine if ChatGPT-3.5 (free version of chatbot developed by OpenAI^®^) could properly answer to some of the most frequently asked questions posed by patients regarding lung cancer surgery.

Methodology: Sixteen common questions about lung cancer surgery were asked to the chatbot during a conversation, without any follow-up questions or repetitions. Each response was subsequently analyzed to verify its accuracy using an evidence-based approach. The answers were evaluated by a group of surgical specialists with at least five years of working experience. The quality of AI answers was assessed using a four-point Likert scale (i.e., “strongly agree, satisfactory2”, “agree, requires minimal clarification”, “disagree, requires moderate clarification”, “strongly disagree, requires substantial clarification”).

Results: The answers provided by the chatbot were judged to be overall satisfactory, generally impartial, and evidence-based. Moreover, the language appeared to be understandable to most patients.

Conclusions: AI-based chatbots can effectively provide evidence-based answers to the most frequently asked questions that patients may have before undergoing lung cancer surgery. User-friendly and potentially free of charge, AI excels in delivering information clearly and understandably across different languages, possibly restructuring the preoperative process and alleviating the burden on surgeons by saving valuable time that would otherwise be spent on explanations. This resource can serve as a valuable adjunctive tool for patient education in the preoperative phase; however, concern remains regarding the holistic care of patients as AI may lack empathy and non-verbal cues essential for fostering emotional reassurance and building a strong doctor-patient relationship. These elements, such as facial expressions and physical contact, play a crucial role in patient-centered care and are irreplaceable by technology.

### 1.64. Abstract 64 (ePoster): Dual-Port Fully Robotic-Assisted Thoracic Surgery for Major Pulmonary Resection: Initial Experience

**Elisa Meacci, Dania Nachira, Khrystyna Kuzmych, Maria Teresa Congedo and Stefano Margaritora**

Fondazione Policlinico Universitario A.Gemelli I.R.C.C.S- Università Cattolica del Sacro Cuore di Roma- Roma

Background: This study aimed to evaluate the intraoperative and short-term outcomes of fully dual-port robotic-assisted thoracic surgery (F-DRATS) for major lung resection.

Methodology: A retrospective analysis was conducted to evaluate the clinical data and short-term outcomes of patients who underwent F-DRATS for major lung resection. Based on our experience with uniportal video-assisted thoracic surgery (VATS) and multiportal robotic-assisted thoracic surgery (RATS), the F-DRATS approach was conceptualized and initiated in November 2023. Between November 2023 and May 2024, 18 patients underwent F-DRATS for major lung resection. F-DRATS is defined as robotic thoracic surgery performed using two intercostal incisions (Figure 12), without rib spreading, utilizing the robotic camera, robotic dissecting instruments, and exclusively robotic staplers.

Results: Major lung resections (9 lobectomies, 9 segmentectomies) were successfully performed in all 18 patients using a biportal robotic system, with no conversions to thoracotomy during the operations. The cohort included 11 males and 7 females, aged 25 to 82 years (mean age 67.9 ± 15.5 years). The mean total operation time was 160.6 ± 83.9 min, console time of 105.4 ± 72.2 min, and chest closing time of 11 ± 3.3 min. The average blood loss was 62.9 ± 51.5 mL. The number of lymph nodes dissected ranged from 0 to 22 (mean 4.6 ± 6.4). The mean chest tube duration was 3.5 ± 2.3 days, with first-day post-surgery drainage averaging 108.9 ± 84.7 mL and total drainage averaging 411.1 ± 260.9 mL. The average hospital stay was 3 ± 1.1 days. The median visual analogue scores for pain were 4 (range 3–7) at 24 h and 3 (range 2–5) at 72 h post-surgery. Post-operative complications occurred in 3 cases (16.7%), but there were no serious complications or deaths within 30 days post-surgery. The final pathological diagnoses included 12 malignancies NSCLC and 6 lung metastases.

Conclusions: The biportal robot-assisted major lung resection was found to be a safe and effective treatment for lung cancer.

### 1.65. Abstract 65 (ePoster): The Role of Pulmonary Metastasectomy in Oligometastatic Melanoma Patients after the Introduction of Immuno and Targeted Therapies: Results and Prognostic Factors from a Single Center Experience: Introduction of Immuno and Targeted Therapies: Results and Prognostic Factors from a Single Center Experience

**Elisa Meacci ^1^, Dania Nachira ^1^, Khrystyna Kuzmych ^1^, Maria Teresa Congedo ^1^, Marco Chiappetta ^1^, Alessandro Di Stefani ^2^, Laura Del Regno ^2^, Ketty Peris ^2^, Elizabeth Katherine Anna Triumbari ^3^, Giovanni Schinzari ^4^, Ernesto Rossi ^4^, Leonardo Petracca Ciavarella ^1^, Maria Letizia Vita ^1^, Edoardo Zanfrini ^5^, Diomira Tabacco ^1^, Giuseppe Calabrese ^1^, Jessica Evangelista ^1^ and Stefano Margaritora ^1^**

1. Department of General Thoracic Surgery, Fondazione Policlinico Universitario “A. Gemelli”, IRCCS, Università Cattolica del Sacro Cuore, Rome, Italy
2. Dermatology Unit, Fondazione Policlinico Universitario, A. Gemelli IRCCS, Università Cattolica del Sacro Cuore, Rome, Italy
3. Nuclear Medicine Unit, G-STeP Radiopharmacy Research Core Facility, Department of Radiology, Radiotherapy and Hematology, Fondazione Policlinico Universitario A. Gemelli, IRCCS, Rome, Italy
4. Medical Oncology Unit, Fondazione Policlinico Universitario A. Gemelli IRCCS, Università Cattolica del Sacro Cuore, Rome, Italy
5. Service of Thoracic Surgery, University Hospital of Lausanne, Lausanne, Switzerland

Background: The role of pulmonary metastasectomy (PM) and prognostic factors influencing survival in oligometastatic patients affected by malignant melanoma (MM) in the era of Effective-Systemic-Therapies (ESTs) is still not completely clear. We aimed to investigate the efficacy of PM and determine prognostic factors affecting survival to improve patient selection.

Methodology: Clinical, surgical, and oncological data of 60 patients who underwent PM between June 2008 and January 2022 were collected.

Results: All patients underwent surgical resection of the primary melanoma before PM. Nine (15%) patients had a synchronous lung metastasis. A wedge resection was performed in 95% of cases to radically remove the pulmonary localizations, while in the remaining cases, an anatomical resection was necessary. No major postoperative complications or postoperative mortality were recorded. Ten patients (16%) experienced minor complications. Mean in-hospital stay was 4.51 ± 2.4 days. Following lung surgery, 92% of patients received adjuvant treatments (48.2% immunotherapy and 43.8% targeted therapy). Over a mean follow-up period of 107.9 ± 81.5 months, 24 (40%) patients died due to melanoma disease, and 3 (5%) to other causes. Twenty-two patients (36.6%) experienced disease recurrence, and 8 (13.3%) developed extrapulmonary metastases after PM. The CSS (time between first melanoma resection or PM and death from cancer) from melanoma resection was 86% at 5 years, 72% at 10, 53% at 15, 45% at 20, and 2% at 25, respectively (Figure 13). 

The 5- and 10-year CSS from PM were 73% and 29%, respectively. Prognostic factors negatively impacting CSS from PM in multivariable analysis were melanoma vertical growth (*p* = 0.018), synchronous lung metastasis (*p* < 0.001), previous metastatic sites other than lungs (*p* < 0.001), and Disease-Free-Interval (DFI) < 24 months (*p* = 0.007) (Figure 14).

Conclusions: PM confirms its crucial role in stage IV oligometastatic melanoma with resectable pulmonary metastases. Patients with long DFI, radialgrowth melanoma phase, metachronous, and no site of metastatization other than lung appears the best candidates for PM. 

### 1.66. Abstract 66 (ePoster): Advances in Endoscopic Management of Endobronchial Carcinoid

**Gaetana Messina, Giuseppe Vicario, Riccardo Vinciguerra, Davide Pica, Francesca Capasso, Vicenzo Di Filippo, Beatrice Leonardi, Francesco Leone, Maria Antonietta Puca, Maria Marvulli, Noemi Giorgiana, Alessia Caputo, Giovanni Natale, Giovanni Vicidomini and Alfonso Fiorelli**

UOC Chirurgia Toracica Università degli Studi della Campania ‘Luigi Vanvitelli’ Napoli

Background: Bronchial carcinoid (BC) tumors represent between 1% and 5% of all lung cancers and about 20–30% of carcinoid tumors; they are classified into two groups: typical and atypical bronchial carcinoids. The aim of the present study was to review the results of endoscopic treatments as an alternative to surgical treatment in selected patients. 

Methodology: The present study was a retrospective and multicentric study, in which all data were reviewed for patients with BC in the central airways, referred to the Thoracic Surgery Units of Luigi Vanvitelli University of Naples and Sant’Andrea Hospital in Rome between October 2013 and December 2023 Overall, 35 patients, 13 of whom were female, were included in the study (median age, 53 years; range, 29–75 years). All patients underwent rigid bronchoscopy combined with flexible bronchoscopy. Tumor clearance was mostly performed by use of Argon Plasma Coagulation or Thulep Laser, mechanical debridement and excision with the use of forceps and aspirator through the working channel of the 8.5 mm-sized rigid bronchoscope. There were no complications during the treatment. 

Results: Endobronchial treatment provided complete tumor eradication in all patients; two patients had controlled bleeding complications; however, bleeding was well controlled without patient desaturation. We found two recurrences in the left and right main bronchus, in patients with atypical carcinoma during fiberoptic bronchoscopy follow-up. Only one patient died of renal failure during the follow-up period. At the first analysis, there were no significant differences between the patients receiving endobronchial treatment and patients receiving surgical treatment in the present study (*p*-value > 0.05—it means statistically insignificant). 

Conclusions: Endobronchial treatment is a valid and effective alternative for patients with BC unsuitable for surgery.

### 1.67. Abstract 67 (ePoster): Endoscopic Treatment of Tracheoesophageal Fistula (Tef): Personal Experience

**Flavio Marco Mirabelli ^1^, Emma Repaci ^1^, Ilaria Menichini ^2^, Paolo Palange ^1^ and Giovanni Galluccio ^2^**

1. Department of Public Health and Infectious Diseases, Sapienza University of Rome, Italy. Pulmonology, Policlinico Umberto I Hospital Rome, Italy
2. Thoracic Endoscopy and Interventional Pulmonology Center, Regina Apostolorum Hospital, Albano Laziale, Italy

Background: Tracheoesophageal fistula (TEF) is an abnormal communication between the gastrointestinal tract and airways. It is most frequently congenital, often associated with esophageal atresia, but can also be acquired due to tumors, trauma, or inflammatory diseases. The gold standard for TEF treatment is surgery, but it is not feasible for all patients. This study explores an endoscopic technique for TEF repair as an alternative to surgery.

Methodology: From 2015 to 2022, 10 patients aged 18 to 81 with non-neoplastic TEF were treated using an endoscopic approach. The technique involved rigid bronchoscopy under deep sedation, using a tracheoscope to expose and suture the fistulas with resorbable or non-resorbable stitches. Follow-up examinations were conducted at 1 month and at regular intervals up to 1 year using a flexible bronchoscope.

Results: Out of 10 patients, 4 achieved complete healing at 1 year post-procedure. Five patients had a reopening of the fistula within 1 to 3 months, requiring additional procedures, and one patient experienced a complete reopening after 1 month. No complications occurred during or after the procedures. Two patients died during follow-up due to unrelated causes.

Conclusions: TEFs are severe complications often seen in patients with prolonged intubation or tracheostomy, typically leading to respiratory infections and high mortality if untreated. Although surgery is the preferred treatment, it is not suitable for all patients due to its complexity. Endoscopic techniques, especially the placement of esophageal stents, provide palliative care but are associated with various complications. The endoscopic suturing technique presented in this study offers a viable alternative, showing promising results in 40% of patients without relapses. This method allows for quick recovery and can be a critical option for patients not eligible for surgery. Further refinement and larger patient studies are needed to enhance the technique’s effectiveness and reliability.

### 1.68. Abstract 68 (ePoster): Post-Intubation Tracheal Lacerations: Diagnosis and Treatment in Operative Endoscopy: Personal Experience

**Flavio Marco Mirabelli ^1^, Emma Repaci ^1^, Ilaria Menichini ^2^, Paolo Palange ^1^ and Giovanni Galluccio ^2^**

1. Department of Public Health and Infectious Diseases, Sapienza University of Rome, Italy. Pulmonology, Policlinico Umberto I Hospital Rome, Italy
2. Thoracic Endoscopy and Interventional Pulmonology Center, Regina Apostolorum Hospital, Albano Laziale, Italy

Background: Post-intubation tracheal tear (PITT) is a rare but serious complication of tracheal intubation, potentially fatal, necessitating prompt identification, management, and treatment. Clinical manifestations vary by damage severity, commonly including subcutaneous and mediastinal emphysema, and pneumothorax. Diagnoses primarily rely on fiberoptic bronchoscopy and chest CT. Historically, early surgical intervention of the trachea was the mainstay of treatment, but there are increasing evidences that support a non-surgical conservative treatment approach in selected patients without mediastinitis/pneumomediastinum and without esophageal injuries.

Methodology: This study examined patients with PITT from 2003 to 2021 at San Camillo Forlanini hospital. Diagnoses were confirmed via bronchoscopy and chest CT. Lesions were classified by depth and complications into four grades, with Grade I, II, and IIIa were managed conservatively and Grade IIIb was referred for surgical intervention.

Results: Among 81 patients (average age 59.8 years, 13 males, 68 females), common symptoms included cough, dyspnoea, and hemoptysis. Lesions averaged 3.1 cm, mostly in the mid-tracheal segment. There were 13 Grade I, 53 Grade II, 5 Grade IIIa, and 10 Grade IIIb lesions. Conservative treatment was successful in all 71 patients with PITT grade I to IIIa, achieving complete healing within 28 days. Grade IIIb lesions necessitated surgery.

Conclusions: While surgical treatment was traditionally preferred, recent evidence supports conservative management for select cases, particularly in experienced centers. The proposed classification system, based on depth and complications of PITT aids in standardizing treatment approaches. The conservative management in PITT grade I to IIIa showed a good succession rate (100%) supporting the indication for conservative treatment in not-complicated PITT. The COVID-19 pandemic likely increased PITT incidence due to higher intubation rates and operational challenges. PITT requires rapid diagnosis and tailored treatment. Conservative management is effective for Grades I, II, and IIIa lesions in specialized centers, while Grade IIIb lesions require surgical repair. The pandemic has potentially contributed to a rise in PITT cases.

### 1.69. Abstract 69 (ePoster): Conservative Treatment of Iatrogenic Tracheal Lesion with Self-Expandable Tracheal Stent: A Monocentric Case Series

**Diletta Mongiello ^1,2^, Maria Teresa Bevilacqua ^1,2^, Francesca Cialdella ^1^, Nicoletta Pia Ardò ^1^, Roberto De Bellis ^1^, Francesco Lastaria ^1^, Sara Tango ^1^, Giulia Pacella ^1^, Rita Daniela Marasco ^1^, Leonardo Fino ^1^, Francesco Sollitto ^1^ and Domenico Loizzi ^1^**

1. Università di Foggia, Azienda ospedaliero-universitaria “Policlinico Riuniti” Foggia, Struttura Complessa di Chirurgia Toracica Universitaria, Foggia-Italy
2. Università degli Studi di Bari, Azienda ospedaliero-universitaria “Policlinico Consorziale” Bari-Italy

Background: Iatrogenic tracheal rupture (ITR) represents a rare but life-threatening condition requiring timely diagnosis and appropriate treatment. The most frequent cause of iatrogenic tracheobronchial rupture is tracheal intubation. Surgical repair has always been the gold standard approach. Conservative treatment in selected cases is gaining an emerging role. We report our experience in the treatment of iatrogenic tracheal tears in 4 patients conservatively and effectively treated with self-expandable covered metal stents. 

Methodology: We retrospectively analyzed the cases of iatrogenic tracheal injury treated in our center from 2017 to 2024. After clinical stabilization, tracheal injuries were treated conservatively with covered metallic self-expanding stents placed under rigid bronchoscopy and general anesthesia. We wanted to evaluate: the efficacy and safety of the treatment, intraoperative mortality rate, stent dislocation rate, procedure time, post-operative complications, and time from stent placement to stent removal.

Results: From 2017 to 2023, 4 patients (2 male, mean age 50.5 ± 20.2) came to our attention with iatrogenic tracheobronchial rupture due to tracheal intubation (2 cases during emergency surgery and 2 cases in elective surgery). In 2 cases, there was a full-thickness, longitudinal, quite severe injury of the pars membranacea (type IIIa of Cardillo’s classification). In all cases, a metallic covered self-expanding stent was positioned (in 3 cases Y-shaped stent, and in 1 case, a straight stent). In all cases, a tracheostomy was performed, and the distal part of the cuffed cannula was placed within the lumen of the stent. In all cases, an immediate improvement in the clinical condition of the patients was observed with weaning from ventilation within 24 h of the procedure.

No intraoperative death or stent-associated complications were registered. The mean time of the procedure was 55 ± 26.45 min. No stent dislocation was observed. After the procedure, the mean length of stay in the intensive care unit was 6.75 ± 8 days. The mean time from stent placement to stent removal was 43 ± 5 days. In all cases, endoscopic control showed the complete healing of the lesion. 

Conclusions: The use of a tracheal stent is an effective treatment of tracheal tears. In our experience, for injuries of the pars membranacea near the carena, the placement of a stent can be considered a valid and well-tolerated treatment alternative to surgery, especially the y-stent for its greater stability. Tracheal injuries should be referred to experienced centers.

### 1.70. Abstract 70 (ePoster): Performance of Genexpert Mtb/rif on Pleural Biopsy

**Lamya Chrif Morand ^1^, Imane Saidi ^2^ and Mehdi Bounou ^3^**

1. Private Office, Kénitra, Morocco
2. UM6P Hospital, Marrakech, Morocco
3. La Province Laboratory, Kénitra, Morocco

Background: The diagnosis of pleural tuberculosis is often a real challenge.

The yield of mycobacteria appears to be very low in pleural effusions because it is a pauci-bacillary disease.

The aim of our study is to evaluate the performance of GeneXpert for the diagnosis of tuberculosis on pleural biopsy fragments.

Methodology: Prospective study, carried out between 1 November 2022 and 30 April 2024. The study enrolled 70 patients with exudative lymphocytic pleural effusion. All patients underwent an ultrasound-guided pleural biopsy. The biopsy fragments were sent for pathology and gene amplification using the Xpert MTB/RIF technique (Cepheid, Sunnyvale, CA, USA).

For data analysis, the presence of granuloma on the biopsy was considered the gold standard of diagnosis for tuberculosis.

The sensitivity, specificity, PPV and NPV of GeneXpert MTB RIF on pleural biopsy fragments in the diagnosis of pleural tuberculosis were calculated using the presence of granuloma in the histopathology of pleural biopsy specimens as the gold standard.

Results: Among the 70 patients, 46 were men (65.7%). The average age was 38 years old. Among these 70 patients, 53 (75.7%) had histopathology confirming tuberculosis. The GeneXpert MTB RIF were positive in 26 cases (37.1%). Among the patients with positive histology, 24 (45.3%) had positive GeneXpert.

The sensitivity and specificity of GeneXpert were 61.5% and 93.5%, respectively. The PPV and NPV were 92.3% and 65.9%, respectively.

Conclusions: Gene amplification on a pleural biopsy fragment is a rapid diagnostic method that can be used as first-line diagnostic to save time and cost despite its low sensitivity because its PPV is very high. In the case of a negative result, the pathological analysis will complete the diagnosis. The rapid result it offers makes this diagnosis very useful in situations where a rapid diagnosis is important.

### 1.71. Abstract 71 (ePoster): Pseudo-Relapse of Lung Cancer Secondary to Post Surgical Mediastinal Foreign Body Reaction—A Case Series

**Filippo Natali ^1^, Damaride Micaela Bitetto ^2,3^, Gian Piero Bandelli ^1^, Marco Ferrari ^1^, Thomas Galasso ^1^, Martina Ferioli ^1^, Tommaso Abbate ^1^, Francesca Ambrosi ^4,5^, Nazarena Nannini ^3^, Pietro Bertoglio ^6^ and Piero Candoli ^1^**

1. Interventional Pulmonology Unit, IRCCS Azienda Ospedaliero-Universitaria di Bologna, Italy
2. IRCCS Azienda Ospedaliero-Universitaria di Bologna, University Hospital Sant’Orsola-Malpighi—Respiratory and Critical Care Unit, Bologna, Italy
3. Alma Mater Studiorum, Department of Medical and Surgical Sciences (DIMEC), University of Bologna, Italy
4. Pathology Unit, Maggiore Hospital AUSL, Bologna, Italy
5. Department of experimental, diagnostic and specialist medicine (DIMES), University of Bologna, Bologna, Italy
6. Department of Thoracic Surgery, IRCCS Azienda Ospedaliero-Universitaria di Bologna, Italy

Background: Hemostatic materials are used to manage intraoperative bleeding and are often intentionally left in place in the surgical field. Among these agents, oxidized cellulose is a resorbable material widely employed across various medical specialties, including thoracic surgery. Specifically, chest surgeons may find it beneficial during mediastinal lymphadenectomy in the context of lung cancer treatment.

However, their incomplete absorption can result in a foreign body reaction that mimics non-homogeneous lymph node enlargement, often positive on fluorodeoxyglucose positron emission tomography (FDG-PET), which can be confused for cancer relapse or metastasis. 

Methodology: During 2023 six patients were referred to our Interventional Pulmonology Unit for the evaluation of suspicious mediastinal enlargements following lymphadenectomy, which resulted in FDG avidity on PET scans (SUVmax ranged from 2.7 to 8.1—Figure 15A,B). 

All of them underwent lobectomy or wedge resection via robotic surgery for lung cancer at the Thoracic Surgery Department of Bologna within the past year. 

Results: The average age was around 65 years and all were former or current smokers. All patients were asymptomatic. The suspected lymph nodes were sampled via Endobronchial Ultrasound-Transbronchial Needle Aspiration (EBUS-TBNA). Both rapid on-site evaluation (ROSE) and the final histological exam revealed the presence of amorphous, exogenous, fibrillar material in a background of chronic inflammation, with lymphocytes and macrophages (Figure 15C,D).

The presence of neoplastic cells was ruled out.

The pattern was consistent with a foreign body inflammatory reaction resulting from previous robotic surgery. Moreover, dimensional and metabolic features remained stable at the subsequent radiological follow-up.

After reviewing the cases with the Thoracic Surgeons, we can conclude that the fibrillar substance is compatible with oxidized cellulose material utilized during surgical lymphadenectomy to ensure hemostatic control. 

Conclusions: Resorbable, oxidized cellulose materials are valuable for achieving bleeding control during thoracic surgery. Unfortunately, their incomplete resorption may trigger a persistent foreign body inflammatory reaction, which leads physicians to suspect lung cancer relapse. EBUS-TBNA is a reliable tool to exclude malignancy. The need for endoscopic investigations and the persistence of such findings over time cause both stress for the patients and difficulty in interpretation during the oncological follow up.

### 1.72. Abstract 72 (ePoster): The Lung Cancer Pathway as a Model of Excellence at Policlinico Universitario Agostino Gemelli

**Adriana Nocera, Antonio Giulio Napolitano, Claudia Leoni, Leonardo Petracca-Ciavarella, Maria Letizia Vita, Maria Teresa Congedo and Stefano Margaritora**

Dipartimento di Chirurgia Toracica, Fondazione Policlinico Universitario Agostino Gemelli, IRCCS, 00168, Roma

Background: Clinical Pathways (CPs) play a significant role in the study and treatment of neoplasms. The “Lung Cancer Pathway” (LCP) has been present at Policlinico Gemelli for over 15 years, aiming to provide patients with lung neoplasms rapid and comprehensive access to diagnosis and treatment. This study aims to examine the efficiency of the LCP in optimizing the process of study and pre-hospitalization for surgical patients, with particular attention to the implementation of a multidisciplinary approach and the acceleration of waiting times, especially for high-complexity patients.

Methodology: The LCP involves access to a Day-Hospital setting where hemato-chemical tests, ECG, spirometry, blood gas analysis, pulmonary, cardiological, and anesthesiological assessments are conducted to stratify the patient’s surgical risk and establish the correct therapeutic pathway (Table 11).

Data were collected from 1 January 2018 to 1 April 2024 concerning the results of LCP test performance. Patients unfit for surgery were directed to oncological care. Special attention was paid to the analysis of high-complexity patients who required different specialist evaluations to obtain surgical clearance.

Results: Over the last 76 months (January 2018—April 2024), the LCP has managed 2377 patients. There has been an increase from 318 patients in 2018 to 482 patients in 2023. Even the data from the first quarter of 2024 (January–April) show a steady growth compared to the first quarter of previous years (Figure 16). 

The percentage of ineligible patients for surgery remains stable over time (approximately 5%). Despite the variety of specialist visits provided during the LCP, even high-complexity patients do not undergo, on average, more than two Day-Hospital accesses. These results indicate that the LCP in recent years has managed to include a greater number of patients without compromising the quality of care provided. The multidisciplinary approach has facilitated a comprehensive evaluation of patients, allowing for the prompt identification and management of comorbidities and potential complications, thereby improving the outcome of surgical procedures even for high-complexity patients.

Conclusions: The LCP represents an efficient CP example for the diagnosis, staging, and preoperative assessment of patients with lung cancer. LCP is constantly supported by a multidisciplinary team. The preoperative pathway is entirely managed in a Day-Hospital setting, even for high-complexity cases requiring second and third-level exams. LCP also ensures rapid access to diagnosis and treatment and reduces costs to the Healthcare System. From 2018 to date, there has been a progressive and steady increase in access to LCP.

### 1.73. Abstract 73 (ePoster): Negative Pet/ct and Pleural Thickenings in Pleural Effusion: Do We Really Need More Exams?

**Virginia Santello, Filippo Lanfranchi, Gabriele Foltran and Lucio Michieletto**

Respiratory Disease Unit, Department of Cardiac Thoracic and Vascular Sciences, Ospedale dell’Angelo, Venice, Italy

Background: Medical thoracoscopy (MT) with pleural biopsies is the gold standard for Malignant Pleural Mesothelioma (MPM) diagnosis. PET-CT negativity, absence of pleural thickenings (PTs) or both, in patients with pleural effusion (PE) can delay MPM diagnosis. Usually, pleural pathologies are undiagnosed because of the absence of a dedicated pleural disease unit.

To determine whether MT should be performed to investigate PE of unknown origin despite PET-CT negativity or absence of PTs described at chest CT scan.

Methodology: Between January 2022 and January 2024, 42 patients with Malignant Pleural Effusion (MPE) who performed PET/CT scan prior procedure were consecutively enrolled. 22 of them received a diagnosis of MPM via MT and pleural biopsies. Patients were divided in 2 different groups based on pleural fluorodeoxyglucose (FDG) uptake at PET-CT scan: negative (group 1) and positive (group 2). Gender, age, exposure, pleural fluid cytology and diagnosis were evaluated.

Results: In 4/22 patients (18.8%), MPM was found despite PET-CT negativity. In the negative PET- CT group, 3/4 patients (75%) did not present PTs described at CT scan. Gender distribution was similar in two groups, with male predominance (75.0% vs. 83%); mean age was higher in group 1 (77 vs. 69 years). Exposure and smoking history were similar: asbestos exposure was 50.0% vs. 61.1%, global exposure for pneumotoxic agents was 75.0% vs. 88.9% and smoking history was 50.0% vs. 44.5% (Table 12). Pleural fluid cytology positivity for malignant cells was similar in two groups (75.0% vs. 61.1%).

Conclusions: According to MPM guidelines, our data shows that PET/CT should not be considered for MPM investigation; furthermore, the absence of PTs at chest CT scan does not exclude an underlying MPM.

In our experience, we suggest that patients with PE of unknown origin and asbestos exposure, MT should be performed even if they do not present neither PTs nor PET/CT positivity.

Further studies are needed to evaluate if a pleural disease unit can investigate and manage every PE of unknown origin, especially in well-known asbestos exposure working areas. 

### 1.74. Abstract 74 (ePoster): Safety and Feasibility of Ketamine for Rigid Bronchoscopic Interventions: A Single-Center Experience

**Antonio Sarubbi ^1^, Luca Frasca ^1^, Filippo Longo ^1^, Giovanni Tacchi ^1^, Fabio Costa ^2^ and Pierfilippo Crucitti ^1^**

1. Department of Thoracic Surgery, Fondazione Policlinico Universitario Campus Bio-Medico, Rome
2. Department of Anesthesia and Intensive Care, Fondazione Policlinico Universitario Campus Bio-Medico, Rome

Background: Rigid Bronchoscopy is considered the gold standard for the management of central airway obstruction (CAO), a potentially life-threatening condition that can arise from both malignant and benign causes. Anesthetic management of these patients is challenging. Indeed, the use of neuromuscular blocking drugs can abolish muscle tone, potentially leading to complete tracheobronchial obstruction and loss of airway patency. On the other hand, achieving deep anesthesia is necessary to ensure patient compliance during the procedure. Ketamine preserves respiratory drive and airway reflexes, exhibits bronchodilating properties, and provides profound analgesia. Given the limited reports on the feasibility of using ketamine in rigid bronchoscopy, we present our anesthetic protocol based on this medication. 

Methodology: We performed rigid bronchoscopic intervention in five patients with CAO: four with lung cancer due to intraluminal growth or extraluminal compression/infiltration, and one with postintubation tracheal stenosis. All patients underwent the procedure under spontaneous ventilation with total intravenous anesthesia using ketamine and midazolam. This combination was chosen to balance the side effects of both drugs (respiratory depression and hypotension for midazolam and respiratory stimulus and sympathomimetic effect for ketamine).

Results: All procedures were successful in restoring airway patency and were uneventful. Our anesthetic protocol allowed us to manage various procedures, including metal/silicon stent placement, dilation, and argon coagulation. The patients were compliant, and no general or drug-related side effects were reported. 

Conclusions: Ketamine-based total intravenous anesthesia is safe and feasible for anesthetic management of rigid bronchoscopy in CAO patients, maintaining spontaneous ventilation and airway patency. Further studies are needed to confirm these findings and to establish standardized protocols.

### 1.75. Abstract 75 (ePoster): Ulavats (Uniportal Laser-Assisted Video-Assisted Thoracoscopy) for Lung Metastasectomy: A Pilot Experience

**Carolina Sassorossi ^1,2^, Marco Chiappetta ^1,2^, Dania Nachira ^1,2^, Annalisa Campanella ^1^, Giuseppe Calabrese ^1,2^, Antonio Giulio Napolitano ^1,2^, Alessia Senatore ^1,2^, Khrystyna Kuzmych ^1,2^, Arianna Gallo, Claudia Leoni ^1,2^, Claudia Bellettati ^1,2^, Chiara Scognamiglio ^1,2^, Adriana Nocera ^1,2^, Filippo Lococo ^1,2^ and Stefano Margaritora ^1,2^**

1. Thoracic Surgery, A. Gemelli University Hospital Foundation IRCCS, Rome, Italy
2. Catholic University of Sacred Heart, Rome, Italy

Background: Pulmonary metastasectomy (PM) is a well-established treatment in the cure of oligometastatic cancer. Surgery should adopt the most lung sparing approach possible, to preserve pulmonary function and for potential additional lung resections. Laser technology has been introduced in the recent decades but only few experiences combining laser technology with VATS approaches have been reported till now. The main focus of our work is to report our pilot experience in performing lung sparing laser-assisted PM by Uniportal VATS (Uniportal Laser-Assisted VATS: U-LA-VATS). The surgical technique and peri operative results from our series of patients were analyzed.

Methodology: Between March 2021 and November 2023, among 98 patients who underwent PM a total of 22 patients (78%, 17, men, 22%, 5, women, mean age, 61.4 years; range, 13–83 years) were treated with laser-assisted PM at our Institution. Patients who underwent anatomical resection were excluded for the purpose of the analysis. The ULAVATS procedure adopted a modified laser-assisted lung resection technique for performing PM via VATS. Dedicated instruments are used, characterized by a long shape, curved shape, with distal and proximal articulations. A surgical laser system (Thulium + Diodo OUTPUT 30 W-10 W, Quanta System S.p.a., Solbiate Olona, Italy) were used and 550-μm sterile optical fiber conducted through a specific thoracoscopic handpiece introduced in the lowest part of the incision. 

Results: In 10 cases (46%) thulium laser-assisted resection was performed using uniportal video-assisted thoracic surgery (VATS), and in the remaining cases (12, 55%) the open access was used. In particular, lateral muscle sparing thoracotomy (27%) and mini-thoracotomy (27%) were performed (Table 13). 

In the thoracotomy group, mean duration of surgery was 95 ± 57.7 min, instead it was 73.5 ± 35.5 in the Uniportal VATS group (*p* = ns). We didn’t observe intra-operative complications or remarkable malfunction of the laser system and not report major complications after surgery and also the air-leak rate was nil for both approaches. Surgical margins were free from disease in all cases. The mean hospitalization after surgery was 2.9 ± 0.3 days for the U-VATS group and 3.8 ± 0.9 days for the thoracotomy group, this difference being statistically significant at the univariate analysis (*p*-value = 0.015) (Table 14).

Conclusions: ULAVATS is a safe and effective procedure, able to combine a parenchymal sparing exeresis with a mini-invasive approach. This procedure is associated with a shorter hospital stay compared to PM performed by thoracotomic approach.

### 1.76. Abstract 76 (ePoster): Diagnostic Yield of Contrast-Enhanced Ebus in Mediastinal and Hilar Lymphadenopathy: A Preliminary Study

**Ilaria Suriano, Luca Frasca, Filippo Longo, Antonio Sarubbi and Pierfilippo Crucitti**

Department of Thoracic Surgery, Fondazione Policlinico Universitario Campus Bio-Medico, Rome, Italy

Background: Contrast-enhanced endobronchial ultrasound transbronchial needle aspiration (CE-EBUS TBNA) is a minimally invasive technique that combines the traditional endobronchial ultrasound transbronchial needle aspiration (EBUS-TBNA) with the administration of a contrast agent, (sulphur hexafluoride) that enhance the visualization of blood flow within mediastinal and hilar lymph nodes. We investigated the application of this procedure in patients with radiologically evident bulky lymphadenopathy and nodes presenting central necrosis. 

Methodology: We conducted a retrospective observational study from April 2021 to December 2023 involving 49 patients. The patients were divided into two groups: EBUS-TBNA (*n* = 26) and CE-EBUS TBNA (*n* = 23). We included patients with suspected or confirmed lung cancer involving mediastinal lymph nodes, hilar masses and bulky lymphadenopathy or nodal metastases showing central necrosis. Patients with a certain or suspect diagnosis of autoimmune diseases and lymphoproliferative diseases, were excluded. For the CE-EBUS TBNA procedure, 4.8 mL of sulphur hexafluoride was administered intravenously. Morphological, echogenic, vascular characteristics, diagnostic accuracy, adequate sample collection and further molecular testing were collected and compared between the two groups. 

Results: In the CE-EBUS group, 56.5% patients had a lesion size >30 mm (bulky lesions), while in the EBUS group was the 61.5%. Lesions showing necrosis sign presence was found in 74% patients of the CE-EBUS group and 80% in EBUS group. Molecular testes were assessed with significantly results in the CE-EBUS group: 60.8% vs. 48.7% in the EBUS group. CE-EBUS showed superiority in terms of diagnostic accuracy: 91% vs. 73% in traditional EBUS. Sensitivity was 92% in CE-EBUS groups vs. 76% EBUS group.

Conclusions: In presence of bulky lymphadenopathy and nodes presenting central necrosis we observed CE-EBUS TBNA improves diagnostic accuracy. In addition, the use of contrast makes it possible to obtain a greater proportion of vital neoplastic tissue that is essential for performing molecular tests.

### 1.77. Abstract 77 (ePoster): Efficacy of Indwelling Pleural Catheters on Malignant Pleural Effusion: Focus on Quality of Life

**Martina Ubaldi ^1^, Paola Rebecca Iovine ^2^, Giuseppe Ielo ^3^, Francesco Todisco ^4^, Elena Paracchini ^5^ and Filippo Patrucco ^5^**

1. University of Roma La Sapienza, AOU Sant’Andrea, Italy
2. University of Napoli Federico II, AOU Monaldi, Italy
3. University of Catania, AOU Policlinico G. Rodolico—San Marco, Italy
4. University of Torino, AOU Molinette, Italy
5. Respiratory Diseases Unit, Medical Dpartment, AOU Maggiore della Carità di Novara

Background: Malignant pleural effusion (MPE) is the second leading cause of exudative effusions and involves about 15% of patients with advanced cancer. MPE is associated with limited life expectancy with a median survival of 4–7 months and determines a significant deterioration of quality of life (QoL) due to symptoms and repeated invasive pleural procedures. Indwelling tunneled pleural catheters (IPCs) are increasingly being used in the management of MPE and represent the treatment of choice in those patients with trapped/non-expandable lung and in whom pleurodesis failed.

The main purpose of this study would be to value the efficacy of IPC on symptom relief and improvement of quality of life. We also want to study the complication rate and their severity, as they can negatively impact the patient’s well-being.

Methodology: The aim of our study is to analyze patients evaluated for MPE at Interventional Pulmonology Unit of Ospedale Maggiore della Carità in Novara, who are eligible for IPC placement between March 2024 and September 2024. The IPC placement occurs during an outpatient visit, when both the patient and caregiver are educated in the use and management of the IPC, particularly, in periodic fluid draining and how to monitor the amount of drained fluid, as well as the possible appearance of new symptoms or adverse events. Our drainage protocol consists in emptying 500 mL daily for the first 5 days and then 500 mL daily or every two days according to the patient’s needs until complete depletion. Subsequently the patient is clinically re-evaluated at 10 days, 1 and 3 months. Symptom control and quality of life scores are obtained by the mMRC scale for dyspnea, VAS for pain and SGRQ. The complication severity is evaluated by the Clavien Dindo Classification. 

Results: The enrolment and follow up phase is still ongoing so at the moment we are not able to present definitive conclusions. We will be soon able to show more concrete results at the end of the recruitment phase, but the preliminary data appear encouraging. 

Conclusions: In this scenario the management of MPEs should focus on patient-centered goals such as symptom palliation, improvement of quality of life and minimizing invasive repeated procedures. The IPC placement has reported high efficacy and safety on several trials, so we expect to achieve significant results also in terms of improvement of quality of life. 

### 1.78. Abstract 78 (ePoster): Ebus-Tbna: Retrospective Analysis of the First Two Years of Activity at Our Center

**Annalucia Urgese ^1^, Roberta Rapanà ^1^, Giovanna Imbriglio ^1^, Francesca Signore ^1^, Vladimiro Dell’Anna ^2^ and Camillo Lopez ^1^**

1. Thoracic Surgery Unit, Vito Fazzi Hospital, Lecce
2. Pathological Anatomy Operational Unit, Vito Fazzi Hospital, Lecce

Background: The purpose of this study is to report on the experience of two years since the introduction of EndoBronchial UltraSound-guided TransBronchial Needle Aspiration (EBUS-TBNA) at our center. We specifically evaluate its diagnostic yield in terms of accuracy, sensitivity, specificity, histology, needle size, number of samples and the learning curve.

Methodology: We retrospectively analyzed a case series of 133 patients who underwent EBUS-TBNA at the Thoracic Surgery Unit in Lecce from January 2022 to April 2024. We sampled parenchymal lesions in 12 cases and lymph nodes starting from the most significant staging node (N3→N2→N1). Deep sedation with a laryngeal mask was performed in 74% of cases, while the remaining 26% used an orotracheal tube. Procedures were performed with Rapid On Site Examination (ROSE) in 97% of cases. Diagnostic accuracy was assessed based on definitive cytological reports. In cases without a diagnosis, verification was done through other biopsy or surgical procedures.

Results: Out of 133 procedures, TBNA was not performed in one case due to small lymph node size for staging purposes. Adequate sampling was achieved in 98% of cases. Among 111 diagnostic samples, 40 (36%) were true negatives. Fifty-four patients underwent biopsy verification, and in 72% of cases, the diagnosis was confirmed. The overall accuracy was 86%.

Multivariate regression analysis revealed increased diagnostic accuracy with higher PET uptake and larger lymph node size. Smaller-caliber needles demonstrated greater diagnostic power. However, increasing the number of samples from the same lesion did not improve diagnostic accuracy.

This study provides valuable insights into diagnostic performance of EBUS-TBNA in our clinical practise, supporting its use for immunophenotypic and biomolecular analyses in most cases of suspected lung neoplasms.

Sensitivity was 100% in diagnosing granulomatosis, metastases, neuroendocrine tumors, and hematological diseases. Specificity was 100% for neuroendocrine tumors and poorly differentiated neoplasms, 99% for lymphomas, 98% for metastases, 96% for squamous cell carcinoma and non-neoplastic diagnoses, and 95% for adenocarcinomas.

The learning curve was initially steep in the first six months after the procedure’s introduction, but it gradually decreased with an increasing number of procedures, eventually reaching an optimal plateau in the last year.

Conclusions: Based on our initial experience, EBUS-TBNA appears to be a safe and reliable method for diagnosing various mediastinal and pulmonary pathologies.

It provides suitable biological samples for biomolecular testing, with diagnostic accuracy consistent with existing literature.

### 1.79. Abstract 79 (ePoster): The Prognostic Role of Flexible Bronchoscopy in Patients with Inhalation Injury: A Systematic Review and Meta-Analysis

Erika Virdis ^1,^*, Chiara Minonne ^1^, Pietro Pirina ^1,2^ and Alessandro G. Fois ^1,2^

1. Department of Medicine, Surgery and Pharmacy, University of Sassari, Sassari, Italy
2. Clinical and Interventional Pneumology, University Hospital of Sassari, Sassari, Italy

Background: Smoke inhalation injury may flow in unpredictable pattern and warrants close monitoring after exposure. For this reason, it is particularly useful to have an assessment tool available in clinical practice that can provide information on the extent of the damage, and which can have a prognostic value. Nowadays bronchoscopy is considered the gold standard for inhalation injury assessment: it allows sample collection for microbiology testing and targeted antimicrobial therapy and washing out of the bronchial tree, improving ventilation and reducing the risk of atelectasis and pneumonia, nevertheless, its prognostic role is still debated. Over the years, based on the bronchoscopic findings, multiple scores were proposed to evaluate the damage caused by inhalation injury and to predict the prognosis. Among proposed scores, the most used three are: the abbreviated injury score (AIS), the inhalation injury severity score (I-ISS), and the bronchoscopic mucosal score (MS). 

Each system uses an ordinal scale (AIS: 0–4; I-ISS and MS: 0–3) with higher scores representing more severe injuries. Despite the different types of expression, all scales consider absence or presence of erythema, oedema and necrosis. 

Several studies tried to correlate the degree of severity with clinical outcomes, in order to understand whether bronchoscopy had a real prognostic role. 

Methodology: In this context, we conducted a systematic review and meta- analysis to evaluate the predictive role of bronchoscopy and the correlation between severity of inhalation injury and clinical outcomes, such as mortality, hospital length of stay (HLS), intensive care length of stay (ICULS), duration of ventilation (VD), and development of pneumonia. Our study was conducted complying the PRISMA 2020 statement and the full protocol is available at PROSPERO CRD42024518244).

Results: We found a significant increase in the mortality odds for patients with high-grade injury compared to those with low-grade lesions (OR = 2.94, CI 1.78 to 4.87, *p* < 0.001). Furthermore, statistically significant difference was present in HLS (pooled SMD = 1.05, 95% CI 0.39 to 1.70, *p* = 0.002), ICULS (SMD = 0.53, 95% CI 0.07 to 0.98, *p* = 0.02), duration of ventilation (SMD = 0.62, 95% CI 0.19 to 1.06, *p* = 0.005) and development of pneumonia (OR = 1.60, 95% CI 0.63 to 4.11, *p* = 0.33) in the high-grade group when compared to the low-grade. 

Conclusions: Our study supports the need of bronchoscopy evaluation in the early phases of exposition to smoke inhalation injury, however, further studies are required to determine the real prognostic capacity of bronchoscopy. 

### 1.80. Abstract 80 (Video): Uniportal Robotic (U-Rats) Middle Lobectomy with Direct Suture of the Bronchus

**Mary Bove, Nicola Martucci, Giorgia Opromolla, Giuseppe De Luca, Antonello La Rocca, Carmine La Manna and Edoardo Mercadante**

Thoracic Surgery Unit, Istituto Nazionale Tumori IRCCS Fondazione G. Pascale, Naples, Italy

Background: Since 2022, at our institution, we routinely perform robotic lobectomies through uniportal approach (U-RATS). The video shows a U-RATS middle lobectomy with direct suture of the bronchus, performed for an incidental double indication: a peripheral adenocarcinoma of the middle lobe and a proximal endobronchial hamartoma.

Methodology: A 73-years-old man referred to our attention with the radiological finding of a pulmonary nodule of about 3 cm of the middle lobe with positive PET-CT scan (SUV 3.7), thus, we indicate robotic lobectomy. A 4 cm incision at the 5th intercostal space between the anterior and the middle axillary lines is performed. The presence of the nodule crossing the horizontal fissure confirmed the indication to perform the middle lobectomy with a minimal wedge resection of the upper lobe.

Results: After docking of Da Vinci Xi, the three arms are placed through the single incision, with the camera at its posterior edge. The video shows the dissection and section of the middle pulmonary vein using robotic Hem-o-lok. The anterior oblique fissure is completed with a firing of a blue Sureform 45 stapler, as well as the horizontal fissure, en bloc with a small wedge resection of the anterior segment of the right upper lobe. The middle lobar bronchus is isolated, sutured and sectioned with Sureform 45 blue. Then, the branches of the pulmonary artery are sutured and sectioned with Sureform 45 white. Extraction of the specimen with endo-bag is showed. Intraoperative fiber optic bronchoscopy showed the presence of a lesion in the bronchial stump, included in the suture line. 

For this reason, the bronchial stump has been cut more proximally with robotic scissors and a direct running suture of the bronchus is performed. Overall surgical time was 200 min. No postoperative complications were observed. The chest drain was removed an the fifth postoperative day and the patient was discharged on the same day. Histological examination revealed an adenocarcinoma pT_2_, N0, M0 plus endobronchial hamartoma.

Conclusions: The video demonstrates that U-RATS is a safe and feasible technique, even in more challenging procedures, such as bronchial reconstruction. 

### 1.81. Abstract 81 (Video): Robotic Surgical Exeresis of Horseshoe Esophageal Leyomioma: Details of Surgical Technique

**Carlo Curcio ^1^, Dario Amore ^2^ and Dino Casazza ^2^**

1. Pineta Grand Hospital, Thoracic Surgery Unit, Castelvolturno (CE) ITALY
2. AO dei Colli, Monaldi Hospital Thoracic Surgery Unit (NA) ITALY

Background: The Authors take inspiration from a case of robotic exeresis of a large esophageal leiomyoma to illustrate a detail of surgical technique, which minimizes the possibility of complications, especially the onset of an esophageal fistula.

Methodology: The video shows a case of robotic exeresis of a large leiomyoma of the horseshoe-shaped thoracic esophagus. The Authors higlight some particular aspects of the surgical technique used, which made the operation safer especially with regards to the prevention of a postoperative esophageal fistula. In fact, the exeresis with robotic technique (Da Vinci XI) was carried out under esophagoscopic guidance throughout the entire surgical procedure with the dual objective: (1) highlight the margins of the leiomyoma better with insufflation than the esophageal mucosa, and (2) above all endoscopical check any lesions of the esophageal mucosa in order to repair immediately intraoperatively.

Results: The surgery was performed entirely with robotic technique, no intraoperative esophageal lesions occurred. The postoperative course was regular, with the resumption of oral feeding already after 58 h, without any functional problems. Discharge occurred on the sixth postoperative day.

Conclusions: Robotic surgery is the gold standard for the exeresis of this type of rare esophageal neoplasms, and the use of continuous intraoperative esophagoscopic control facilitates exeresis and prevents the onset of esophageal fistulas. 

### 1.82. Abstract 82 (Video): Dual-Portal Robotic-Assisted Thoracic Surgery (Drats) and Indocyanine Green-Navigated Segmentectomy

**Khrystyna Kuzmych, Carolina Sassorossi, Dania Nachira, Maria Teresa Congedo, Stefano Margaritora and Elisa Meacci**

Department of General Thoracic Surgery, Fondazione Policlinico Gemelli IRCCS, Catholic University of Sacred Heart of Rome, 00168 Rome, Italy

Background: In the constantly advancing field of thoracic surgery, new techniques are being developed to enhance patient outcomes and reduce invasiveness. Based on our expertise in Uniportal Video-Assisted Thoracic Surgery (U-VATS) and standard robotic methods, we recently introduced Fully-Biportal Robotic-Assisted Thoracic Surgery (RATS), termed F-Dual-Portal RATS (F-DRATS). Integrating robotic assistance has transformed thoracic surgery, allowing for greater precision and minimal invasiveness. F-DRATS represents an advancement in this evolution, combining the advantages of robotic technology with dual-port access to reduce the number of incisions further and enhance patient recovery.

Methodology: We present a case involving a 57-year-old female with a history of malignant melanoma, surgically treated in 2020, who presented in 2024 with an 11 mm subsolid nodule in the lower lingular segment detected on a chest CT scan. Pulmonary function tests indicated potential air trapping. After a multidisciplinary discussion and 3D reconstruction evaluation, segmentectomy was chosen to preserve lung parenchyma while ensuring appropriate oncological intervention. The F-DRATS approach was defined as robotic thoracic surgery performed through two intercostal incisions without rib spreading, using the robotic camera, dissecting instruments, and exclusively robotic staplers. We herein describe our F-DRATS approach in lingulectomy and lymphadenectomy of stations 5, 6, 7, and 10 using the daVinci Surgical System.

Results: The F-DRATS procedure was performed using a 2–3 cm working port in the fifth intercostal space along the posterior-axillary line and a 1.2 cm working port at the eighth intercostal space on the midaxillary line. Robotic staplers and indocyanine green (ICG) fluorescence allowed for precise delineation and resection of the intersegmental plane. The postoperative course was uneventful, with the patient discharged on the third postoperative day. Final pathology confirmed a low-grade adenocarcinoma, with all resected lymph nodes free of metastases. The ICG fluorescence method provided a clear view of the intersegmental plane, facilitating accurate segmentectomy.

Conclusions: F-DRATS, with its unique combination of robotic technology and dual-port access, is a promising and potentially valuable tool in thoracic surgery. ICG fluorescence navigation, providing real-time visualization of the intersegmental plane, enhances the precision and safety of segmentectomies. This technique shows potential for reducing invasiveness and improving recovery times compared to traditional methods. While further studies are needed to validate and refine this technique, early results are encouraging and suggest that F-DRATS could indeed become a standard approach in thoracic surgery.

### 1.83. Abstract 83 (Video): “One-Shot” Simultaneous Stapling of the Lobar Bronchus and Pulmonary Artery during Left Lower Lobectomy for Destroyed Lung

**Stefano Lovadina ^1^, Marina Troian ^1^, Davide Drigo ^3^, Alessia Arbore ^1^, Elisabetta Benci ^1^, Simonetta Masaro ^1^, Enrico Arbore ^1^, Alice Ravasin ^2^ and Maurizio Cortale ^1^**

1. Thoracic Surgery Unit, Cattinara University Hospital, Trieste, Italy
2. Thoracic Surgery Unit, Careggi University Hospital, Florence, Italy
3. Department of General Surgery, Cattinara University Hospital, Trieste, Italy

Background: In common practice, VATS lobectomy involves isolation and separate sectioning of hilar structures. Although described in literature, en-bloc simultaneous bronchus and vessels resection using a mechanical stapler is still considered controversial due to the following potential risks: intraoperative hemorrhage from vascular rupture, bronchopleural/bronchovascular fistula, suboptimal oncologic resection due to incomplete lymph node dissection in the lung fissure, and unintentional sectioning of arterial branches for upper lobes in case of lower lobectomies. Nevertheless, according to literature data this technique seems to be feasible, safe, rapid, and effective in selected cases.

Methodology: A 70-year-old patient on long-term medical therapy for atypical mycobacterial infection presented on CT scan diffuse bronchiectasis of the left lower lobe with extensive lung destruction due to chronic bacterial colonization and periodic infectious flare-ups. After multidisciplinary evaluation, the patient was referred to the thoracic surgery unit for elective left lower lung resection.

Results: The patient underwent biportal VATS left lower lobectomy. Surgical access was achieved through a 4-cm vertical mini-thoracotomy with total muscle sparing and an additional more caudal access on the mid-axillary line for camera insertion. Using a radiofrequency sealing device, the pulmonary ligament was dissected, and the left lower pulmonary vein was prepared and divided using a vascular endostapler. By means of an antero-posterior approach and eased by the presence of a complete fissure, we then performed simultaneous stapling of the left lower lobe pulmonary artery and bronchus using an extra-thick stapler reload. The procedure was completed by section of the residual parenchyma. Subsequent intra-operative water-submersion test was deemed negative for air-leaks. Postoperative course was uneventful. Histologic examination of the surgical specimen confirmed the presence of chronic granulomatous and lymphocytic inflammation within permanent enlargements of bronchial structures.

Conclusions: This case illustrates a practical application of simultaneous broncho-vascular stapling during VATS lower lobectomy. We believe this is a quick, safe, and easier technique compared to conventional separate dissection, and a valuable addition to the armamentarium of thoracic surgeons. In our opinion, it can be helpful even in patients with incomplete fissures, representing a simpler and less dangerous alternative compared to the retrograde approach. Moreover, it is usable even by less experienced surgeons and it may contribute to reduction of air leaks, blood loss, and operating times. Further studies may be required to validate its use, especially in the oncological field.

### 1.84. Abstract 84 (Video): Robotic-Assisted Chest Wall Resection and Reconstruction: A Case Report

**Federico Mathieu ^1^, Chiara Catelli ^2^, Antonio Fabiano ^1^, Roberto Corzani ^1^, Susanna Guerrini ^3^, Piero Paladini ^1^ and Luca Luzzi ^2^**

1. Lung Transplant Unit, Department of Medical, Surgical and Neuro Sciences, Azienda Ospedaliero-Universitaria Senese, University of Siena, 53100 Siena, Italy
2. Thoracic Surgery Unit, Department of Medical, Surgical and Neuroscience Sciences, University Hospital of Siena, 53100 Siena, Italy
3. Unit of Diagnostic Imaging, Department of Radiological Sciences, Azienda Ospedaliero-Universitaria Senese, University of Siena, 53100 Siena, Italy

Background: We report the first case, to our knowledge, of chest wall resection and reconstruction using robotic technique for a benign rib tumor.

Methodology: A 36-year-old woman underwent a chest MRI for evaluation of a benign mammary lesion and worsening left chest wall pain. The MRI revealed a lesion on the left chest wall corresponding to the median arch of the fifth rib. A CT scan confirmed a bulging structural alteration of approximately 10 cm of the rib without any signs of surrounding tissue infiltration. Due to the suspect of rib osteochondroma, we opted for a robotic minimally invasive radical resection.

Results: The surgery was performed with the patient in a right lateral position, using three surgical accesses. The robot was docked from the left side with only two arms and the camera. A unipolar hook was used for dissection and to encircle the normal portion of the rib before osteotomy. The bipolar forceps were used on the left robotic arm. Both intercostal muscles were dissected from the rib, which was mobilized from the posterior chest muscular plane. The rib was then cut in the posterior arch and removed from the structure behind, with the anterior arch cut using an endoscopic rib cutter. To restore chest structural integrity and prevent pulmonary herniation, a non-absorbable mesh was sutured to the internal chest wall using four running 3–0 V-lock barbed sutures. The postoperative diagnosis was benign osteochondroma with 1 cm margins on both sides. No intraoperative complications were registered, with a total blood amount of 150 mL at the end of the procedure. The patient was discharged in good conditions on the 4th postoperative day, with an excellent pain control at discharge.

Conclusions: Robotic chest wall resection and reconstruction is feasible and safe. The use of robotic endowrist allows for easy chest wall reconstruction using mesh and running barbed sutures, without the need to make an additional skin incision at the level of the wall defect, potentially allowing for a reduction in post-operative pain and a quick recovery.

### 1.85. Abstract 85 (Video): Fully Dual Portal Robotic Assisted Thoracic Surgery Right Lower Lobectomy

**Elisa Meacci, Dania Nachira, Khrystyna Kuzmych, Giuseppe Calabrese, Maria Teresa Congedo and Stefano Margaritora**

Fondazione Policlinico Universitario A.Gemelli I.R.C.C.S- Università Cattolica del Sacro Cuore di Roma- Roma

Background: In the landscape of thoracic surgery, innovation continually drives progress, offering novel approaches to address complex pathologies while prioritizing patient well-being. Dual-portal robotic-assisted thoracic surgery (DRATS) represents the next frontier in this evolution. In this report, we describe our experience with Fully-Dual-portal robotic-assisted thoracic surgery (F-DRATS) approach for right lower lobectomy. 

Methodology: We define as F-DRATS the robotic thoracic surgery performed by two intercostal incisions without rib spreading, using the robotic camera, robotic dissecting instruments and exclusively robotic staplers.

We herein describe a case of F-DRATS right lower lobectomy using the da Vinci Surgical System.

Results: A 64-year-old female with a history of smoking and ground-glass opacity in the right lower lobe, initially discovered in 2021 was addressed to our Department. The opacity grew in size over the years, actually measuring 13 × 14 mm on the latest CT scan (Figure 17A). The preoperative PET-CT scan did not show increased uptake of 18-FDG (Figure 17B).

A 3-cm working port using a wound protector (Alexis wound retractor XS^®^, Applied Medical, Rancho Santa Margarita, CA, USA) was created in the fifth intercostal space along the posterior-axillary line as a main port. A 1.2 cm working port was created, in accordance with the patient body shape, in the 8th intercostal space, on the midaxillary-line (Figure 17C). The lung nodule was identified and resected using sequential charges of a robotic stapler and then sent for a fresh frozen section. The pathologist diagnosed an infiltrating adenocarcinoma, prompting us to proceed with a lobectomy and lymphadenectomy.

The postoperative period was uneventful. The chest tube was removed on the fourth postoperative day, and the patient was discharged on the same day.

Conclusions: We described a successful case of F-DRATS right lower lobectomy. To the best of our knowledge no other cases have been previously described in Italian and/or European literature.

### 1.86. Abstract 86 (Video): Robotic Excision of an Atypically Located Pericardial Cyst

**Giorgia Opromolla, Mary Bove, Giuseppe De Luca, Nicola Martucci, Antonello La Rocca, Carmine La Manna and Edoardo Mercadante**

Thoracic Surgery Unit, Istituto Nazionale Tumori IRCCS Fondazione G. Pascale, Naples, Italy

Background: Pericardial cysts are uncommon benign malformations, usually congenital. In most cases they are located at the right cardiophrenic angle, but atypical locations are possible. The video shows an EBUS-TBNA aspiration and subsequent robotic excision (RATS) of a pericardial cyst at the right paratracheal area of the superior mediastinum. 

Methodology: A 60-years-old asymptomatic woman referred to our attention with a CT scan showing a right paratracheal cyst of 66 mm, with compression and dislocation of the trachea and adjacent vessels. The video shows the EBUS-TBNA with aspiration of 120 mL of serous fluid and consequent complete collapse of the anechoic region. The cytologic evaluation of the fluid yielded no malignant cells. One month later, the CT scan revealed the recurrence of the cyst, so we decided for the robotic surgical excision. 

Results: A right three-portal RATS approach with CO_2_ inflation is showed in the video, with the patient in supine position with a bump on the operative side. The cyst, of about 7 cm in diameter, is evident in the upper mediastinum, located posteriorly to the SVC and medially to the azygos vein. These vessels are dislocated and compressed. The cyst arises deeply in the mediastinum, with compression of the right pulmonary artery and the right superior pulmonary vein. A pedicle, toward the pericardium and close to the emergence of the right pulmonary artery, revealed the pericardial origin of the cyst. The mediastinal pleura is incised and the cyst is dissected from azygos vein, SVC, trachea and right pulmonary artery. Then it is transected at the level of the pedicle and entirely excised. 

The patient had no post-operative complications and was discharged on post-operative day 2. Pathological examination confirmed the diagnosis of a pericardial cyst. No recurrence was identified at the CT scan performed one month later.

Conclusions: EBUS-TBNA is useful for visualization, aspiration and diagnosis of atypically located pericardial cysts but, of course, it does not ensure their permanent resolution. The video demonstrates that robotic excision, using CO_2_ inflation, is a feasible, safe and effective approach allowing the dissection to proceed very deeply in the mediastinum.

### 1.87. Abstract 87 (Case Report-Poster): Post-Ablation Atrial-Esohageal Fistula: Surgical Treatment without Extracorporeal Circulation

**Riccardo Amatucci, Mara Romito, Domenico Pourmolkara, Alberto Melis, Jessica Malizia, Jacopo Vannucci and Francesco Puma**

Thoracic Surgery, University of Perugia; Perugia

Background: An atrial-esophageal fistula is a possible complication after cardiac ablation procedure, leading to patient death in 80% of cases. Epidemiology is rare (<0.1% to 0.25% post-ablation). It is more frequent in males, and it tends to occur around three weeks after cardiac procedure. The causes can be referred to the used thermal-heat, possible ischemic damage, esophageal hypomotility, increased gastric reflux, nerve damage and general anesthesia. Here, we report our surgical experience about a patient developing an atrial-esophageal fistula almost one-month post-cardiac ablation.

Case presentation: Male patient, 75 y.o. with non-specific symptoms (fever, nausea, vomiting) and dysuria under antibiotic treatment. Anamnesis relevant for hypertensive dilatative-hypokinetic cardiomyopathy, diabetes mellitus type 2, primary prevention ICD, paroxysmal atrial fibrillation subjected to multiple cardioversion procedures. The patient was hospitalized because of suspected urosepsis. At the blood tests increased inflammatory markers and mild anemia were revealed. Hemocultures were positive for S. Parasanguinis. Because of altered state of consciousness, right limbs movements and speech impairment, and epileptic seizure, the patient underwent brain-CT scan, EEG, MRI which revealed subacute vascular event. TEU showed fluctuant formations in the left atrium. The following total-body CT-scan demonstrated air presence in the left atrium suggestive for atrial-esophageal fistula. The patient was subjected to surgery without cardiopulmonary bypass thought right posterolateral thoracotomy at V intercostal space with intercostal muscle flap preparation. Atrial-esophageal fistula was evident after accurate adhesiolysis. Atrial defect was repaired using pericardial heterologous patch and the esophagus sutured by layers. The patient moved to rehabilitation center 154 days following the procedure.

Conclusions: Atrial-esophageal fistulas are rare complications with high mortality rate, that might be related to thermal-heat damage on the esophagus during cardiac ablation procedure. The patient can present with non-specific symptoms. Imaging techniques such as CT scan with i.v. and oral contrast media appear to be the best method to get the diagnosis. Surgery success is related to early diagnosis and fast surgical intervention.

### 1.88. Abstract 88 (Case Report-Poster): The Key Role of the Video-Assisted Thoracic Surgery (Vats) in the Diagnosis and Treatment of Peritoneal Communication (Ppc)

**Luigi Gaetano Andriolo ^1^, Paolo Ria ^2^, Luca Luongo ^3^, Anna Zito ^2^, Vilma Martella ^2^, Emiliana Ferramosca ^2^, Francesca Signore ^1^, Silvia Matino ^2^, Davide Gianfreda ^2^, Maria Caterina Carbonara ^2^ and Marcello Napoli ^2^**

1. Unità Operativa Complessa di Chirurgia Toracica, Ospedale Vito Fazzi, Lecce
2. Unità Operativa Complessa di Nefrologia, Ospedale Vito Fazzi, Lecce
3. Unità Operativa Complessa di Medicina Nucleare, Ospedale Vito Fazzi, Lecce

Background: Peritoneal communication (PPC) is a rare condition occurring in patients undergoing long lasting automated peritoneal dialysis (APD), with an incidence of approximately 2% in the first year of treatment. Patients develop pleural effusion through diaphragmatic defects or blebs on the right side in 90% of cases. To date, there is no consensus regarding the definitive method to treat PPC and also the diagnosis requires a multidisciplinary work. The aim of the present study is to describe our diagnostic and therapeutic approach to PPC, in which the video-assisted thoracic surgery (VATS) plays a pivotal role.

Case presentation: From January 2022, 2 cases of PPCs have been diagnosed in as many patients (1 M and 1 F) undergoing APD treatment at the Nephrology Unit and Dialysis in our hospital. The causes of end stage renal disease were congenital obstructive uropathy and chronic glomerulonephritis, respectively. The presence of recurrent right pleural effusion despite repeated thoracentesis and chest tube positioning raised the suspicion of communication peritoneal-pleural, confirmed by the passage of ^99m^TC in the pleural cavity during peritoneal scintigraphy.

The chest CT images obtained at the same time also allowed us to hypothesize the location of PPC in the tendon center of the diaphragm. VATS permitted us not only the definitive diagnosis but also the treatment.

As described in the rare case reports already published, the surgical intervention consisted in direct closure of little diaphragmatic fistulas by stitches made of polypropylene (Prolene^®^) and also in affixing metal clips at the bottom of the same sites. This further technical tip, however not described elsewhere, probably facilitated the success of the treatment in the absence of the relapses described in the cases treated with only the direct suture of PPCs. At the end of the procedure, fibrin glue (Tisseel^®^) was insufflated to stimulate tissue regeneration.

To date, treated patients could resume their APD therapy without signs of recurrence.

Conclusions: A multidisciplinary approach and the availability of VATS allowed diagnosis and effective treatment of patients treated with APD and complicated by PPC.

### 1.89. Abstract 89 (Case Report-Poster): Endoscopic Management of Malignant Tracheobronchial Stenosis with Stent-in-Stent Placement: A Case Report

**Dr. Ludovica Benuzzi, Dr. Francesca Accordino, Dr. Giulia Fabietti and Dr. Marco Trigiani**

Firenze—Università degli Studi di Firenze—Azienda Ospedaliero-Universitaria di Careggi

Background: Airway stenting is an effective approach to manage malignant airway disease (MAD), leading to improvement in survival [17].

Case presentation: A 48-year-old man, former smoker with no relevant medical history, reported significant weight loss and persistent cough without fever, haemoptysis or dyspnoea. He underwent a chest CT scan, which documented an inhomogeneous mass reducing the left main bronchus (LMB) lumen without obstruction; the mass was 18-FDG PET positive. A CT-guided lung needle-biopsy performed in another hospital revealed lung adenocarcinoma (PD-L1 and driver mutations negative). A second CT scan performed before the beginning of treatment showed pulmonary atelectasis due to obstruction of the LMB. A fibrobronchoscopy was therefore performed in our centre, with evidence of distal tracheal stenosis (with lumen reduction of 50%) and complete obstruction of the LMB due to ab extrinsic compression; a metallic 20 × 16 × 16 Y-shaped stent was placed. For persistent compression of LMB distal to the stent, a second 16 × 40 metal stent was placed: the proximal part of this stent was inserted into the left arm of the previously positioned Y-shaped stent, so the left bronchial hemisystem was finally pervious.

Conclusions: Airway stenting in MAD is an effective palliation treatment [18]. Y-shaped silicon stents have been traditionally used to treat malignant carinal stenosis, but they have poor shape-adaptability, difficult placement and secretion retention [19] Self-expanding Y metal stents are relatively easy to insert and they lead to less secretion retention; but their length cannot be adjusted by clinicians like silicone Y stent [20]. Our clinical case agrees with the future perspectives of customized stent placement for the specific patient and to our knowledge it seems to be the only case of stent-in-stent metallic placement in the literature.

### 1.90. Abstract 90 (Case Report-Poster): Misdiagnosed Asthma in a 32-Year-Old Woman: A Case Report

**Margherita Biagini ^1^, Giovanni Buonocore ^1^, Laura Carrozzi ^1^ and Alessandro Ribechini ^2^**

1. Pulmonary Unit, Cardiothoracic and Vascular Department, University of Pisa, Pisa, Italy
2. Service of Thoracic Endoscopy, Cardiothoracic and Vascular Department, University of Pisa, Pisa, Italy

Background: We describe the case of a 32-year-old woman with asthma-like symptoms for about a year, who was referred to the Thoracic Endoscopy of our hospital for evaluation, following the finding on chest CT of an endoluminal tracheal mass. 

Case presentation: The patient had been presented, for about one year, nonspecific respiratory symptoms such as dyspnea on mild exertion, chest tightness and wheezing. At pulmonary evaluation, spirometry showed a very severe airway obstruction (FEV1 0.91 L, 26% of predicted value), the diagnosis of asthma was made and an inhalation therapy was prescribed. Due to persistence of symptoms, a chest CT scan with contrast was performed, showing a tracheal mass of about 20 mm in maximum diameter with endoluminal development. Bronchoscopy confirmed the presence of a roundish neoformation with superficial vascularization and cerebroid appearance sub-occluding the trachea (estimated obstruction around 90%) with longitudinal extension of 2 cm and at 6 cm from the glottic plane and 4 cm above carina. Rigid bronchoscopy was performed with Nd:YAG laser coagulation of the mass and subsequent mechanical debulking with rigid bronchoscope and forceps; at the end of the procedure, a pathological infiltration of the left anterolateral wall of the trachea was detected. Histologic examination allowed diagnosis of adenoid cystic carcinoma. In another medical center, the patient underwent robotic-assisted tracheal resection with ECMO support and then was treated with adjuvant proton therapy. Currently, patient is in clinical and instrumental follow-up.

Conclusions: Primary tracheal tumours are rare. Adenoid cystic carcinoma is the second most frequent type of primary tracheal tumours. The mean age at diagnosis is estimated to be around 45 years and cigarette smoking does not appear to be major risk factor. From a clinical point of view, it is usually silent until it results in at least 75% tracheal stenosis. The symptoms are dyspnoea, wheezing, cough and chest tightness. Therefore, in light of young mean age at diagnosis and asthma-like clinical presentation, a misdiagnosis of asthma is often made, delaying the correct diagnostic-therapeutic process and increasing the risk of emergency bronchoscopic procedures for severe dyspnoea [21,22]. 

Primary tracheal tumours are often difficult to diagnose, because of their nonspecific clinical presentation that mimics other respiratory conditions, particularly asthma. Therefore, it is needed to suspect this diagnosis in young patients with asthma-like symptoms not-responders to inhalation therapy.

### 1.91. Abstract 91 (Case Report-Poster): A Tricky Case of Pleural Effusion

**Sara Bombelli ^1,2,3^, Jacopo Civello ^1,2^, Carmine Riccio ^1^, Fabrizio Nava ^1^, Salvatore Longobardi ^1^ and Paolo Tarsia ^1^**

1. Department of Pneumology—Grande Ospedale Metropolitano Niguarda—Milan (Italy)
2. Post Graduate School in Respiratory Medicine, Università degli Studi di Milano—Milan (Italy)
3. Master’s Degree in Interventional Pneumology—Università degli Studi di Firenze—Firenze (Italy)

Background: This report aims to describe a case of atypical Meigs syndrome in a 51-year-old woman, highlighting the importance of considering this diagnosis in the presence of unexplained pleural effusion without ascites, especially when gynecological pathology is present. Given the rarity of Meigs syndrome, maintaining a high index of suspicion is crucial for accurate diagnosis.

Case Presentation: A 51-year-old woman with a medical history of asthma, anxiety-depressive syndrome, recently diagnosed arterial hypertension, and fibromatous uterus scheduled for surgery presented with a pleural effusion after an episode of influenza treated with antibiotics. Her home medications included beclometasone/formoterol, delorazepam, amitriptyline, aripiprazole, and triptorelin. A chest X-ray revealed a right pleural effusion. We performed a diagnostic and evacuative thoracentesis: pleuric fluid was negative for malignant cells and bacterial infections; Light’s criteria were suggestive for an exudate. Despite thoracentesis, a follow-up CT scan indicated increased pleural effusion with lower and middle lobe collapse. PET imaging did not show significant pleuroparenchymal uptake but revealed mild heterogeneous uptake in the fibromatous uterus. Right thoracoscopy and biopsy of the parietal pleura indicated chronic pleuritis. After steroid therapy, at revaluation, recurrent pleural effusion was noted. After a multidisciplinary discussion, suspecting atypical Meigs syndrome, we recommended pleural drainage and continuation of the surgical removal of the uterine fibroma. During laparotomy, a fibroma of the right ovary was discovered, and the patient underwent total hysterectomy with bilateral salpingo-oophorectomy. Histology of the ovarian tissue confirmed a fibroma. Post-surgery, follow-ups showed no recurrence of pleural effusion. The resolution of pleural effusion post-surgery supported the diagnosis of atypical Meigs syndrome, characterized by the presence of an ovarian fibroma and pleural effusion without ascites.

Conclusions: This case highlights the importance of considering Meigs syndrome and atypical Meigs syndrome in patients with unexplained pleural effusions, particularly in the presence of gynecological pathology. The diagnosis of atypical Meigs syndrome can be challenging in the absence of ascites, requiring a high index of suspicion and thorough diagnostic evaluation. The resolution of symptoms post-surgery confirms the crucial role of tumor removal in managing these syndromes. Accurate identification and management of atypical Meigs syndrome can significantly improve patient outcomes, emphasizing the need to consider this rare disease when gynecological conditions are present.

### 1.92. Abstract 92 (Case Report-Poster): Kinking of the Left Lower Lobar Bronchus after a Left Upper Lobectomy

**Maria Caterina Bottoli, Marco Monteverde, Michele Giovanardi, Stefano Sestini, Angelo Carretta**

ASST Carlo Poma Mantova, Mantova

Case Presentation: 75-year-old woman with a diagnosis of adenocarcinoma of the left upper lung lobe. In previous medical history, aortic and mitral valve replacement, sternotomy and pulmonary vein ablation with auricular exclusion; previous right mastectomy for cancer with subsequent negative follow-up.

Once the cardiopulmonary evaluation and preoperative staging were completed, the patient underwent left upper VATS lobectomy with lysis of the pulmonary ligament. On the second postoperative day, due to left lung hypoventilation, she repeated a chest X-ray which highlighted atelectasis of the residual parenchyma on the left (Figure 18), FBS with evidence of edematous padding of the mucosa of the lower lobar bronchus which could not be explored by the endoscopic instrument and a chest CT scan (Figure 19), which reports kinking of the lower lobar bronchus, just before the segmental branches, with reduction in its caliber until it appears collapsed for a stretch of approximately 7 mm with an endopleural picture of initial empyematization.

Therapy with HFNC (High Flow Nasal Cannula) was undertaken with good compensation of respiratory exchanges, but persistent parenchymal dysventilation, recurrent episodes of atrial fibrillation and initial signs of septic state.

For this reason, on the fifth postoperative day the patient underwent surgical revision with repositioning and straightening of the lower lobar bronchus and pexy and fixation of the lower lobe to the diaphragm in thoracotomy (Figure 20). 

During the postoperative course, the patient was treated with NIV for a few days and subsequently with HFNC and respiratory physiotherapy, until she was completely weaned from ventilator support. Control bronchoscopy demonstrated patency and correct alignment of the lower lobar bronchus and its branches.

The patient was re-evaluated 2 months after discharge, after a period of respiratory rehabilitation in a dedicated ward, with very good general conditions, complete resumption of daily activities without dyspnoea and with normal gas exchanges. On chest X-ray, well expanded lung parenchyma without atelectatic areas.

Conclusions: Kinking of the lower lobar bronchus following upper lobectomy is described in few works in the literature. In our experience, we confirm that lysis of the pulmonary ligament in upper lobectomy can be a risk factor favoring this complication and that the treatment of choice is the repositioning and surgical fixation of the residual lower lobe.

### 1.93. Abstract 93 (Case Report-Poster): Tracheal Diverticulum: Two Rare Cases of Tracheal Diverticula

**Sara Cagnetti, Gianluca Ancona, Casimiro Eugenio Giorgetta, Francesco Inzirillo, Eugenio Ravalli, Giuseppe Naldi and Paolo Scanagatta**

Division of Thoracic Surgery, ASST Valtellina e Alto Lario, “Eugenio Morelli” Hospital, 23035 Sondalo, Italy

Background: Tracheal diverticula (TD) are generally benign and are often discovered incidentally through radiologic imaging in asymptomatic adults. They can result from either a congenital or acquired weakness of the tracheal wall. TDs are classified into two variants: congenital and acquired. Symptoms may include dysphagia, odynophagia, neck pain, hoarseness, hemoptysis, and infections. High-resolution CT (HRCT) is essential for diagnosis, while fibrobronchoscopy is less effective in demonstrating the connection with the tracheal lumen. Treatment is typically conservative for asymptomatic patients. For symptomatic patients, treatment options may be either surgical or conservative, depending on the patient’s age and overall physical condition.

Case Presentation: We describe two cases of patients under follow-up for COPD: a 57-year-old man and a 66-year-old woman. In both cases, HRCT detected a right paratracheal air-filled mass, suggesting a tracheal diverticulum. The diagnosis was confirmed by fibrobronchoscopy, which identified a diverticulum with a neck communicating with the tracheal lumen, thereby confirming the presence of a tracheal diverticulum in both cases. The patients also presented with symptoms such as hoarseness, persistent cough, and recurrent pulmonary infections.

Conclusions: Tracheal diverticula have an incidence of 2.0% and a prevalence ranging from 0.75% to 8.1%, with 98.5% located in the right posterolateral area and only 1.5% on the left side at the T1–T3 vertebrae level. Although most cases are asymptomatic, diverticula can cause dry cough, pain, dyspnoea, and hoarseness. If untreated, they can lead to repeated and potentially life-threatening infections.

Tracheal diverticulum is a rare clinical condition that, although generally asymptomatic, can cause complications requiring urgent intervention. Treatment options for symptomatic patients vary based on specific patient factors and existing comorbidities, with surgery being considered a viable option in selected cases.

### 1.94. Abstract 94 (Case Report-Poster): Endobronchial Silver Lesion: A Case of Aspergillus-Associated Metaplasia

**Alessandro Capuano ^1^, Roberto Piro ^2^, Matteo Fontana ^2^, Eleonora Casalini ^2^, Leonardo Maselli ^1^, Cristian Locorotondo ^1^ and Nicola Cosimo Facciolongo ^2^**

1. Institute of Respiratory Disease, University of Medicine “Aldo Moro”—Bari, Bari, Italy
2. Pulmonology Unit, Azienda Unità Sanitaria Locale-IRCCS—Reggio Emilia, Reggio Emilia, Italy

Background: Thoracic endoscopy is nowadays a crucial diagnostic technique, even in the field of infectious disease of the bronchial tree, with the possibility of performing targeted biopsies on suspicious lesions useful for microbiological analysis.

Case Presentation: A chest CT scan in a 81-year-old former butcher and heavy smoker, hospitalized for an acute exacerbation of COPD, revealed diffuse centrilobular micro-bullous emphysema, tree-in-bud patterns, and bronchial wall thickening. Following this a flexible bronchoscopy, performed for microbiological investigations, revealed a vegetative, silver-colored lesion at the entrance of the right upper lobar bronchus (Figure 21), alongside another different endobronchial lesion was observed in the bronchus intermedius. 

Both lesions were biopsied using forceps, and the samples sent for microbiological and histopathological analysis.

Histopathological examination of the biopsied samples revealed bronchial mucosa with marked chronic erosive inflammation and squamous metaplasia of the lining epithelium, notably associated with the curious presence of fungal hyphae. Microbiological cultures confirmed a fungal infection, identifying Aspergillus fumigatus as the causative organism. Additionally, fragments of submucosal bronchial leiomyoma were diagnosed in samples taken from the second lesion. Treatment with voriconazole was initiated following the diagnosis of endobronchial metaplasia associated with Aspergillus infection.

Conclusions: This case underscores the unusual appearance of the lesion and the rarity of finding metaplasia associated with fungal hyphae, to our knowledge. We suggest considering Aspergillus in similar cases and speculate on a potential interaction between fungal infection, chronic inflammation and metaplastic changes in the bronchial epithelium. However, further studies are warranted to better understand the underlying pathogenic mechanisms.

### 1.95. Abstract 95 (Case Report-Poster): Vats as Approach of Choice for the Management of Spontaneous Hemopneumotorax: A Case Report

**Graziana Carleo, Ondina Pizzuto, Mirko Girolamo Cantatore, Maria Luisa Zhurda, Francesca Tedone, Naomi Savarelli, Loredana D’Aucelli, Debora Brascia and Angela De Palma**

Thoracic Surgery Unit, Department of Precision and Regenerative Medicine and Ionian Area, University of Bari “Aldo Moro”, Bari, Italy

Background: Spontaneous hemopneumothorax (SHP) is the spontaneous accumulation of both blood and air within the pleural cavity, without any previous medical or trauma history, generally related to the rupture of apical pleuro-pulmonary adhesions and, when not promptly recognized, can represent an emergency situation. We report a case of SHP early identified and successfully treated by video-assisted thoracoscopic surgery (VATS) as approach of choice.

Case Presentation: A 23-year-old Caucasian man, smoker, was referred to the emergency room of a province hospital with left chest pain, resistant to analgesic drugs. Chest X-ray revealed a left spontaneous pneumothorax (Figure 22A), thus the patient was transferred to our Polyclinic where a chest tube (trocar 20 CH) was inserted, showing abundant air leaks. 

After admission to our ward, suction was applied to the pleural drainage, with evidence of air leaks associated to blood leaks (overall 600 mL). Chest X-ray showed an air-fluid level in the left apical paramediastinal region (Figure 22B), thus a chest CT-scan with contrast was performed in emergency, confirming a hemothorax, even if no signs of spreading of contrast were detected (Figure 22C).

Considering the significant chest pain complained by the patient, the persistence of minimal blood loss from the drainage and the slight anemia detected by blood tests, we decided to submit the patient to urgent surgery. 

Through a left triportal VATS approach, a significant amount of blood and clots (about 1000 mL) was found in the pleural cavity. After removal of blood and clots, the source of bleeding was identified to be a ruptured apical pleuro-pulmonary adhesion. The lung apex, at the level of the ruptured adhesion, showed bullous dystrophy, therefore it was resected by stapler (Figure 22D). Hemostasis of the broken adhesion on the side of the pleural dome was performed using electrocautery. Post-operative course was uneventful, chest tubes were removed on the sixth postoperative day and the patient was discharged on the seventh postoperative day. 

Conclusions: This case emphasizes the role of VATS as approach of choice in the management of early diagnosed SHP, in the absence of hemodynamic instability. 

### 1.96. Abstract 96 (Case Report-Poster): Multidisciplinary Approach for Resectable Solitary Lung Nodule in High Risk Patient

**Ilenia Cavaliere ^1^, Daniele Scarascia ^1^, Valeriy Puzhliakov ^1^, Flavio Fedele ^2^, Stefania Infusino ^3^ and Angelo Chirillo ^4^**

1. Division of Thoracic Surgery, Cardio-Thoracic-Vascular Departement, Hospital of Cosenza “SS Annunziata”, Cosenza, Italy
2. Division of Bronchology, Cardio-Thoracic-Vascular Departement, Hospital of Cosenza “SS Annunziata”, Cosenza, Italy
3. Division of Oncology, Onco-Hematology Department, Hospital of Cosenza “SS Annunziata”, Cosenza, Italy
4. Division of Radiotherapy, Onco-Hematology Department, Hospital of Cosenza “SS Annunziata”, Cosenza, Italy

Background: Surgical resection still represents gold standard in the treatment of lung cancer. Here we present multidisciplinary approach for resectable lung nodule in poor lung function patient. 

Case Presentation: In February 2024, a 62 years old male, high smoker, presented to Division of Thoracic Surgery a chest CT scan: RUL nodule suggesting cancer and a LUL vanishing lung (Figure 23). FDG PET scan demonstrated tracer trapping into RUL nodule and did not reveal pulmonary, nodal or extrapulmonary pathology. 

The evaluation of chest disease multidisciplinar team (CD MDT) suggests to execute functional evaluation before contraindicatig surgical treatment.

Spirometry following by DLCO, lung scintigrafy and cardiopulmonary test were performed, showing that LUL tissue did not contribute to lung function, which was globally reduced (Figure 24). 

Consequently, a predictive model was used to calculate theoretical postoperative lung function, which shows that patient could tolerate a minor lung resection. According to CD MDT decision, patient underwent two steps surgery. At first a LUL volume reduction was performed. Operating time was 70 min, no intra and postoperative complications occurred. Chest tube duration was 5 days, postoperative stay was 6 days (Figure 25). 

After about a month a uniportal-VATS RUL wedge resection and mediastinal node sampling. Operating time was 65 min, no intra and postoperative complications occurred. Chest tube duration was 3 days, postoperative stay was 4 days. Histopathology diagnosis was invasive nonmucinous adenocarcinoma T1b N0 PDL1 positive. Patient underwent to clinical follow-up, according to CD MDT decision (Figure 26).

Conclusions: A collaborative approach, appropriately leveraging the different expertise of CD MDT members, allows to ensure optimal per-patient decision making as treatment options become ever more specialized in the current time of developing technologies, techniques and treatment

### 1.97. Abstract 97 (Case Report-Poster): Successful Multidisciplinary Treatment of Aorto-Esophageal Fistula: A Case Report

**Giacomo Cusumano ^1^, Francesco Borrata ^1^, Alessandra Criscione ^1^, Damiano Calvo ^1^, Mariapia Gangemi ^1^, Maurizio Mannino ^1^, Marco Sigona ^1^, Santi Mangiafico ^2^, Pierfrancesco Veroux ^2^ and Alberto Terminella ^1^**

1. Thoracic Surgery Unit, University Hospital-Policlinico-San Marco, Catania, Italy
2. Gastroenterology and Digestive Endoscopy, University Hospital-Policlinico-San Marco, Catania, Italy
3. Vascular surgery Unit, University Hospital-Policlinico-San Marco, Catania, Italy

Background: Aorto-esophageal fistula (AEF) is a rare but typically fatal condition. This report aims to describe a case of AEF successfully treated through a multidisciplinary approach involving endovascular, surgical, and endoscopic techniques, with special emphasis on the value of in situ aortic allograft replacement.

Case Presentation: Our patient, a 59-year-old male with a history of smoking and hypertension, presented to the emergency department with moderate hematemesis. Esophagogastroscopy revealed a clot-covered hole in the middle esophagus. A CT scan promptly identified a primary AEF caused by the rupture of an atherosclerotic aneurysm into the esophagus, accompanied by prodromic hematemesis. The patient exhibited anemia and signs of infection as evidenced by blood tests.

An endovascular aortic stent was placed within 2 h of diagnosis. A nasogastric tube was inserted for aspiration, and the patient was started on total parenteral nutrition, hemocultures, and an antibiotic regimen. Surgical intervention to close the fistula was scheduled.

Seven days after the vascular treatment, with negative hemocultures, the patient underwent surgery. A posterolateral right thoracotomy was performed, revealing that the esophagus was completely fused with the aneurysmal vascular sac for at least 10 cm. The right wall of the esophagus was longitudinally incised, exposing the fistula on the left wall. The aneurysmal sac was cleaned, and the fistula, along with the incision on the right side, was closed with sutures.

Six days later, a small fistula on the left side of the esophagus was identified by a CT scan with oral contrast. Endoscopic treatment was then performed, placing a fully covered esophageal stent. After a 25-day hospital stay, the patient was discharged. Thirty-four days post-discharge, the esophageal stent was removed, and a follow-up contrasted CT scan showed complete resolution of the fistula. The patient remains alive and in good condition.

Conclusions: Our experience confirms that AEF is a rare but potentially catastrophic disorder. Recognizing sentinel bleeding is crucial for the immediate initiation of endovascular treatment, which is a key component in managing this life-threatening condition.

### 1.98. Abstract 98 (Case Report-Poster): Robotic-Assisted Thoracic Surgery for Azygos Vein Ectasia: A Case Report

**Elisabettamaria Frongillo, Mario Sorice, Luigi Busiello, Della Morte Aniello, Emilia Polimeno, Lucia Beneduce, Valbona Telha, Emanuele Russo, Chiara Freda, Antonio Noro and Gianluca Guggino**

U.O.C. di Chirurgia Toracica, A.O.R.N. “A.Cardarelli” Napoli, Italia

Background: Azygos vein ectasia is very rare and its finding is usually incidental. It is classified in idiopathic and acquired (i.e., caused by pulmonary artery hypertension, arterial-venous fistula, pregnancy, cardiac failure and catheter-induced trauma). Most of the ectasias are limited to the arch. They can be either asymptomatic or cause compression symptoms such as dysphagia. Complications include rupture and thromboembolism. There are neither guidelines nor systematic studies assessing the gold standard treatment. Interventional radiology treatment or surgical resection have to be considered if symptomatic, in case of an increase of the ectasia, saccular ectasia and in patients with a connective tissue disease. The most common procedure is surgical ectasia ligation and/or endovascular occlusion. Video-assisted thoracic surgery (VATS) and thoracotomic approach are described in literature. but there is no reference for robotic assisted thoracic surgery (RATS).

Case Presentation: A.A., 74 years old, was referred to our Outpatient Clinic for a 3-years worsening dysphagia. She has no comorbidities, except hip bilateral replacement, causing walking impairment. She underwent EGDS negative, and an angio-CT chest scan, reporting a 3.8 cm max diam azygos ectasia (Figure 27). 

Because of her sympoms, she was admitted for Da Vinci Xi robotic surgery, in left lateral position. After mediastinal pleural dissection, we isolated the azygos vein in its superior cava vein confluence and the supreme intercostal vein (Figure 28). 

We resected several tributaries branches of the azygos arch, then removing the ectasia en bloc with the arch (Figure 29). 

The post-operative hospitalization was free of complications, with an optimal pain control. The patient was discharged in post-operative day 4. She declared the resolution of the dysphagia both during the first post-op examination and during follow-up. 

Conclusions: Due to its accuracy and feasibility, robotic surgery has to be also considered for azygos vein ectasia treatment. 

### 1.99. Abstract 99 (Case Report-Poster): Traumatic Sternoclavicular Joint Posterior Dislocation: A New Fixation System

**Eleonora La Rocca, Federica Mellone, Antonella Alloisio, Ludovica Balsamo, Giacomo Leoncini, Mattia Manitto, Lucia Morelli, Luca Novello, Maria Teresa Piras and Gian Luca Pariscenti**

U.O.C. Chirurgia Toracica, IRCCS Ospedale Policlinico San Martino, Genova

Background: Although clavicle osteosynthesis represents an orthopedic expertise, thoracic surgeon may be involved especially in traumatic sternoclavicular joint posterior dislocation. This condition can be potentially a life-threatening injury as the mediastinum can be involved with possible severe bleeding.

There have been many reported techniques for the stabilization of the joint, including Kirschner wires fixation, hook plates, repair of the sternoclavicular and costoclavicular ligaments, reconstruction of the joint with a tendon or fascial graft, and resection of the clavicle medial end. 

Our team developed a new peculiar fixation system made of differently sized cannulated screws which can be fixed to the surface of interest and through which steel wires or non-absorbable wires can be passed.

This technique allows the osteosynthesis of irregular or curve-shaped surfaces, which can be particularly challenging.

We report two cases of young patients who presented with a traumatic sternoclavicular joint posterior dislocation.

Case Presentation: The first patient was a 15-year-old girl transported to the emergency room of our Centre after high-kinetics car-accident with facial and chest trauma.

No comorbidities or previous medical history were reported. The patient underwent an initial plastic reconstructive surgery for avulsion of face soft tissues. CT scan highlighted posterior dislocation of the right clavicle at the sternoclavicular joint with anterior mediastinal hematoma and vascular compression, without active bleeding. Two days after, with previous anesthesiologic and ENT clearance to nasal intubation, we performed a reduction of dislocation with positioning of sternal and clavicular cannulated screws (respectively 14 mm and 16 mm) and fixation steel wires plus non absorbable wires (Ti-Cron 5) for reconstruction of the sternoclavicular joint. Correct stabilization of the clavicle with the maintenance of mobility at the sternoclavicular level was verified intraoperatively with arm movements. The second patient was a 17-year-old boy who was involved in a high-kinetics motocross accident with chest trauma. CT scan demonstrated posterior dislocation of the sternoclavicular joint with vascular compression, without active bleeding. We performed the reduction of dislocation with the same technique. Both patients underwent postoperative motion physiotherapy with progressive recovery of full mobility of the right upper limb, without complication.

Conclusions: The presented technique allowed simple and effective repair of the sternoclavicular joint, with good functional outcomes. Cannulated screws may also be implemented in the future for the osteosynthesis of sternal fractures, the repair of which still appears problematic and not standardized.

### 1.100. Abstract 100 (Case Report-Poster): D-Dimer Elevation and Lymphangioleiomyomatosis: A Case Report

**Antonio Lamesta ^1^, Noemi Camarda ^1^, Ciro Ruggiero ^2^, Christian Casali ^2^ and Pier Luigi Filosso ^1^**

1. University of Modena and Reggio Emilia
2. Thoracic Surgery Unit, Baggiovara Hospital of Modena, Modena, Italy

Background: Lymphangioleiomyomatosis (LAM) is a rare lung disease characterized by diffuse cystic changes caused by a destructive proliferation of smooth muscle-like cells. 

Case Presentation: We report a case of a 56-year-old woman who presented with spontaneous pneumothorax and d-dimer elevation. Computed tomography pulmonary angiography (CTPA) scans of the chest and limbs doppler ultrasound ruled out pulmonary embolism and venous thrombosis. LAM diagnosis was made based on lung biopsies. According to the literature, the combination of d-dimer elevation and LAM is unusual. We monitored d-dimer levels long after surgical intervention and noted that it not only remained high even after the acute phase but it increased gradually, reaching higher values than the initial data available. The patient’s comorbidities did not justify such an increase, and during these time-distance controls from the episode, the patient was asymptomatic, eupnoeic in room air.

Conclusions: We attempted to understand if a correlation between LAM and elevated d-dimer levels was possible. To do this, we analyzed the current pathophysiological knowledge of LAM and the mechanisms of d-dimer formation in the bloodstream. According to our primary hypothesis, vascular endothelial growth factor D (VEGF-D), a glycoprotein produced by LAM cells, could lead to the formation of cross-linked fibrin deposits in the pulmonary microcirculation. In some cases, the quantity of fibrin microclots could be such as to stimulate Glu-plasminogen activation, resulting in the formation of Glu-plasmin. Glu plasmin dissolves the microclots, serving as a source of d-dimer.

### 1.101. Abstract 101 (Case Report-Poster): Intravascular Tumor Invasion Mimicking Pulmonary Embolism: A Case Study Highlighting the Role of Ebus

**Ernesto Lulaj ^1^, Giuseppe Antonio Palmiotti ^2^, Cristian Locorotondo ^1^, Armando Leone ^2^, Giancarlo D’Alagni ^2^ and Giovanna Elisiana Carpagnano ^1^**

1. U.O.C. Malattie apparato respiratorio, Policlinico di Bari, Bari, Italia
2. U.O.C. Pneumologia, Ospedale San Giuseppe Moscati, Taranto, Italia

Background: Tumors invading the pulmonary arteries, such as angiosarcoma and metastatic embolizations from other organs, though rare, carry significant clinical implications. Primary lung cancers can macroscopically extend into the pulmonary arteries, making intravascular tumors a crucial differential diagnosis for pulmonary thromboembolism (PE). The primary diagnostic tool, computed tomography (CT) with intravenous contrast (angio-CT), cannot definitively distinguish between tumor-related and non-tumor embolic lesions.

Case Presentation: A 53-year-old male smoker with a history of chronic exposure to dust and chemicals presented with dyspnea, cough, and hemoptysis. Thoracic CT revealed a heteroplastic lesion in the apical segment of the right upper lobe, partially infiltrating the upper mediastinum, characterized by spiculated margins and encasing peripheral broncho-vascular bundles. Significant lymphadenopathy was observed in the 4R station (Figure 30).

CT also detected a unilateral proximal PE contiguous to the primary pulmonary lesion (Figure 31). 

Bronchoscopy with EBUS-transbronchial needle aspiration (TBNA) of the 4R station was performed. The EBUS probe, placed at the anterior wall of the main bronchus, visualized the right pulmonary artery with a round, centimeter-sized lesion in continuity with the vascular wall and surrounded by blood flow (Figure 32). 

This lesion was not punctured; however, based on its endosonographic characteristics, it appeared to be in continuity with the pulmonary mass, leading us to believe that what was initially reported as an embolism is an intravascular invasion of the tumor.

Conclusions: Pulmonary artery invasion by tumors, often misdiagnosed as PE, can lead to inappropriate treatments like prolonged anticoagulation or thrombolysis and delay surgical intervention. Therefore, differentiating pulmonary artery tumors from thromboembolisms is crucial. PET/CT helps confirm PE suspicion and differentiates between pulmonary artery tumors (PATs) but is not always available. EBUS facilitates this differentiation by assessing the contiguity and varying echogenicity, strain, and vascular patterns of the main lesion and intravascular mass. Typically, pulmonary arteries appear echo-free; emboli manifest as echogenic masses within the lumen, detectable by power- mode doppler of the EBUS bronchoscope. EBUS improves visualization of tumors and vascular flow, aiding early and accurate PAT diagnosis. TBNA sampling of endovascular lesions is feasible and safe, supported by routine use of transvascular access with small gauge needles in radiological and interventional procedures. 

We emphasize the importance of considering tumor invasion in the differential diagnosis of PE to ensure timely and appropriate treatment. Incorporating vascular evaluation during EBUS is essential for systematic diagnosis, especially when vascular invasion and concomitant PE can alter therapeutic management.

### 1.102. Abstract 102 (Case Report-Poster): Flu-like Syndrome-Induced Acute Asthma Exacerbation Complicated by Pneumomediastinum, Subcutaneous Emphysema, and Pneumothorax

**Guido Marchi ^1^, Giacomo Guglielmi ^2^ Luciano Gabbrielli ^1^, Francesco Pistelli ^2^ and Laura Carrozzi ^2^**

1. Pulmonary Unit, Cardiothoracic and Vascular Department, Pisa University Hospital—Pisa Italy
2. Department of Surgical, Medical, and Molecular Pathology and Critical Care Medicine, University of Pisa—Pisa Italy

Background: Flu-like syndromes can trigger acute asthma exacerbations (AAE), and rarely lead to spontaneous pneumomediastinum (SPM). SPM involves the accumulation of air in the mediastinum due to increased intra-alveolar pressure, which is typically resolved with supportive therapy, though severe cases may require invasive interventions. It can also coincide with other forms of extra-alveolar air, such as pneumothorax (PTX), subcutaneous emphysema (SE), pneumopericardium, and pneumoperitoneum. Predisposing factors include elevated airway pressure, intrinsic lung diseases like asthma, smoking, drug abuse, and exposure to irritants. We present a case of a young patient experiencing an AAE triggered by a flu-like illness, complicated by SPM, SE, and PTX.

Case Presentation: A 27-year-old man with a history of bronchial asthma, allergic rhinitis, occasional marijuana use, and inconsistent adherence to inhaler therapy, presented to the emergency department with flu-like symptoms, including fever, myalgia, headache, rhinitis, dyspnoea, and dry cough. Laboratory tests revealed elevated inflammatory markers, and arterial blood gas analysis indicated type 1 respiratory failure. Initial chest X-ray showed no pleural-parenchymal abnormalities. Further evaluation during the emergency department admission revealed worsening dyspnoea, change in voice timbre, and severe SE of the neck. Subsequent chest CT scan confirmed the presence of severe pneumomediastinum, SE, small right PTX, ground glass opacities, and irregular lung consolidations (Figure 33). 

Prompt treatment was initiated with intravenous steroids, bronchodilators, antibiotics, and oxygen therapy. During hospitalization, the patient adhered to strict bed rest and showed rapid clinical improvement, with complete reabsorption of SE. Serial chest X-rays showed progressive resolution of findings. Upon discharge, the patient was in good clinical condition and breathing ambient air. He received regular medical therapy for bronchial asthma and was advised to discontinue marijuana use, while also being instructed to follow up regularly.

Conclusions: SPM can rarely occur as a complication of AAE. While usually benign and self-limiting, its occurrence alongside PTX can lead to acute airway compromise and respiratory failure in severe cases of AAE. Clinicians should maintain a high index of suspicion for cases showing atypical features, significant oxygen demand, or lack of improvement with conventional therapies. Management includes vigilant monitoring, rest, analgesia, oxygen supplementation, bronchodilators, and appropriate antibiotics. This case is presented to raise awareness among healthcare providers about this severe and uncommon complication associated with AAE. We highlight the potential effectiveness of conservative management, which can lead to rapid and complete recovery. It’s essential to acknowledge that every AAE carries the potential for life-threatening outcomes, requiring prompt and comprehensive treatment.

### 1.103. Abstract 103 (Case Report-Poster): Benign Bronchial Tumors: A Rare Case of Endobronchial Fibroma Associated with Ilo-Mediastinal Lymphadenopathy

**Giuseppina Marrazzo ^1^, Teresa Ferrazzo ^2^, Giuseppe Failla ^3^, Tropea Francesco ^2^, Nicola Montenegro ^2^, Gianluca Ippolito ^2^, Fabio Alfredo Nania ^4^, Carlo Gentile ^5^, Annamaria Lavecchia ^5^, Corrado Pelaia ^1,6^ and Girolamo Pelaia ^1,2^**

1. Pulmonology Unit, “Renato Dulbecco” University Hospital, Catanzaro, Italy
2. Department of Health Sciences, University “Magna Græcia” of Catanzaro, Catanzaro, Italy
3. Diagnostic and Therapeutic Bronchoscopy Unit, ARNAS “Civico e Benfratelli”, Palermo, Italy
4. Anesthesia and Intensive Care Unit, “Santa Maria Goretti” Hospital, Latina, Italy
5. Pathology Unit, “Renato Dulbecco” University Hospital, Catanzaro, Italy
6. Department of Medical and Surgical Sciences University “Magna Græcia” of Catanzaro Catanzaro, Italy

Background: Endobronchial fibromas are sporadic benign tumors that can cause local invasion of the airways. 

Case Presentation: We present a case of a 73-year-old man with endobronchial fibroma and hilar-mediastinal lymphadenopathy diagnosed during radiological follow-up for pulmonary nodules, with an individual risk of developing cancer due to both family predisposition and environmental exposure.

The control chest computed tomography revealed pulmonary thickening in the middle lobe and the appearance of mediastinal lymphadenopathy; a bronchoscopy was performed, which highlighted a vegetative growth with a smooth surface and hard-elastic consistency in the lateral segmental bronchus (B4) of the middle lobar bronchus. Fine needle aspiration (FNA) of the endobronchial lesion and EndoBronchial UltraSound-guided TransBronchial Needle Aspiration (EBUS-TBNA) of the mediastinal lymphadenopathies were performed.

The patient subsequently underwent rigid bronchoscopy with forceps and laser excision of the endobronchial vegetating lesion, which caused sub-occlusion of the segmental bronchus B4.

The histological examination of the FNA sample and the entire removed lesion was positive for a fibromatous lesion. On the other hand, the lymph node histological examination was positive for an inflammatory lesion.

A double diagnostic investigation was carried out in the case presented, considering the high neoplastic risk.

### 1.104. Abstract 104 (Case Report-Poster): Bronchial Termoplasty Can Lead an Incredible Clinical and Functional Improvement

**Leonardo Maselli ^1^ MD, Alessandro Capuano ^1^ MD, Ernesto Lulaj ^1^ MD, Esterina Boniello ^1^ MD, Roberto Piro ^2^ MD, Eleonora Casalini ^2^ MD, Matteo Fontana ^2^ MD and Nicola Cosimo Facciolongo ^2^ MD**

1. Institute of Respiratory Disease, University of Medicine “Aldo Moro”—Bari, Bari, Italy
2. Pulmonology Unit, Azienda Unità Sanitaria Locale-IRCCS—Reggio Emilia, Reggio Emilia, Italy

Background: Bronchial thermoplasty (BT) is a non-pharmacological endoscopic treatment for patients with uncontrolled severe asthma despite maximum inhalation therapy and continuous use of oral steroids. BT consists in the controlled release of radiofrequency thermal energy on the walls of the airways with the consequent reduction in the amount of the smooth muscles and improvement in asthma control.

Case Presentation: An 80 year-old patient suffering from severe asthma for 20 years with a T2-low inflammatory pattern was taking significant doses of oral steroids due to poor symptomatic control, which complicated her arterial hypertension and osteoporosis. 

Pulmonary function tests showed poor functional reserve with severe obstructive deficit (FEV1 31%), while FeNO test was in range but affected by the intake of steroids. Clinical control was also poor (ACT 7/25), and the patient complained of not carrying out daily activities despite maximum adherence to inhalation and antileukotriene therapy, in the impossibility of prescribing monoclonal target therapy. BT was recommended to reduce the dosage of oral steroid taken and in the hope of having a better clinical and functional control of severe asthma. So, three cycles of BT were performed and immediately after the conclusion of the third cycle there was an incredible increase of FEV1 (80%), a clear clinical improvement and the total absence of oral steroids intake. The only data that went against the trend was the increase in FeNO 50 mL/s (32 ppb), but probably linked to the bronchial inflammatory process due to BT. Currently the patient shows continuous clinical improvement, resumption of physical activity and step down of inhalation therapy with ICS/LABA.

Conclusions: We speculate that the late onset of asthma coincides with less airway remodelling, and this facilitates the successful outcome of BT.

In recent years the role of lung function among the selection criteria for BT has been reduced; recent evidences suggest that the procedure is safe and effective even for patients with FEV1 between 30% and 50%. In our case, BT leads to extraordinary results, suggesting that this therapeutic option should be given greater consideration, especially for severe asthma patients not who are not candidate to monoclonal target therapies or with poor control despite the maximum available therapy.

Further comparative studies, especially with emerging monoclonal therapies for T2-low asthma are needed.

### 1.105. Abstract 105 (Case Report-Poster): Surgical Treatment in Persistent Chylotorax: A Case Report

**Federica Mellone, Eleonora La Rocca, Giacomo Leoncini, Antonella Alloisio, Ludovica Balsamo, Mattia Manitto, Lucia Morelli, Luca Novello, Maria Teresa Piras and Gian Luca Pariscenti**

UOC Chirurgia Toracica, IRCCS Ospedale Policlinico San Martino, Genova

Background: Chylothorax is defined as a thoracic duct (or one of its tributaries) chylous leak in the pleural space. Chylothorax treatment is only partially standardized and it depends on the cause of the altered chylo flow. It can be due to congenital, traumatic (iatrogenic 80%, non-iatrogenic 20%), neoplastic or miscellaneous causes. This case report is about the surgical treatment of a non-iatrogenic chylothorax.

Case Presentation: Male, 62 years old, previous smoker, without relevant comorbidities. Significant weight loss in previous 3 months, sudden abdominal lymphorrea. Total body CT scan and PET-CT scan highlighted bilateral pleural and pelvic effusion and abdominal subcutaneous thickening. Subsequent diagnosis of abdominal lymphangioma was obtained by subcutaneous tissue biopsy. Multiple thoracentesis’ have been performed in another hospital, without clinical relief. Evacuated pleural fluid had a milky appearance. Triglycerides in it were 375 mg/dL. The patient has been accepted to our department and underwent fasting and complete endovenous nutrition. A 28 Ch chest drain was positioned on the left pleural space and another one in the right pleural space. Bilateral daily profuse pleural effusion drained out from chest tubes, without any benefit form fasting. The patient underwent a first attempt of lymphography via inguinal access with Lipiodol injection (20 mL) with diagnostic e therapeutic purpose. The procedure was not successful in highlighting lymphatic system. A second attempt of lymphography was conducted with a retrograde access (via basilic left vein): this time it was obtained the complete lymphatic system view, but no Lipiodol leaks were find out. Because of the persistent copious bilateral pleural fluid loss, we decided to perform a surgical closure of the thoracic duct. The procedure was conducted with a right uniportal videothoracoscopic access; the thoracic duct was ligated in its onset in pleural space with 2 non-resorbable stitches and metal clips. Moreover, chemical pleurodesis with 6 gr of sterile talc was carried out. Post operative course was regular with progressive resolution of pleural effusion bilaterally. After oral food intake reintroduction, there was no occurrence of pleural effusion bilaterally or abdominal lymphorrhea. Clinical and radiological follow-up two months later demonstrated resolution of the pleural and abdominal picture after surgery.

Conclusions: Videothoracoscopic surgical ligation of the thoracic duct may be a reasonable alternative in the treatment of chylothorax secondary to abdominal lymphangioma, and chemical pleurodesis may be indicated in cases of failure or recurrence after lymphoma.

### 1.106. Abstract 106 (Case Report-Poster): Surgical Treatment of Giant Solitary Fibrous Tumor of the Pleura: A Case Report

**Federica Mellone, Eleonora La Rocca, Mattia Manitto, Antonella Alloisio, Ludovica Balsamo, Giacomo Leoncini, Lucia Morelli, Luca Novello, Maria Teresa Piras and Gian Luca Pariscenti**

UOC Chirurgia Toracica, IRCCS Ospedale Policlinico San Martino, Genova

Background: Solitary fibrous tumour (SFT) is a rare, mesenchymal neoplasm that usually arises in the pleura, but rarely involves other sites outside the serosal space. Histologically, SFT malignancy is defined by: mitotic count with more than 4 mitosis/10 high power fields (HPF) (×400), presence of necrosis, hyper cellularity as judged by nuclear crowding and overlapping, presence of nuclear atypia. Such tumors are generally slow-growing, with a low risk of metastasis and asymptomatic until they exert compressive effects on adjacent organs. Diagnosis of malignancy is histologically determined after surgical resection and the clinical behaviour of single tumors is notoriously difficult to predict.

Case Presentation: We present a rare case of a 67-year-old female patient with no previous pulmonary history who presented with progressive dyspnoea and fatigue. On CT scan, she was found to have a 15 cm × 19 cm × 18 cm (LL × AP × CC) bulky mass occupying the right hemithorax with associated compressive atelectasis of the lung. In addition, there was mild compression of the inferior vena cava with consequent edema of the lower limbs and deviation of mediastinal structures without apparent infiltration. Pleural mass determined asymptomatic high level of carbon dioxide (pCO2 60). She underwent a computed tomography guided biopsy that revealed the mass to be a solitary fibrous tumor. Aortography was performed the day before surgery and four arterial branches were embolized with microparticles (800 micron) and metal spirals: right diaphragmatic artery (directly from left gastric artery), and three intercostal arteries (D9, D10, D11). These vessels combined with the pericardiophrenic artery provided tumor vascularization, but the pericardiophrenic one had not been embolized. The patient underwent right posterior thoracotomy with complete resection of the tumor. The lesion’s weight was 2.91 kg. There were no intraoperative complications and intraoperative blood loss was about 400 mL. At the end of the procedure, the right lung was fully expanded. The patient had no post-operative complications. The patient was discharged on postoperative day 6. 

Conclusions: These tumours cause few symptoms which are usually diagnosed as a casual finding when a chest X-ray is performed on account of other cause. Treatment should be surgical, with removal of all tumoral mass. In addition, preoperative embolization is recommended to minimize the risk of intraoperative bleeding and time of surgery. Embolization performed the day before surgery is enough to avoid intraoperative bleeding.

### 1.107. Abstract 107 (Case Report-Poster): Mesothelioma after Lung Transplantation: A Very Rare Late Complication

**Maddalena Messina ^1^, Chiara Catelli ^2^, Luca Luzzi ^2^, Piero Paladini ^2^, Elena Bargagli ^1^, Antonella Fossi ^1^ and David Bennett ^1^**

1. Respiratory Diseases Unit, Department of Medical and Surgical Sciences and Neuro-sciences, University of Siena, 53100 Siena, Italy
2. Thoracic Surgery, University Hospital of Siena (Azienda Ospedaliera Universitaria Senese, AOUS), 53100 Siena, Italy

Background: we presented a case of a lung transplant recipient who developed malignant mesothelioma on the native lung. Malignancies are the second cause of death, after bronchiolitis obliterans syndrome (BOS), in the long-term follow-up of lung transplant recipients. 

Case Presentation: we present the clinical course of a 50 year-old male, who underwent single lung right transplantation for idiopathic pulmonary fibrosis in January 2006. 

A 50 year-old male, former school teacher, non-smoker, underwent single lung right transplantation for idiopathic pulmonary fibrosis in January 2006. The immunosuppressive therapy consisted of prednisone, cyclosporine and azathioprine. In 2009 worsening of respiratory condition and pulmonary function tests was observed and diagnosis of BOS was made. The immunosuppressive regimen was modified, cyclosporine was switched to tacrolimus and azathioprine to mycophenolate mofetil and azithromycin was also introduced. In 2010 mycophenolate we switched to everolimus with a complete stabilization of respiratory function (FEV1 57% of predicted). In November 2022, the patient developed massive hemorrhagic left pleural effusion. A CT scan of the chest showed a multiple thickening mainly macronodular and ubiquitous distribution of the left pleura, with extensive centrally necrotic tissue plaque of the cost-phrenic breakthrough adjacent to the antero-lateral insertion of the diaphragm. Bronchoscopy with bronchoalveolar lavage did not show evidence of infection or malignancy. Robotic thoracoscopy revealed widespread pleural thickening and multiple pleural biopsy specimens were obtained. Histological examination showed diffuse infiltration of the pleura by biphasic mesothelioma with desmoplastic component (60%) and high-grade epithelioid component (40%) with modest inflammatory lymphoplasmacellular infiltrate. BAP1 expression evaluation resulted positive. A systemic chemotherapy approach was adopted with Pemetrexed and Cisplatin. Despite that the disease progressed and in March 2023 unfortunately the patient died.

Conclusions: pleural mesothelioma is a rare malignant tumor directly attributable to asbestos exposure, however it can also present in cases, as our here presented, with no documented exposure [23,24]. It has been reported that the overall risk for malignancy is 2 to 4 times higher after solid organ transplantation than general population [25,26]. Post-transplant tumors are mainly associated with the use of chronic immunosuppressive regimen, impaired immunosurveillance and subsequent oncogenic viral infections [25,27]. Despite literature review we only found one similar case of pleural mesothelioma onset, without asbestos exposure, after single lung transplantation in the native fibrotic lung [28]. Further research is needed in order to better understand mesothelioma epidemiology and pathogenesis in lung transplant recipients.

### 1.108. Abstract 108 (Case Report-Poster): Uncommon Mediastinal Mass Involving the Heart

**Klodjana Mucaj ^1,2^, Laura Saracino ^2^, Stefano Tomaselli ^2^, Alessandro Cascina ^2^, Matteo Bosio ^2^, Francesco Rocco Bertuccio ^2^, Gioacchino D’Ambrosio ^3^ and Angelo Guido Corsico ^1,2^**

1. Department of Internal Medicine and Medical Therapeutics, University of Pavia, Italy
2. Cardiothoracic and Vascular Department, Unit of Respiratory Diseases, IRCCS Policlinico San Matteo, Pavia, Italy
3. Pathology Unit, Department of Diagnostical Services and Imaging, IRCCS Policlinico San Matteo, Pavia, Italy

Case presentation: This case involved a 78-years-old Caucasian man, former smoker. He developed symptoms such as fever, cough, dyspnoea, and mild hemoptysis. His previous medical history included pulmonary emphysema, left nephrectomy for renal carcinoma (2012), paroxysmal atrial fibrillation, arterial hypertension. 

A chest X-ray showed an extensive right lung consolidation, confirmed by thorax CT scan that revealed also the presence of a mediastinal round lesion in continuity with an endoluminal formation in the left atrium of the heart. The patient was admitted to the General Medicine Unit and, after improvements of his infectious conditions, he was transferred to our Hospital to continue the diagnostic process. A new CT scan evidenced that the large right pulmonary consolidation was almost resolved. The complete CT staging extended to brain and abdomen did not detect significant lesions. The diagnostic hypotheses were: primitive cardiac tumor, lymphoproliferative disease, other mediastinal lesion, metastasis from a solid tumor. The patient underwent bronchoscopy with linear probe EBUS, confirming a lesion with a homogeneous, richly vascularized structure visualized from the lower right hilar region. The visible portion measured approximately 3 cm. Exploration of the main mediastinal and hilar lymph node stations did not reveal any significant lymph nodes, save for a small one (short axis 6 mm) that was visualized in the 4R position, with ultrasound features of benignity. 

Ultrasound-guided transbronchial needle aspiration (EBUS-TBNA) of the mediastinal lesion was performed using a crown-cut needle 22 G (Boston Acquire™ Pulmonary, Malborough, MA, USA). The obtained material was completely placed in formalin as cell block. The procedure was conducted without complications. 

Cytological examination revealed proliferation of cells with eosinophilic cytoplasm and anisometric nuclei. Immunohistochemistry analysis showed positivity for CKAE1/AE3, PAX8, CD10, CAIX, while TTF1 was negative. The findings suggested a mediastinal metastasis of the previously resected renal cell carcinoma (RCC). In consideration of both the localization of the recurrence site and the infiltration of heart structures, a surgical loco-regional approach as well as radiotherapy were excluded; hence, the patient will undergo a systemic treatment with a multikinase TKI.

Conclusions: RCC rarely shows solitary mediastinal lymph node metastases in absence of lung ones. In patients with mediastinal lesions and past history of RCC is fundamental to distinguish metastatic recurrence from other causes. This is, to our knowledge, the first described case of RCC mediastinal metastasis involving the heart. 

Sampling of hypervascularized lesions performed through linear EBUS-TBNA is feasible, safe and has elevated diagnostic yield. 

### 1.109. Abstract 109 (Case Report-Poster): Pulmonary Inflammatory Pseudotumor in Differential Diagnosis of Lung Cancer: A Case-Report

**Samanta Nicosia, Paraskevas Lyberis and Enrico Ruffini**

A.O.U. Città della salute e della Scienza di Torino

Background: The pulmonary inflammatory pseudotumor (PIP) is a relatively rare disease with a reported incidence between 0.04 to 1.2% of all lung cancers. It is still debated if it represents a non neoplastic lesion characterized by inflammation with uncontrolled cell growth or a true benign or low-grade malignancy. PIP is more common in young adults and does not show sex predilection. PIP should be considered as part of the differential diagnosis of primary or secondary lung cancer.

Case Presentation: In October 2023, a 35 years old man referred to our centre for left paravertebral pleuro-pulmonary lesion (50 mm diameter) highly suspected for invasive pleuro-pulmonary secondary to aggressive osteosarcoma treated with amputation of the right leg in 2009. He performed total-body computed tomography (TC) with no other lesions and positron emission tomography (PET) resulted positive (Figure 34). 

Trans-thoracic biopsy gave uncertain results so surgery became mandatory for the diagnosis.

Patient underwent left thoracoscopy and exeresis of the lesion originating by the lower lobe, partial pleurectomy for the presence of pleural nodules and lymph node picking. No postoperative complications occurred. Pathological anatomy examination showed a PIP on the lung sample, flogosis on pleura and lymph node and no genetic mutations.

Conclusions: The true incidence of PIP is unclear. Preoperative diagnosis is difficult to reach. Whenever possible, surgery should be considered the gold standard and is useful for both treatment and diagnosis. Complete surgical resection is essential to prevent recurrence. Despite the strong diagnostic suspicion based on clinical and radiological data, a diagnosis on a surgical sample is essential to define a therapeutic process and the prognosis of the patient. Prognosis after surgical resection of PIP is usually excellent. Long-term follow-up is still required due to the possibility of local or distant recurrence. 

### 1.110. Abstract 110 (Case Report-Poster): Bilateral Spontaneous Haemothorax: A Case of Primary Pleural Angiosarcoma and Literature Review of a Rare Disease

**Daniel Piamonti ^1^, Silvia Giannone ^1^, Federico Pasqualotto ^1^, Letizia D’Antoni ^1^, Arianna Sanna ^1^, Nicholas Landini ^2^, Angelina Pernazza ^3^, Massimiliano Bassi ^4^, Carolina Carillo ^4^, Federico Venuta ^4^, Paolo Graziano ^3^, Matteo Bonini ^1^ and Paolo Palange ^1^**

1. Department of Public Health and Infectious Diseases, Sapienza University of Rome, Italy
2. Department of Radiology, Oncology and Pathology, Sapienza University of Rome, Italy
3. Department of Radiology, Oncology and Pathology, Sapienza University of Rome, Italy
4. Department of Thoracic Surgery and Lung Transplantation, Sapienza University of Rome, Italy

Background: Angiosarcomas, rare soft tissue malignancies originating from endothelial cells, represent only 1–2% of all soft tissue sarcomas, with skin lesions being the most common presentation. Primary pleural angiosarcoma (PPA) is exceptionally rare, with only 43 reported cases since 1943. Diagnostic and therapeutic challenges arise from delayed identification and limited understanding of optimal treatment strategies due to the rarity of these tumors. 

Case Presentation: We present the case of a 72-year-old man with a medical history of hypertension, spinal cord ischemia and endovascular abdominal aortic aneurysm repair, presenting with back pain, dyspnoea and anemia. He was a former smoker and had been exposed to asbestos. Conventional imaging revealed bilateral pleural effusion and a thickened parietal pleura, while contrast chest MR was able to identify pleural sites of contrast enhancement. Left chest tube placement evidenced a haemothorax and cytology resulted negative. For its complexity and severity, the case was discussed in an internal multidisciplinary setting and on “Pleural-HUB”, a Facebook platform that connects many Italian and international physicians, discussing mainly about pleural pathology. A thoracoscopic approach was chosen allowing to perform different parietal pleural biopsies. Radiological and pathological features (Figure 35) led to the diagnosis of epithelioid PPA.

Despite repeated bilateral pleural drainage and blood transfusions, the patient died only 4 days after diagnosis.

We reviewed cases of PPA in literature (1954—2024) searching the PubMed database for the terms “pleural angiosarcoma” and “pleura + angiosarcoma”. 

We found a total of 49 cases described between 1987 and May 2024, 46 of which with sufficient data to be included in our review. Three cases were excluded because of insufficient data. Main results are described in Table 15. 

Conclusions: PPA predominantly affects adult males, with nonspecific symptoms often overlapping with other thoracic malignancies. Management of recurrent haemothorax and anemia can be needed, and early thoracoscopy with biopsy is crucial for accurate diagnosis. The complexity of diagnosing and treating PPA was evident in the presented case, showcasing the importance of innovative approaches like MRI, described for the first time in this case, and emphasizing the significance of multidisciplinary collaboration for optimal patient management. Bilateral spontaneous haemothorax, as seen in this case, is uncommon and poses additional challenges in disease management. Further research to advance diagnostic capabilities and treatment efficacy is needed.

### 1.111. Abstract 111 (Case Report-Poster): A Patient Who Has Only One Lung Experiences Severe Hypercapnia: How the Use of Hfnc Has Had a Positive Impact on Patient Life: A Case Report

**Silvia Piccirillo ^1^, Miriam Schirò ^1^, Jessica Esposito ^1^, Valeria Spugnardi ^2^ and Luca Notizia ^1^**

1. U.O.C. Pneumologia—”San Giovanni di Dio e Ruggi D’Aragona” University Hospital, Salerno, Italy
2. U.O.C Internal Medicine—”San Giovanni di Dio e Ruggi D’Aragona” University Hospital, Cava de’ Tirreni, Italy

Background: The literature does not contain any examples of patients with only one lung who were treated with High Flow Nasal Cannula (HFNC) after identifying hypoxemic and hypercapnic respiratory failure secondary to pneumonia. Our approach is showcased in this case report.

Case Presentation: A 69 years-old male COPD associated, who had already suffered a post-traumatic exeresis from an accident at work, went to the ER of our Hospital to severe dyspnoea. At the CT of the thorax, an extensive consolidation was found in the left basal region of a likely phlogistic nature. Arterial blood gas analysis revealed severe hypercapnia associated with severe hypoxemia. So, the patient was admitted to the Emergency Medicine, where non-invasive mechanical ventilation (NIV) + OLT was administered for the management of hypoxemia and severe hypercapnia. We were contacted for a transfer to the Pulmonology Department. In our department, a bronchoscopy was performed, which documented the presence of abundant dense secretions Figure 36). 

A new antibiotic therapy was also initiated. The patient was also placed on High-Flow Nasal Cannula (HFNC) with the following parameters: temperature 34 °C, flow rate 60 L/min, FiO_2_ 28%. The decision to use HFNC was considered for better secretion management to improved mucociliary clearance and prevent the formation of mucus plugs, which is crucial in a patient whose ventilation system is reliant on a single lung. The use of HFNC was beneficial like CO_2_ wash-out effect, positive pressure in the airways (PEEP), and humidified gas mixtures. During hospitalization, the patient adapted to this system, and a progressive improvement in clinical, laboratory, and radiological conditions was observed.

Thirty days after there was a significant reduction in blood CO_2_ levels, from 59 to 51 mmHg. These results were confirmed at the subsequent follow-up 90 days after discharge. 

Conclusions: Aware that the chronic use of HFNC is still under study, we believe it could be a viable solution for patients with global chronic respiratory failure in patients with stable COPD.

### 1.112. Abstract 112 (Case Report-Poster): Late Tumor Endobronchial Recurrence Mimicking Copd Exacerbation

**Elena Pordon ^1^, Magda Viani ^1^, Vittoria Ventura ^1^ and Claudia Ghiribelli ^2^**

1. UOC Respiratory diseases, Medical, Surgical and Neurochirigical Sciences—University of Siena
2. UOSA Diagnostic and interventional Bronchoscopy, Siena

Background: Endobronchial metastases from extrathoracic malignancies are rare (2–28%). Diagnosis is often difficult because it becomes necessary to differentiate from primary bronchogenic carcinoma. Moreover, the time between the primary tumour diagnosis and appearance of endobronchial metastasis is approximately 9 months–5 years. Local treatments allow substantial improvement of pulmonary symptoms.

Case Presentation: Here we discuss the case of an 82-year-old white man who previously received colon adenocarcinoma diagnosis in 2014 (KRAS mutation), surgically treated with hemicolectomy. He had two tumour recurrences in 2018 and 2020 treated with metastasectomy in the left upper lobe and a third one in right lower lobe (2021) treated with radiotherapy and chemotherapy (still ongoing). In his personal history, he suffers from COPD (former heavy smoker). The patient arrived at our emergency room department after two months of increasing dyspnoea and a recent occurrence of cough with mucus and rare blood traces. No tirage. At chest high-resolution CT, a new periscissural cavitation in left upper lobe appeared. It was suspected of ongoing inflammation process. At physical examination, we found tracheal secretions accompanied by expiratory rhonchi and wheezes at auscultation mimicking a COPD exacerbation. We performed a fibrobronchoscopy and it was impossible to proceed over the medium trait of the trachea due to a huge protruding lesion so an emergency unblocking (laser and forceps) rigid bronchoscopy was performed. Histology was positive for colorectal metastasis. At control after rigid procedure the patient is stable and his airways are open.

Conclusions: As far as we know there are only few cases in literature of respiratory exacerbation symptoms masquerading endobronchial metastases due to a ball valve effect. It generally involves younger patients (55–70 years) and comes previously (within 5 years from first cancer diagnosis). Although it implies a negative prognosis regarding life expectancy, it did not seem to significantly reduce survival time. There are no established criteria for management in patients with tracheal metastasis (airway stents, bronchoscopic extraction, laser ablation, and radiation therapy), the primary step is to secure the airway.

### 1.113. Abstract 113 (Case Report-Poster): Challenging Tracheal Resection Anastomosis with Ecmo Support

**Ilaria Potenza, Nicola Tamburini, Francesco Dolcetti, Francesco Quarantotto, Pio Maniscalco and Giampiero Dolci**

Arcispedale Sant’Anna, Ferrara, Italy

Background: This case study was conducted to review our experience in Arcispedale Sant’Anna, Ferrara, in the management of high-risk patients who underwent tracheal resection due to a complex tracheal stenosis in history of HBV-related nasopharyngeal carcinoma.

Case Presentation: The case study includes a patient with severe tracheal stenosis. This 48 year-old man is considered a high risk patient due to the etiology of stenosis and associated surgical field morbidity. In order both to maintain the patient’s oxygen supply during surgery and to reduce the size of the surgical incision, we have integrated the ECMO support, perfectly achieving tracheal resection anastomosis. By eliminating the need to constantly reposition the endotracheal tube with ECMO support, visualization and tracheal resection anastomosis outcomes are improved. The patient returned to the intensive care unit and recovered smoothly after the surgery without any reported major intraoperative or postoperative compilations.

Conclusions: ECMO-assisted tracheal resection anastomosis surgery provides a method for colleagues. However, a highly skilled team, well familiar with these surgeries, is mandatory to achieve an optimum outcome.

### 1.114. Abstract 114 (Case Report-Poster): Treatment of Digital Ulcers in Systemtic Sclerosis with Thoracoscopic Sympathectomy

**Ilaria Potenza, Nicola Tamburini, Francesco Dolcetti, Francesco Quarantotto, Pio Maniscalco and Giampiero Dolci**

Arcispedale Sant’Anna, Ferrara, Italy

Background: This case study is useful to demonstrate that bilateral thoracoscopic sympathectomy, in patients with Raynaud’s phenomenon (RP) and systemic sclerosis (SSc), may result in bilateral resolution of digital ulcerations.

Case Presentation: We report two cases of SSc and RP that had bilateral benefits from thoracoscopic sympathectomy. The majority of patients with SSc have RP suffer from digital ulcerations. Medical and behavioural management may have limited benefit and surgical intervention can be considered in recalcitrant cases, although efficacy data are sparse. We describe two women, respectively 58-year-old and 37-year-old, who underwent bilateral thoracoscopic sympathectomy T2–T4 with benefit at the level of bilateral digital ulcerations.

The patients were discharged from the hospital after surgery in good clinical condition. During the follow-up after surgery, also thanks to the rheumatologists’ support, there was a resolution of the digital ulcerations and a complete re-epithelialisation of the lesions. 

Conclusions: Thoracoscopic sympathectomy is a well-tolerated procedure in patients with SSc and is associated with predictable pain relief and ulcer healing. For sure, in our institute we have a recent story of surgical approach applied to this pathology, but in light of these findings it seems prudent to offer surgical treatment not as a last resort but rather earlier in the disease process to decrease the duration that patients suffer pain.

### 1.115. Abstract 115 (Case Report-Poster): Tracheobronchopathia Osteochondroplastica: A Case Report

**Emma Repaci ^1^, Flavio Marco Mirabelli ^1^, Ilaria Menichini ^2^, Paolo Palange ^1^ and Giovanni Galluccio ^2^**

1. Department of Public Health and Infectious Diseases, Sapienza University of Rome, Rome, Italy
2. Thoracic Endoscopy, Ospedale Regina Apostolorum, Albano Laziale, Rome, Italy

Case Presentation: A 58-year-old man presented to the emergency department with a 10-day history of cough and two episodes of hemoptysis. He denied dyspnea and fever. His medical history was notable only for a significant smoking history of 40 pack-years. Physical examination revealed normal breath sounds without pathological noises. Arterial blood gas analysis indicated mild hypoxemia (pO2 65 mmHg), with normal acid-base balance. Blood tests showed a slight increase in C-reactive protein (CRP), other values were normal. Sputum cultures were negative for common bacteria, fungi, and Mycobacterium tuberculosis. Chest X-ray showed no acute inflammatory foci, but revealed rare basal atelectatic streaks. Chest CT scan showed normal lung parenchyma, but revealed submucosal calcified nodules in the trachea starting from the subglottic plane. Flexible bronchoscopy revealed an irregularly shaped trachea with multiple cartilaginous nodules (Figure 37) narrowing the distal tracheal lumen, particularly at the junction of the right main bronchus. 

The membranous part of the trachea was spared (Figure 38).

Nodules were also present in the carina and main bronchi. Histological examination of biopsy samples confirmed osteocartilaginous tissue with ossification processes in the submucosa, diagnosing tracheobronchopathia osteochondroplastica (TO). The patient underwent rigid bronchoscopy with laser treatment, removing the nodule at the right tracheobronchial angle and other protruding lesions. Follow-up endoscopies were conducted every three months to monitor disease progression and airway patency. Additional calcified lesions were removed from the left main bronchus in March 2023 and from the trachea in October 2023.

Conclusions: Tracheobronchopathia osteochondroplastica (TO) is a rare condition of unknown etiology affecting large airways, typically in adults of both sexes. It is characterized by the development of multiple osseous and cartilaginous nodules in the submucosa of the trachea and main bronchi, sparing the posterior membranous wall. Patients usually present with cough, hemoptysis, and recurrent respiratory infections. TO is often unsuspected until bronchoscopy is performed, which facilitates diagnosis due to the distinctive tracheobronchial anatomy. Bronchial biopsies show abnormal cartilaginous and osseous tissue. The disease generally remains stable or progresses slowly. There is no specific treatment for TO; management is symptomatic, addressing recurrent infections and complications. Intubation can be challenging due to calcified tracheal rings, and tracheostomy may occasionally be necessary. Surgical options include resection of the affected tracheal segment or removal of lesions via bronchoscopy when conservative measures fail.

### 1.116. Abstract 116 (Case Report-Poster): Slipping Ribs Syndrome: The Minimally Invasive Surgical Treatment

**Francesca Spinelli, Federico Raveglia, Federica Danuzzo, Maria Chiara Sibilia, Enrico Mario Cassina, Lidia Libretti, Emanuele Pirondini, Antonio Tuoro and Francesco Petrella**

Division of Thoracic Surgery, Fondazione IRCCS San Gerardo dei Tintori, Monza, Italy

Background: Slipping rib syndrome (SRS) is a condition characterized by the abnormal subluxation of the floating ribs, chronic pain, and damage to surrounding tissues. 

The incidence of SRS, often underestimated due to symptom similarity with other musculoskeletal disorders, appears to be prevalent in young females and athletes. 

Symptoms are variable but commonly include pain along the lower costal margin that tends to exacerbate with trunk movements, deep breathing or coughing, affecting daily activities. 

Initial treatment is conservative, primarily aiming at pain management through physiotherapy, analgesic medications, corticosteroids, and in severe or refractory cases, extrapleural resection of the entire costal segment.

In the following case report, we propose a surgical minimally invasive approach with rib preservation, which is unconventional.

Case Presentation: In April 2023, a 24-year-old woman suffering from right hemithorax pain at the 11th rib for one year came to our attention. Instrumental examinations (abdominal ultrasonography and magnetic resonance imaging) were negative for abdominal and osteochondral changes. Pain intensity, quantified using the Numerical Rating Scale (NRS) scale, was reported as 6. The patient undertook pain therapy but reported no beneficial effect on her symptoms.

Therefore, after careful literature research and informing the patient about the possibility of an unconventional surgical approach, we decided to refer her for surgery. The surgery was performed under general anesthesia using a mini-thoracotomy along the anterior course of the 11th rib. After exposing the costal plane, mobility of the spur of the 11th rib was revealed, leading to the resection of the medial margin and anchoring it through nonabsorbable stitches to the overlying rib. Histological examination showed no abnormalities. 

The patient was discharged on postoperative day one. One week after surgery, she reported taking analgesics with pain corresponding to an intensity of 6 on the NRS. After one month, she stopped taking analgesics, reporting a pain intensity of 1 on the NRS. A chest X-ray one month after surgery showed normal outcomes. 

Three months after surgery, the patient reported increased pain with an intensity of 3 on the NRS with a fluctuating course, occasionally treated with analgesics; however, the reported pain does not appear to limit daily life activities.

Conclusions: Minimally invasive surgical treatment has proven valuable in reducing pain, hospitalization time, and the risks associated with the more invasive surgical treatment involving rib resection, which is considered conventional to date.

Based on our experience, we present this case to provide additional support for colleagues facing similar situations.

### 1.117. Abstract 117 (Case Report-Poster): The Role of Supraclavicular Rib-Sparing Scalenectomy as a Treatment for Thoracic Outlet Syndrome (Tos)

**Sara Vaquer ^1^, Maria Chiara Sibilia ^1^, Emanuele Pirondini ^1^, Lidia Libretti ^1^, Andrea Cara ^1^, Antonio Tuoro ^1^, Federico Raveglia ^1^, Enrico Cassina ^1^, Massimo Del Bene ^2^, Erica Michela Cavalli ^2^, Andrea Marchese ^2^ and Francesco Petrella ^1^**

1 Division of Thoracic Surgery, Fondazione IRCCS san Gerardo dei Tintori, Monza, Italy
2 Division of Plastic Surgery, Fondazione IRCCS san Gerardo dei Tintori, Monza, Italy

Background: Thoracic outlet syndrome (TOS) is characterized by compression of neurological or vascular structures in the thoracic outlet, that extends from the supraclavicular fossa to the axilla. The thoracic outlet includes the brachial plexus, the subclavian vein, and the subclavian artery, all of which are vulnerable to compression in TOS caused by bony, muscular, or fibrous structures. Surgical treatment of TOS is performed via thoracic outlet decompression. The techniques that can be use are complete resection of the first rib (cartilage to cartilage), transection of the scalene muscles and complete neurolysis, venolysis or arteriolysis. Four different approaches can be chosen: the transaxillary, supraclavicular, paraclavicular and infraclavicular. Resection of the first rib is a complex and an extremely invasive approach compared to supraclavicolar rib sparing scalenectomy.

Case Presentation: Here we will describe two case reports from our experience. First, a 31-year-old woman presents bilateral paresthesia, hypothermia of the distal hand digits and pain on the left suprascapular region. Doppler ultrasound documented compression of the left subclavian artery during arm abduction. Second, a 43-year-old woman with TOS symptomatology and MRI findings of bilateral accessory cervical ribs on vertebra C7. In both cases, supraclavicular access was established via an incision starting from the posterior border of the sternocleidomastoid muscle continuing laterally above the clavicle. This type of incision allows for exposure of the brachial plexus and scalene muscle without necessitating bone resection. We later exposed the brachial plexus and scalene muscle at the supraclavicular level and isolated the phrenic nerve, accessory phrenic nerve, long thoracic nerve and the spinal accessory nerve branch. Furthermore, we isolated the superior, middle and inferior brachial plexus trunks, and performed anterior and middle scalenectomy. In the second case, we continued with resection of the C7 accessory ribs. The surgeries lasted eighty minutes and one hundred and twenty minutes respectively. Both patients were discharged on the 2nd postoperative day, without any complication.

Conclusions: Many troublesome complications (such as iatrogenic nerve injury, pneumothorax and bleeding) can result from first rib removal, which is why most physicians recommend a conservative approach. Conversely, advantages of the supraclavicular approach to scalenectomy should include reduced intraoperative time and less invasive surgery, as well as improved postoperative recovery times and fast functional rehabilitation for patients. We share our experience with the goal of describing an effective alternative solution for colleagues facing similar cases.

## Figures and Tables

**Figure 1:**
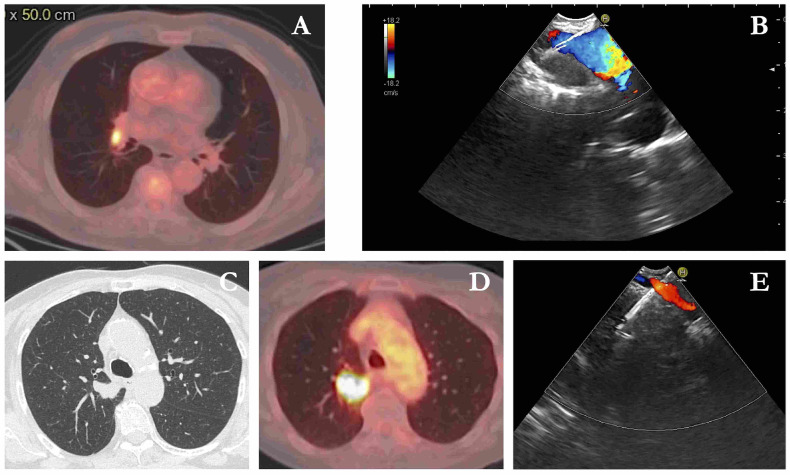
Positron emission tomography (PET)/computed tomography (CT) scan of a right interlobar lymph node (**A**) and transvascular EBUS−TBNA of the lymph adenopathy (**B**). CT (**C**) and PET/CT (**D**) scan of a right upper lobe pulmonary nodule and transvascular EUS−B-FNA of the lung lesions (**E**).

**Figure 2:**
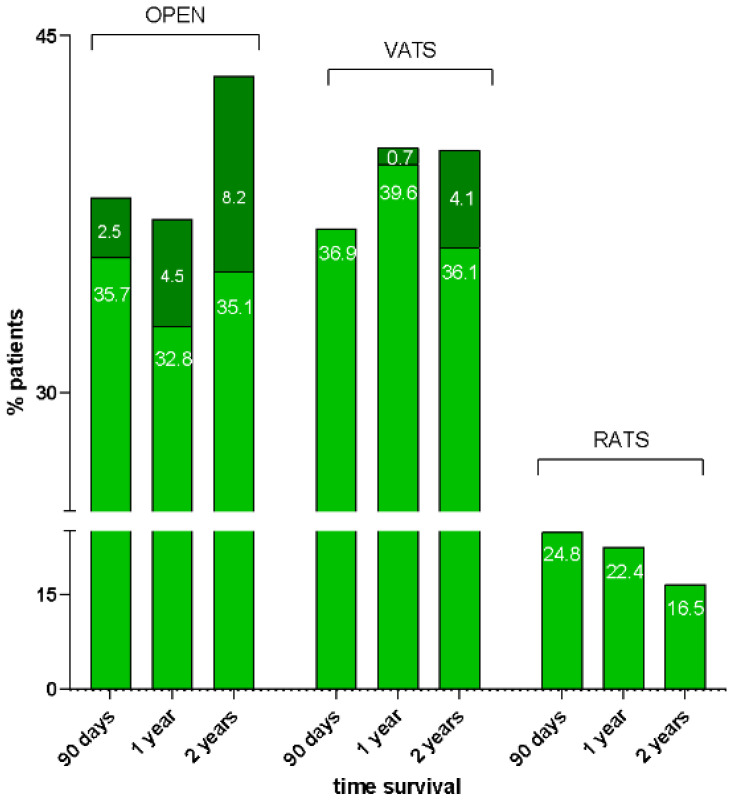
Survival analysis in NSCLC patients who underwent open surgery, VATS and RATS.

**Figure 3:**
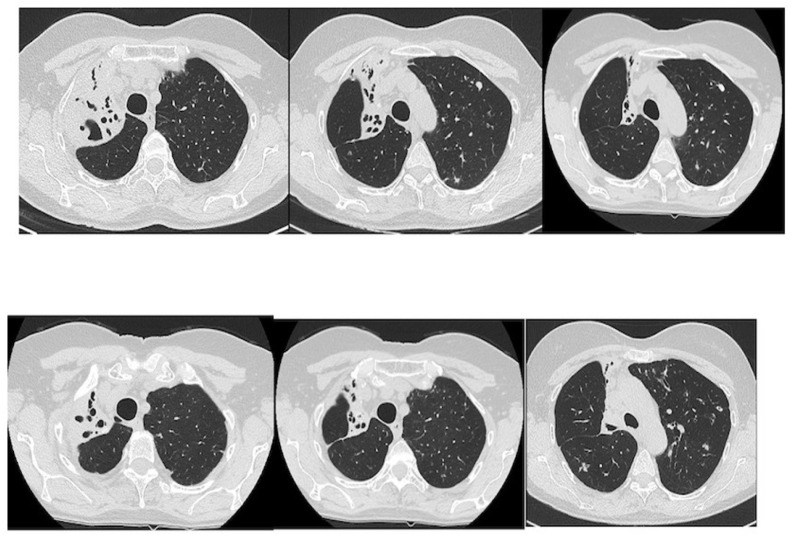
NTM cavities pre and post valve placement.

**Figure 4:**
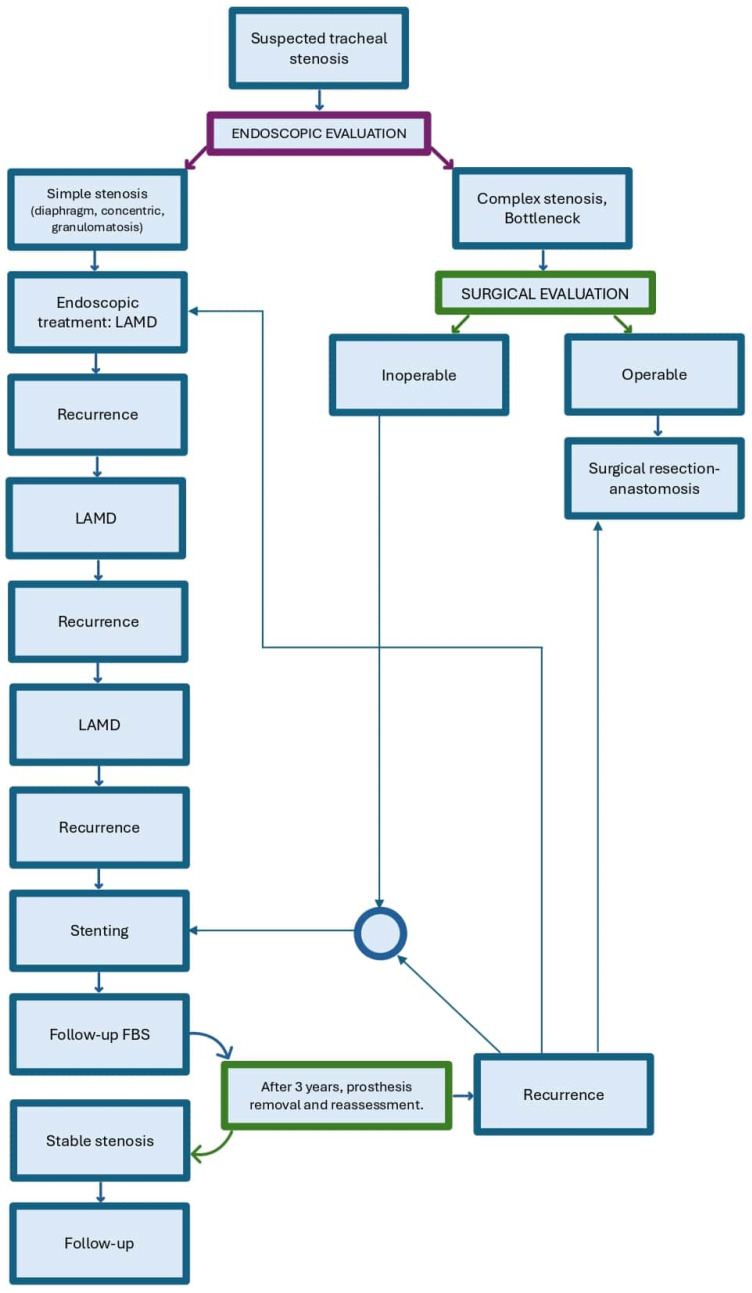
Diagnostic and therapeutic process.

**Figure 5:**
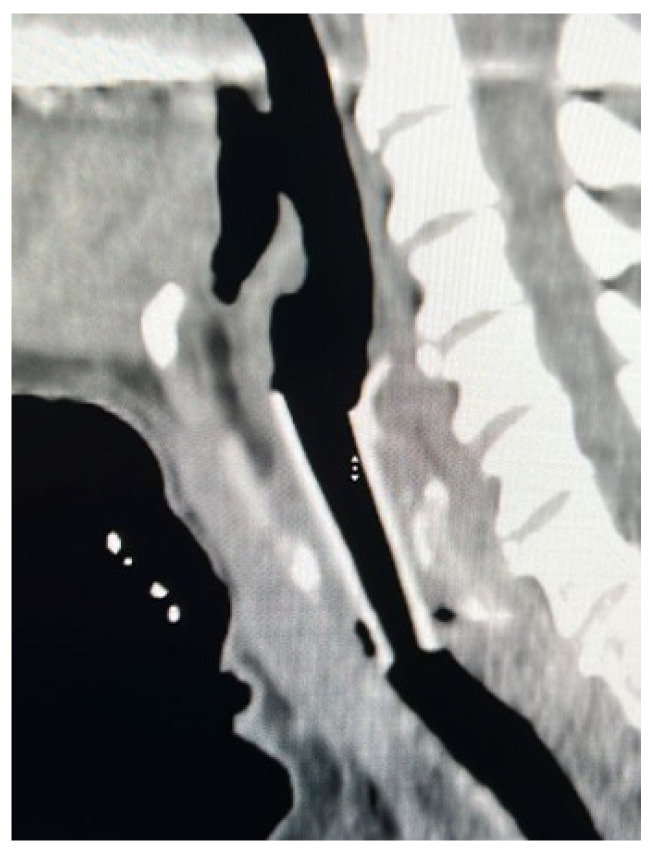
Stent in anastomotic dehiscence.

**Figure 6:**
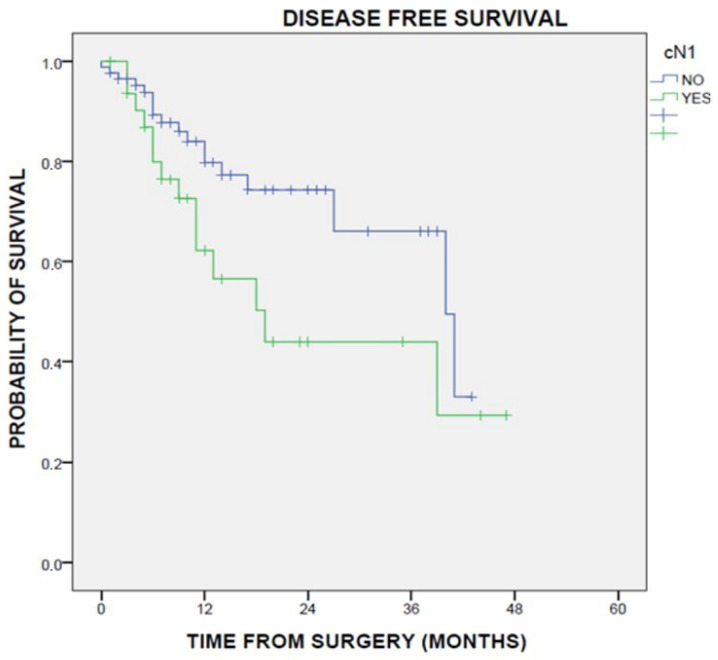
Univariate analysis for DFS.

**Figure 7:**
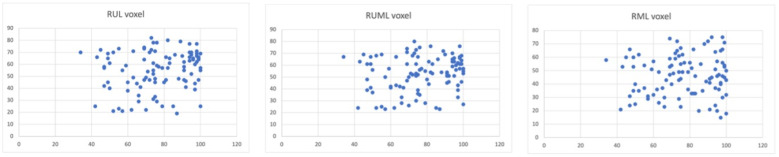
Evaluation of severity of emphysema and HF integrity.

**Figure 8:**
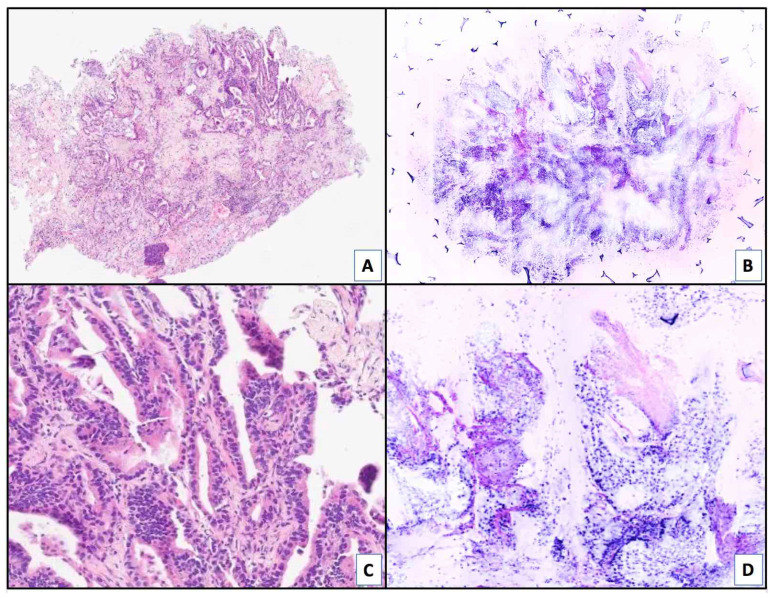
(**A**,**B**) Scan magnification of lung cryobiopsies: H&E (**A**) and ex-vivo confocal microscopy (**B**). (**C**,**D**) Higher magnification.

**Figure 9:**
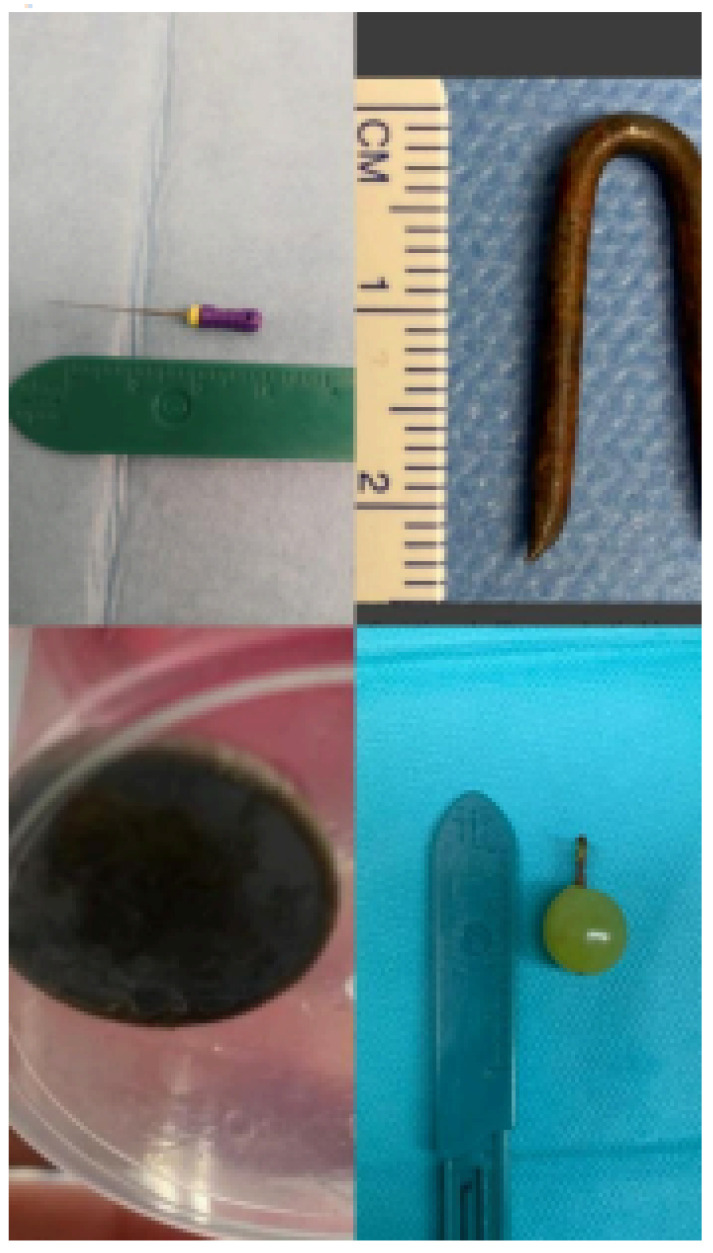
Example of tracheobronchial foreign body (TFB) retrieved from the airways of adult patients (clockwise, from left to right): dental device for root canal treatment, nail, coin, grape.

**Figure 10:**
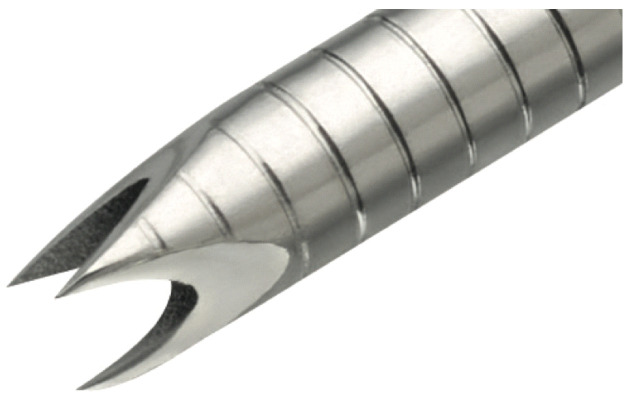
Crown-cut TBNB needle.

**Figure 11:**
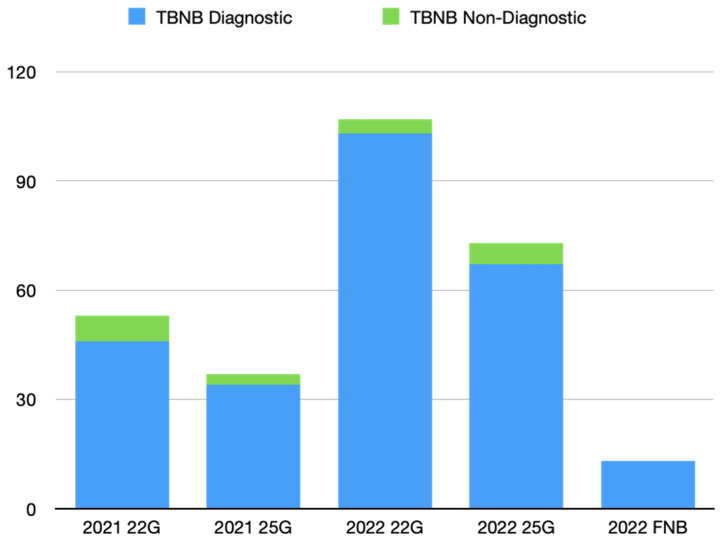
Diagnostic Yield (DY) of 22 G and 25 G TBNB needles, divided per year and EUS-B-FNB DY.

**Figure 12:**
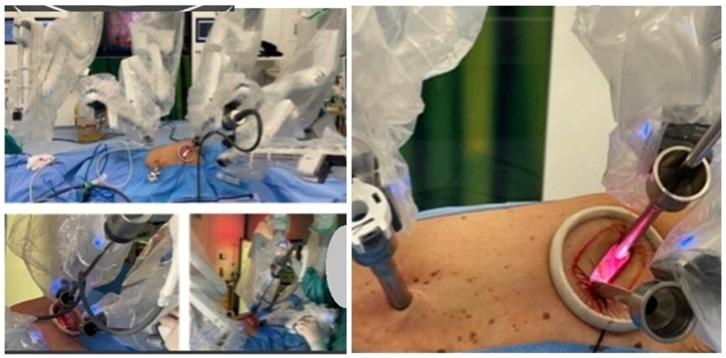
Dual-Port Fully Robotic approach port mapping: 3–4 cm working port using a wound protector (Alexis wound retractor XS^®^, Applied Medical, Rancho Santa Margarita, CA, USA) in the fifth intercostal space, along the posterior axillary line and 1.2 cm working port on the eight intercostal space, along the midaxillary line.

**Figure 13:**
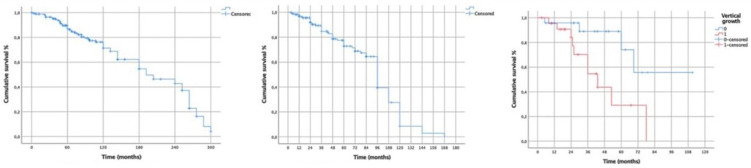
Survival analysis.

**Figure 14:**
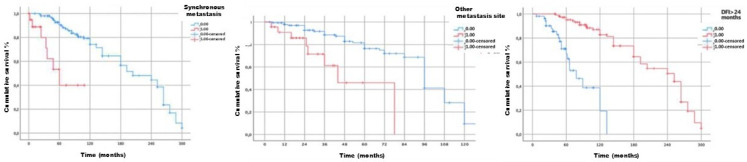
Prognostic factor analysis.

**Figure 15:**
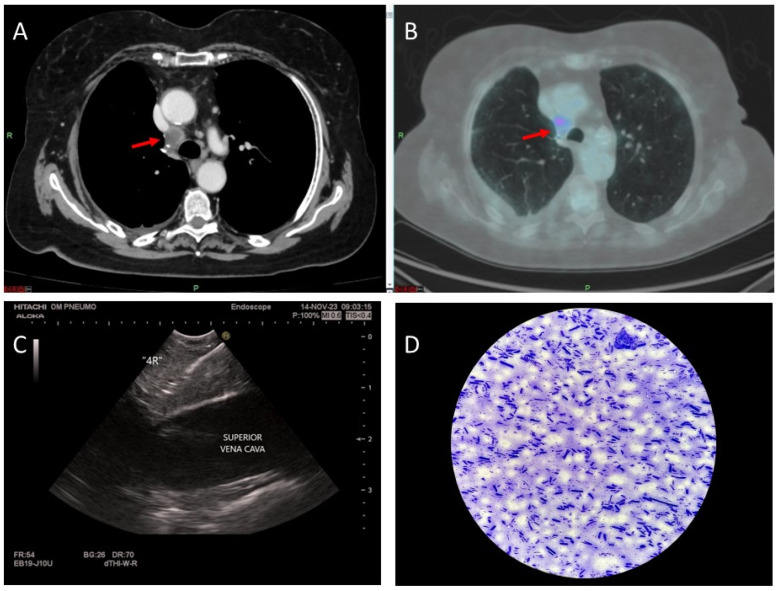
Right paratracheal enlargement (indicated by the red arrow) at chest CT (**A**), and at FDG PET/CT, with SUVmax 6.0 (**B**); EBUS-TBNA on right paratracheal enlargement (**C**); ROSE with diff quick stain, 100× magnification: fibrillar material, lymphocytes and macrophages (**D**).

**Figure 16:**
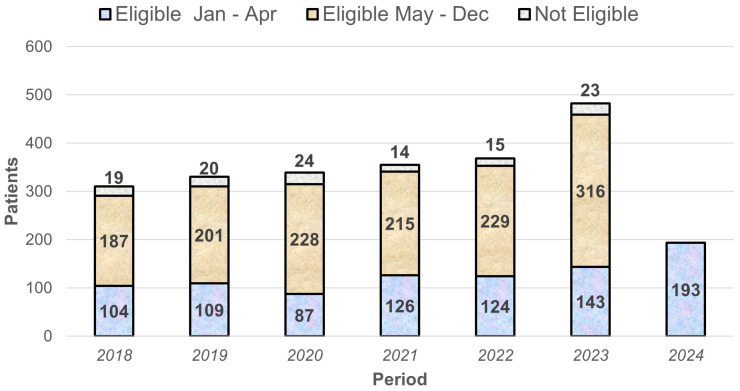
Patients managed by LCP during the years.

**Figure 17:**
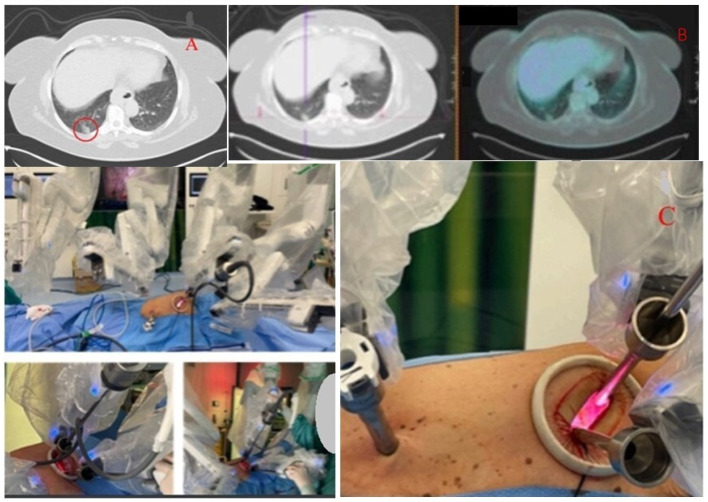
Preoperative CT, showing pulmonary lesion in red circle (**A**) and PET (**B**). Surgical port mapping (**C**).

**Figure 18:**
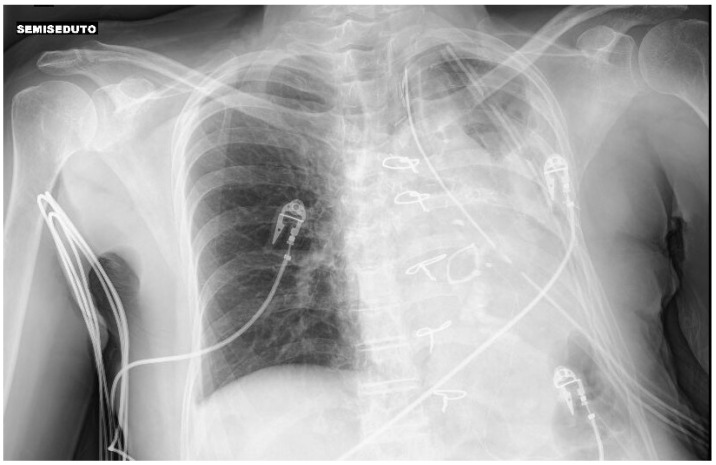
Postoperative chest X-ray.

**Figure 19:**
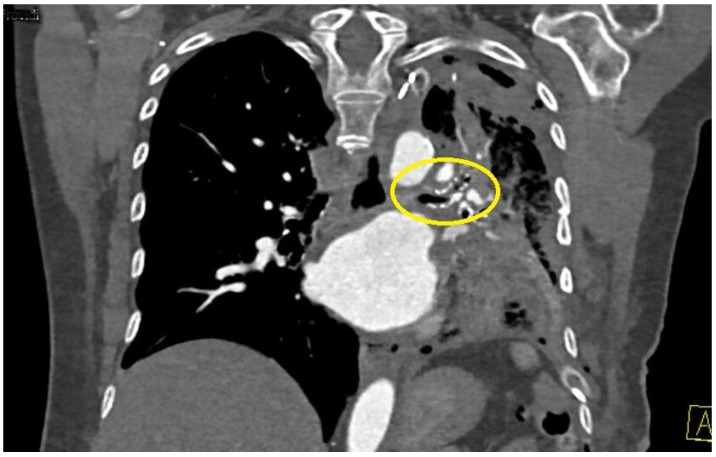
Post operative chest Ct scan, showing kinking of the lower lobar bronchus (yellow circle).

**Figure 20:**
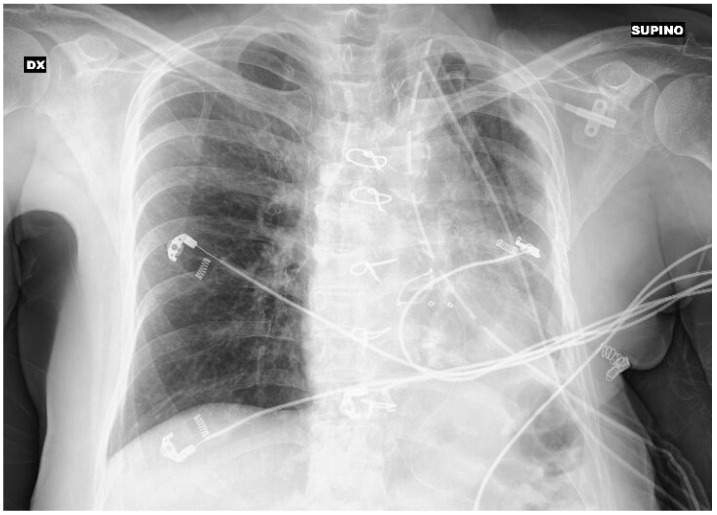
Chest X-ray after surgical revision.

**Figure 21:**
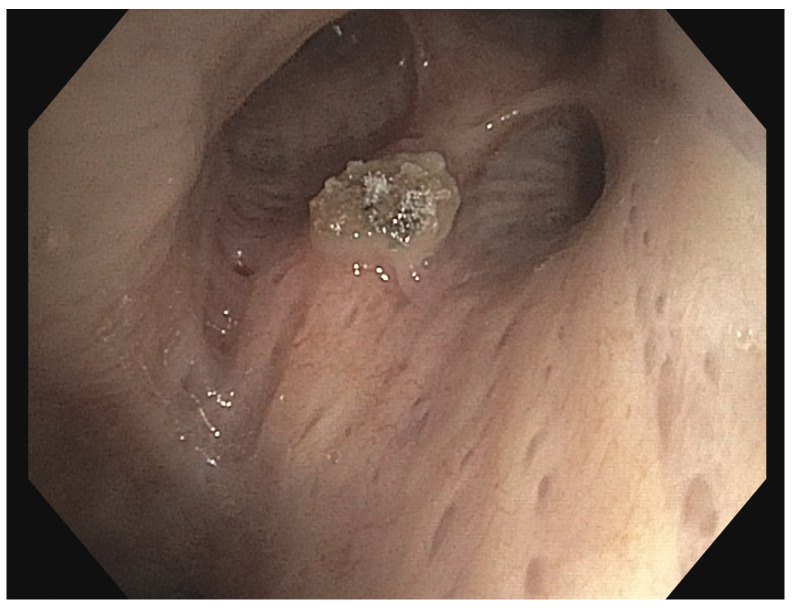
Silver-colored endobronchial lesion, highlighting the characteristic silver hue of the lesion during the endoscopic examination.

**Figure 22:**
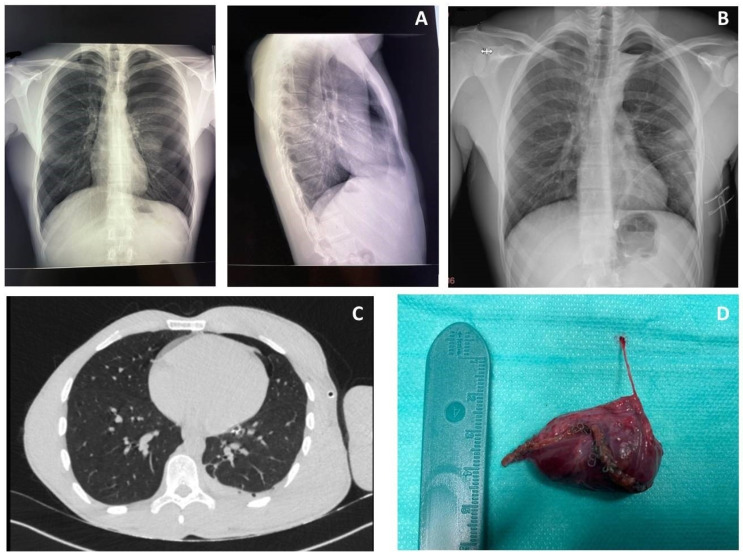
Chest X-ray showing left spontaneous pneumothorax (**A**); Chest X-ray showed an air-fluid level in left apical paramediastinal region after chest tube positioning (**B**); Chest Ct-scan (**C**); Surgical specimen (**D**).

**Figure 23:**
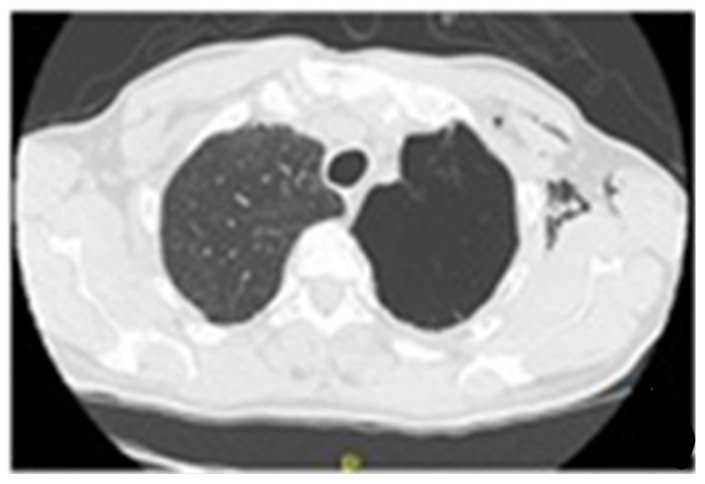
Pre LVRS TC-scan.

**Figure 24:**
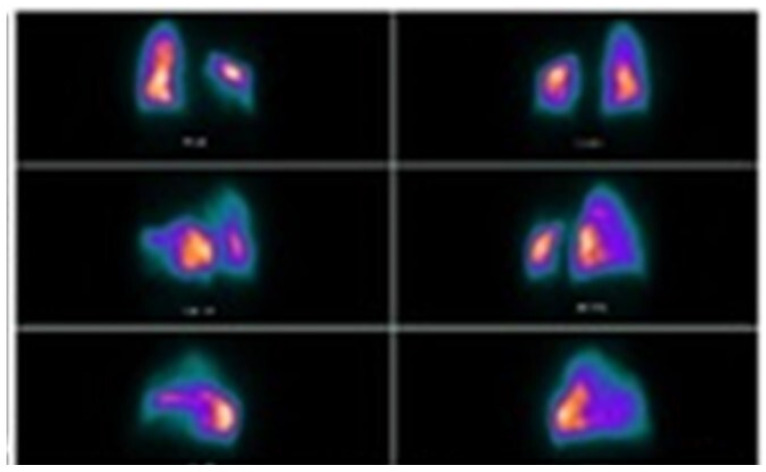
Lung scintigrafy.

**Figure 25:**
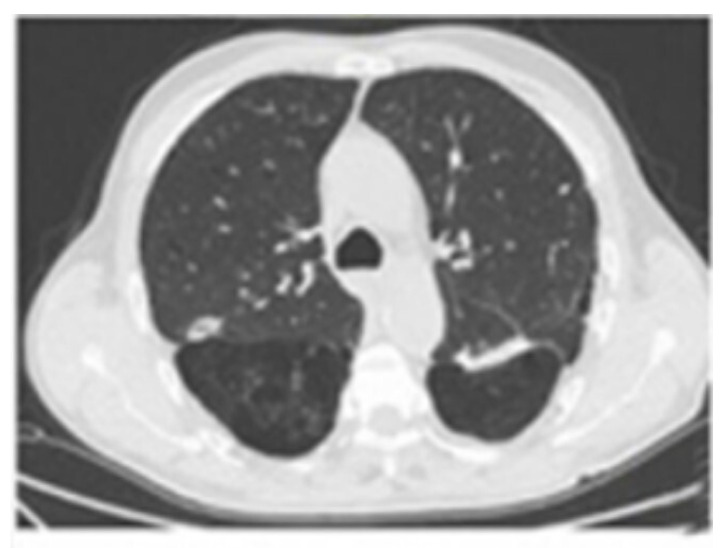
After LVRS TC-scan (nodule).

**Figure 26:**
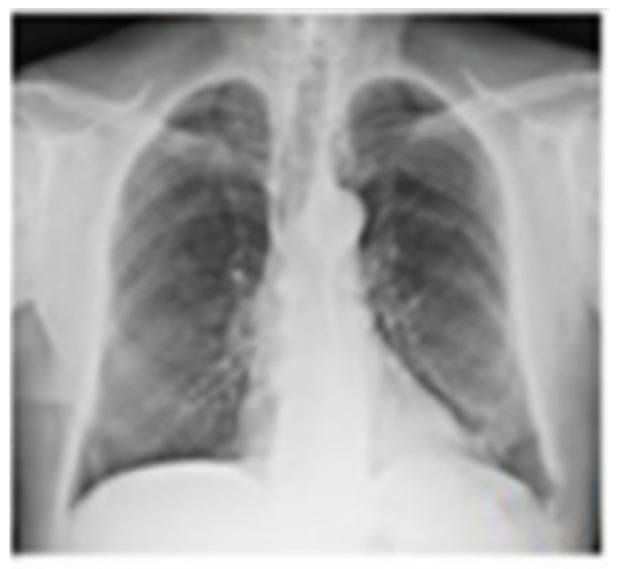
1 month X-ray chest.

**Figure 27:**
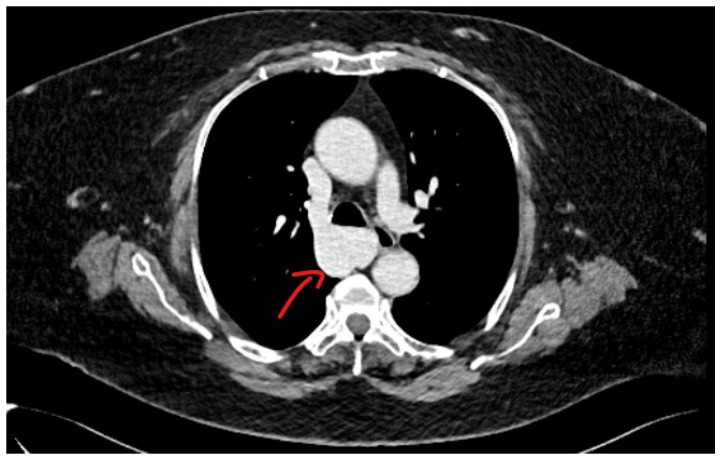
Chest angio-CT scan shows azygos ectasia (arrow).

**Figure 28:**
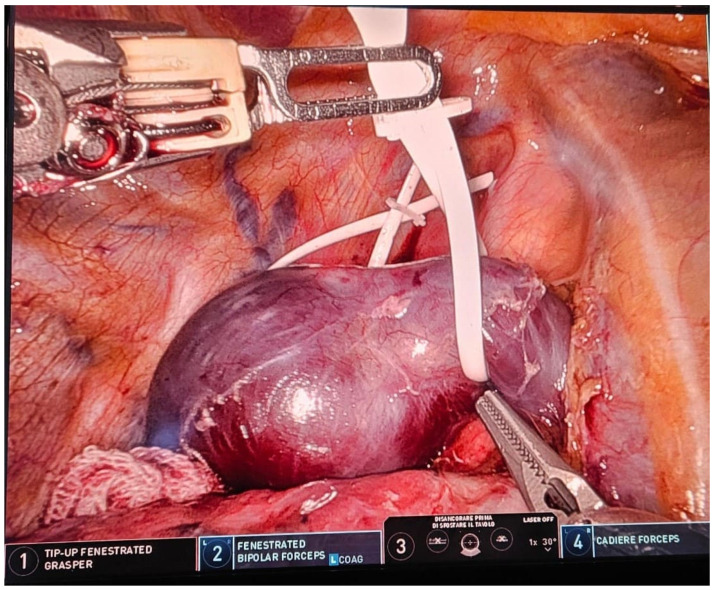
Intraoperative view of azygos vein.

**Figure 29:**
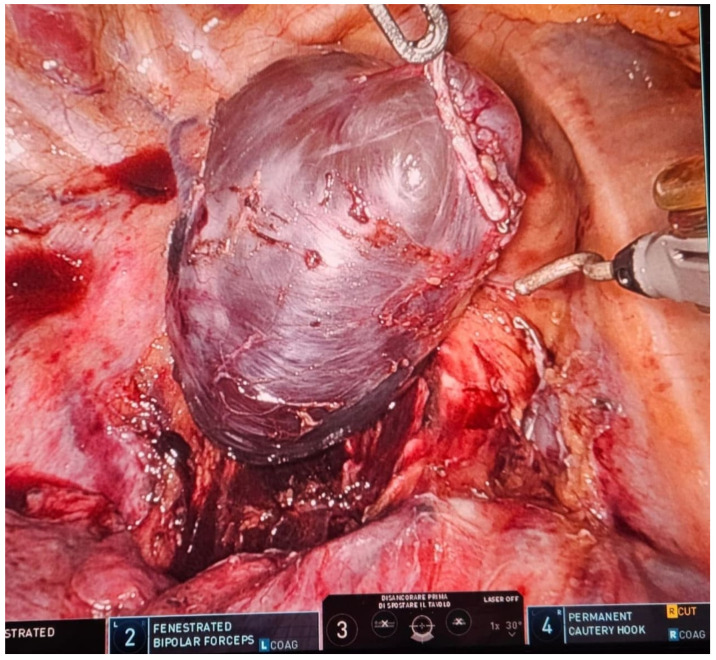
Intraoperative view after azygos section.

**Figure 30:**
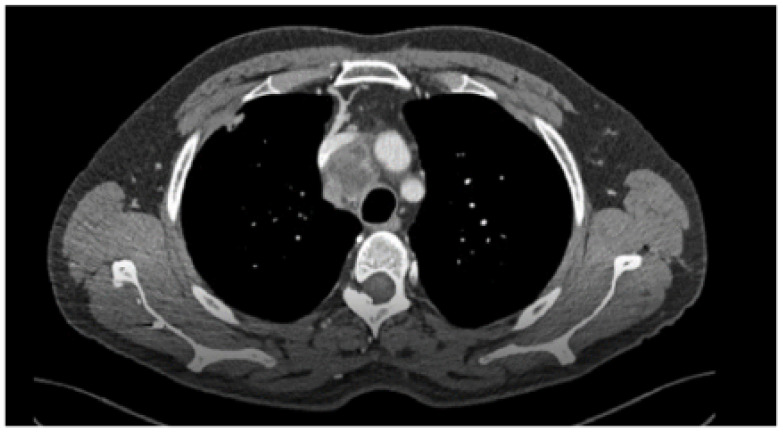
Lymphadenopathy of station 4R.

**Figure 31:**
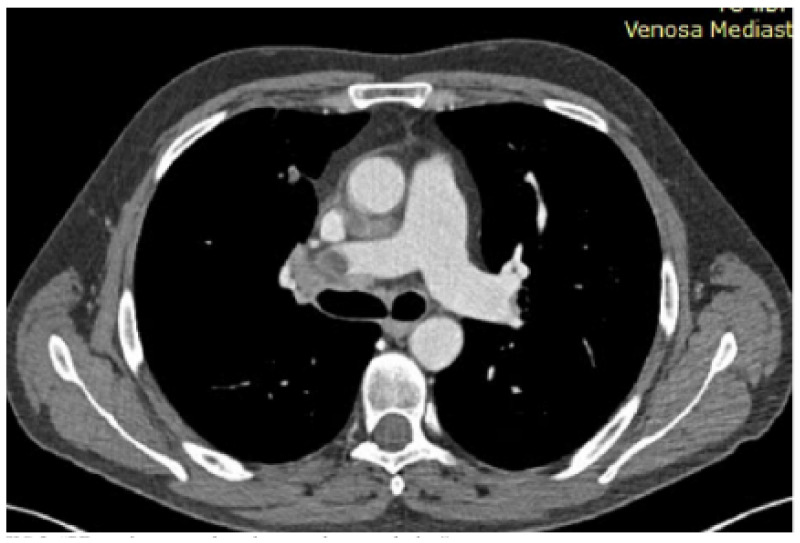
PE contiguous to the primary pulmonary lesion.

**Figure 32:**
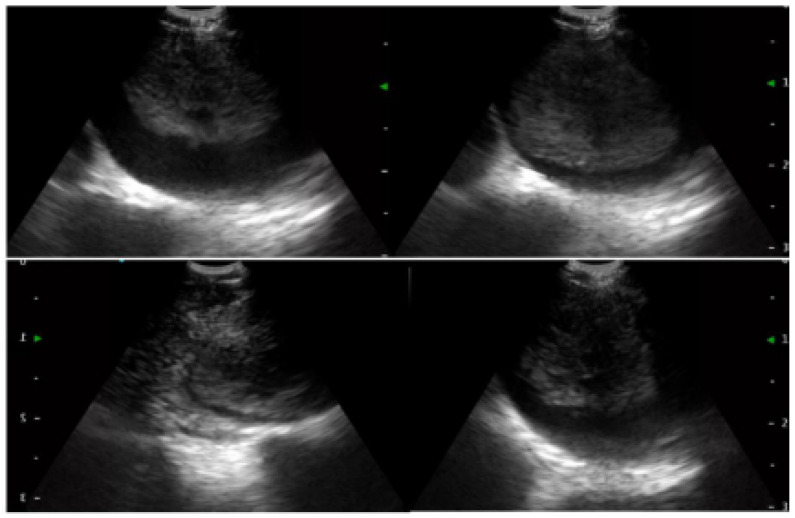
EBUS view of round, centimeter-sized lesion in continuity with the vascular wall and surrounded by blood flow.

**Figure 33:**
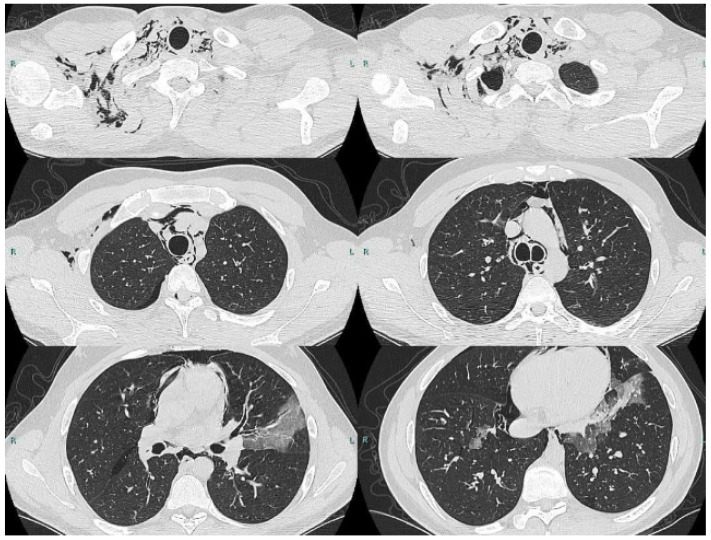
Chest CT scan at admission.

**Figure 34:**
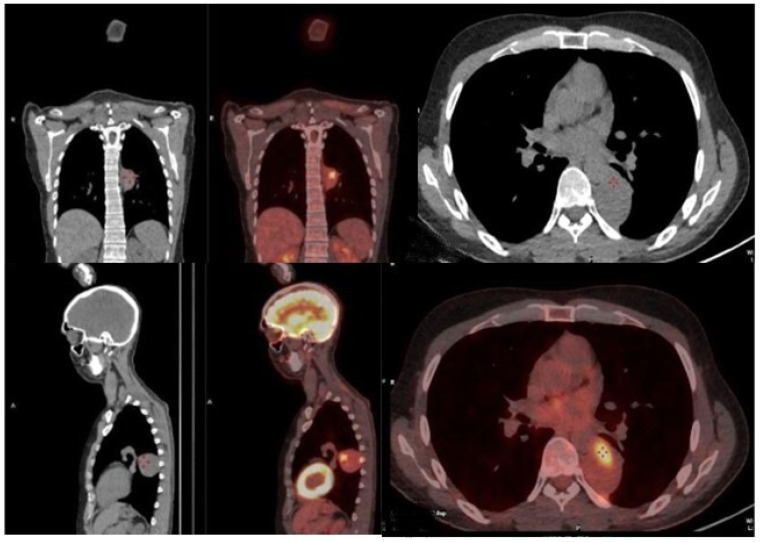
Preoperative PET.

**Figure 35:**
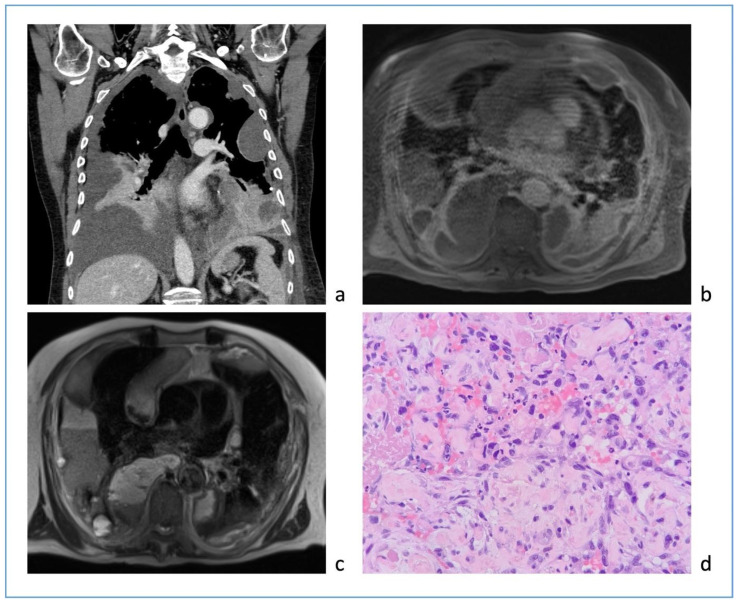
Radiological and pathological features of the presented case. (**a**) Diffuse parietal pleura thickening and bilateral organized pleural effusion on contrast CT of the chest. (**b**,**c**) Contrast chest MR showing bilateral pleural effusion with multiple saccular aspects due to partially bloody content. In the contrast phase, bilateral linear contrast enhancements of the pleura and laminar areas of restricted protonic diffusivity were evident. (**d**) Microscopic pathological findings: irregularly shaped anastomosing vascular channels lined by atypical endothelial cells with a highly infiltrative architecture and poor demarcation (hematoxylin and eosin, magnification 10×).

**Figure 36:**
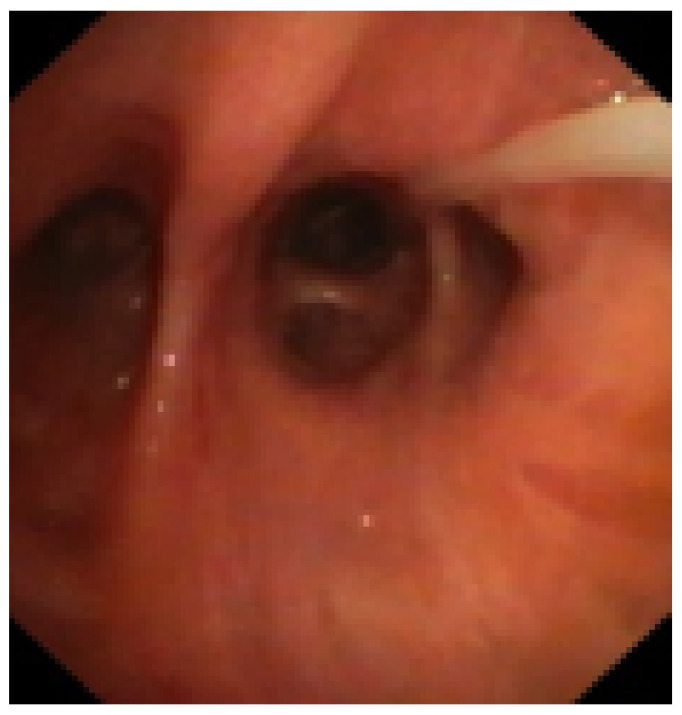
Broncoscopy.

**Figure 37:**
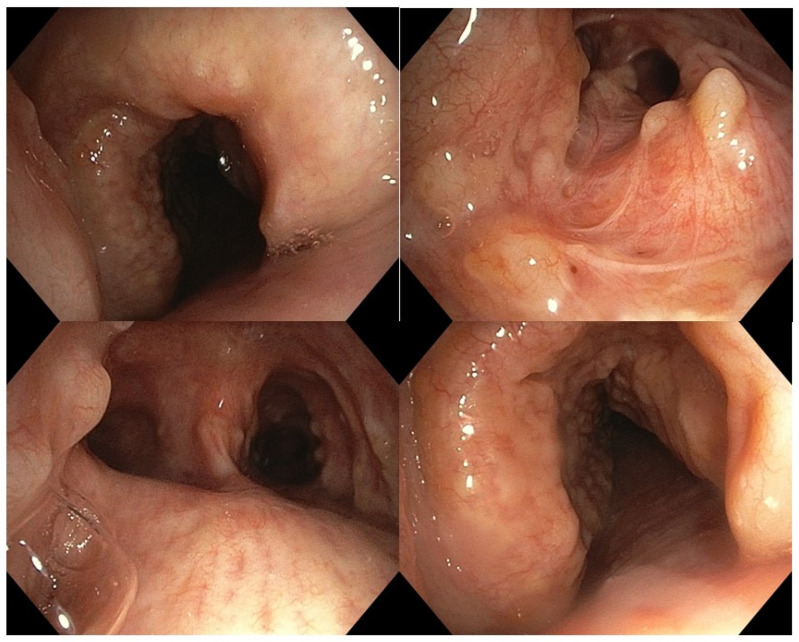
Irregularly shaped trachea with multiple cartilaginous nodules.

**Figure 38:**
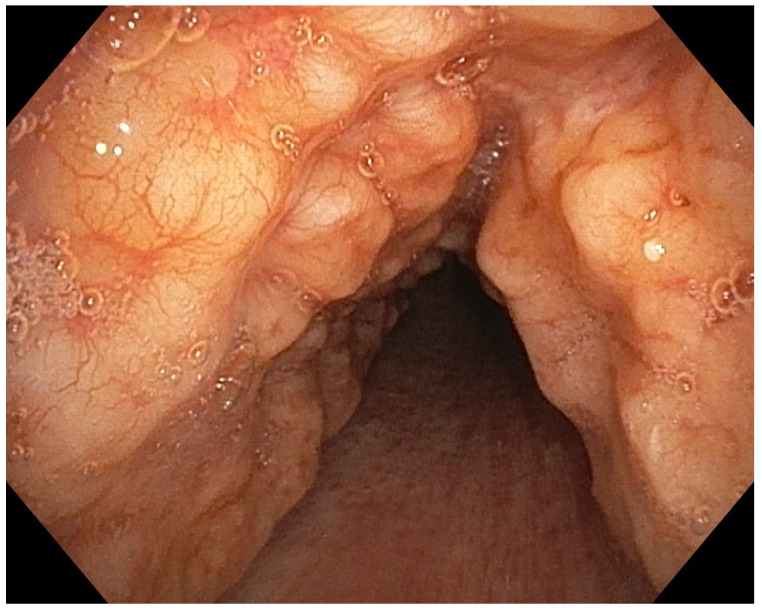
Ultiple cartilaginous nodules present in the carina and main bronchi.

**Table 1:** Summary of transvascular endosonographic procedures.

Endoscopic Procedure	Patient Age/Gender	Target Lesion	Lesion Size (mm) *	Traversed Vessel	Endoscopic Diagnosis	Final Diagnosis	Gold Standard Technique	Complication
EBUS-TBNA	77/F	12R	9	PA right interlobar branch	Breast cancer	Breast cancer	-	None
EBUS-TBNA	78/M	12R	9	PA right interlobar branch	Negative	Lung adenocarcinoma	VATS	Mild bleeding
EBUS-TBNA	70/M	12L	10	PA left interlobar branch	Colorectal adenocarcinoma	Colorectal adenocarcinoma	-	None
EUS-B-FNA	74/M	RUL mass	41	Azygos vein	SCLC	SCLC	-	None
EUS-B-FNA	75/F	RUL nodule	26	Azygos vein	SCLC	SCLC	-	None
EUS-B-FNA	79/M	RUL mass	36	Azygos vein	Lung adenocarcinoma	Lung adenocarcinoma	-	None

* long axis for pulmonary lesions, short axis for lymph nodes. EBUS-TBNA: endobronchial ultrasound-guided transbronchial needle aspiration; VATS: video-assisted thoracoscopic surgery; EUS-B-FNA: transesophageal endoscopic ultrasound with bronchoscope fine needle aspiration; RUL: right upper lobe; PA: pulmonary artery; SCLC: small-cell lung cancer.

**Table 2:** Clinical details.

Features	Value and Percentage or Mean ± SD
Gender	
Male	3 (15%)
Female	17 (85%)
Age	31.80 ± 10.58 years
Comorbidity	8 (40%)
Smoking habit	2 (10%)
Type of lymphoma	
Hodgkin Lymphoma	16 (80%)
Non Hodgkin Lymphoma	4 (20%)
Clinical stage (at diagnosis)	
IIA	6 (30%)
IIB	11 (55%)
IV	3 (15%)
Therapy	
Chemotherapy	20 (100%)
Radiotherapy	1 (5%)
Immunotherapy	2 (10%)
Follow up CT	
Stable	11 (55%)
Progression	9 (45%)
Dimension of lesion at CT scan	36.95 ± 16.82 mm
Time to suspicion of relapse	25.00 ± 22.54 months
Follow up PET (Deouville score)	
Deouville 4	11 (55%)
Deouville 5	9 (45%)
SUV max	7.01 ± 5.70
SUV max/mid hepatic	3.58 ± 1.51

**Table 3:** Demographic characteristics.

Mean age at test	26.7 ± 2.5
Gender, *n* (%)	
Male	50 (48.5%)
Female	53 (51.5%)
School of residency area, *n* (%)	
North	48 (46.6%)
Centre	35 (34%)
South	20 (19.4%)
Year of enrollment, *n* (%)	
2019 (5th year)	22 (21.5%)
2020 (4th year)	34 (33%)
2021 (3rd year)	31 (30%)
Relationship, *n* (%)	
Single	22 (21.7%)
Stable relationship (non marriage)	49 (47.4%)
Married	11 (10.3%)
Recent relationship	13 (12.4%)
Infants, *n* (%)	
Yes	4 (3.8%)
No	99 (96.2%)

**Table 4:** Operative and postoperative details.

Variable	Simple Segmentectomy Number (%) or Mean	Complex Segmentectomy Number (%) or Mean	Univariable *p* Value	Single Segmentectomy Number (%) or Mean	Multiple Segmentectomy Number (%) or Mean	Univariable *p* Value
**Segments Included**	S6 DX/SX S1-S2-S3 SX S4-S5 SX	S1 DX/SX S2 DX/SX S3 DX/SX S1-S2 DX/SX S1-S3 SX S6-S7 SX S7 DX S7-S8-S9 DX S8-S9 SX S9-S10 SX	-	S1 DX/SX S2 DX/SX S3 DX/SX S6 DX/SX S7 DX	S1-S2-S3 SX S4-S5 SX S1-S2 DX/SX S1-S3 SX S6-S7 SX S7-S8-S9 DX S8-S9 SX S9-S10 SX	-
**Patients Number**	80	47		64	63	
**Complication**			0.243			0.337
yes	18 (22.5)	15 (31.9)		19 (29.6)	14 (22.2)	
no	62 (77.5)	32 (68.1)		45 (70.4)	49 (67.8)	
**Prolonged Air Leak**			0.73			0.341
Yes	5 (6.2)	5 (10.6)		7 (10.9)	3 (4.7)	
no	75 (93.8)	42 (89.4)		57 (89.1)	60 (95.3)	
**Haemorrhagic Complication**			0.51			0.774
Yes	4 (5.0)	0 (0)		2 (3.1)	2 (3.2)	
no	76 (95.0)	47 (100)		62 (96.9)	61 (96.8)	
**ICU**			0.725			0.073
yes	7 (8.7)	5 (11.9)		9 (14.0)	3 (4.7)	
no	73 (92.3)	42 (88.1)		55 (86.0)	60 (95.3)	
**Respiratory Failure**			0.215			0.905
Yes	4 (5.0)	1 (2.1)		3 (4.6)	2 (3.2)	
no	76 (95.0)	46 (97.9)		61 (95.4)	61 (96.8)	
**Hospital Stay (days)**	4.5 ± 1.9	4.9 ± 2.2	0.33	4.8 ± 2.1	4.56 ± 1.9	0.486
**pT**			0.114			0.652
1a	20 (25.0)	20 (42.5)		22 (34.4)	18 (28.6)	
1b	40 (50.0)	14 (29.8)		26 (40.7)	28 (44.5)	
1c	15 (18.8)	9 (19.2)		13 (20.3)	11 (17.4)	
2	5 (6.2)	4 (8.5)		3 (4.6)	6 (9.5)	
**Tumor Dimension (CM)**	1.65 ± 0.7	1.55 ± 0.9	0.55	1.57 ± 0.8	1.66 ± 0.9	0.546
**Margin Distance (CM)**	7.3 ± 5.4	6.9 ± 5.7	0.75	5.9 ± 5.5	8.4 ± 5.2	0.012
**Resected *N* Stations**	1.9 ± 1.2	2.1 ± 1.3	0.36	2.1 ± 1.3	2.0 ± 1.2	0.632
**Resected N1 Lymphnodes**	1.32 ± 2.6	3.03 ± 1.8	0.186	1.7 ± 2.3	1.6 ± 2.4	0.932
**Resected N2 Lymphnodes**	3.0 ± 3.6	4.8 ± 5.0	0.02	4.6 ± 4.8	2.7 ± 3.4	0.012
**Total Resected Lymphnodes**	5.0 ± 4.6	6.1 ± 5.6	0.207	6.3 ± 5.5	4.5 ± 4.3	0.047

**Table 5:** Analysis of factors influencing the operative time.

	Univariate Analysis		Multivariate Analysis		
	Mean (SD)	pue	RC	95% CI	*p*-Value
Gender		<0.001	−10.4	−20; −0.8	0.033
M (0)	135 (55)				
F (1)	115 (40)				
Age >60 years		0.004	−1.2	−12; 9.6	0.823
no (0)	118 (45)				
si (1)	135 (54)				
Myasthenia gravis		0.962			
no (0)	123 (55)				
si (1)	123 (46)				
Histology		0.010	11.7	1.7; 21.7	0.022
Iperplasia (0)	119 (43)				
Neoplasia (1)	133 (57)				
Experienced surgeon		<0.001	−29.9	−42.3; −17.6	<0.001
no (0)	144 (54)				
si (1)	119 (46)				
Thymus volume	0.324	<0.001	0.097	0.06; 0.13	<0.001

**Table 6:** Patient characteristics.

	Sex, Age (Years)	Medical History	Disease and Previous Treatment	ECMO Enrolment Criteria	Procedure (Date)	ECMO Duration	Postoperative Follow-Up (Duration)	Current Condition
1	M, 67	Past smoker Tuberculosis Epilepsy Esophagectomy for cancer Left vocal cord paralysis	Cancer recurrence Total esophagectomy Per-operative laceration of the entire trachea Mediastinitis	Respiratory failure	Extended Y stenting Tracheotomy (May 2016)	1.5 h	Uneventful Tracheotomy removal (1 year) Stent removal (1 year, 4 months)	Alive No cancer recurrence No tracheotomy No stent
2	F, 25	Chronic cough	Typical carcinoid tumour Right main stem bronchus obstruction Right lung atelectasis Preoperative embolization	Massive bleeding Respiratory failure	Massive bleeding Incomplete bronchoscopic tumour removal Stent Tracheotomy (October 2016)	22 days	Pneumothorax Pneumonia Tracheotomy removal (2 months) Stent removal (4 months)	Alive Medical treatment (mTOR inhibitor) Pneumonectomy No tracheotomy No stent
3	F, 57	Past smoker Asthma Breast cancer: Surgical resection + Chemoradiation Tracheal adenoid cystic carcinoma (ACC): endoscopic resection	Recurrent ACC: carinal resection (January 2016) ARDS Left main stem bronchus stenting Long-term mediastinal stent migration	Massive bleeding Respiratory failure	Minor bleeding Placement of a new stent in the left main stem bronchus (October 2017)	1.5 h	Uneventful Chronic colonization by Pseudomonas aeruginosa Asthma	Alive No recurrence of ACC No tracheotomy One stent in place
4	F, 60	Past smoker Morbid obesity Hypertension Gastro-esophageal reflux disease Pulmonary embolism (×3), sleep apnea, bowel obstruction, Hysterectomy	Excessive dynamic airway collapse (EDAC): surgical treatment in 2004 Multiple rigid bronchoscopies since 2008: stent placement and removal, granulation tissue removal	Respiratory failure	Extended Y stenting Tracheotomy (October 2017)	2 h	Uneventful Acute bronchitis Tracheotomy Removal (8 months) Tracheoplasty (1 year 4 months)	Alive No tracheotomy Stent in place
5	F, 20	Undetermined inflammatory tracheobronchial disease Recurrent pneumonia and respiratory failure Corticosteroid-induced osteoporosis, Cachexia	Multiple tracheal and bronchial stenoses Medical treatment FEV1 = 3 40 mL (11.4%) Residual lumen of the Trachea = 3–4 mm	Respiratory failure	Tracheal dilatation (x2) and stenting Bronchial dilatation (April 2019)	24 h	Uneventful Stent obstruction Stenosis recurrence Stent removal and new Y stenting under VV ECMO	Alive No tracheotomy One stent in place
6	M, 49	T-cell lymphoma treated by chemotherapy complicated by mediastinitis and tracheal wall necrosis febrile pancytopenia	Fiberoptic bronchoscopy: tracheal wall necrosis and right main bronchus stenosis	Respiratory failure	Tracheal stenting (November 2022)	1.5 h	Uneventful	Alive with stent in place
7	M, 59	Tracheal adenoid cystic carcinoma (ACC)	Rigid bronchoscopy: desobstruction and Y stent placement (September 2020) Tracheal and carinal replacement by stented aortic allograft (January 2021)	Respiratory failure	In situ fenestration of 2 h tracheal stent at the level of main left bronchus (January 2021)	2 h	Uneventful Readmission for inspiratory dyspnea and purulent sputum (Aspergillus infection) Pneumothorax Granulation removal by rigid bronchoscopy (4years, 3 months)	Alive with tracheal stenting
8	F, 48	Partial gastrectomy for caustic ingestion and temporary esophageal stenting (2010–2014). Cholecystectomy Depressive disorder Esophageal. squamous cell carcinoma treated by McKeown esophagectomy Jejunostomy for nutrition	Posterolateral tracheal wall lesion post-esophagectomy for cancer Septic shock Rigid bronchoscopy: tracheal stenting (April 2021)	Respiratory failure	Tracheal Y stenting Main left bronchus dilatation (April 2021)	2 h	ARDS, septic shock. Major air leakage and hypercapnia under Mechanical ventilation	Dead (16 days)
9	M, 26	Cranial injury resulting from traffic accident, mechanical ventilation for 17 days Subglottic post-tracheostomy stenosis (September 2017)	Tracheal resection and reanastomosis (October 2017) Tracheostomy for tracheal stenosis recurrences undergone iterative rigid bronchoscopies Tracheal replacement with stented aortic allograft (October 2022)	Respiratory failure	Stent removal Bilateral main stem bronchi dilatation Y tracheal stenting (January 2024)	2 h	Multiple tracheal and bronchial stenosis treated with dilatation and desobstruction	Alive with tracheostomy Y stent in place Pseudomonas aeruginosa infection

**Table 7:** Preoperative, operative, and postoperative characteristics.

	UNMATCHED				MATCHED		
	All	Incentive Spirometry	Early Ambulation	*p*-Value	Incentive Spirometry	Early Ambulation	*p*-Value
Total	304	153	151		52	52	
Age							
Median [range]	65.5 [25–85]	64.0 [33–85]	67.0 [25–82]	0.01	65 [34–84]	65 [32–79]	
<60	92 (30.3)	56 (36.6)	36 (23.8)		15 (28.8)	15 (28.8)	=
60–69	106 (34.9)	52 (34.0)	54 (35.8)		21 (40.4)	21 (40.4)	=
70+	106 (34.9)	45 (29.4)	61 (40.4)	0.03	16 (30.8)	16 (30.8)	=
Sex							
Women	170 (55.9)	118 (77.1)	52 (34.4)		25 (48.1)	25 (48.1)	=
Men	134 (44.1)	35 (22.9)	99 (65.6)	<0.0001	27 (51.9)	27 (51.9)	=
BMI							
Median [range]	25.2 [16.4–45.7]	22.9 [16.4–45.7]	28.1 [20.0–37.3]	<0.0001	25.1 [17.7–37.8]	25.7 [20.0–34.2]	
Normal weight	144 (47.4)	117 (76.5)	27 (17.9)		25 (48.1)	25 (48.1)	=
Overweight	109 (35.9)	25 (16.3)	84 (55.6)		20 (38.5)	20 (38.5)	=
Obese	51 (16.8)	11 (7.2)	40 (26.5)	<0.0001	7 (13.5)	7 (13.5)	=
Smoking							
Never	88 (28.9)	56 (36.6)	32 (21.2)		17 (32.7)	9 (17.3)	
Former	149 (49.0)	62 (40.5)	87 (57.6)		23 (44.2)	32 (61.5)	
Current	67 (22.0)	35 (22.9)	32 (21.2)	0.004	12 (23.1)	11 (21.2)	0.14
Previous lung surgery							
No	265 (87.2)	134 (87.6)	131 (86.8)		46 (88.5)	47 (90.4)	
Yes	39 (12.8)	19 (12.4)	20 (13.2)	0.83	6 (11.5)	5 (9.6)	0.75
Neoadjuvant therapy							
None	266 (87.5)	132 (86.3)	134 (88.7)		46 (88.5)	47 (90.4)	
CT	36 (11.8)	19 (12.4)	17 (11.3)		4 (7.7)	5 (9.6)	
RT	1 (0.3)	1 (0.7)	0 (0.0)		1 (1.9)	0 (0.0)	
CT-RT	1 (0.3)	1 (0.7)	0 (0.0)	0.55	1 (1.9)	0 (0.0)	0.55
Type of surgery							
Sub-lobar	127 (41.8)	74 (48.4)	53 (35.1)		22 (42.3)	12 (23.1)	
Lobectomy	154 (50.7)	70 (45.8)	84 (55.6)		25 (48.1)	31 (59.6)	
Bi-lobectomy	6 (2.0)	2 (1.3)	4 (2.6)		1 (1.9)	3 (5.8)	
Pneumonectomy	17 (5.6)	7 (4.6)	10 (6.6)	0.12	4 (7.7)	6 (11.5)	0.17
Access							
Thoracotomy	154 (50.7)	77 (50.3)	77 (51.0)		26 (50.0)	26 (50.0)	
VATS	68 (22.4)	41 (26.8)	27 (17.9)		13 (25.0)	8 (15.4)	
RATS	82 (27.0)	35 (22.9)	47 (31.1)	0.10	13 (25.0)	18 (34.6)	0.37
Oxygen requirement							
No	254 (83.6)	133 (86.9)	121 (80.1)		43 (82.7)	46 (88.5)	
Yes	50 (16.4)	20 (13.1)	30 (19.9)	0.11	9 (17.3)	6 (11.5)	0.40
Fever							
No	272 (89.5)	137 (89.5)	135 (89.4)		43 (82.7)	48 (92.3)	
Yes	32 (10.5)	16 (10.5)	16 (10.6)	0.97	9 (17.3)	4 (7.7)	0.14
Atelectasis							
No	295 (97.0)	150 (98.0)	145 (96.0)		51 (98.1)	51 (98.1)	
Yes	9 (3.0)	3 (2.0)	6 (4.0)	0.30	1 (1.9)	1 (1.9)	1.00
Pneumothorax							
No	75 (24.7)	27 (17.6)	48 (31.8)		11 (21.2)	16 (30.8)	
Yes	229 (75.3)	126 (82.4)	103 (68.2)	0.004	41 (78.8)	36 (69.2)	0.26
Bronchoscopy toilette							
No	278 (91.4)	145 (94.8)	133 (88.1)		47 (90.4)	47 (90.4)	
Yes	26 (8.6)	8 (5.2)	18 (11.9)	0.04	5 (9.6)	5 (9.6)	1.00
Re-hospitalization							
No	290 (95.4)	144 (94.1)	146 (96.7)		49 (94.2)	50 (96.2)	
Yes	14 (4.6)	9 (5.9)	5 (3.3)	0.28	3 (5.8)	2 (3.8)	0.65
Hospital stay							
Median [range]	5.0 [2–19]	4.0 [2–18]	5.0 [2–19]	0.17	4.5 [2–18]	5.0 [2–12]	0.82
Drainage							
Median [range]	4.0 [1–18]	3.0 [1–16]	4.0 [1–18]	0.03	3.0 [1–16]	4.0 [1–12]	0.49

**Table 8:** Clinical pathological findings.

	Total (n = 158)	0.291 *p* Value (HR 95%)
Sex		
Female	59 (37.3)	
Male	99 (62.7)	
Tobacco exposure	48 (30.4)	0.934
Diabetes	28 (17.7)	0.124
Tumor location		
LLL	32 (20.3)	0.633
LUL	42 (26.6)	
RLL	26 (16.5)	
ML	11 (7)	
RUL	45 (28.5)	
Right hilum	1 (0.6)	
Left hilum	1 (0.6)	
cN1	46 (29.1)	0.05
Kind of resection		
Lobectomy	131 (82.9)	
Bilobectomy/Sleeve	7 (4.4)	
Pneumonectomy	16 (10.1)	
Segmentectomy	5 (2.5)	
pT dimension		
<2	44 (27.8)	0.215
2–3 month	46 (29.1)	
3–5 month	40 (25.3)	
>5	27 (17.1)	
Missing	1 (0.6)	
Histology		
Adenocarcioma	102 (27.8)	0.19
Squamous cell carcinoma	40 (25.3)	
Other	16 (10.1)	
STAS	26 (16.5)	0.457
Pleural infiltration	67 (42.4)	0.861
Lymphatic invasion	46 (29.1)	0.3
Vascular invasion	47 (29.1)	0.422
Kind of lymphadenectomy		
Lobe specific	52 (32.9)	0.848
Radical dissection	106 (67.1)	
N hilar resected station		
0–1	37 (23.4)	0.155
>1	121 (76.6)	
N hilar metastatic station		
0–1	139 (88)	0.495
>1	19 (12)	
N hilar resected nodes		
0–3	25 (15.8)	0.118
≥	133 (84.2)	
N hilar metastatic nodes		
0–3	89 (56.3)	0.102
≥3	69 (43.7)	
N mediastinal resected station		
0–3	75 (47.5)	0.23
≥3	83 (52.5)	
Adjuvant therapy		
No	51 (32.3)	0.133
Yes	81(51.3)	
Missing	26 (16.5)	

**Table 9:** Characteristics of NSCLC patients who underwent pulmonary lobectomy.

CHARACTERISTICS	NOT READMITTED N = 271	READMITTED N = 9	*p* Value
Sex, n (%) M F	161 (59%) 110 (41%)	6 (67%) 3 (33%)	0.74
Smoke history, n (%) No Yes	48 (18%) 223 (82%)	1 (11%) 8 (89%)	1
Actual smoker, n (%) No Yes	138 (51%) 86 (32%)	7 (78%) 1 (11%)	0.26
Coronary heart disease, n (%) No Yes	238 (88%) 33 (12%)	8 (89%) 1 (11%)	1
IPA, n (%) No Yes	156 (58%) 115 (42%)	5 (56%) 4 (44%)	1
COPD, n (%) No Yes	232 (86%) 39 (14%)	7 (78%) 2 (22%)	0.62
ASA score, n (%) 1 2 3 4	11 (4%) 131 (48%) 128 (47%) 1 (1%)	0 2 (22%) 7 (78%) 0	0.26
Neoadjuvant therapy, n (%) No Yes	265 (98%) 6 (2%)	9 (100%) 0	1
Surgical side, n (%) Right Left	177 (65%) 94 (35%)	5 56(%) 4 (44%)	0.72
Pulmonary lobe, n (%) Superior Inferior	179 (66%) 92 (35%)	6 (67%) 3 (33%)	1
En bloc resection, n (%) No Yes	269 (99%) 2 (1%)	9 (100%) 0	1
Sleeve lobectomy, n (%) No Yes	270 (99%) 1 (1%)	9 (100%) 0	1
Histology, n (%) ADK Squamo Altro	214 (79%) 52 (19%) 5 (2%)	7 (78%) 2 (22%) 0	0.73
pT, n (%) is la(mi) la lb lc 2 2a 2b 3	1 (1%) 0 (0%) 24 (8%) 86 (32%) 48 (18%) 2 (1%) 66 (24%) 20 (7%) 24 (9%)	0 1 (11%) 0 2 (22%) 1 (11%) 0 2 (22%) 1 (11%) 2 (22%)	0.08
pN, n (%) 0 1	246 (91%) 20 (9%)	8 (89%) 1 (11%)	0.51
pSTAGE, n (%) 1 2	202 (75%) 64 (24%)	5 (56%) 4 (44%)	0.23
Last GB trend, n (%) Reduction Increase	203 (75%) 68 (25%)	7 (78%) 2 (22%)	1
Last CRP trend, n (%) Reduction Increase	136 (50%) 135 (50%)	5 (56%) 4 (44%)	1
Infective complications, n (%) No Yes	252 (93%) 19 (7%)	8 (89%) 1 (11%)	0.49

**Table 10:** DY in patients with free pleural fluid and no pleural fluid: 88.9% vs. 75%; chi-quadro 2.9481; *p*-value 0.086.

	Diagnostic	Not Diagnostic	
Free pleural fluid	105	13	118
No pleural fluid	15	5	20
	120	18	138 (total)

**Table 11:** Preoperative assessment.

Iter	Evaluation	Description
Diagnostic-Therapeutic Iter	Thoracic Surgeon	Remote medical history Total body CT with contrast PET with 18F-FDG
	Multidisciplinary Discussion (“Tumor Board”)	
Pre-operative Iter: Step I	Pulmonologist	Spirometry DLCO Blood gas analysis
	Cardiologist (if cardiac patient)	ECG Echocardiogram
	Anesthesiologist	Blood chemistry tests (coagulation, CBC, electrolytes)
Pre-operative Iter: Step II	Pulmonologist	6-min walking test Perfusion scintigraphy
	Cardiologist (if cardiac patient)	ECG-stress test Pharmacological Echo-stress
	Specialty Examinations by Anesthesiologist	For example, fibrolaryngoscopy for intubation evaluation
Pre-operative Iter: Step III	Pulmonologist	Myocardial scintigraphy Coronary angiography
	Cardiologist	Cardiopulmonary stress test
	Specialty Evaluations if Needed	

**Table 12:** Clinical characteristics.

	PET − (Group 1)	PET + (Group 2)
Absence of PTs	3 (75.0%)	7 (38.9%)
Malignant cells in pleural fluid	3 (75.0%)	11 (61.1%)
Asbestos exposure	2 (50.0%)	11 (61.1%)
Other pneumotoxic agents exposure	3 (75.0%)	16 (88.9%)
Smoking history	2 (50.0%)	8 (44.5%)

**Table 13:** Surgical features.

	Thoracotomy (n = 12)	Uniportal VATS (n = 10)
Sex		
M	11 (92%)	6 (60%)
F	1 (8%)	4 (40%)
Age at surgery (Mean ± SD)	56.7 ± 16.1	67.1 ± 12.2
Intervention duration (min) (Mean ± SD)	95 ± 57.7	73.5 ± 35.5
Drainage duration (days) (Mean ± SD)	3.5 ± 0.6	2.9 ± 0.3
Major complications	0	0
Post-surgical air leakage	0	0
Hospitalization (days) (Mean ± SD)	3.8 ± 0.9	2.9 ± 0.3
Origin of lung metastasis		
Lower gastrointestinal tumor	11 (92%)	7 (70%)
Soft tissue sarcoma	1 (8%)	2 (20%)
Cervix cancer		1 (10%)
Metastasis		
Single	4 (33%)	7 (70%)
Multiple	8 (67%)	2 (20%)
Major lesion size (cm) (Mean ± SD)	1.1 ± 0.8	1.6 ± 0.4
Surgical margin (cm) (Mean ± SD)	0.7 ± 0.5	0.5 ± 0.1
Relapse of previous resection	0	1 (10%)
Smoker		
NO	1 (8%)	5 (50%)
Yes	3 (25%)	1 (10%)
Former	8 (67%)	4 (40%)
COPD	2 (17%)	0
Diabetes	2 (17%)	1 (10%)
Cardiovascular disease	2 (17%)	6 (60%)

**Table 14:** Univariate analysis.

	U-VATS	Open Approach	*p*-Value
Age at surgery (Mean ± SD)	67.1 (12.2)	56.7 ± 16.1	0.111
Sex			
M	6 (60%)	11 (92%)	0.135
F	4 (40%)	1 (8%)	
Intervention duration (min) (Mean ± SD)	73.5 ± 35.5	95 ± 57.7	**0.025**
Hospitalization (days) (Mean ± SD)	2.9 ± 0.3	3.8 ± 0.9	**0.015**
Drainage duration (days)			0.076
2	1 (10%)		
3	9 (90%)	7 (58.3%)	
4		4 (33.3%)	
5		1 (8.3%)	
Smoke			0.115
NO	5 (50%)	1 (8%)	
Yes	1 (10%)	3 (25%)	
Former	4 (40%)	8 (67%)	
Cardiovascular disease			0.074
NO	4 (40%)	10 (83.3%)	
YES	6 (60%)	2 (16.7%)	
COPD			0.481
NO	10(100%)	10 (83.3%)	
YES		2 (16.7%)	
Diabetes			1
NO	9(90%)	10 (83.3%)	
YES	1 (10%)	2 (16.7%)	
Number of metastases (Mean ± SD)	1.9 (2.3)	3.7 (2.7)	0.133
Dimension (cm) (Mean ± SD)	1.7 (0.4)	1.1 (0.8)	0.166
Surgical margin (cm) (Mean ± SD)	0.5 (0.1)	0.7 (0.5)	0.36

**Table 15:** Primary pleural angiosarcoma epidemiology and clinical, diagnostic and therapeutic features. The total number of cases is 47, unless otherwise specified.

Age (Mean ± SD)	61.8 ± 15.3 Years
Sex	Males 39 Females 10
Symptoms	Dyspnoea 22 Chest pai 15 Haemoptysis 6 Cough 5 No respiratory symptoms 2
Pleural effusion	No 11 (23.9%) Monolateral 26 (56.5%) Bilateral 9 (19.6%) Hematic effusion 17 (48.6%) Non hematic effusion 18 (51.4%)
Pleural effusion citology, when performed	Diagnostic 3 (11.1%) Not Diagnostic 24 (88.9%)
CT specific findings, when performed	Yes 24 (57.1%) No 18 (42.9%)
Diagnostic procedure	Thoracoscpy and pleural biopsy 33 (71.7%) Autopsy 7 (15.3%) CT guided biopsy 6 (13%)
Ttreatment	No therapy 11 (27.5%) Chemotherapy 18 (45%) Radiotherapy 3 (7.5%) Surgery 7 (17.5%) Palliative care 11(27.5%)
Survival from diagnosis (mean ± SD)	20 ± 29.2 weeks

## Supplementary Materials

The following supporting information can be downloaded at: https://www.mdpi.com/article/10.3390/jcm13195954/s1. The affiliation information of all abstracts’ authors can be found in the Supplementary File.
