# Supplementary material for: Abstracts of the Italian Society of Thoracic Endoscopy (SIET) 2024 Annual Congress
Source: J Clin Med. 2024 Oct 7;13(19):5954. doi: 10.3390/jcm13195954 (PMC11477479; doi:10.3390/jcm13195954)
Supplement: Supplementary file 1 [file jcm-13-05954-s001.zip › jcm-3222064-Supplementary Materials.pdf]

Abbate	Tommaso	Interventional Pulmonology Unit, IRCCS Azienda Ospedaliero-Universitaria di Bologna, Italy
Accordino	Francesca	Università degli Studi di Firenze - Azienda Ospedaliero Universitaria di Careggi
Albrici	Cristina	Respiratory Unit, ASST Santi Paolo e Carlo, San Paolo Hospital, Department of Health Sciences, University of Milan, Milan, Italy
Alloisio	Marco	Thoracic Surgery Unit, Humanitas Hospital, Milan, Italy
Alloisio	Antonella	U.O.C. Chirurgia Toracica, IRCCS Ospedale Policlinico San Martino, Genova
Amatucci	Riccardo	Department of Thoracic Surgery, University of Perugia Medical School, Perugia, Italy
Ambrogi	Vincenzo	Thoracic Surgery Unit, Tor Vergata University Policlynic, Viale Oxford 81, 00133 Rome, Italy
Ambrosi	Francesca	Pathology Unit, Maggiore Hospital AUSL, Bologna, Italy, Department of experimental, diagnostic and specialist medicine (DIMES), University of Bologna, Bologna, Italy
Amore	Dario	AO dei Colli, Monaldi Hospital Thoracic Surgery Unit (NA) ITALY
Amores Naranjo	Karen	Department of Diagnostic and Specialty Medicine, University of Bologna, Thoracic Surgery Unit, Morgagni-Pierantoni Hospital, Forli, Italy
Anastasi	Andrea	Department of Mental Health, Fondazione IRCCS Ca' Granda Ospedale Maggiore Policlinico, Milan, Italy
Ancona	Gianluca	Division of Thoracic Surgery, ASST Valtellina e Alto Lario, "Eugenio Morelli" Hospital, 23035 Sondalo, Italy
Andreani	Alessandro	UOC Pneumologia Mirandola ASL Modena (Italy)
Andreetti	Claudio	Thoracic Surgery Sant'Andrea Hospital, La Sapienza University, Rome, Italy
Andriolo	Luigi Gaetano	Unità Operativa Complessa di Chirurgia Toracica, Ospedale Vito Fazzi, Lecce
Angelescu	Dan	Thoracic Surgery Division, Périgueux General Hospital, France
Anile	Marco	UOC Chirurgia Toracica e Trapianti di Polmone, Dipartimento di Chirurgia Generale e Specialistica, Sapienza Università di Roma
Aprile	Vittorio	Thoracic Surgery Unit, Università di Pisa, Pisa, Italy
Arbore	Alessia	Thoracic Surgery Unit, Cattinara University Hospital, Trieste, Italy
Arbore	Enrico	Thoracic Surgery Unit, Cattinara University Hospital, Trieste, Italy
Arcoleo	Giuseppe	A.O.O.R. Villa Sofia—Cervello—Palermo (Italy)
Ardò	Nicoletta Pia	Università di Foggia, Azienda ospedaliero-universitaria "Policlinico Riuniti" Foggia, Struttura Complessa di Chirurgia Toracica Universitaria, Foggia-Italy
Argnani	Desideria	Department of Diagnostic and Specialty Medicine, University of Bologna, Thoracic Surgery Unit, Morgagni-Pierantoni Hospital, Forli, Italy
Astaneh	Arash	Department of Thoracic Surgery, ASST Grande Ospedale Metropolitano Niguarda, Milano, Italy
Aulino	Rita	UOC Chirurgia toracica, Azienda ospedaliero universitario Policlinico-San Marco, Catania, Italy
Balsamo	Ludovica	U.O.C. Chirurgia Toracica, IRCCS Ospedale Policlinico San Martino, Genova
Bandelli	Gian Piero	Interventional Pulmonology Unit, IRCCS Azienda Ospedaliero-Universitaria di Bologna, Bologna Italy
Bandiera	Alessandro	Department of Thoracic Surgery, IRCCS Scientific Institute San Raffaele, Milan
Bargagli	Elena	Respiratory Diseases Unit, Department of Medical and Surgical Sciences and Neuro- Sciences, University of Siena, 53100 Siena, Italy
Barra	Floriana	Diagnostic Imaging Unit, Department of Medical, Surgical and Neuro Sciences and of Medical Sciences, University of Siena, Azienda Ospedaliero-Universitaria Senese, Siena, Italy
Bassi	Massimiliano	Department of Thoracic Surgery and Lung Transplantation, Sapienza University of Rome, Italy
Bastone	Sebastiano Angelo	Thoracic Surgery Unit, Tor Vergata University Policlynic, Viale Oxford 81, 00133 Rome, Italy
Bellettati	Claudia	Dipartimento di Chirurgia Toracica, Fondazione Policlinico Universitario Agostino Gemelli, IRCCS, 00168, Roma
Bellini	Alice	Department of Diagnostic and Specialty Medicine, University of Bologna, Thoracic Surgery Unit, Morgagni-Pierantoni Hospital, Forli, Italy
Benci	Elisabetta	Thoracic Surgery Unit, Cattinara University Hospital, Trieste, Italy
Beneduce	Lucia	U.O.C. di Chirurgia Toracica, A.O.R.N. "A.Cardarelli" Napoli, Italia
Bennett	David	Respiratory Diseases Unit, Department of Medical and Surgical Sciences and Neuro- Sciences, University of Siena, 53100 Siena, Italy
Benuzzi	Ludovica	Università degli Studi di Firenze - Azienda Ospedaliero Universitaria di Careggi
Bertoglio	Pietro	Division of Thoracic Surgery, IRCCS Azienda Ospedaliera Universitaria Bologna, Bologna, Italy
Bertolaccini	Luca	Division of Thoracic Surgery, IEO, European Institute of Oncology, IRCCS, Milan, Italy
Bertuccio	Francesco Rocco	Cardiothoracic and Vascular Department, Unit of Respiratory Diseases, IRCCS Policlinico San Matteo, Pavia, Italy
Bevilacqua	Maria Teresa	Università di Foggia, Azienda ospedaliero-universitaria "Policlinico Riuniti" Foggia, Struttura Complessa di Chirurgia Toracica Universitaria, Foggia, Università degli Studi di Bari, Azienda ospedaliero-universitaria "Policlinico Consorziale" Bari-Italy
Biagini	Margherita	Pulmonary Unit, Cardiothoracic and Vascular Department, University of Pisa, Pisa, Italy
Bianco	Andrea	Department of Translational Medicine, University of Campania "L. Vanvitelli", Naples, Italy
Bitetto	Damaride Micaela	IRCCS Azienda Ospedaliero-Universitaria di Bologna, University Hospital Sant'Orsola- Malpighi—Respiratory and Critical Care Unit, Bologna, Italy, Alma Mater Studiorum, Department of Medical and Surgical Sciences (DIMEC), University of Bologna, Italy
Bogina	Giuseppe	Pathology Anatomy, IRCCS Sacro Cuore-Don Calabria Hospital, Verona, Italy
Bombelli	Sara	Department of Pneumology—Grande Ospedale Metropolitano Niguarda—Milan (Italy), Post Graduate School in Respiratory Medicine, Università degli Studi di Milano, Master's Degree in Interventional Pneumology—Università degli Studi di Firenze, Italy

Boniello	Esterina	U.O.C. Malattie apparato respiratorio, Policlinico di Bari, Bari, Italia
Bonini	Matteo	Department of Public Health and Infectious Diseases, Sapienza University of Rome, Italy
Bonis	Alessandro	Padova University Hospital—Thoracic Surgery Unit, Padova, Italy
Borrata	Francesco	Thoracic Surgery Unit, University Hospital-Policlinico-San Marco, Catania, Italy
Bosio	Matteo	Cardiothoracic and Vascular Department, Unit of Respiratory Diseases, IRCCS Policlinico San Matteo, Pavia, Italy
Bottoli	Maria Caterina	ASST Carlo Poma Mantova, Mantova
Bounou	Mehdi	La Province Laboratory, Kénitra, Morocco
Bove	Mary	Thoracic Surgery Unit, Istituto Nazionale Tumori IRCCS Fondazione G. Pascale, Naples, Italy
Brascia	Debora	Thoracic Surgery Unit, Department of Precision and Regenerative Medicine and Ionian Area, University of Bari "Aldo Moro", Bari, Italy
Bucchi	Chiara	IRCCS Azienda Ospedaliero-Universitaria di Bologna, University Hospital Sant'Orsola-Malpighi—Respiratory and Critical Care Unit, Bologna, Italy; Alma Mater Studiorum, Department of Medical and Surgical Sciences (DIMEC), University of Bologna, Italy
Buonocore	Giovanni	Pulmonary Unit, Cardiothoracic and Vascular Department, University of Pisa, Pisa, Italy
Busiello	Luigi	U.O.C. di Chirurgia Toracica, A.O.R.N. "A.Cardarelli" Napoli, Italia
Caffarena	Giovanni	Department of Thoracic Surgery, IEO, European Institute of Oncology IRCCS, Milan, Italy
Cagini	Lucio	Department of Thoracic Surgery, Ospedale del Mare, ASL NA1 Centro, Naples, Italy
Cagnetti	Sara	Division of Thoracic Surgery, ASST Valtellina e Alto Lario, "Eugenio Morelli" Hospital, 23035 Sondalo, Italy
Calabrese	Giuseppe	Department of Thoracic Surgery, Fondazione Policlinico Universitario A.Gemelli IRCCS, Università Cattolica del Sacro Cuore, Rome, Italy
Calvello	Mariarosaria	Lung Unit, S. Filippo Neri Hospital, ASLROMA1, Rome
Calvo	Damiano	Thoracic Surgery Unit, University Hospital-Policlinico-San Marco, Catania, Italy
Camarda	Noemi	University of Modena and Reggio Emilia
Campanella	Annalisa	Dipartimento di Chirurgia Toracica, Fondazione Policlinico Universitario Agostino Gemelli, IRCCS, 00168, Roma
Campisi	Alessio	Thoracic Surgery Department, University and Hospital Trust—Ospedale Borgo Trento, Verona, Italy
Candoli	Piero	Interventional Pulmonology Unit, IRCCS Azienda Ospedaliero-Universitaria di Bologna, Bologna Italy
Cannone	Giorgio	Padova University Hospital—Thoracic Surgery Unit, Padova, Italy
Cantatore	Mirko Giro-lamo	Thoracic Surgery Unit, Department of Precision and Regenerative Medicine and Ionian Area, University of Bari "Aldo Moro", Bari, Italy
Capasso	Francesca	UOC Chirurgia Toracica Università degli studi della Campania 'Luigi Vanvitelli', Napoli
Caporale	Domenico	Department of Thoracic Surgery, IRCCS-CROB Centro di Riferimento Oncologico della Basilicata, Rionero in Vul-ture, PZ, Italy
Capuano	Alessandro	Institute of Respiratory Disease, University of Medicine "Aldo Moro"—Bari (BA)
Caputo	Alessia	UOC Chirurgia Toracica Università degli studi della Campania 'Luigi Vanvitelli', Napoli
Cara	Andrea	Division of Thoracic Surgery, Fondazione IRCCS San Gerardo dei Tintori, Monza, Italy
Carbonara	Maria Caterina	Unità Operativa Complessa di Nefrologia, Ospedale Vito Fazzi, Lecce
Carbone	Luigi	San Camillo Forlanini Hospital, Rome, Italy
Cardillo	Giuseppe	San Camillo Forlanini Hospital, Rome, Italy
Carillo	Carolina	Department of Thoracic Surgery and Lung Transplantation, Sapienza University of Rome, Italy
Carleo	Francesco	San Camillo Forlanini Hospital, Rome, Italy
Carleo	Graziana	Thoracic Surgery Unit, Department of Precision and Regenerative Medicine and Ionian Area, University of Bari "Aldo Moro", Bari, Italy
Carli	Serena Maria	UOC Malattie infettive dell'apparato respiratorio, IRCCS INMI Spallanzani-Rome (Italy)
Carlucci	Annalisa	Department of Thoracic Surgery, IRCCS-CROB Centro di Riferimento Oncologico della Basilicata, Rionero in Vul-ture, PZ, Italy
Carlucci	Roberto	Intensive Care Unit, S. Filippo Neri Hospital, ASLROMA1, Rome
Carpagnano	Giovanna Elisiana	U.O.C. Malattie apparato respiratorio, Policlinico di Bari, Bari, Italia
Carretta	Angelo	ASST Carlo Poma Mantova, Mantova
Carrozzi	Laura	Department of Surgical, Medical, and Molecular Pathology and Critical Care Medicine, University of Pisa, Pisa, It-aly
Cartucci	Paolo	Respiratory Unit, ASST Santi Paolo e Carlo, San Paolo Hospital, Department of Health Sciences, University of Mi-lan, Milan, Italy
Casali	Christian	Thoracic Surgery Unit, Baggiovara Hospital of Modena, Modena, Italy
Casalini	Eleonora	Pulmonology Unit, Azienda Unità Sanitaria Locale-IRCCS di Reggio Emilia—Reggio Emilia (RE)
Casazza	Dino	AO dei Colli, Monaldi Hospital Thoracic Surgery Unit (NA) ITALY
Casciani	Cristiano	Departement of Surgical Sciences, University of Rome Tor Vergata, Via Montpellier 1, 00133, Rome, Itay
Cascina	Alessandro	Cardiothoracic and Vascular Department, Unit of Respiratory Diseases, IRCCS Policlinico San Matteo, Pavia, Italy
Cascone	Roberto	UOC Chirurgia Toracica, IRCCS Centro di Riferimento Oncologico della Basilicata, Rionero in Vulture (Italy)
Casiraghi	Monica	Department of Thoracic Surgery, IEO, European Institute of Oncology IRCCS, Milan, Italy
Cassina	Enrico Mario	Department of Thoracic Surgery, Fondazione IRCCS San Gerardo dei Tintori, 20900 Monza, Italy
Catelli	Chiara	Lung Transplant Unit, Department of Medical, Surgical and Neuro Sciences, Azienda Ospedaliero-Universitaria Senese, University of Siena, 53100 Siena, Italy
Cavaleri	Marco	Servizio di anestesia 1, Azienda ospedaliero universitario Policlinico-San Marco, Catania, Italy

Cavaliere	Ilenia	Division of Thoracic Surgery, Cardio-Thoracic-Vascular Departement, Hospital of Cosenza "SS Annunziata", Cosenza, Italy
Cavalli	Erica Michela	Division of Plastic Surgery, Fondazione IRCCS San Gerardo dei Tintori, Monza, Italy
Ceccarelli	Ilaria	Minimally Invasive and Robotic Thoracic Surgery—Surgical, Medical, Molecular and Critical Care Pathology Department, University Hospital of Pisa
Ceccarelli	Marco	Department of Industrial Engineering, University of Rome Tor Vergata, Rome, Italy
Centanni	Stefano	Respiratory Unit, ASST Santi Paolo e Carlo, San Paolo Hospital, Department of Health Sciences, University of Milan, Milan, Italy
Centofanti	Anastasia	UOC Chirurgia Toracica e Trapianti di Polmone, Dipartimento di Chirurgia Generale e Specialistica, Sapienza Università di Roma
Cesaro	Cristiano	Bronchology Unit, Monaldi Hospital, Naples, Italy
Charrier	Thomas	Thoracic Surgery Unit, APHP, Centre Université de Paris Cité, site Cochin, Paris, France
Chiappetta	Marco	Department of General Thoracic Surgery, Fondazione Policlinico Universitario“A.Gemelli”, IRCCS, Università Cattolica del Sacro Cuore, Rome, Italy
Chiappetta	Caterina	Dipartimento di Scienze Medico-Chirurgiche e Biotecnologie, Sapienza Università di Roma
Chiari	Matteo	Division of Thoracic Surgery, IEO, European Institute of Oncology, IRCCS, Milan, Italy; Department of Oncology and Hemato-Oncology, University of Milan, Milan, Italy
Chirillo	Angelo	Division of Radiotherapy, Onco-Hematology Departement, Hospital of Cosenza "SS Annunziata", Cosenza, Italy
Cialdella	Francesca	Università di Foggia, Azienda ospedaliero-universitaria “Policlinico Riuniti” Foggia, Struttura Complessa di Chirurgia Toracica Universitaria, Foggia-Italy
Cialì Sposato	Luciano	Thoracic Surgery Unit, Tor Vergata University Policlynic, Viale Oxford 81, 00133 Rome, Italy
Cianci	Roberta	Department of Translational Medicine, University of Campania “L. Vanvitelli”, Naples, Italy
Ciani	Luca	Pneumologia Interventistica, AOU Careggi, Firenze
Ciarrocchi	Angelo Paolo	Department of Diagnostic and Specialty Medicine, University of Bologna, Thoracic Surgery Unit, Morgagni-Pierantoni Hospital, Forli, Italy
Ciccone	Anna Maria	Thoracic Surgery Sant’Andrea Hospital, La Sapienza University, Rome, Italy
Cinel	Jacqueline	Department of Thoracic Surgery, Ospedale Santa Maria della Misericordia, Udine, Italy
Ciocan	Catalina	Occupational Medicine Division, Laboratory of Toxicology and Industrial Epidemiology, Department of Public Health and Paediatrics, University of Turin, Turin, Italy
Cipolla	Giuseppe	UOC Pneumologia AST Macerata, Ospedale Generale di Macerata (Italy)
Ciriaco	Paola	Department of Thoracic Surgery, IRCCS Scientific Institute San Raffaele, Milan
Civello	Jacopo	Department of Pneumology—Grande Ospedale Metropolitano Niguarda—Milan (Italy), Post Graduate School in Respiratory Medicine, Università degli Studi di Milano
Colafigli	Claudia	Interventional Radiology Perugia
Colella	Sara	UOC Pneumologia Teramo (Italy)
Comacchio	Giovanni Maria	Padova University Hospital—Thoracic Surgery Unit, Padova, Italy
Combattelli	Chiara	Thoracic Surgery Unit, Tor Vergata University Policlynic, Viale Oxford 81, 00133 Rome, Italy
Conforti	Serena	Department of Thoracic Surgery, ASST Grande Ospedale Metropolitano Niguarda, Milano, Italy
Congedo	Maria Teresa	Department of Thoracic Surgery, Fondazione Policlinico Universitario A.Gemelli IRCCS, Università Cattolica del Sacro Cuore, Rome, Italy
Congiu	Stefano	Thoracic Surgery, University Hospital Maggiore della Carita, Novara, Italy
Contino	Simone	Respiratory Unit, ASST Santi Paolo e Carlo, San Paolo Hospital, Department of Health Sciences, University of Milan, Milan, Italy
Corbetta	Lorenzo	Pneumologia Interventistica, AOU Careggi, Firenze
Corsico	Angelo Guido	Department of Internal Medicine and Medical Therapeutics, University of Pavia, Italy, Cardiothoracic and Vascular Department, Unit of Respiratory Diseases, IRCCS Policlinico San matteo, Pavia, Italy
Cortale	Maurizio	Thoracic Surgery Unit, Cattinara University Hospital, Trieste, Italy
Corzani	Roberto	Lung Transplant Unit, Department of Medical, Surgical and Neuro Sciences, Azienda Ospedaliero-Universitaria Senese, University of Siena, 53100 Siena, Italy
Costa	Fabio	Department of Anesthesia and Intensive Care, Fondazione Policlinico Universitario Campus Bio-Medico, Rome
Coviello	Eleonora	San Camillo Forlanini Hospital, Rome, Italy; University of Perugia, Perugia, Italy
Criscione	Alessandra	Thoracic Surgery Unit, University Hospital-Policlinico-San Marco, Catania, Italy
Crucitti	Pierfilippo	Department of Thoracic Surgery, Fondazione Policlinico Universitario Campus Bio-Medico, Rome
Cuccaro	Ilaria	Division of Pulmonary Medicine, Sandro Pertini Hospital—Rome (Italy)
Curcio	Carlo	Pineta Grand Hospital, Thoracic Surgery Unit, Castelvolturno (CE) ITALY
Curto	Isacco	Terapia Intensiva Neurochirurgica, Ospedale Santa Chiara, Trento
Cusumano	Giacomo	Thoracic Surgery Unit, University Hospital-Policlinico-San Marco, Catania, Italy
D’Agnano	Vito	Department of Translational Medicine, University of Campania “L. Vanvitelli,” Naples, Italy
D’Alessandro	Miriana	Respiratory Diseases Unit, Department of Medicine, Surgery and Neurosciences, University of Siena, 53100 Siena, Italy
D’Andrilli	Antonio	Thoracic Surgery Sant’Andrea Hospital, La Sapienza University, Rome, Italy
D’Aucelli	Loredana	Thoracic Surgery Unit, Department of Precision and Regenerative Medicine and Ionian Area, University of Bari “Aldo Moro”, Bari, Italy
Dahan	Marcel	Thoracic Surgery Division, Toulouse University Hospital, France
D'Alagni	Giancarlo	U.O.C. Pneumologia, Ospedale San Giuseppe Moscati, Taranto, Italia

D'Ambrosio	Gioacchino	Pathology Unit, Department of Diagnostical Services and Imaging, IRCCS Policlinico San Matteo, Pavia
Damiani	Stefania	Pathology Unit, IRCCS Azienda Ospedaliero-Universitaria di Bologna, Department of Medical and Surgical Science (DIMEC), Italy
D'Antoni	Letizia	Department of Public Health and Infectious Diseases, Sapienza University of Rome, Italy
Danuzzo	Federica	University of Milan, Milan, Italy; Department of Thoracic Surgery, Fondazione IRCCS San Gerardo dei Tintori, 20900 Monza, Italy
Davini	Federico	Minimally Invasive and Robotic Thoracic Surgery—Surgical, Medical, Molecular and Critical Care Pathology Department, University Hospital of Pisa
De Bellis	Roberto	Università di Foggia, Azienda ospedaliero-universitaria “Policlinico Riuniti” Foggia, Struttura Complessa di Chirurgia Toracica Universitaria, Foggia-Italy
De Blasi	Francesco	Unit of Thoracic Surgery, Department of Precision and Regenerative Medicine and Ionian Area, University of Bari, Bari, Italy
De Franceschi	Elisa	Department of Thoracic Surgery, Ospedale Santa Maria della Misericordia, Udine, Italy
De Giacomo	Tiziano	UOC Chirurgia Toracica e Trapianti di Polmone, Dipartimento di Chirurgia Generale e Specialistica, Sapienza Università di Roma
De Luca	Giuseppe	Thoracic Surgery Unit, Istituto Nazionale Tumori IRCCS Fondazione G. Pascale, Naples, Italy
De Meo	Michela	Dipartimento di Medicina Molecolare, Dipartimento di Chirurgia Generale e Specialistica, Sapienza Università di Rom
De Palma	Angela	Unit of Thoracic Surgery, Department of Precision and Regenerative Medicine and Ionian Area, University of Bari, Bari, Italy
De Renzi	Gianluigi	Dipartimento di Medicina Molecolare, Dipartimento di Chirurgia Generale e Specialistica, Sapienza Università di Rom
De Rosa	Marino	Lung Unit, S. Filippo Neri Hospital, ASLROMA1, Rome
Del Bene	Massimo	Division of Plastic Surgery, Fondazione IRCCS San Gerardo dei Tintori, Monza, Italy
Del Regno	Laura	Dermatology Unit, Fondazione Policlinico Universitario A. Gemelli IRCCS, Università Cattolica del Sacro Cuore, Rome, Italy
Dell’Amore	Andrea	Padova University Hospital—Thoracic Surgery Unit, Padova, Italy
Della Beffa	Eleonora	Thoracic Surgery Unit, Università di Torino, Turin, Italy
Della Morte	Aniello	U.O.C. di Chirurgia Toracica, A.O.R.N. “A.Cardarelli” Napoli, Italia
Dell'Anna	Vladimiro	Pathological Anatomy Operational Unit, Vito Fazzi Hospital, Lecce
Di Filippo	Vincenzo	UOC Chirurgia Toracica Università degli studi della Campania ‘Luigi Vanvitelli’, Napoli
Di Fonzo	Riccardo	Thoracic Surgery Unit, San Raffaele Hospital, Milan, Italy
Di Martino	Marco	San Camillo Forlanini Hospital, Rome, Italy
Di Matteo	Evelyn	Campus Bio-Medico University Hospital Foundation of Rome, Santa Scolastica Hospital of Cassino
Di Nunzio	Francesca	Campus Bio-Medico University Hospital Foundation of Rome, Santa Scolastica Hospital of Cassino
Di Raimondo	Francesco	Unità operativa di Ematologia 1, Azienda ospedaliero universitario Policlinico-San Marco, Catania, Italy
Di Rienzo	Alberto	Department of Thoracic Surgery, Ospedale Santa Maria della Misericordia, Udine, Italy
Di Stasio	Mario	Department of Thoracic Surgery, Ospedale del Mare, ASL NA1 Centro, Naples, Italy
Di Stefani	Alessandro	Dermatology Unit, Fondazione Policlinico Universitario A. Gemelli IRCCS, Università Cattolica del Sacro Cuore, Rome, Italy
Diotti	Cristina	Department of Thoracic Surgery, IEO, European Institute of Oncology IRCCS, Milan, Italy
Diso	Daniele	UOC Chirurgia Toracica e Trapianti di Polmone, Dipartimento di Chirurgia Generale e Specialistica, Sapienza Università di Roma
Dolcetti	Francesco	Arcispedale Sant’Anna, Ferrara, Italy
Dolci	Giampiero	Arcispedale Sant’Anna, Ferrara, Italy
D'Onofrio	Manuel	Department of Thoracic Surgery, University of Rome Tor Vergata, Rome, Italy
Drigo	Davide	Department of General Surgery, Cattinara University Hospital, Trieste, Italy
Dutau	Hervé	Department of Thoracic Oncology, Pleural Diseases and Interventional Pulmonology, APHM, Marseille, France
Esposito	Jessica	U.O.C. Pneumologia—”San Giovanni di Dio e Ruggi D’Aragona” University Hospital, Salerno, Italy
Evangelista	Jessica	Department of Thoracic Surgery, Fondazione Policlinico Universitario A.Gemelli IRCCS, Università Cattolica del Sacro Cuore, Rome, Italy
Evangelista	Antonio Pio	UOC Chirurgia Toracica e Trapianti di Polmone, Dipartimento di Chirurgia Generale e Specialistica, Sapienza Università di Roma
Fabiano	Antonio	Lung Transplant Unit, Department of Medical, Surgical and Neuro Sciences, Azienda Ospedaliero-Universitaria Senese, University of Siena, 53100 Siena, Italy
Fabietti	Giulia	Università degli Studi di Firenze - Azienda Ospedaliero Universitaria di Careggi
Facciolo	Francesco	Thoracic Surgery Unit,Istituto Nazionale Tumori “Regina Elena”, Rome, Italy
Facciolongo	Nicola Cosimo	Pulmonology Unit, Azienda Unità Sanitaria Locale-IRCCS—Reggio Emilia, Reggio Emilia, Italy
Failla	Giuseppe	Diagnostic and Therapeutic Bronchoscopy Unit, ARNAS "Civico e Benefratelli", Palermo, Italy
Fedele	Flavio	Division of Bronchology, Cardio-Thoracic-Vascular Departement, Hospital of Cosenza "SS Annunziata", Cosenza, Italy
Ferioli	Martina	Interventional Pulmonology Unit, IRCCS Azienda Ospedaliero-Universitaria di Bologna, Bologna Italy
Ferramosca	Emiliana	Unità Operativa Complessa di Nefrologia, Ospedale Vito Fazzi, Lecce
Ferrante	Francesco	Department of Thoracic Surgery, University of Rome Sapienza, Rome, Italy

Ferrari	Marco	Interventional Pulmonology Unit, IRCCS Azienda Ospedaliero-Universitaria di Bologna, Bologna Italy
Ferrazzo	Teresa	Department of Health Sciences, University “Magna Græcia” of Catanzaro, Catanzaro, Italy
Ferretti	Gianmaria	Division of Thoracic Surgery, SS. Annunziata Hospital, Taranto, Italy
Ferroni	Valentina	UOC Pneumologia AST Macerata, Ospedale Generale di Macerata (Italy)
Filosso	Pier Luigi	University of Modena and Reggio Emilia
Finamore	Panaiotis	Campus Bio-Medico University Hospital Foundation of Rome, Santa Scolastica Hospital of Cassino
Fino	Leonardo	Università di Foggia, Azienda ospedaliero-universitaria “Policlinico Riuniti” Foggia, Struttura Complessa di Chirurgia Toracica Universitaria, Foggia-Italy
Fiorelli	Alfonso	UOC Chirurgia Toracica Università degli studi della Campania ‘Luigi Vanvitelli’, Napoli
Flores	Krisstopher Richard	Division of Pulmonary Medicine, Sandro Pertini Hospital—Rome (Italy)
Fois	Alessandro G.	Department of Medicine, Surgery and Pharmacy, University of Sassari, Sassari, Italy, Clinical and Interventional Pneumology, University Hospital of Sassari, Sassari, Italy
Foltran	Gabriele	Respiratory Disease Unit, Department of Cardiac Thoracic and Vascular Scienses, Ospedale dell’Angelo, Venice, Italy.
Fontana	Emanuele	UOC Chirurgia toracica, Azienda ospedaliero universitario Policlinico-San Marco, Catania, Italy
Fontana	Davide Onofrio	Campus Bio-Medico University Hospital Foundation of Rome, Santa Scolastica Hospital of Cassino
Fontana	Matteo	Pulmonology Unit, Azienda Unità Sanitaria Locale-IRCCS—Reggio Emilia, Reggio Emilia, Italy
Fossi	Antonella	Respiratory Diseases Unit, Department of Medical and Surgical Sciences and Neuro- Sciences, University of Siena, 53100 Siena, Italy
Fournel	Ludovic	Thoracic Surgery Unit, APHP, Centre Université de Paris Cité, site Cochin, Paris, France
Frasca	Luca	Department of Thoracic Surgery, Fondazione Policlinico Universitario Campus Bio-Medico, Rome
Freda	Chiara	U.O.C. di Chirurgia Toracica, A.O.R.N. “A.Cardarelli” Napoli, Italia
Freynet	Olivia	AP-HP, Hôpitaux Universitaires Paris Seine-Saint-Denis, Hôpital Avicenne, Pneumologie,Université Sorbonne Paris Nord, Faculté de Médecine SMBH, Bobigny,France
Frongillo	Elisabettamaria	U.O.C. di Chirurgia Toracica, A.O.R.N. “A.Cardarelli” Napoli, Italia
Fumagalli	Giorgio	Lung Unit, S. Filippo Neri Hospital, ASLROMA1, Rome
Gabbrielli	Luciano	Pulmonary Unit, Cardiothoracic and Vascular Department, Pisa University Hospital, Pisa, Italy
Galasso	Thomas	Interventional Pulmonology Unit, IRCCS Azienda Ospedaliero-Universitaria di Bologna, Bologna Italy
Galgano	Angela	Thoracic Surgery Unit, Department of Medical, Surgical and Neuro Sciences, University of Siena, Azienda Ospedaliero-Universitaria Senese, Siena, Italy
Gallina	Filippo	Thoracic Surgery Unit,Istituto Nazionale Tumori “Regina Elena”, Rome, Italy
Gallo	Michele	A.O.O.R. Villa Sofia—Cervello—Palermo (Italy)
Gallo	Arianna	Dipartimento di Chirurgia Toracica, Fondazione Policlinico Universitario Agostino Gemelli, IRCCS, 00168, Roma
Galluccio	Giovanni	Center of Thoracic Endoscopy and Interventional Pulmonology, Regina Apostolorum Hospital, Albano Laziale, Italy
Gangemi	Mariapia	UOC Chirurgia toracica, Azienda ospedaliero universitario Policlinico-San Marco, Catania, Italy
Gazzaniga	Paola	Dipartimento di Medicina Molecolare, Dipartimento di Chirurgia Generale e Specialistica, Sapienza Università di Rom
Gentile	Carlo	Pathology Unit, "Renato Dulbecco", University Hospital, Catanzaro, Italy
Genualdo	Marcella	Thoracic Surgery Unit, Department of Precision and Regenerative Medicine and Ionian Area, University of Bari “Aldo Moro”, Bari, Italy
Gherardi	Marco	Pulmonary Unit, Cardiothoracic and Vascular Department, Pisa University Hospital, Pisa, Italy
Ghiribelli	Claudia	UOSA Diagnostic and interventional Bronchoscopy, Siena
Giacomo	Cusumano	UOC Chirurgia toracica, Azienda ospedaliero universitario Policlinico-San Marco, Catania, Italy
Gianfreda	Davide	Unità Operativa Complessa di Nefrologia, Ospedale Vito Fazzi, Lecce
Giannone	Silvia	Department of Public Health and Infectious Diseases, Sapienza University of Rome, Italy
Gioffrè	Giuseppina	Division of Pulmonary Medicine, Sandro Pertini Hospital—Rome (Italy)
Giorgetta	Casimiro Eugenio	Division of Thoracic Surgery, ASST Valtellina e Alto Lario, “Eugenio Morelli” Hospital, 23035 Sondalo, Italy
Giorgiana	Noemi	UOC Chirurgia Toracica Università degli studi della Campania ‘Luigi Vanvitelli’, Napoli
Giovanardi	Michele	ASST Carlo Poma Mantova, Mantova
Giovanniello	Delia	San Camillo Forlanini Hospital, Rome, Italy; University of Rome, Rome, Italy
Girelli	Lara	Department of Thoracic Surgery, IEO, European Institute of Oncology IRCCS, Milan, Italy
Giudice	Gabriella	Department of Thoracic Surgery, IRCCS-CROB Centro di Riferimento Oncologico della Basilicata, Rionero in Vulture, PZ, Italy
Giunchi	Francesca	Pathology Unit, IRCCS Azienda Ospedaliero-Universitaria di Bologna, Department of Medical and Surgical Science (DIMEC), Italy
Giuntoli	Leonardo	Pneumologia Interventistica, AOU Careggi, Firenze
Godono	Alessandro	Occupational Medicine Division, Laboratory of Toxicology and Industrial Epidemiology, Department of Public Health and Paediatrics, University of Turin, Turin, Italy
Graziano	Paolo	Department of Radiology, Oncology and Pathology, Sapienza University of Rome, Italy
Grisorio	Giacomo	Department of Thoracic Surgery, ASST Grande Ospedale Metropolitano Niguarda, Milano, Italy
Grispi	Chiara	Thoracic Surgery Division, Department of Oncology, San Luigi Hospital, University of Turin, Orbassano, Italy
Grossi	William	Department of Thoracic Surgery, Ospedale Santa Maria della Misericordia, Udine, Italy

Gualano	Gina	UOC Malattie infettive dell'apparato respiratorio, IRCCS INMI Spallanzani-Rome (Italy)
Guerrera	Francesco	Thoracic Surgery Unit, Università di Torino, Turin, Italy
Guerrini	Susanna	Diagnostic Imaging Unit, Department of Medical Sciences, University of Siena, Azienda Ospedaliero-Universitaria Senese, Siena, Italy
Guggino	Gianluca	U.O.C. di Chirurgia Toracica, A.O.R.N. "A.Cardarelli" Napoli, Italia
Guglielmi	Giacomo	Department of Surgical, Medical, and Molecular Pathology and Critical Care Medicine, University of Pisa, Pisa, Italy
Guglielmi	Giovanni	Preventive and Occupational Medicine Unit, Pisa University Hospital, Pisa, Italy
Guidobaldi	Leo	Division of Anatomic Pathology, Sandro Pertini Hospital—Rome
Huet	Olivier	Department of Anaesthesiology and Intensive Care Medicine, Brest University Hospital, Brest, France
Ielo	Giuseppe	University of Catania, AOU Policlinico G. Rodolico—San Marco, Italy
Imbriglio	Giovanna	Thoracic Surgery Unit, Vito Fazzi Hospital, Lecce
Infusino	Stefania	Division of Oncology, Onco-Hematology Departement, Hospital of Cosenza "SS Annunziata", Cosenza, Italy
Inzirillo	Francesco	Division of Thoracic Surgery, ASST Valtellina e Alto Lario, "Eugenio Morelli" Hospital, 23035 Sondalo, Italy
Iovine	Paola Rebecca	University of Napoli Federico II, AOU Monaldi, Italy
Ippolito	Gianluca	Department of Health Sciences, University "Magna Græcia" of Catanzaro, Catanzaro, Italy
Jaus	Massimo Osvaldo	San Camillo Forlanini Hospital, Rome, Italy
Jougon	Jacques	Thoracic Surgery Division, Bordeaux University Hospital, France
Juvin	Charles	Cardiovascular Surgery, Assistance Publique Hôpitaux de Paris, Paris, France
Kumar	Karan	Thoracic Surgery Unit, Tor Vergata University Policlynic, Rome, Italy
Kuzmych	Khrystyna	Department of Thoracic Surgery, Fondazione Policlinico Universitario A.Gemelli IRCCS, Università Cattolica del Sacro Cuore, Rome, Italy
La Manna	Carmine	Thoracic Surgery Unit, Istituto Nazionale Tumori IRCCS Fondazione G. Pascale, Naples, Italy
La Mattina	Giuseppe	A.O.O.R. Villa Sofia—Cervello—Palermo (Italy)
La Rocca	Antonello	Thoracic Surgery Unit, Istituto Nazionale Tumori IRCCS Fondazione G. Pascale, Naples, Italy
La Rocca	Eleonora	U.O.C. Chirurgia Toracica, IRCCS Ospedale Policlinico San Martino, Genova
Lamesta	Antonio	University of Modena and Reggio Emilia
Landini	Nicholas	Department of Radiology, Oncology and Pathology, Sapienza University of Rome, Italy
Lanfranchi	Filippo	Respiratory Disease Unit, Department of Cardiac Thoracic and Vascular Scienses, Ospedale dell'Angelo, Venice, Italy.
Lastaria	Francesco	Università di Foggia, Azienda ospedaliero-universitaria "Policlinico Riuniti" Foggia, Struttura Complessa di Chirurgia Toracica Universitaria, Foggia-Italy
Lavecchia	Annamaria	Pathology Unit, "Renato Dulbecco", University Hospital, Catanzaro, Italy
Lebreton	Guillaume	Cardiovascular Surgery, Assistance Publique Hôpitaux de Paris, Paris, France
Leo	Francesco	Thoracic Surgery Division, Oncology Department, S. Luigi Hospital, University of Torino, Italy; Smoking Cessation Unit, Périgueux General Hospital, France
Leonardi	Beatrice	UOC Chirurgia Toracica Università degli studi della Campania 'Luigi Vanvitelli', Napoli
Leoncini	Giacomo	U.O.C. Chirurgia Toracica, IRCCS Ospedale Policlinico San Martino, Genova
Leone	Francesco	UOC Chirurgia Toracica Università degli studi della Campania 'Luigi Vanvitelli', Napoli
Leone	Armando	U.O.C. Pneumologia, Ospedale San Giuseppe Moscati, Taranto, Italia
Leoni	Claudia	Dipartimento di Chirurgia Toracica, Fondazione Policlinico Universitario Agostino Gemelli, IRCCS, 00168, Roma
Lequaglie	Cosimo	Department of Thoracic Surgery, IRCCS-CROB Centro di Riferimento Oncologico della Basilicata, Rionero in Vulture, PZ, Italy
Libretti	Lidia	Department of Thoracic Surgery, Fondazione IRCCS San Gerardo dei Tintori, 20900 Monza, Italy
Licata	Maria Angela Vittoria Anna Chiara	UOC Malattie infettive dell'apparato respiratorio, IRCCS INMI Spallanzani-Rome (Italy)
Ligabue	Tommaso	Thoracic Surgery Unit, Department of Medical, Surgical and Neuro Sciences, University of Siena, Azienda Ospedaliero-Universitaria Senese, Siena, Italy
Lione	Luigi	Padova University Hospital—Thoracic Surgery Unit, Padova, Italy
Lo Iacono	Giorgio	Department of Thoracic Surgery, IEO, European Institute of Oncology IRCCS, Milan, Italy
Lo Torto	Sara	Department of Thoracic Surgery, Tor Vergata University Polyclinic, Rome, Italy
Lococo	Filippo	Department of Thoracic Surgery, Fondazione Policlinico Universitario A.Gemelli IRCCS, Università Cattolica del Sacro Cuore, Rome, Italy
Locorotondo	Cristian	Institute of Respiratory Disease, University of Medicine "Aldo Moro"—Bari, Italy
Loi	Mauro	Radioterapia, AOU Careggi, Firenze
Loizzi	Domenico	Università di Foggia, Azienda ospedaliero-universitaria "Policlinico Riuniti" Foggia, Struttura Complessa di Chirurgia Toracica Universitaria, Foggia
Londero	Francesco	Department of Thoracic Surgery, Ospedale Santa Maria della Misericordia, Udine, Italy
Longhini	Federico	Università degli Studi di Catanzaro "Magna Graecia"
Longo	Pierfrancesco	UOC Pneumologia, Ospedale Cardinale G. Panico Tricase (Italy)
Longo	Filippo	Department of Thoracic Surgery, Fondazione Policlinico Universitario Campus Bio-Medico, Rome
Longobardi	Salvatore	Department of Pneumology—Grande Ospedale Metropolitano Niguarda—Milan (Italy)
Lopez	Camillo	U.O.C. Chirurgia Toracica, Ospedale "Vito Fazzi", Lecce

Lovadina	Stefano	Thoracic Surgery Unit, Cattinara University Hospital, Trieste, Italy
Luca	Pogliani	Department of Thoracic Surgery, ASST Grande Ospedale Metropolitano Niguarda, Milano, Italy
Lucchi	Marco	Thoracic Surgery Unit, Università di Pisa, Pisa, Italy
Lulaj	Ernesto	U.O.C. Malattie apparato respiratorio, Policlinico di Bari, Bari, Italia
Luongo	Luca	Unità Operativa Complessa di Medicina Nucleare, Ospedale Vito Fazzi, Lecce
Luzzi	Valentina	Pneumologia Interventistica, AOU Careggi, Firenze
Luzzi	Luca	Lung Transplant Unit, Department of Medical, Surgical and Neuro Sciences, Azienda Ospedaliero-Universitaria Senese, University of Siena, 53100 Siena, Italy
Lyberis	Paraskevas	A.O.U. Città della salute e della Scienza di Torino
Maiolino	Elena	Thoracic and Vascular Surgery, Assistance Publique—Hôpitaux de Paris, Bobigny, France
Malizia	Jessica	Thoracic Surgery, University of Perugia; Perugia
Mammana	Marco	Padova University Hospital—Thoracic Surgery Unit, Padova, Italy
Mancino	Laura	Respiratory Disease Unit, Department of Cardiac Thoracic and Vascular Scienses, Ospedale dell'Angelo, Venice, Italy.
Manfredini	Beatrice	Minimally Invasive and Robotic Thoracic Surgery—Surgical, Medical, Molecular and Critical Care Pathology Department, University Hospital of Pisa
Mangiafico	Santi	Gastroenterology and Digestive Endoscopy, University Hospital-Policlinico-San Marco, Catania, Italy
Mangiapan	Gilles	Service de Pneumologie CHI de Créteil, Créteil, France
Maniscalco	Pio	Arcispedale Sant'Anna, Ferrara, Italy
Manitto	Mattia	U.O.C. Chirurgia Toracica, IRCCS Ospedale Policlinico San Martino, Genova
Mannino	Maurizio	Thoracic Surgery Unit, University Hospital-Policlinico-San Marco, Catania, Italy
Mantovani	Sara	San Camillo Forlanini Hospital, Rome, Italy
Marasco	Rita Daniela	Università di Foggia, Azienda ospedaliero-universitaria "Policlinico Riuniti" Foggia, Struttura Complessa di Chirurgia Toracica Universitaria, Foggia-Italy
Marcaccini	Marco	Thoracic Surgery Division, Department of Oncology, San Luigi Hospital, University of Turin, Orbassano, Italy
Marchesani	Francesca	UOC Pneumologia AST Macerata, Ospedale Generale di Macerata (Italy)
Marchese	Andrea	Division of Plastic Surgery, Fondazione IRCCS San Gerardo dei Tintori, Monza, Italy
Marchi	Guido	Pulmonary Unit, Cardiothoracic and Vascular Department, Pisa University Hospital, Pisa, Italy
Margaritora	Stefano	Department of Thoracic Surgery, Fondazione Policlinico Universitario A.Gemelli IRCCS, Università Cattolica del Sacro Cuore, Rome, Italy
Marinari	Stefano	UOC Pneumologia Teramo (Italy)
Marrazzo	Giuseppina	Pulmonology Unit, "Renato Dulbecco", University Hospital, Catanzaro, Italy
Martella	Vilma	Unità Operativa Complessa di Nefrologia, Ospedale Vito Fazzi, Lecce
Martinod	Emmanuel	AP-HP, Hôpitaux Universitaires Paris Seine-Saint-Denis, Hôpital Avicenne, Chirurgie Thoracique et Vasculaire, Université Sorbonne Paris Nord, Faculté de Médecine SMBH, Bobigny, France
Martucci	Nicola	Thoracic Surgery Unit, Istituto Nazionale Tumori IRCCS Fondazione G. Pascale, Naples, Italy
Marvulli	Maria	UOC Chirurgia Toracica Università degli studi della Campania 'Luigi Vanvitelli', Napoli
Masaro	Simonetta	Thoracic Surgery Unit, Cattinara University Hospital, Trieste, Italy
Maselli	Leonardo	Institute of Respiratory Disease, University of Medicine "Aldo Moro"—Bari, Italy
Masi	Umberto	Department of Translational Medicine, University of Campania "L. Vanvitelli", Naples, Italy
Mastrobattista	Annelisa	UOC Malattie infettive dell'apparato respiratorio, IRCCS INMI Spallanzani-Rome (Italy)
Masullo	Gianluca	Department of Thoracic Surgery, Ospedale Santa Maria della Misericordia, Udine, Italy
Mathieu	Federico	Lung Transplant Unit, Department of Medical, Surgical and Neuro Sciences, Azienda Ospedaliero-Universitaria Senese, University of Siena, 53100 Siena, Italy
Matino	Silvia	Unità Operativa Complessa di Nefrologia, Ospedale Vito Fazzi, Lecce
Mattioli	Gian Paolo	UOC Pneumologia AST Macerata, Ospedale Generale di Macerata (Italy)
Mattioni	Giovanni	Department of Thoracic Surgery, University of Milan, Milan, Italy
Mattoccia	Francesco M.	UOC Chirurgia Toracica e Trapianti di Polmone, Dipartimento di Chirurgia Generale e Specialistica, Sapienza Università di Roma
Maurizi	Giulio	Thoracic Surgery Sant'Andrea Hospital, La Sapienza University, Rome, Italy
Mazzarra	Sara	Department of Diagnostic and Specialty Medicine, University of Bologna, Thoracic Surgery Unit, Morgagni-Pierantoni Hospital, Forli, Italy
Mazzei	Maria Antonietta	Diagnostic Imaging Unit, Department of Medical, Surgical and Neuro Sciences and of Medical Sciences, University of Siena, Azienda Ospedaliero-Universitaria Senese, Siena, Italy
Mazzella	Antonio	Department of Thoracic Surgery, IEO, European Institute of Oncology IRCCS, Milan, Italy
Mc Bride	Tarun	Thoracic Surgery Division, Périgueux General Hospital, France
Meacci	Elisa	Department of Thoracic Surgery, Fondazione Policlinico Universitario A.Gemelli IRCCS, Università Cattolica del Sacro Cuore, Rome, Italy
Melan	Luca	Padova University Hospital—Thoracic Surgery Unit, Padova, Italy
Melfi	Franca	Minimally Invasive and Robotic Thoracic Surgery—Surgical, Medical, Molecular and Critical Care Pathology Department, University Hospital of Pisa
Melis	Alberto	Thoracic Surgery, University of Perugia; Perugia
Mellone	Federica	U.O.C. Chirurgia Toracica, IRCCS Ospedale Policlinico San Martino, Genova
Mencarini	Paola	UOC Malattie infettive dell'apparato respiratorio, IRCCS INMI Spallanzani-Rome (Italy)

Mencarini	Jessica	Malattie infettive e Tropicali, AOU Careggi, Firenze
Menichini	Ilaria	Center of Thoracic Endoscopy and Interventional Pulmonology, Regina Apostolorum Hospital, Albano Laziale, Italy
Menna	Cecilia	Thoracic Surgery Sant'Andrea Hospital, La Sapienza University, Rome, Italy
Mercadante	Edoardo	Thoracic Surgery Unit, Istituto Nazionale Tumori IRCCS Fondazione G. Pascale, Naples, Italy
Messa	Fabiana	Thoracic Surgery Sant'Andrea Hospital, La Sapienza University, Rome, Italy
Messina	Gaetana	UOC Chirurgia Toracica Università degli studi della Campania 'Luigi Vanvitelli', Napoli
Messina	Salvatore	Nuclear Medicine, Perugia
Messina	Maddalena	Respiratory Diseases Unit, Department of Medicine, Surgery and Neurosciences, University of Siena, Siena 53100, Italy
Michieletto	Lucio	Respiratory Disease Unit, Department of Cardiac Thoracic and Vascular Scienses, Ospedale dell'Angelo, Venice, Italy.
Migliano	Francesco	Thoracic Surgery, University Hospital Maggiore della Carita, Novara, Italy
Migliaretti	Giuseppe	Department of Public Health and Paediatric Sciences, University of Torino, Italy
Minonne	Chiara	Department of Medicine, Surgery and Pharmacy, University of Sassari, Sassari, Italy
Mirabelli	Flavio Marco	Department of Public Health and Infectious Diseases, Sapienza University of Rome, Italy. Pulmonology, Policlinico Umberto I Hospital Rome, Italy
Misceo	Francesca	Division of thoracic surgery, IRCCS San Raffaele Scientific Institute, Milan, Italy; Università Vita-Salute San Raffaele, Milan, Italy
Mohsen	Ibrahim	Thoracic Surgery Sant'Andrea Hospital, La Sapienza University, Rome, Italy
Mondonni	Michele	Respiratory Unit, ASST Santi Paolo e Carlo, San Paolo Hospital, Department of Health Sciences, University of Milan, Milan, Italy
Mongiello	Diletta	Thoracic Surgery Unit, Department of Precision and Regenerative Medicine and Ionian Area, University of Bari Aldo Moro, Bari, Italy; Thoracic Surgery Unit, Policlinico Universitario Riuniti di Foggia, Foggia, Italy
Montenegro	Nicola	Department of Health Sciences, University "Magna Græcia" of Catanzaro, Catanzaro, Italy
Monteverde	Marco	ASST Carlo Poma Mantova, Mantova
Morand	Lamya Chrif	Private Office, Kènitra, Morocco
Morelli	Lucia	U.O.C. Chirurgia Toracica, IRCCS Ospedale Policlinico San Martino, Genova
Morriello	Michele	UOC Pneumologia Teramo (Italy)
Mosti	Silvia	UOC Malattie infettive dell'apparato respiratorio, IRCCS INMI Spallanzani-Rome (Italy)
Motta	Giovanna	Unità operativa di Ematologia 1, Azienda ospedaliero universitario Policlinico-San Marco, Catania, Italy
Mucaj	Klodjana	Department of Internal Medicine and Medical Therapeutics, University of Pavia, Italy, Cardiothoracic and Vascular Department, Unit of Respiratory Diseases, IRCCS Policlinico San matteo, Pavia, Italy
Muriana	Piergiorgio	Department of Thoracic Surgery, IRCCS Scientific Institute San Raffaele, Milan
Nachira	Dania	Department of Thoracic Surgery, Fondazione Policlinico Universitario A.Gemelli IRCCS, Università Cattolica del Sacro Cuore, Rome, Italy
Naldi	Giuseppe	Division of Thoracic Surgery, ASST Valtellina e Alto Lario, "Eugenio Morelli" Hospital, 23035 Sondalo, Italy
Nania	Fabio Alfredo	Anesthesia and intensive care unit, "Santa Maria Goretti" Hospital, Latina, Italy
Nannini	Nazarena	Alma Mater Studiorum, Department of Medical and Surgical Sciences (DIMEC), University of Bologna, italy
Napoli	Marcello	Unità Operativa Complessa di Nefrologia, Ospedale Vito Fazzi, Lecce
Napolitano	Antonio Giulio	Department of Thoracic Surgery, Fondazione Policlinico Universitario A.Gemelli IRCCS, Università Cattolica del Sacro Cuore, Rome, Italy
Nardoni	Stefano	Pathology Unit, S. Filippo Neri Hospital, ASLROMA1, Rome
Natale	Giovanni	UOC Chirurgia Toracica Università degli studi della Campania 'Luigi Vanvitelli', Napoli
Natali	Gian Luca	Departement of Surgical Sciences, University of Rome Tor Vergata, Via Montpellier 1, 00133, Rome, Itay
Natali	Filippo	Interventional Pulmonology Unit, IRCCS Azienda Ospedaliero-Universitaria di Bologna, Bologna Italy
Nava	Fabrizio	Department of Pneumology—Grande Ospedale Metropolitano Niguarda—Milan (Italy)
Nerli	Gianluca	Department of Translational Research and New technologies in Medicine and Surgery, University of Pisa, Pisa, Italy
Nicosia	Samanta	A.O.U. Città della salute e della Scienza di Torino
Nicotra	Samuele	Padova University Hospital—Thoracic Surgery Unit, Padova, Italy
Nocera	Adriana	Department of Thoracic Surgery, Fondazione Policlinico Universitario A.Gemelli IRCCS, Università Cattolica del Sacro Cuore, Rome, Italy
Noro	Antonio	U.O.C. di Chirurgia Toracica, A.O.R.N. "A.Cardarelli" Napoli, Italia
Notizia	Luca	U.O.C. Pneumologia—"San Giovanni di Dio e Ruggi D'Aragona" University Hospital, Salerno, Italy
Novellis	Pierluigi	Division of thoracic surgery, IRCCS San Raffaele Scientific Institute, Milan, Italy
Novello	Luca	U.O.C. Chirurgia Toracica, IRCCS Ospedale Policlinico San Martino, Genova
Olmati	Federica	Department of Public Health and Infectious Diseases, Sapienza University. Division of Pulmonary Medicine, Policlinico Umberto I Hospital—Rome
Onofri	Andrea	Department of Agricultural, Food and Environmental Sciences, University of Perugia, Perugia, Italy
Onorati	Ilaria	AP-HP, Hôpitaux Universitaires Paris Seine-Saint-Denis, Hôpital Avicenne, Chirurgie Thoracique et Vasculaire, Université Sorbonne Paris Nord, Faculté de Médecine SMBH, Bobigny, France
Opromolla	Giorgia	Thoracic Surgery Unit, Istituto Nazionale Tumori IRCCS Fondazione G. Pascale, Naples, Italy
Orlandi	Riccardo	Department of Thoracic Surgery, University of Milan, Milan, Italy
Pacella	Giulia	Università di Foggia, Azienda ospedaliero-universitaria "Policlinico Riuniti" Foggia, Struttura Complessa di Chirurgia Toracica Universitaria, Foggia-Italy

Pagliarini	Giulia	Padova University Hospital—Thoracic Surgery Unit, Padova, Italy
Paladini	Luigi	Department of Medical and Surgical Sciences, Institute of Respiratory Disease, University Hospital, Foggia, Italy; Respiratory Diseases Unit Antonio Blasi, Presidio Ospedaliero A. Perrino, Brindisi, Italy
Paladini	Piero	Lung Transplant Unit, Department of Medical, Surgical and Neuro Sciences, Azienda Ospedaliero-Universitaria Senese, University of Siena, 53100 Siena, Italy
Palange	Paolo	Department of Public Health and Infectious Diseases, Sapienza University of Rome, Italy. Pulmonology, Policlinico Umberto I Hospital Rome, Italy
Palmieri	Fabrizio	UOC Malattie infettive dell’apparato respiratorio, IRCCS INMI Spallanzani-Rome (Italy)
Palmiotti	Giuseppe Antonio	U.O.C. Pneumologia, Ospedale San Giuseppe Moscati, Taranto, Italia
Paracchini	Elena	Respiratory Diseases Unit, Medical Dpartment, AOU Maggiore della Carità di Novara
Parini	Sara	Thoracic Surgery, University Hospital Maggiore della Carita, Novara, Italy
Pariscenti	Gian Luca	U.O.C. Chirurgia Toracica, IRCCS Ospedale Policlinico San Martino, Genova
Pasqualotto	Federico	Department of Public Health and Infectious Diseases, Sapienza University of Rome, Italy
Passone	Erika	Thoracic Surgery, University Hospital Maggiore della Carita, Novara, Italy
Patirelis	Alexander	Departement of Surgical Sciences, University of Rome Tor Vergata, Via Montpellier 1, 00133, Rome, Itay
Patirelis	Alexandro	Thoracic Surgery Unit, Tor Vergata University Policlynic, Viale Oxford 81, 00133 Rome, Italy
Patrucco	Filippo	Respiratory Diseases Unit, Medical Dpartment, AOU Maggiore della Carità di Novara
Pelaia	Corrado	Pulmonology Unit, "Renato Dulbecco", University Hospital, Catanzaro, Italy, Department of Medical and Surgical Sciences University "Magna Graecia" of Catanzaro
Pelaia	Girolamo	Pulmonology Unit, "Renato Dulbecco", University Hospital, Catanzaro, Italy , Department of Health Sciences, University “Magna Græcia” of Catanzaro, Catanzaro, Italy
Peretti	Marine	AP-HP, Hôpitaux Universitaires Paris Seine-Saint-Denis, Hôpital Avicenne, Chirurgie Thoracique et Vasculaire, Université Sorbonne Paris Nord, Faculté de Médecine SMBH, Bobigny, France
Peris	Ketty	Dermatology Unit, Fondazione Policlinico Universitario A. Gemelli IRCCS, Università Cattolica del Sacro Cuore, Rome, Italy
Pernazza	Angelina	Department of Radiology, Oncology and Pathology, Sapienza University of Rome, Italy
Pesci	Alessandra	UOC Medicina Interna ad alta intensità, Ospedale Santa Maria Nuova, Firenze (Italy)
Petracca-Ciavarella	Leonardo	Department of Thoracic Surgery, Fondazione Policlinico Universitario A.Gemelli IRCCS, Università Cattolica del Sacro Cuore, Rome, Italy
Petrella	Francesco	Division of Thoracic Surgery, Fondazione IRCCS San Gerardo dei Tintori, Monza, Italy
Petroni	Lorenzo	Università degli Studi di Firenze, Scuola di Specializzazione Malattie dell’Apparato Respiratorio, Firenze Italy
Piamonti	Daniel	Department of Public Health and Infectious Diseases, Sapienza University of Rome, Italy
Pica	Davide	UOC Chirurgia Toracica Università degli Studi della Campania ‘Luigi Vanvitelli’ Napoli
Piccioni	Giorgia	Thoracic Surgery Sant’Andrea Hospital, La Sapienza University, Rome, Italy
Piccirillo	Silvia	U.O.C. Pneumologia—”San Giovanni di Dio e Ruggi D’Aragona” University Hospital, Salerno, Italy
Pierandrei	Chiara	UOC Pneumologia AST Macerata, Ospedale Generale di Macerata (Italy)
Piras	Maria Teresa	U.O.C. Chirurgia Toracica, IRCCS Ospedale Policlinico San Martino, Genova
Pirina	Pietro	Department of Medicine, Surgery and Pharmacy, University of Sassari, Sassari, Italy, Clinical and Interventional Pneumology, University Hospital of Sassari, Sassari, Italy
Piro	Roberto	Pulmonology Unit, Azienda Unità Sanitaria Locale-IRCCS di Reggio Emilia—Reggio Emilia (RE)
Pirondini	Emanuele	Division of Thoracic Surgery, Fondazione IRCCS San Gerardo dei Tintori, Monza, Italy
Pistelli	Francesco	Department of Surgical, Medical, and Molecular Pathology and Critical Care Medicine, University of Pisa, italy
Pizzuto	Ondina	Thoracic Surgery Unit, Department of Precision and Regenerative Medicine and Ionian Area, University of Bari “Aldo Moro”, Bari, Italy
Poggi	Camilla	UOC Chirurgia Toracica e Trapianti di Polmone, Dipartimento di Chirurgia Generale e Specialistica, Sapienza Università di Roma
Polimeno	Emilia	U.O.C. di Chirurgia Toracica, A.O.R.N. “A.Cardarelli” Napoli, Italia
Pollorsi	Chiara	Pulmonology Unit, Azienda Unità Sanitaria Locale-IRCCS di Reggio Emilia—Reggio Emilia (RE)
Pontillo	Domenico	Department of Anesthesia and Intensive Care, IRCCS Scientific Institute San Raffaele, Milan
Porciatti	Francesco	Department of Translational Research and New technologies in Medicine and Surgery, University of Pisa, Pisa, Italy
Pordon	Elena	UOC Respiratory diseases, Medical, Surgical and Neurochirigical Sciences—University of Siena
Portela	Ana Maria	AP-HP, Hôpitaux Universitaires Paris Seine-Saint-Denis, Hôpital Avicenne, Chirurgie Thoracique et Vasculaire, Université Sorbonne Paris Nord, Faculté de Médecine SMBH, Bobigny, France
Potenza	Ilaria	Arcispedale Sant’Anna, Ferrara, Italy
Pourmolkara	Domenico	Department of Thoracic Surgery, University of Perugia Medical School, Perugia, Italy
Puca	Maria Antonietta	UOC Chirurgia Toracica Università degli Studi della Campania ‘Luigi Vanvitelli’ Napoli
Puma	Francesco	Department of Thoracic Surgery, University of Perugia Medical School, Perugia, Italy
Puzhaliakov	Valeriy	Division of Thoracic Surgery, Cardio-Thoracic-Vascular Departement, Hospital of Cosenza "SS Annunziata", Cosenza, Italy
Quarantotto	Francesco	Arcispedale Sant’Anna, Ferrara, Italy
Quercia	Rosatea	Thoracic Surgery Unit, Department of Precision and Regenerative Medicine and Ionian Area, University of Bari “Aldo Moro”, Bari, Italy

Radu	Dana	AP-HP, Hôpitaux Universitaires Paris Seine-Saint-Denis, Hôpital Avicenne, Chirurgie Thoracique et Vasculaire, Université Sorbonne Paris Nord, Faculté de Médecine SMBH, Bobigny, France
Randazzo	Raul	Thoracic Surgery Unit, Tor Vergata University Policlynic, Viale Oxford 81, 00133 Rome, Italy
Rapanà	Roberta	Thoracic Surgery Unit, Vito Fazzi Hospital, Lecce
Ravalli	Eugenio	Division of Thoracic Surgery, ASST Valtellina e Alto Lario, “Eugenio Morelli” Hospital, 23035 Sondalo, Italy
Ravasin	Alice	Thoracic Surgery Unit, Careggi University Hospital, Florence, Italy
Raveglia	Federico	Division of Thoracic Surgery, Fondazione IRCCS San Gerardo dei Tintori, Monza, Italy
Rea	Federico	Padova University Hospital—Thoracic Surgery Unit, Padova, Italy
Rebusso	Alessandro	Padova University Hospital—Thoracic Surgery Unit, Padova, Italy
Reda	Marco	Department of Thoracic Surgery, ASST Grande Ospedale Metropolitano Niguarda, Milano, Italy
Rena	Ottavio	Thoracic Surgery, University Hospital Maggiore della Carita, Novara, Italy
Rendina	Erino Angelo	Thoracic Surgery Sant’Andrea Hospital, La Sapienza University, Rome, Italy
Repaci	Emma	Department of Public Health and Infectious Diseases, Sapienza University of Rome, Italy. Pulmonology, Policlinico Umberto I Hospital Rome, Italy
Ria	Paolo	Unità Operativa Complessa di Nefrologia, Ospedale Vito Fazzi, Lecce
Ribechini	Alessandro	Service of Thoracic Endoscopy, Cardiothoracic and Vascular Department, University of Pisa, Pisa, Italy
Ricciardi	Sara	San Camillo Forlanini Hospital, Rome, Italy; University of Bologna, Bologna, Italy
Riccio	Carmine	Department of Pneumology—Grande Ospedale Metropolitano Niguarda—Milan (Italy)
Riefolo	Mattia	Pathology Unit, IRCCS Azienda Ospedaliero-Universitaria di Bologna, Department of Medical and Surgical Science (DIMEC), Italy
Righi	Luisella	Patology Unit, Department of Oncology, San Luigi Hospital, University of Turin, Orbassano, Italy
Romano	Gaetano	Minimally Invasive and Robotic Thoracic Surgery—Surgical, Medical, Molecular and Critical Care Pathology Department, University Hospital of Pisa
Romano	Rosalia	Division of Thoracic Surgery, IRCCS Sacro Cuore-Don Calabria Hospital, Verona, Italy
Romito	Mara	Thoracic Surgery, University of Perugia
Rossetti	Francesca	Department of Thoracic Surgery, IRCCS Scientific Institute San Raffaele, Milan
Rossi	Federica	Occupational Medicine Division, Laboratory of Toxicology and Industrial Epidemiology, Department of Public Health and Paediatrics, University of Turin, Turin, Italy
Rossi	Ernesto	Medical Oncology Unit, Fondazione Policlinico Universitario A. Gemelli IRCCS,Università Cattolica del Sacro Cuore, Rome, Italy
Ruffini	Enrico	A.O.U. Città della salute e della Scienza di Torino
Ruggiero	Patrizia	Pulmonology Unit, Azienda Unità Sanitaria Locale-IRCCS di Reggio Emilia—Reggio Emilia (RE)
Ruggiero	Ciro	Thoracic Surgery Unit, Baggiovara Hospital of Modena, Modena, Italy
Russo	Alessandra	Thoracic Surgery Division, Department of Oncology, San Luigi Hospital, University of Turin, Orbassano, Italy
Russo	Matteo	Department of Industrial Engineering, University of Rome Tor Vergata, Rome, Italy
Russo	Emanuele	U.O.C. di Chirurgia Toracica, A.O.R.N. “A.Cardarelli” Napoli, Italia
Saggio	Damiano	Department of Thoracic Surgery, University of Rome Tor Vergata, Rome, Italy
Saidi	Imane	UM6P Hospital, Marrakech, Morocco
Salerni	Carmine	Respiratory Unit, ASST Santi Paolo e Carlo, San Paolo Hospital, Department of Health Sciences, University of Milan, Milan, Italy
Salih	Dian	Infectious Diseases Unit, Department of Clinical and Surgical Sciences, University of Foggia, Foggia-Italy
Salvadori	Lorenzo	San Camillo Forlanini Hospital, Rome, Italy
Sambataro	Viola	Padova University Hospital—Thoracic Surgery Unit, Padova, Italy
Sampietro	Doroty	Thoracic Surgery Unit, Department of Precision and Regenerative Medicine and Ionian Area, University of Bari Aldo Moro, Bari, Italy
Sanna	Arianna	Department of Public Health and Infectious Diseases, Sapienza University of Rome, Italy
Santantonio	Teresa	Infectious Diseases Unit, Department of Clinical and Surgical Sciences, University of Foggia, Foggia-Italy
Santello	Virginia	Respiratory Disease Unit, Department of Cardiac Thoracic and Vascular Sciences, Ospedale dell'Angelo, Venice Italy
Santhirakumaran	Gowthanan	Barts Thorax Centre, St Bartholomew’s Hospital, Barts Health NHS Foundation Trust, London, UK
Saracino	Laura	Cardiothoracic and Vascular Department, Unit of Respiratory Diseases, IRCCS Policlinico San Matteo, Pavia, Italy
Sarubbi	Antonio	Department of Thoracic Surgery, Fondazione Policlinico Universitario Campus Bio-Medico, Rome
Sassorossi	Carolina	Department of Thoracic Surgery, Fondazione Policlinico Universitario A.Gemelli IRCCS, Università Cattolica del Sacro Cuore, Rome, Italy
Savarelli	Naomi	Thoracic Surgery Unit, Department of Precision and Regenerative Medicine and Ionian Area, University of Bari “Aldo Moro”, Bari, Italy
Scala	Cinzia	Division of Thoracic Surgery, IRCCS San Raffaele Scientific Institute, Milan, Italy; Università Vita-Salute San Raffaele, Milan, Italy
Scanagatta	Paolo	Division of Thoracic Surgery, ASST Valtellina e Alto Lario, “Eugenio Morelli” Hospital, 23035 Sondalo, Italy
Scarascia	Daniele	Division of Thoracic Surgery, Cardio-Thoracic-Vascular Departement, Hospital of Cosenza "SS Annunziata", Cosenza, Italy
Scarlata	Simone	Campus Bio-Medico University Hospital Foundation of Rome, Santa Scolastica Hospital of Cassino
Schiavon	Marco	Padova University Hospital—Thoracic Surgery Unit, Padova, Italy

Schinzari	Giovanni	Medical Oncology Unit, Fondazione Policlinico Universitario A. Gemelli IRCCS,Università Cattolica del Sacro Cuore, Rome, Italy
Schirò	Miriam	U.O.C. Pneumologia—"San Giovanni di Dio e Ruggi D'Aragona" University Hospital, Salerno, Italy
Scognamiglio	Chiara	Dipartimento di Chirurgia Toracica, Fondazione Policlinico Universitario Agostino Gemelli, IRCCS, 00168, Roma
Sebastianelli	Valerio	UOC Chirurgia Toracica e Trapianti di Polmone, Dipartimento di Chirurgia Generale e Specialistica, Sapienza Università di Roma
Senatore	Alessia	Department of Thoracic Surgery, Fondazione Policlinico Universitario A.Gemelli IRCCS, Università Cattolica del Sacro Cuore, Rome, Italy
Serafini	Maria	UOC Pneumologia AST Macerata, Ospedale Generale di Macerata (Italy)
Sestini	Stefano	ASST Carlo Poma Mantova, Mantova
Sibilia	Maria Chiara	University of Milan, Milan, Italy; Department of Thoracic Surgery, Fondazione IRCCS San Gerardo dei Tintori, 20900 Monza, Italy
Siciliani	Alessandra	Thoracic Surgery Sant'Andrea Hospital, La Sapienza University, Rome, Italy
Sicolo	Elisa	Minimally Invasive and Robotic Thoracic Surgery—Surgical, Medical, Molecular and Critical Care Pathology Department, University Hospital of Pisa
Signore	Francesca	Thoracic Surgery Unit, Vito Fazzi Hospital, Lecce
Sigona	Marco	Thoracic Surgery Unit, University Hospital-Policlinico-San Marco, Catania, Italy
Sirbu	Andra Stefana	Department of Thoracic Surgery, University of Rome Tor Vergata, Rome, Italy
Sobrero	Simona	Thoracic Surgery Division, Department of Oncology, San Luigi Hospital, University of Turin, Orbassano, Italy
Solli	Piergiorgio	Division of Thoracic Surgery, IRCCS Azienda Ospedaliera Universitaria Bologna, Bologna, Italy; Alma Mater Studiorum, Università di Bologna, Bologna, Italy
Sollitto	Francesco	Università di Foggia, Azienda ospedaliero-universitaria "Policlinico Riuniti" Foggia, Struttura Complessa di Chirurgia Toracica Universitaria, Foggia
Sorice	Mario	U.O.C. di Chirurgia Toracica, A.O.R.N. "A.Cardarelli" Napoli, Italia
Spaggiari	Lorenzo	Division of Thoracic Surgery, IEO, European Institute of Oncology, IRCCS, Milan, Italy; Department of Oncology and Hemato-Oncology, University of Milan, Milan, Italy
Spinelli	Francesca	University of Milan, Milan, Italy; Department of Thoracic Surgery, Fondazione IRCCS San Gerardo dei Tintori, 20900 Monza, Italy
Sposato	Luciano	Departement of Surgical Sciences, University of Rome Tor Vergata, Via Montpellier 1, 00133, Rome, Itay
Spugnardi	Valeria	U.O.C. Pneumologia—"San Giovanni di Dio e Ruggi D'Aragona" University Hospital, Cava dè Tirreni, Italy
Stella	Franco	Department of Diagnostic and Specialty Medicine, University of Bologna, Thoracic Surgery Unit, Morgagni-Pierantoni Hospital, Forli, Italy
Sterrantino	Sara	Department of Diagnostic and Specialty Medicine, University of Bologna, Thoracic Surgery Unit, Morgagni-Pierantoni Hospital, Forli, Italy
Succu	Angela Maria Pia	Division of Pulmonary Medicine, Sandro Pertini Hospital—Rome (Italy)
Suriano	Ilaria	Department of Thoracic Surgery, Fondazione Policlinico Universitario Campus Bio-Medico, Rome
Tabacco	Diomira	Department of General Thoracic Surgery, Fondazione Policlinico Universitario"A.Gemelli", IRCCS, Università Cattolica del Sacro Cuore, Rome, Italy
Tacchi	Giovanni	Department of Thoracic Surgery, Fondazione Policlinico Universitario Campus Bio-Medico, Rome
Tacconi	Federico	Thoracic Surgery Unit, Tor Vergata University Policlynic, Viale Oxford 81, 00133 Rome, Italy
Tagliaboschi	Linda	Lung Unit, S. Filippo Neri Hospital, ASLROMA1, Rome
Tamburini	Nicola	Arcispedale Sant'Anna, Ferrara, Italy
Tango	Sara	Università di Foggia, Azienda ospedaliero-universitaria "Policlinico Riuniti" Foggia, Struttura Complessa di Chirurgia Toracica Universitaria, Foggia-Italy
Tarsia	Paolo	Department of Pneumology—Grande Ospedale Metropolitano Niguarda—Milan (Italy)
Taurchini	Marco	Division of Thoracic Surgery, SS. Annunziata Hospital, Taranto, Italy
Tedone	Francesca	Thoracic Surgery Unit, Department of Precision and Regenerative Medicine and Ionian Area, University of Bari "Aldo Moro", Bari, Italy
Telha	Valbona	U.O.C. di Chirurgia Toracica, A.O.R.N. "A.Cardarelli" Napoli, Italia
Terminella	Alberto	Thoracic Surgery Unit, University Hospital-Policlinico-San Marco, Catania, Italy
Tiracorrendo	Matteo	Thoracic Surgery Sant'Andrea Hospital, La Sapienza University, Rome, Italy
Todisco	Francesco	University of Torino, AOU Molinette, Italy
Tomaselli	Stefano	Cardiothoracic and Vascular Department, Unit of Respiratory Diseases, IRCCS Policlinico San Matteo, Pavia, Italy
Tomassetti	Sara	Pneumologia Interventistica, AOU Careggi, Firenze
Tornese	Andrea	San Camillo Forlanini Hospital, Rome, Italy
Torre	Massimo	Department of Thoracic Surgery, ASST Grande Ospedale Metropolitano Niguarda, Milano, Italy
Trabalza Marinucci	Beatrice	Thoracic Surgery Sant'Andrea Hospital, La Sapienza University, Rome, Italy
Treggiari	Stefano	San Camillo Forlanini Hospital, Rome, Italy
Trequattrini	Tiziana	Division of Pulmonary Medicine, Sandro Pertini Hospital—Rome (Italy)
Trigiani	Marco	Università degli Studi di Firenze - Azienda Ospedaliero Universitaria di Careggi
Triolo	Luca	Lung Unit, S. Filippo Neri Hospital, ASLROMA1, Rome

Triumbari	Elizabeth Katherine Anna	Nuclear medicine Unit, G-STeP Radiopharmacy Research Core Facility, Department of Radiology, Radiotherapy and Hematology, Fondazione Policlinico Universitario A.Gemelli IRCCS, Università Cattolica del Sacro Cuore, Rome, Italy
Troian	Marina	Thoracic Surgery Unit, Cattinara University Hospital, Trieste, Italy
Tropea	Francesco	Department of Health Sciences, University “Magna Græcia” of Catanzaro, Catanzaro, Italy
Tuoro	Antonio	Division of Thoracic Surgery, Fondazione IRCCS San Gerardo dei Tintori, Monza, Italy
Ubaldi	Martina	University of Roma La Sapienza, AOU Sant’Andrea, Italy
Ugolini	Sara	Barts Thorax Centre, St Bartholomew’s Hospital, Barts Health NHS Foundation Trust, London, UK
Urgese	Annalucia	Thoracic Surgery Unit, Vito Fazzi Hospital, Lecce
Uzunhan	Yurdagül	AP-HP, Hôpitaux Universitaires Paris Seine-Saint-Denis, Hôpital Avicenne, Pneumologie, Université Sorbonne Paris Nord, Faculté de Médecine SMBH, Bobigny, France
Valentini	Mariangela	Unit of Thoracic Surgery, Department of Precision and Regenerative Medicine and Ionian Area, University of Bari, Bari, Italy
Valentini	Leonardo	Division of Thoracic Surgery, IRCCS Azienda Ospedaliera Universitaria Bologna, Bologna, Italy; Alma Mater Studiorum, Università di Bologna, Bologna, Italy
Vanni	Camilla	Thoracic Surgery Sant’Andrea Hospital, La Sapienza University, Rome, Italy
Vannucci	Jacopo	Thoracic Surgery, University of Perugia; Perugia
Vanoni	Nicolo’ Maria	UOC Pneumologia AST Macerata, Ospedale Generale di Macerata (Italy)
Vaquer	Sara	University of Milan, Milan, Italy; Department of Thoracic Surgery, Fondazione IRCCS San Gerardo dei Tintori, 20900 Monza, Italy
Vaz Sousa	Rita	UOC Chirurgia Toracica e Trapianti di Polmone, Dipartimento di Chirurgia Generale e Specialistica, Sapienza Università di Roma
Ventura	Vittoria	UOC Respiratory diseases, Medical, Surgical and Neurochirigical Sciences—University of Siena
Venuta	Federico	Department of Thoracic Surgery and Lung Transplantation, Sapienza University of Rome, Italy
Vermi	Morgana	UOC Pneumologia Mirandola ASL Modena (Italy)
Veronesi	Giulia	Division of thoracic surgery, IRCCS San Raffaele Scientific Institute, Milan, Italy; Università Vita-Salute San Raffaele, Milan, Italy
Veroux	Pierfrancesco	Vascular surgery Unit, University Hospital-Policlinico-San Marco, Catania, Italy
Verzeletti	Vincenzo	Padova University Hospital—Thoracic Surgery Unit, Padova, Italy
Viani	Magda	UOC Respiratory diseases, Medical, Surgical and Neurochirigical Sciences—University of Siena
Vicario	Giuseppe	UOC Chirurgia Toracica Università degli studi della Campania ‘Luigi Vanvitelli’, Napoli
Vicidomini	Giovanni	UOC Chirurgia Toracica Università degli studi della Campania ‘Luigi Vanvitelli’, Napoli
Vigliarolo	Rossana	Division of Pulmonary Medicine, Sandro Pertini Hospital—Rome (Italy)
Vinciguerra	Riccardo	UOC Chirurgia Toracica Università degli studi della Campania ‘Luigi Vanvitelli’, Napoli
Virdis	Erika	Department of Medicine, Surgery and Pharmacy, University of Sassari, Sassari, Italy
Viscardi	Stefano	Department of Thoracic Surgery, IRCCS Scientific Institute San Raffaele, Milan
Vita	Maria Letizia	Department of Thoracic Surgery, Fondazione Policlinico Universitario A.Gemelli IRCCS, Università Cattolica del Sacro Cuore, Rome, Italy
Voulaz	Emanuele	Thoracic Surgery Unit, Humanitas Hospital, Milan, Italy
Waller	David	Barts Thorax Centre, St Bartholomew’s Hospital, Barts Health NHS Foundation Trust, London, UK
Zacchini	Beatrice	UOC Chirurgia Toracica e Trapianti di Polmone, Dipartimento di Chirurgia Generale e Specialistica, Sapienza Università di Roma
Zanardo	Serena	Department of Thoracic Surgery, Ospedale Santa Maria della Misericordia, Udine, Italy
Zanfrini	Edoardo	Service of Thoracic Surgery, University Hospital of Lausanne, Lausanne, Switzerland
Zappa	Maria Cristina	Division of Pulmonary Medicine, Sandro Pertini Hospital—Rome (Italy)
Zhurda	Maria Luisa	Thoracic Surgery Unit, Department of Precision and Regenerative Medicine and Ionian Area, University of Bari “Aldo Moro”, Bari, Italy
Zirafa	Carmelina Cristina	Minimally Invasive and Robotic Thoracic Surgery—Surgical, Medical, Molecular and Critical Care Pathology Department, University Hospital of Pisa
Zito	Anna	Unità Operativa Complessa di Nefrologia, Ospedale Vito Fazzi, Lecce
Zolezzi	Alberto	UOC Malattie infettive dell’apparato respiratorio, IRCCS INMI Spallanzani-Rome (Italy)
Zuin	Andrea	Department of Thoracic Surgery, Ospedale Santa Maria della Misericordia, Udine, Italy
